# Allogeneic blood transfusion and prognosis following total hip replacement: a population-based follow up study

**DOI:** 10.1186/1471-2474-10-167

**Published:** 2009-12-29

**Authors:** Alma B Pedersen, Frank Mehnert, Soren Overgaard, Soren P Johnsen

**Affiliations:** 1 Department of Clinical Epidemiology, Aarhus University Hospital, Olof Palmes Allé 43-45, 8200 Aarhus N, Denmark; 2 Department of Orthopedic Surgery, Odense University Hospital, Sdr. Boulevard 29, 5000 Odense, Denmark

## Abstract

**Background:**

Allogeneic red blood cell transfusion is frequently used in total hip replacement surgery (THR). However, data on the prognosis of transfused patients are sparse. In this study we compared the risk of complications following THR in transfused and non-transfused patients.

**Methods:**

A population-based follow-up study was performed using data from medical databases in Denmark. We identified 28,087 primary THR procedures performed from 1999 to 2007, from which we computed a propensity score for red blood cell transfusion based on detailed data on patient-, procedure-, and hospital-related characteristics. We were able to match 2,254 transfused with 2,254 non-transfused THR patients using the propensity score.

**Results:**

Of the 28,087 THR patients, 9,063 (32.3%) received at least one red blood cell transfusion within 8 days of surgery. Transfused patients had higher 90-day mortality compared with matched non-transfused patients: the adjusted OR was 2.2 (95% confidence interval (CI): 1.2-3.8). Blood transfusion was also associated with increased odds of pneumonia (OR 2.1; CI: 1.2-3.8), whereas the associations with cardiovascular or cerebrovascular events (OR 1.4; CI: 0.9-2.2) and venous thromboembolism (OR 1.2; CI: 0.7-2.1) did not reach statistical significance. The adjusted OR of reoperation due to infection was 0.6 (CI: 0.1-2.9).

**Conclusions:**

Red blood cell transfusion was associated with an adverse prognosis following primary THR, in particular with increased odds of death and pneumonia. Although the odds estimates may partly reflect unmeasured bias due to blood loss, they indicate the need for careful assessment of the risk versus benefit of transfusion even in relation to routine THR procedures.

## Background

Primary total hip replacement (THR) is a common surgical procedure in developed countries, and incidence rates appear to be increasing [1-3]. Although THR surgery is an elective procedure (i.e. there is usually time to haemodynamically stabilize the patient preoperatively), it is associated with blood loss resulting in transfusion rates between 30-80% [4,5]. Considerable evidence points to an increase in the risk of serious complications and death in critically ill patients who are transfused, especially in patients who are undergoing cardiac surgery [6-9]. However, data is limited on the safety of blood transfusion among orthopaedic patients, including patients undergoing THR, which are considered to be healthier patients than persons from the general population. Most [10-14] but not all [15] previous studies on orthopaedic patients have reported an increased risk of postoperative infections including pneumonia, short-term mortality, length of hospital stay, intensive care unit stay, and systemic inflammatory response syndrome in patients receiving blood transfusion(s). Interpretation of published findings is limited by various methodological shortcomings, such as selected and heterogeneous study populations (e.g. single-institution, only patients over 60 years of age, or a mixture of hip and knee replacement, trauma and hip fracture patients), retrospective data collection, incomplete follow-up, insufficient confounder control, and small sample sizes.

The objective of this large population-based follow-up study was to determine whether allogeneic red blood cell transfusion was associated with increased odds of complications following THR. Complications recorded included hospitalization with cardiovascular and/or cerebrovascular events, venous thromboembolism, pneumonia, reoperation due to infection of primary THR, and mortality within 90 days of primary THR.

## Methods

### Study population and settings

The National Board of Health and The Danish Data Protection Agency approved the study. The present study was conducted among patients undergoing primary THR at hospitals in the Danish counties of North Jutland, Aarhus, Funen, and Copenhagen, encompassing 20 orthopaedic departments. These 20 departments serve approximately 45% of the Danish population (nearly 2.3 million people).

Patients were identified via the Danish Hip Arthroplasty Registry (DHR), a registry of all primary total hip replacements and revisions performed in Denmark since 1995. Since its establishment, the DHR has recorded 94% of all procedures performed at 45 orthopaedic departments [16]. The recorded data, including preoperative, perioperative, and postoperative data, were collected prospectively by the operating surgeon using standardized forms.

We first identified primary THR procedures registered in the DHR and performed at the hospitals reporting to the Danish Transfusion Database from 1 January 1999 to 31 December 2007 (n = 28,709). Patients without possibility for follow up (n = 90) and bilateral primary THR procedures performed during the same surgery (n = 532 corresponding to 266 patients) were excluded. However, we did not exclude patients who sustained right and left THR during the study period as long as surgery was not performed on the same day; those patients were treated as independent observations. In total, data for 28,087 primary THR procedures were available for further analyses.

The Danish National Health Service provides tax-supported healthcare for the entire population, guaranteeing free access to family physicians and public hospitals. Unambiguous linkage between various registers can be performed by means of the civil registration number, a unique permanent personal identification number given to all Danish citizens.

### Data on allogeneic red blood cell transfusions

The Danish Transfusion Database is a national clinical registry monitoring the use of blood components. The database retains data on all blood transfusions administered at the included hospitals during the study period and includes information on the civil registration number of the patient receiving the specific blood component, types and number of blood components administered to the patient, date of delivery of the blood component from the blood bank, and clinical biochemical data. Via the Transfusion Database, data was obtained on all allogeneic transfusions of red blood cells administered to included patients within eight days of primary THR surgery. Patients were classified as having received either none or one or more units. Haemoglobin concentrations measured preoperatively and at the time of discharge, were also obtained from the Transfusion Database.

### Data on patient outcomes

The five following transfusion-related complications were defined as outcomes if they occurred within 90 days of the primary THR procedure: 1) death, 2) hospitalization with cardiovascular events including myocardial infarction, congestive heart failure, peripheral vascular disease, or cerebrovascular events, 3) hospitalization with venous thromboembolism, including deep venous thrombosis and/or pulmonary embolism, 4) hospitalization with pneumonia, and 5) reoperation due to infection. Data on death were obtained from the Civil Registration System which has kept daily updated electronic records on any change of address, date of emigration, and date of death for the entire Danish population since 1968 [17]. Data on hospitalizations were identified in the Danish National Registry of Patients, which has kept information on the dates of all admissions and discharges including up to 20 diagnoses for every discharge from non-psychiatric hospitals in Denmark since 1977. Diagnoses are classified according to the Danish version of the International Classification of Diseases (ICD). The eighth edition (ICD-8) was used from 1977 to 1993 and the tenth edition (ICD-10) has been used hereafter. The physician who discharged the patient assigned all discharge diagnoses. Data on reoperations due to infection were obtained from the DHR.

### Statistics

#### Propensity score matching

In order to overcome bias due to confounding, we matched patients receiving red blood cell transfusion to patients not receiving transfusion with a 1:1 ratio using propensity score matching [18,19]. Thus, we identified a set of transfused and non-transfused THR patients who had a similar baseline chance of being transfused. For this purpose we computed a propensity score for each patient using logistic regression. The propensity score reflected the probability of receiving a red cell transfusion given the individual patient's covariate values. Covariates (n = 69) included into the propensity score have been chosen because they are supposed to be used by physicians in the decision making regarding the use of red blood cell transfusion or not, according to current transfusion guidelines, previous literature, and univariate analyses in our study. Diagnosis for primary THR was categorised as primary arthrosis, sequelae after trauma (i.e. fresh fracture and late sequelae from proximal femur fracture, fracture of acetabulum, and traumatic hip dislocation), and other diseases. Using the Danish National Registry of Patients, we extracted data on the 18 major comorbidities before primary THR for each patient, which are generally used for the construction of the Charlson comorbidity index score translated from corresponding ICD-8 and ICD-10 hospital discharge codes [20].

Then, using a macro (available at: http://www2.sas.com/proceedings/sugi26/p214-26.pdf), we matched each case (THR patients who received transfusion) to a unique control (THR patients who did not receive transfusion) on the propensity score alone. Thus, we started with a 5-digit match, and if this could not be done, we then continued to a 4-, 3-, 2-, or 1-digit match. For data regarding the quality of matches see Additional file 1. Unmatched transfused patients were excluded. We were able to match 2254 transfused THR patients to 2254 unique non-transfused THR patients.

We evaluated the balance of the covariates between the two treatment groups before and after matching using standardized mean differences. A standardized mean difference that exceeds 0.1 is indicative of significant imbalance between groups.

Information on blood loss during the surgery, which is an important predictor for transfusion, was not available in our dataset. Information on smoking status, obesity/body-mass index (BMI), prior history of transfusion, pre-operative history of increased perioperative bleeding, and pre-operative history of chronic anaemia was also not available and thus not included in the propensity matching score.

#### Analyses

The study population was followed from the day of primary THR to the occurrence of death, hospitalization for cardiovascular or cerebrovascular events, venous thromboembolism, pneumonia, reoperation due to infection, or 90 days after surgery. We used multivariate logistic regression analysis to assess the association between transfusion and later outcome by computing odds ratios (OR) and 95% confidence interval (CI) as a measure of relative risk. We also adjusted for the haemoglobin concentration 1-7 days postoperative as a surrogate measure of blood loss.

Subgroup analyses were done according to history of previous hospitalization with cardiovascular events (i.e. myocardial infarction, congestive heart failure, cerebrovascular, and peripheral vascular disease) and postoperative haemoglobin level (below or above 105 g/l within 7 days of surgery). Because of the relatively small number of events for each outcome, we used a composite risk estimate combining all five outcomes in the subgroup analyses.

Finally, we performed dose-response analyses on the association between number of transfusions and the composite outcome.

All statistical analyses were performed using SAS software (Version 9.1.3; SAS Institute Inc, Cary, NC).

## Results

Of the 28,087 THR procedures, 9,063 (32.3%) received ≥ 1 red cell transfusion during or within 8 days of primary THR procedure. The median number of red cell units per patient transfused was 2 (range: 1 to 20).

Compared with non-transfused patients, transfused patients were older and had more comorbid conditions (table 1). Transfused patients were also more likely to be female, to receive a cemented prosthesis, and to have a THR procedure of more than two hours. Thus, there were substantial differences in the patient characteristics between transfused and non-transfused patients (table 1).

**Table 1:** Descriptive characteristics of all total hip replacement (THR) patients according to red blood cell transfusion within 8 days of surgery.

	Transfusion n = 9,063	No transfusion n = 19,024	Standardized mean difference***
**Age (years)**			

10-49	338 (3.7%)	1363 (7.2%)	0.18797

50-59	834 (9.2%)	3162 (16.6%)	0.22038

60-69	2098 (23.2%)	6334 (33.3%)	0.18585

70-79	3189 (35.2%)	5957 (31.3%)	0.11451

+80	2604 (28.7%)	2208 (11.6%)	0.42107

**Sex**			

Female	6440 (71.1%)	10450 (54.9%)	0.36876

**Primary hip diagnosis**			

Primary arthrosis	5646 (62.3%)	15588 (81.9%)	0.40642

Trauma	2546 (28.1%)	1628 (8.6%)	0.48898

Other	871 (9.6%)	1808 (9.5%)	0.02348

**Comorbidity history (yes)**			

Myocardial infarction	491 (5.4%)	717 (3.8%)	0.07073

Congestive heart failure	612 (6.8%)	600 (3.2%)	0.17886

Peripheral vascular disease	442 (4.9%)	645 (3.4%)	0.05825

Cerebrovascular disease	990 (10.9%)	1010 (5.3%)	0.22239

Dementia	165 (1.8%)	106 (0.6%)	0.11135

Chronic pulmonary disease	815 (9.0%)	1227 (6.5%)	0.09349

Connective tissue disease	679 (7.5%)	972 (5.1%)	0.11434

Peptic ulcer disease	654 (7.2%)	745 (3.9%)	0.15010

Mild liver disease	167 (1.8%)	214 (1.1%)	0.05590

Diabetes (type I and II)	544 (6.0%)	764 (4.0%)	0.08144

Hemiplegia	19 (0.2%)	19 (0.1%)	0.01715

Moderate to severe renal disease	236 (2.6%)	229 (1.2%)	0.13977

Diabetes with end organ damage	274 (3.0%)	326 (1.7%)	0.10146

Any tumor	1151 (12.7%)	1637 (8.6%)	0.16577

Leukemia	36 (0.4%)	35 (0.2%)	0.05114

Lymphoma	69 (0.8%)	76 (0.4%)	0.06085

Moderate to severe liver disease	63 (0.7%)	39 (0.2%)	0.07666

Metastatic solid tumor	141 (1.6%)	121 (0.6%)	0.11523

**Fixation technique**			

Cemented	4060 (44.8%)	6170 (32.4%)	0.26193

Cementless	2118 (23.4%)	7486 (39.4%)	0.37578

Hybrid	2885 (31.8%)	5368 (28.2%)	0.09093

**Type of anesthesia**			

Regional	5929 (65.4%)	14622 (76.9%)	0.29165

Universal and Combined	3134 (34.6%)	4402 (23.1%)	0.29165

**Duration of surgery (minutes)**			

0-60	1819 (20.1%)	7439 (39.1%)	0.34220

61-120	6199 (68.4%)	11097 (58.3%)	0.10558

>121	1045 (11.5%)	488 (2.6%)	0.34021

20 hospitals performing the THR procedures*			

Prophylaxis for heterotopic bone formations **			

Yes	600 (6.6%)	1317 (6.9%)	0.01945

No	8463 (93.4%)	17707 (93.1%)	0.02058

**Year of surgery**			

1999	114 (1.3%)	115 (0.6%	0.10403

2000	731 (8.1%)	764 (4.0%)	0.11245

2001	623 (6.9%)	1353 (7.1%)	0.05878

2002	1123 (12.4%)	1757 (9.2%)	0.19310

2003	989 (10.9%)	1669 (8.8%	0.13441

2004	1103 (12.2%)	2570 (13.5%)	0.01450

2005	1410 (15.6%)	3148 (16.6%)	0.08021

2006	1579 (17.4%)	3820 (20.1%)	0.19120

2007	1390 (15.4%)	3828 (20.1%)	0.12261

Data are shown in number (%) unless otherwise specified. * Each of the 20 hospitals performing the THR procedures was treated as separate covariate. ** Anti -rheumatic drugs were used as prophylaxis for heterotopic bone formation. *** A standardized mean difference that exceeds 0.1 is indicative of significant imbalance between groups.

### Analyses of the propensity-matched population

Covariates included into the propensity score are presented in table 2. Using the propensity score matching method (see Additional file 2), we identified a population of transfused and non-transfused patients with no substantial differences for any of the characteristics related to the risk of transfusion (table 2). The patients who were matched in the propensity score did not differ substantially from the unmatched excluded patients in any of the covariates included in the propensity score.

**Table 2:** Descriptive characteristics of propensity score matched total hip replacement (THR) patients according to red blood cell transfusion within 8 days of surgery.

	Transfusion n = 2,254	No transfusion n = 2,254	Standardized mean difference***
**Age (years)**			

10-49	146 (6.5%)	159 (7.1%)	0.07561

50-59	272 (12.1%)	256 (11.4%)	0.03827

60-69	602 (26.7%)	635 (28.2%)	0.07124

70-79	830 (36.8%)	793 (35.2%)	0.00596

+80	404 (17.9%)	411 (18.2%)	0.18397

**Sex**			

Female	1,490 (66.1%)	1,439 (63.8%)	0.11293

**Primary hip diagnosis**			

Primary arthrosis	1,647 (73.1%)	1,633 (72.5%)	0.23293

Trauma	323 (14.3%)	319 (14.2%)	0.28662

Other	284 (12.6%)	302 (13.4%)	0.00682

**Comorbidity history (yes)**			

Myocardial infarction	104 (4.6%)	113 (5.0%)	0.02865

Congestive heart failure	116 (5.2%)	125 (5.6%)	0.08273

Peripheral vascular disease	97 (4.3%)	95 (4.2%)	0.02495

Cerebrovascular disease	172 (7.6%)	172 (7.6%)	0.11255

Dementia	15 (0.7%)	8 (0.4%)	0.10249

Chronic pulmonary disease	192 (8.5%)	176 (7.8%)	0.06104

Connective tissue disease	169 (7.5%)	161 (7.1%)	0.02309

Peptic ulcer disease	129 (5.7%)	129 (5.7%)	0.07967

Mild liver disease	37 (1.6%)	30 (1.3%)	0.04168

Diabetes (type I and II)	134 (5.9%)	115 (5.1%)	0.05238

Hemiplegia	4 (0.2%)	6 (0.3%)	0.03791

Moderate to severe renal disease	55 (2.4%)	60 (2.7%)	0.06410

Diabetes with end organ damage	61 (2.7%)	47 (2.1%)	0.06301

Any tumor	260 (11.5%)	235 (10.4%)	0.09151

Leukemia	10 (0.4%)	6 (0.3%)	0.04010

Lymphoma	18 (0.8%)	14 (0.6%)	0.04218

Moderate to severe liver disease	11 (0.5%)	12 (0.5%)	0.03710

Metastatic solid tumor	39 (1.7%)	26 (1.2%)	0.08036

**Fixation technique**			

Cemented	1,186 (52.6%)	1,175 (52.1%)	0.03568

Cementless	523 (23.2%)	522 (23.2%)	0.05074

Hybrid	545 (24.2%)	557 (24.7%)	0.01191

**Type of anesthesia**			

Regional	1,753 (77.8%)	1,757 (78.0%)	0.18378

**Duration of surgery (minutes)**			

0-60	418 (18.5%)	426 (18.9%)	0.09806

61-120	1,685 (74.8%)	1671 (74.1%)	0.03567

>121	151 (6.7%)	157 (7.0%)	0.20997

20 hospitals performing the THR procedures*			

Prophylaxis for heterotopic bone formation **			

Yes	195 (8.7%)	208 (9.2%)	0.06109

No	2059 (91.4%)	2046 (90.7%)	0.06268

**Preoperative Hemoglobin concentration within three months prior to surgery**			

<138.5 g/L	1410 (62.6%)	1362 (60.4%)	0.25041

>138.5 g/L	844 (37.4%)	892 (39.6%)	0.25041

**Year of surgery**			

1999	61 (2.7%)	58 (2.6%	0.03572

2000	60 (2.7%)	56 (2.5%)	0.06520

2001	94 (4.2%)	93 (4.1%)	0.02234

2002	309 (13.7%)	333 (14.8%)	0.02030

2003	239 (10.6%)	254 (11.3%	0.00876

2004	377 (16.7%)	392 (17.4%)	0.04756

2005	347 (15.4%)	333 (14.8%)	0.01024

2006	435 (19.3%)	411 (18.2%)	0.01185

2007	332 (14.7%)	324 (14.4%)	0.00976

Data are shown in number (%) unless otherwise specified. *Good balance between groups for each of the 20 hospitals included in the propensity score was achieved in general. ** Anti -rheumatic drugs were used as prophylaxis for heterotopic bone formation. *** A standardized mean difference that exceeds 0.1 is indicative of significant imbalance between groups.

**Figure 1:**
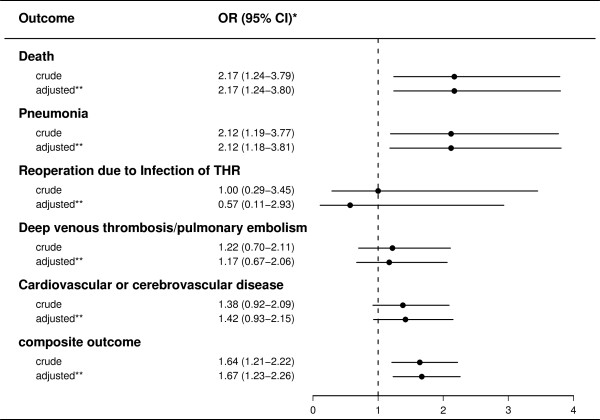
**Crude and adjusted odds ratios of adverse outcomes within 90 days of primary total hip replacement (THR) associated with red blood cell transfusion among propensity score matched patients**. Submitted and labelled separately. Legend: * odds ratio with 95% confidence interval. ** odds ratio adjusted for haemoglobin concentration 1-7 days postoperative. Reference group is non-transfused patients.

Table 3 shows the absolute risk of each of the outcomes in the propensity score matched population. Figure 1 summarises the crude and adjusted odds ratios for each outcome for 2,254 transfused patients and 2,254 propensity score matched non-transfused patients. The cumulative 90-day mortality was 1.7% among transfused patients versus 0.8% among non-transfused patients, corresponding to an adjusted OR of 2.2 (95% CI: 1.2-3.8). For hospitalization with pneumonia, there was also increased OR for transfused patients compared with non-transfused patients (adjusted OR 2.1, 95% CI: 1.2-3.8). Transfused patients had non-significant increased odds of hospitalization with cardiovascular and cerebrovascular events (adjusted OR 1.4, 95% CI: 0.9-2.2) and venous thromboembolism (adjusted OR 1.2, 95% CI: 0.7-2.1), whereas the odds of reoperation due to THR infection was 0.6 (95% CI: 0.1-2.9) compared with non-transfused patients.

**Table 3:** Number of adverse events within 90 days after primary total hip replacement (THR) among propensity score matched patients who were eligible for red blood cell transfusion.

	Propensity score matching model	
**Outcome**	**Transfusion** **n = 2,254**	**Non-transfusion** **n = 2,254**

**Death**	39 (1.7%)	18 (0.8%)

**Cardiovascular or cerebrovascular disease**	54 (2.4%)	39 (1.7%)

**Deep venous thrombosis and/or pulmonary embolism**	28 (1.2%)	23 (1.0%)

**Pneumonia**	36 (1.6%)	17 (0.8%)

**Reoperation due to Infection of THR**	5 (0.2%)	5 (0.2%)

Patients with the most transfusions (6 or more) had the highest odds of a composite adverse outcome (adjusted OR 3.4, 95%CI: 1.3-9.2) compared with non-transfused patients. However, the increased odds were not restricted to patients receiving many transfusions, as patients receiving only 1 transfusion had increased odds of an adverse outcome (adjusted OR 2.7, 95% CI: 1.2-5.7). Patients who received 2-3 or 4-5 transfusions had nonsignificant increased odds for the composite outcome of 1.2 (95% CI: 0.8-1.9) and 1.5 (95% CI: 0.8-2.9), respectively.

### Subgroup analyses

Using the same covariates included in the propensity score matching (table 2), we were able to match 407 transfused THR procedures to 407 unique non-transfused THR procedures, all with a history of cardiovascular events, and 1,834 transfused THR patients to 1,834 unique non-transfused THR patients, all without a history of cardiovascular events. For THR patients with a history of cardiovascular events prior to surgery, red blood cell transfusion was associated with odds of the composite adverse outcome within 90 days of surgery of 1.2 (CI: 0.8-1.9) compared with non-transfused patients. In THR patients without cardiovascular events prior to surgery, the odds of the composite outcome was 1.7 (CI: 1.1-2.7) among transfused versus non-transfused patients.

Using the same covariates included in the propensity score matching (table 2), we matched 729 transfused THR patients to 749 unique non-transfused THR patients, all with a postoperative haemoglobin concentration below 105 g/L, and 1,505 transfused THR patients to 1,505 unique non-transfused THR patients, all with postoperative haemoglobin concentration of greater than 105 g/L. Among THR patients with postoperative haemoglobin below 105 g/L, the adjusted OR for the composite adverse outcome within 90 days of surgery was 1.1 (CI: 0.5-2.7) for transfused patients compared with non-transfused patients. In contrast, among THR patients with a postoperative haemoglobin level above 105 g/L, the adjusted OR for composite outcome within 90 days of surgery was 1.6 (CI: 1.0-2.5) for transfused compared with non-transfused patients.

## Discussion

In this large population-based follow-up study of primary THR patients, we found that red blood cell transfusion was associated with substantially increased odds of an adverse outcome, in particular death and pneumonia.

### Strengths and limitations

Strengths of the present study include the large sample size and the population-based design with complete follow-up for all patients included in the study population. All data were prospective collected independently of the objective of our study. Since the study population was identified only after collection of data had ended, this is a retrospective (or historical) population-based cohort study. The DHR database had a high validity [16]. The quality of registration of diagnoses in the Danish National Registry of Patients has also been established [21-24]. Nevertheless, any misclassification and errors of diagnosis codes may bias our findings if related to transfusion. Further, our estimates would be biased if some hospitalizations with specific outcomes were based on symptoms arising after transfusion versus pre-transfusion. Data on red blood cell transfusions are directly drawn from the blood bank systems in which registration of all blood products is mandatory according to Danish law. The number of transfusions registered in the Danish Transfusion Database is in accordance with the official statistics on use of blood products in Denmark reported by the Danish Medicines Agency [25].

The major methodological concern in observational studies on medical interventions, including transfusion, is the obvious risk of bias due to confounding; this is also the case in our study. Detailed and complete data on patient- and procedure-related characteristics were available and thorough efforts were made, including a combination of propensity score matching and multivariable adjustment techniques, to minimize any impact of confounding on the results. Although the covariates included in the propensity score were chosen based on the current guidelines for transfusion and literature reporting on factors affecting the decision whether to transfuse or not, we don't know to what degree these covariates are truly transfusion triggers in Danish orthopaedic departments or to what extent physicians follow existing guidelines. We reported previously on substantial differences in the use of red blood cell transfusion among THR patients when comparing a sample of Danish orthopaedic departments, which could not be explained by a range of patient- and surgery-related factors [26]. Further, we included patients with a preoperative haemoglobin level above 105 g/L that were transfused, which seems to deviate from current guidelines. However, as we don't have data on blood loss during surgery, the surgeon may still have followed the guidelines if these patients suffered blood loss during surgery and received transfusion afterwards. Information on pre- and postoperative haemoglobin concentration was further included as a surrogate measure of blood loss. We cannot exclude the possibility that residual confounding due to the use of crude variables (e.g. the severity of comorbidities, or pre-operative haemoglobin level above or below 138.5 g/L only) or unknown/unmeasured prognostic factors (e.g. exact volume of blood loss during the surgery which could potentially be the actual driver of adverse outcomes or life style factors) may have influenced the results. Data on use of autologous red blood cell transfusions and leukoreduction of transfusions were not available.

### Comparison with other studies

Our findings regarding the increased odds of death within 90 days after THR are consistent with several studies on critically ill patients, orthopaedic trauma patients, hip fracture patients, and patients undergoing cardiac surgery [6-9,13,15,27]. THR patients are generally considered to be a patient group with a low mortality compared with the general population, and somehow, transfusion adversely affected this relationship [28].

An association between transfusion and increased odds of postoperative vascular complications has previously been found among patients undergoing cardiac surgery and among patients admitted to intensive care units [8,27,29]. A study of patients undergoing total hip or knee replacement showed that fluid overload is a common complication following allogeneic blood transfusion, which agrees with our results [11]. Nevertheless, the results are not directly comparable since diuretic consumption was used as a surrogate measure for fluid overload and a cardiovascular event, and the study by Bierbaum and colleagues did not take underlying comorbidities and other confounding factors into account. Although the mechanism linking transfusion with cardiac complications remains unclear, several factors may contribute. These factors include augmentation of the inflammatory response responsible for the release of inflammatory mediators, or vasoconstrictors that may facilitate plaque rupture and subsequent thrombosis [30]. Another likely explanation is that a patient's underlying heart condition combined with multiple, rapid blood transfusions lead to pulmonary oedema. However, in our study we found increased odds of adverse outcome for patients with and without a history of cardiovascular disease, although the odds were only statistical significant in the latter group.

Increased odds of pneumonia in transfused versus non-transfused patients is consistent with the results of some, but not all previous studies [11,14,31]. These inconsistent results may be due to limited sample size, inhomogeneous study populations, and surgery involving different joints. In addition, previous studies have reported on the risk of postoperative infection in general, including pneumonia, wound infection, urinary infection, and bacteraemia, rather than the risk of pneumonia only. This makes it difficult to directly compare the results of previous studies to one another and to our data. The mechanism underlying the association between transfusion and subsequent pneumonia has not been established. However, it appears likely that systemic immunosuppression due to transfusion-associated infusion of large amounts of foreign antigens changes the cellular responses of the recipient. Nevertheless, a meta-analysis of 15 randomized controlled trials and 40 observational studies showed no clear link between transfusion-related immunomodulation and risk of postoperative infection [32]. However, it was not possible to reach a conclusion specifically on the risk of developing pneumonia based on the meta-analysis, since data on postoperative pneumonia were not available in all studies. Marik and Corwin published a systematic review based on 45 cohort studies, suggesting that transfusions are associated with increased morbidity and mortality risk in high risk hospitalized patients [33]. It has to be noted that our study is based on allogeneic transfusions, and the findings may differ among patients who received autologous red blood cell transfusions. Traditionally, allogeneic blood transfusion has been much more prevalent in Denmark, and data on autologous transfusions were not available for our study.

Previous publications suggest that haemoglobin is a strong predictor for blood transfusion [34]. Nevertheless, data on preoperative haemoglobin concentration was only available for one third of the transfused THR patients in our study. Furthermore, it has been shown that 75% of total hip arthroplasty patients who received a transfusion were not anaemic preoperatively, suggesting that other factors play a considerable role in the decision to transfuse [35].

We had no information on blood loss during surgery, which may affect prognosis after transfusion and change the quality and focus of our observations if related to transfusion, i.e., if patients who received transfusion had more blood loss than patients who did not receive transfusion. If so, the observed adverse outcomes would be associated with blood loss rather than transfusion. To address this issue we performed stratified analyses on postoperative haemoglobin concentration as a surrogate measure of blood loss. Our observations regarding increased odds of adverse outcome among patients with a high or normal postoperative haemoglobin concentration but not among patients with a low postoperative haemoglobin concentration confirm and extend observations made in different settings. One previous study of coronary surgery patients showed increased mortality and incidence of myocardial infarction in transfused patients with a haemoglobin concentration of more than 90 g/L compared to non-transfused patients [36]. Hebert et al. reported in a randomized clinical trial of critically ill patients that liberal transfusion practice (with a threshold for transfusion of 97 g/L and more) lead to a higher rate of cardiac events within 30 days of admission to the intensive care unit compared to restrictive transfusion practice (i.e., transfusion given when the haemoglobin concentration fell below 70 g/L) [37].

## Conclusions

Allogeneic red blood cell transfusion was associated with an adverse prognosis following primary THR. Although the odds ratio estimates may partly be driven by unmeasured bias due to blood loss during surgery, they indicate a need for further examination of the use of allogeneic blood transfusion in total hip replacement patients. The risk versus benefit of transfusion among THR patients warrants careful assessment.

## Competing interests

The authors declare that they have no competing interests.

## Authors' contributions

ABP participated in the design, interpretation of data, drafting and revising of the manuscript. FM carried out the analyses and critically revised the manuscript. SO participated in interpretation of data and critically revised the manuscript. SPJ participated in the design, interpretation of data, and critically revised the manuscript. All authors read and approved the final manuscript.

## Pre-publication history

The pre-publication history for this paper can be accessed here:

http://www.biomedcentral.com/1471-2474/10/167/prepub

## Supplementary Material

Additional file 1 **Data regarding the quality of matches.pdf**. The table include number of matching pairs and percent of 5-digit matched, 4-digit matched, 3-digit matched, 2-digit matched, and 1-digit matched patients. Click here for file

Additional file 2 **The propensity scores and the number of units transfused per individual patients.pdf**. The file include patient ID, side (1 = right hip and 2 = left hip), propensity score, number of transfusions and matched (0 = no and 1 = yes). Click here for file
