# Supplementary material for: Allogeneic blood transfusion and prognosis following total hip replacement: a population-based follow up study
Source: BMC Musculoskelet Disord. 2009 Dec 29;10:167. doi: 10.1186/1471-2474-10-167 (PMC2805607; doi:10.1186/1471-2474-10-167)
Supplement: Additional file 1 — Data regarding the quality of matches.pdf. The table include number of matching pairs and percent of 5-digit matched, 4-digit matched, 3-digit matched, 2-digit matched, and 1-digit matched patients. [file 1471-2474-10-167-S1.PDF]

The quality of matches

MATCH	Matching pairs	Percent
**1-DIGIT**	90	2.00
**2-DIGIT**	488	10.83
**3-DIGIT**	1098	24.36
**4-DIGIT**	308	6.83
**5-DIGIT**	2524	55.99
