# Supplementary material for: Allogeneic blood transfusion and prognosis following total hip replacement: a population-based follow up study
Source: BMC Musculoskelet Disord. 2009 Dec 29;10:167. doi: 10.1186/1471-2474-10-167 (PMC2805607; doi:10.1186/1471-2474-10-167)
Supplement: Additional file 2 — The propensity scores and the number of units transfused per individual patients.pdf. The file include patient ID, side (1 = right hip and 2 = left hip), propensity score, number of transfusions and matched (0 = no and 1 = yes). [file 1471-2474-10-167-S2.PDF]

Patient ID	Side	Propensity score	Number of tranfusions	Matched
1000001	1	0.9061302	0	0
1000002	1	0.8318556	0	0
1000003	1	0.4663877	4	1
1000004	2	0.9732879	0	0
1000005	2	0.4535902	2	1
1000006	2	0.5422639	0	1
1000007	2	0.9145093	0	0
1000008	2	0.1741055	4	0
1000009	1	0.7717639	1	1
1000010	2	0.6826809	1	1
1000011	2	0.7451837	1	1
1000012	1	0.9796864	0	0
1000013	2	0.7025956	0	0
1000014	2	0.2517358	2	1
1000015	2	0.9527564	0	0
1000016	1	0.7836873	0	0
1000017	1	0.381759	4	1
1000018	2	0.8199402	0	0
1000019	1	0.7511338	0	0
1000020	1	0.7460427	0	0
1000021	1	0.5229135	0	1
1000021	2	0.6977639	0	1
1000022	1	0.9262035	0	0
1000023	2	0.9482519	0	0
1000024	1	0.882295	0	0
1000025	1	0.3185485	12	0
1000026	2	0.9082494	0	0
1000027	2	0.8480624	0	0
1000028	2	0.473773	2	1
1000029	1	0.8590258	0	1
1000030	2	0.8427618	0	0
1000031	1	0.4415582	4	1
1000032	2	0.8807684	0	0
1000033	2	0.9623802	0	0
1000034	1	0.6153635	2	1
1000035	2	0.9017812	0	0
1000036	2	0.8531178	0	0
1000037	1	0.3084504	0	1
1000038	2	0.1136335	3	0

Patient ID	Side	Propensity score	Number of tranfusions	Matched
1000039	2	0.6543063	0	0
1000040	1	0.0785387	4	0
1000041	1	0.6461803	0	1
1000042	1	0.8703418	0	0
1000043	1	0.481933	3	1
1000044	2	0.7059057	2	1
1000044	1	0.8151238	0	0
1000045	1	0.5585162	2	1
1000046	1	0.5716151	0	1
1000047	2	0.7633599	2	1
1000048	2	0.5290465	0	1
1000049	1	0.1319859	2	0
1000050	2	0.3065288	0	1
1000051	2	0.2913552	2	0
1000052	2	0.9380204	0	0
1000053	1	0.9277478	0	0
1000054	2	0.5737743	0	1
1000055	1	0.559449	0	1
1000056	1	0.6626588	1	1
1000057	1	0.6987217	0	1
1000058	2	0.9639877	0	0
1000059	2	0.810253	2	1
1000060	2	0.875534	0	1
1000061	2	0.5350229	13	1
1000062	1	0.8380845	0	0
1000063	2	0.7196551	0	1
1000064	1	0.9395485	0	1
1000065	1	0.9546245	2	1
1000066	2	0.9568595	0	0
1000067	2	0.9653663	0	0
1000068	2	0.3915087	1	0
1000069	2	0.3520014	0	1
1000070	2	0.5739017	2	1
1000071	1	0.8627176	0	0
1000072	2	0.8303496	0	0
1000073	1	0.8871914	0	0
1000074	2	0.9684105	0	0
1000075	2	0.6808684	0	1
1000076	1	0.2783631	5	1

Patient ID	Side	Propensity score	Number of tranfusions	Matched
1000077	2	0.8240572	0	0
1000078	1	0.6506863	0	0
1000079	1	0.9712601	0	0
1000080	2	0.3796384	2	1
1000081	2	0.843828	2	1
1000082	1	0.8393318	0	0
1000083	1	0.2648933	0	1
1000083	2	0.2286018	0	1
1000084	1	0.1885288	0	1
1000085	2	0.7470569	2	1
1000086	1	0.8892304	0	0
1000087	2	0.7094563	0	1
1000088	2	0.2797157	5	1
1000089	1	0.8531178	0	0
1000090	1	0.8825185	0	0
1000091	1	0.3004124	3	1
1000092	2	0.8387457	0	0
1000093	1	0.2992102	4	1
1000094	1	0.9339128	0	0
1000095	2	0.2634975	1	0
1000096	2	0.7451044	0	1
1000096	1	0.7451044	0	0
1000097	2	0.2733145	2	0
1000098	1	0.7010806	0	0
1000099	2	0.9186629	0	1
1000100	1	0.9371699	0	0
1000101	2	0.5839917	0	0
1000102	2	0.9380204	0	0
1000102	1	0.8892304	0	0
1000103	1	0.9365261	0	0
1000104	2	0.2658993	1	0
1000105	2	0.9568595	0	0
1000106	2	0.9568595	0	1
1000107	1	0.8410779	2	1
1000108	1	0.9163173	0	0
1000109	2	0.4525465	0	1
1000110	1	0.9000514	0	0
1000111	2	0.7705859	0	0
1000112	1	0.238121	4	0
1000113	2	0.8431585	0	0

Patient ID	Side	Propensity score	Number of tranfusions	Matched
1000114	2	0.9694243	0	0
1000115	2	0.3796384	2	0
1000116	1	0.4516916	5	1
1000117	2	0.7451044	0	1
1000118	1	0.6234114	3	1
1000119	2	0.9653663	0	0
1000120	1	0.6265182	0	1
1000121	1	0.9099991	0	0
1000122	1	0.8923432	0	0
1000123	1	0.815375	0	1
1000124	1	0.9467434	0	0
1000125	2	0.8963693	0	0
1000125	1	0.8963693	0	0
1000126	1	0.6171144	0	1
1000127	1	0.9348254	0	1
1000128	2	0.5740564	2	1
1000129	2	0.9384815	0	1
1000130	1	0.1136646	2	0
1000131	2	0.2317343	0	1
1000132	2	0.7512119	0	0
1000133	1	0.5789374	0	1
1000134	2	0.0986374	7	0
1000135	2	0.2039939	1	0
1000136	2	0.8622236	0	0
1000136	1	0.8622236	0	0
1000137	1	0.8740013	2	1
1000138	1	0.9555191	0	0
1000139	2	0.6608236	0	0
1000140	2	0.941524	0	0
1000141	2	0.6406524	2	1
1000142	2	0.0373787	5	0
1000143	1	0.8825185	0	0
1000143	2	0.9493857	0	0
1000144	2	0.9825048	0	0
1000145	2	0.8788843	0	1
1000146	2	0.3991128	0	1
1000147	1	0.5851313	2	1
1000148	2	0.2816029	0	1
1000149	1	0.3236385	0	1

Patient ID	Side	Propensity score	Number of tranfusions	Matched
1000149	2	0.4435972	2	1
1000150	1	0.9145093	0	0
1000151	2	0.9278036	0	0
1000152	2	0.6790591	0	1
1000153	2	0.9366489	0	0
1000154	1	0.6576642	2	1
1000155	1	0.9256307	0	0
1000156	1	0.4733407	0	1
1000157	2	0.9735961	0	0
1000158	1	0.673649	2	1
1000159	2	0.8923432	0	0
1000160	1	0.8923432	0	0
1000161	2	0.1542122	4	1
1000162	1	0.0986374	2	0
1000163	1	0.7705859	0	0
1000164	2	0.5307699	2	1
1000165	1	0.9086713	0	0
1000166	1	0.559063	3	1
1000167	2	0.8597681	0	0
1000168	2	0.0692887	9	0
1000169	2	0.7736042	0	0
1000170	1	0.9684105	0	0
1000171	2	0.335518	0	1
1000172	2	0.263743	4	1
1000173	2	0.7317822	1	1
1000174	1	0.8722887	0	0
1000175	2	0.9639877	0	0
1000176	2	0.2024319	0	1
1000177	2	0.7139026	0	0
1000178	1	0.5740564	9	1
1000179	2	0.9634501	0	1
1000180	2	0.7451837	0	1
1000181	1	0.2034325	2	1
1000182	1	0.678968	0	0
1000183	1	0.3849963	2	0
1000184	1	0.7451044	0	0
1000185	1	0.7357013	0	0
1000186	2	0.457671	0	1
1000187	2	0.8590341	0	0

Patient ID	Side	Propensity score	Number of tranfusions	Matched
1000188	1	0.9493857	0	0
1000189	1	0.5591499	2	1
1000189	2	0.5591499	0	1
1000190	1	0.9653663	0	0
1000191	2	0.2236856	2	1
1000192	1	0.6363631	0	1
1000193	1	0.9119742	0	0
1000194	2	0.4287977	3	0
1000195	1	0.9639877	0	0
1000196	1	0.74691	0	0
1000197	1	0.6585669	0	0
1000198	1	0.380564	3	0
1000199	1	0.6299819	0	1
1000200	2	0.9557483	0	0
1000201	2	0.7854294	0	0
1000202	2	0.0689089	4	0
1000203	1	0.6444111	2	1
1000204	2	0.3575094	2	0
1000205	2	0.6584128	0	1
1000206	2	0.7663047	0	0
1000207	2	0.9472872	0	0
1000208	2	0.5551149	0	1
1000208	1	0.4218566	0	1
1000209	2	0.6850716	0	1
1000210	1	0.9484148	0	0
1000211	2	0.7216129	0	0
1000212	2	0.7429692	0	0
1000213	2	0.9557483	0	0
1000214	1	0.0924631	11	0
1000215	1	0.3741824	0	1
1000216	2	0.6005852	2	1
1000217	1	0.4037822	2	1
1000218	.	0.0899884	0	1
1000218	1	0.0899884	0	1
1000219	2	0.6829168	2	1
1000219	1	0.7820638	1	1
1000220	1	0.0383943	4	0
1000221	2	0.0626167	0	1
1000222	2	0.7471596	0	0
1000223	2	0.5797566	0	1

Patient ID	Side	Propensity score	Number of tranfusions	Matched
1000224	1	0.8226179	4	1
1000225	1	0.7701729	0	1
1000226	2	0.7853797	3	1
1000226	1	0.7853797	2	1
1000227	2	0.9718675	0	0
1000228	2	0.9653663	0	0
1000229	2	0.801863	0	0
1000230	1	0.6419219	0	1
1000231	1	0.4666292	4	1
1000232	2	0.882295	0	0
1000233	2	0.9309768	0	0
1000234	1	0.7950227	0	1
1000235	1	0.321832	0	1
1000235	2	0.4415582	1	1
1000236	2	0.3530657	2	0
1000237	1	0.317724	13	0
1000238	2	0.4144451	1	1
1000239	2	0.7858006	0	0
1000239	1	0.547296	0	0
1000240	1	0.8151238	1	1
1000241	2	0.9148016	0	0
1000242	2	0.6506863	0	0
1000243	1	0.9380691	0	0
1000244	2	0.8148102	0	0
1000245	1	0.1898562	4	0
1000246	1	0.8652821	0	0
1000247	2	0.8825185	0	0
1000248	2	0.2024319	1	0
1000249	1	0.0273303	2	0
1000250	1	0.3763967	3	1
1000251	2	0.7166007	0	0
1000252	2	0.5211484	2	1
1000253	2	0.6396958	0	1
1000254	1	0.9121083	0	0
1000254	2	0.9398549	0	0
1000255	2	0.9278036	0	0
1000256	2	0.7865697	0	1
1000257	2	0.3253572	0	1
1000258	1	0.9395485	0	0

Patient ID	Side	Propensity score	Number of tranfusions	Matched
1000259	2	0.9364664	0	0
1000260	2	0.8628989	0	0
1000261	1	0.810253	0	0
1000262	1	0.5137705	2	1
1000263	1	0.8956693	1	1
1000264	1	0.5116775	0	1
1000265	1	0.7751945	0	0
1000266	1	0.8825185	3	1
1000267	2	0.56959	0	1
1000268	1	0.8894812	0	0
1000269	1	0.9546245	0	0
1000270	1	0.0833426	7	0
1000271	2	0.8457341	0	0
1000271	1	0.7213635	0	0
1000272	1	0.6006756	0	1
1000273	2	0.2583276	4	0
1000273	1	0.1752803	3	0
1000274	1	0.4866374	0	1
1000274	2	0.4318063	0	1
1000275	1	0.5050592	0	1
1000275	2	0.7833929	0	0
1000276	2	0.9555191	0	0
1000277	2	0.7398223	0	1
1000278	2	0.2145031	5	1
1000279	2	0.8186172	0	1
1000279	1	0.5601314	0	1
1000280	1	0.9095091	0	0
1000281	2	0.806943	0	1
1000282	2	0.9639877	0	0
1000283	2	0.3307463	0	1
1000283	1	0.3250186	0	1
1000284	1	0.8531178	0	0
1000285	1	0.9142818	0	0
1000286	1	0.9468601	0	0
1000287	1	0.321832	0	1
1000288	1	0.3859445	2	1
1000289	1	0.9237913	0	0
1000290	1	0.678968	0	0
1000291	2	0.4754415	2	1

Patient ID	Side	Propensity score	Number of tranfusions	Matched
1000292	2	0.9300016	0	0
1000292	1	0.9637995	0	0
1000293	2	0.6179632	2	1
1000294	1	0.4987304	0	1
1000295	1	0.790328	0	0
1000296	1	0.9723294	0	0
1000297	2	0.810253	0	0
1000298	1	0.9684105	0	0
1000299	2	0.8151135	0	0
1000299	1	0.9262035	4	1
1000300	1	0.4053315	0	1
1000301	1	0.8143077	0	0
1000302	2	0.6179632	0	1
1000303	2	0.5623317	2	1
1000304	1	0.257319	0	1
1000305	2	0.7634533	0	1
1000306	2	0.2575766	2	1
1000307	1	0.882295	0	0
1000308	2	0.6504241	0	1
1000309	2	0.0579848	4	0
1000310	2	0.3042595	0	1
1000311	2	0.957775	0	0
1000312	2	0.1548002	4	0
1000313	2	0.7897282	0	1
1000314	2	0.6299819	2	1
1000315	1	0.8958698	0	0
1000316	2	0.3253572	0	1
1000317	1	0.7664779	0	0
1000318	2	0.4715458	0	1
1000319	2	0.6319744	0	1
1000320	1	0.622981	0	1
1000321	1	0.9557483	0	0
1000322	1	0.8148102	0	0
1000323	1	0.7897282	0	1
1000324	1	0.9512951	0	0
1000325	1	0.5307699	0	1
1000326	2	0.6444111	0	1
1000327	2	0.9222556	0	0
1000328	1	0.9862315	0	0

Patient ID	Side	Propensity score	Number of tranfusions	Matched
1000329	2	0.576835	2	1
1000330	1	0.1505933	2	0
1000331	2	0.5406827	0	1
1000331	1	0.2493241	2	1
1000332	2	0.9569966	0	0
1000333	2	0.9743555	0	0
1000334	2	0.9653663	0	0
1000335	2	0.6343521	2	1
1000336	1	0.7454145	0	0
1000337	1	0.9679403	0	1
1000338	1	0.3697271	4	1
1000339	1	0.8409809	0	0
1000340	1	0.8196841	0	0
1000341	2	0.7451044	0	0
1000342	1	0.7103589	0	1
1000343	1	0.3935633	2	1
1000344	2	0.9557483	0	0
1000345	2	0.6447928	2	1
1000346	1	0.8349239	2	1
1000347	2	0.7797947	0	0
1000347	1	0.7797947	0	1
1000348	1	0.7838778	0	1
1000349	1	0.543217	3	1
1000350	2	0.9680528	0	1
1000351	1	0.6267328	2	1
1000352	1	0.737203	0	1
1000353	1	0.2063098	0	1
1000354	2	0.8894812	0	0
1000355	2	0.9607499	0	0
1000355	1	0.8578785	1	1
1000356	1	0.2972059	3	1
1000357	1	0.8923432	0	0
1000358	1	0.2341951	10	0
1000359	2	0.4044706	0	1
1000360	1	0.7233331	0	0
1000361	2	0.6749625	1	1
1000362	2	0.6349469	0	1
1000362	1	0.6349469	0	0
1000363	1	0.5737743	2	1

Patient ID	Side	Propensity score	Number of tranfusions	Matched
1000363	2	0.9262035	0	0
1000364	2	0.2249803	2	1
1000365	1	0.351637	1	1
1000365	2	0.8892304	0	0
1000366	2	0.9482519	0	0
1000367	2	0.8652821	0	0
1000368	2	0.9557483	0	0
1000369	1	0.1806238	2	1
1000370	1	0.9346629	0	0
1000371	2	0.4044706	4	1
1000372	2	0.3067821	3	0
1000373	2	0.9581994	0	0
1000374	1	0.8379319	0	0
1000375	2	0.2278918	2	1
1000376	1	0.765845	0	0
1000376	2	0.8449489	0	0
1000377	1	0.8541505	0	0
1000378	2	0.8986861	0	0
1000379	1	0.0516718	5	0
1000380	1	0.9239004	0	0
1000381	1	0.7089199	0	1
1000382	2	0.5875321	1	1
1000383	2	0.0442305	10	0
1000384	2	0.9405539	0	1
1000385	2	0.9145093	0	0
1000386	2	0.0893799	3	0
1000387	2	0.5740564	2	1
1000388	2	0.7376414	0	1
1000389	1	0.5135614	0	1
1000389	2	0.7251081	0	0
1000390	1	0.8892304	0	0
1000391	1	0.242314	6	0
1000392	2	0.9557483	0	0
1000393	2	0.9342051	0	0
1000394	2	0.8363182	0	0
1000395	1	0.9380204	0	0
1000396	1	0.7159765	0	0
1000397	2	0.9148016	0	0
1000398	2	0.8143077	0	0
1000399	1	0.0094828	2	0

Patient ID	Side	Propensity score	Number of tranfusions	Matched
1000400	1	0.2550914	2	1
1000401	2	0.8761264	2	1
1000402	2	0.6743881	2	1
1000403	2	0.5308739	2	1
1000403	1	0.6544533	2	1
1000404	1	0.359349	0	1
1000405	2	0.8244078	0	0
1000406	1	0.249751	1	0
1000407	2	0.9565483	0	0
1000407	1	0.9639877	0	0
1000408	1	0.9803312	0	0
1000409	1	0.467087	0	1
1000410	2	0.5482672	0	1
1000411	1	0.8668746	0	1
1000411	2	0.8668746	3	1
1000412	1	0.810253	0	1
1000413	2	0.7827674	0	0
1000414	1	0.7834102	0	1
1000415	2	0.7809334	0	0
1000416	1	0.9471208	0	0
1000417	2	0.0741976	4	0
1000418	1	0.9467011	0	0
1000419	1	0.815375	2	1
1000420	2	0.0724442	2	0
1000421	1	0.8003601	2	1
1000422	1	0.914542	0	0
1000423	2	0.7125284	0	0
1000424	2	0.9694243	0	0
1000425	1	0.3180086	2	0
1000426	2	0.9471208	0	0
1000427	1	0.8848014	2	1
1000428	1	0.4650836	0	1
1000429	2	0.9639877	0	0
1000430	1	0.3826855	0	1
1000431	2	0.8931346	0	0
1000432	1	0.0554506	17	0
1000433	1	0.7010806	0	0
1000434	1	0.8531178	0	0
1000435	1	0.9197193	0	0

Patient ID	Side	Propensity score	Number of tranfusions	Matched
1000435	2	0.9557483	0	0
1000436	1	0.7470569	0	0
1000437	1	0.9004673	0	0
1000438	2	0.5797566	0	1
1000439	1	0.678968	0	0
1000440	1	0.9718675	0	1
1000441	2	0.8565918	0	1
1000442	1	0.4901549	0	1
1000443	2	0.1702515	2	0
1000444	2	0.599822	0	1
1000444	1	0.6897647	0	0
1000445	1	0.5415543	2	1
1000446	2	0.8812618	0	0
1000446	1	0.5116775	0	1
1000447	1	0.8103819	2	1
1000448	1	0.2065761	4	0
1000449	1	0.9781832	0	0
1000450	1	0.0986374	2	0
1000451	1	0.7454145	0	0
1000452	2	0.8159437	0	0
1000453	2	0.9493857	0	0
1000454	1	0.0955726	4	0
1000455	2	0.8684553	6	1
1000455	1	0.9380204	2	1
1000456	2	0.9264305	0	1
1000456	1	0.9264305	0	0
1000457	1	0.6768638	0	1
1000458	1	0.9481091	2	1
1000459	1	0.8615083	0	0
1000460	1	0.3504041	2	0
1000460	2	0.3504041	2	0
1000461	2	0.1683333	6	0
1000462	1	0.8630618	0	0
1000463	1	0.9366489	0	0
1000464	1	0.0532364	4	1
1000465	2	0.9380204	0	0
1000466	1	0.2711451	2	0
1000467	1	0.8103819	0	0
1000468	2	0.9684105	0	0

Patient ID	Side	Propensity score	Number of tranfusions	Matched
1000469	1	0.8604233	0	0
1000470	1	0.9380204	0	0
1000471	1	0.7148756	2	1
1000472	2	0.8861318	17	1
1000472	1	0.8861318	6	1
1000473	1	0.8720679	2	1
1000474	1	0.914542	0	0
1000475	2	0.153223	0	1
1000476	1	0.9527564	0	0
1000477	2	0.0532364	5	0
1000478	2	0.2236856	5	1
1000479	2	0.9043188	0	0
1000480	1	0.8851865	0	0
1000480	2	0.8825185	0	1
1000481	1	0.9684105	0	0
1000481	2	0.9568595	0	0
1000482	1	0.6288589	0	1
1000483	2	0.6163753	4	1
1000484	1	0.8656652	0	0
1000485	1	0.7010806	2	1
1000486	2	0.9277478	0	0
1000487	1	0.4524742	0	1
1000487	2	0.4254257	0	1
1000488	1	0.6660834	0	1
1000489	2	0.9211168	0	0
1000490	2	0.4353576	3	1
1000491	2	0.8143077	0	1
1000492	2	0.8335876	2	1
1000493	1	0.7694928	0	0
1000493	2	0.5384028	0	1
1000494	2	0.817672	0	0
1000495	1	0.8703418	0	1
1000496	2	0.9471208	0	0
1000497	2	0.2548863	2	1
1000498	1	0.1659664	2	0
1000499	1	0.739302	2	1
1000500	2	0.0378552	2	0
1000501	1	0.6633168	0	1
1000502	2	0.5134289	1	1

Patient ID	Side	Propensity score	Number of tranfusions	Matched
1000503	1	0.5041998	0	1
1000504	1	0.9226371	0	0
1000504	2	0.8634995	0	0
1000505	2	0.9325657	0	0
1000506	2	0.9512951	0	0
1000506	1	0.9512951	0	0
1000507	1	0.5904373	4	1
1000508	2	0.9723294	0	1
1000509	1	0.2845894	6	1
1000510	1	0.9398549	0	0
1000511	1	0.9288847	3	1
1000512	2	0.0504265	0	1
1000513	1	0.9684361	0	0
1000514	1	0.9121083	2	1
1000515	2	0.2457842	5	1
1000516	2	0.7010806	3	1
1000517	2	0.2450073	5	1
1000518	1	0.3400615	2	0
1000519	1	0.9404734	0	0
1000520	1	0.7874315	0	1
1000520	2	0.4964228	0	1
1000521	2	0.6368605	0	1
1000522	2	0.9145093	0	0
1000523	1	0.8583781	0	0
1000524	2	0.5867231	2	1
1000525	2	0.4946738	0	1
1000526	1	0.678968	0	1
1000527	1	0.3162543	4	1
1000528	1	0.6271352	1	1
1000529	1	0.9211168	0	0
1000530	2	0.7083231	0	1
1000531	2	0.2272726	3	0
1000532	1	0.6271352	2	1
1000533	2	0.5122697	3	1
1000534	1	0.755473	0	0
1000535	1	0.9471208	0	0
1000536	2	0.6014652	2	1
1000537	1	0.4131999	4	1
1000538	1	0.9086713	0	0

Patient ID	Side	Propensity score	Number of tranfusions	Matched
1000539	1	0.5591499	2	1
1000539	2	0.5057742	2	1
1000540	1	0.8892304	0	0
1000541	2	0.6637491	2	1
1000542	1	0.218488	0	1
1000543	1	0.1935297	1	1
1000544	2	0.9040454	0	0
1000545	2	0.8268883	0	0
1000546	2	0.7641684	2	1
1000547	1	0.8061834	0	0
1000548	2	0.0157703	3	0
1000549	1	0.7051151	2	1
1000550	2	0.4353576	0	1
1000551	2	0.1680335	0	1
1000552	1	0.8979584	0	0
1000553	1	0.693725	0	1
1000554	1	0.810253	0	0
1000555	1	0.9862315	0	0
1000556	2	0.5041998	0	1
1000557	2	0.559449	0	1
1000558	1	0.3830412	2	1
1000559	1	0.5055801	2	1
1000560	1	0.9481907	0	0
1000561	1	0.5711182	0	1
1000561	2	0.5711182	0	1
1000562	1	0.9684105	0	1
1000563	1	0.4144451	0	1
1000564	2	0.6235195	2	1
1000564	1	0.9039868	1	1
1000565	1	0.9342051	0	0
1000566	2	0.9653663	0	0
1000567	1	0.321832	0	1
1000568	1	0.8665687	0	0
1000569	2	0.5308739	0	1
1000570	2	0.1149155	10	0
1000570	1	0.2216177	2	0
1000571	1	0.8061834	0	0
1000572	2	0.9166842	0	0
1000573	2	0.8420542	1	1

Patient ID	Side	Propensity score	Number of tranfusions	Matched
1000574	1	0.9151533	0	0
1000575	1	0.4144451	0	1
1000576	2	0.7565187	0	0
1000577	1	0.565664	3	1
1000578	1	0.4353576	7	1
1000579	2	0.4820229	0	1
1000580	2	0.3504041	2	0
1000581	2	0.8556663	1	1
1000582	2	0.8652821	0	0
1000582	1	0.8652821	0	0
1000583	1	0.2166086	0	1
1000584	2	0.0527342	4	0
1000585	1	0.8464121	0	0
1000586	1	0.5543528	0	1
1000587	2	0.6103809	1	1
1000588	1	0.5078036	2	1
1000589	2	0.8199402	0	0
1000590	1	0.882295	0	0
1000591	1	0.4873089	8	1
1000592	1	0.5955713	0	1
1000593	2	0.9743555	0	0
1000594	2	0.7511338	0	0
1000595	2	0.9342051	0	0
1000596	1	0.9454951	0	0
1000597	2	0.9119742	0	0
1000598	1	0.1038843	8	0
1000599	2	0.8979311	0	0
1000600	2	0.6361699	0	0
1000601	1	0.9277758	0	0
1000602	2	0.4927672	2	1
1000603	1	0.8968255	0	0
1000604	1	0.9393371	0	0
1000605	1	0.1253911	6	0
1000606	1	0.4349635	2	1
1000607	2	0.895284	2	1
1000608	2	0.7199393	2	1
1000609	1	0.7838778	0	0
1000610	1	0.1335658	6	0
1000611	2	0.8838318	0	0

Patient ID	Side	Propensity score	Number of tranfusions	Matched
1000612	1	0.6788792	0	1
1000613	2	0.5557157	1	1
1000614	2	0.7622421	2	1
1000615	1	0.9431339	0	0
1000616	2	0.9186629	0	0
1000617	1	0.3305405	4	1
1000618	1	0.5092125	2	1
1000619	2	0.9348254	0	0
1000620	2	0.9718675	0	0
1000621	2	0.9070476	0	0
1000622	1	0.9145093	0	0
1000623	1	0.9262035	0	0
1000624	1	0.5836412	1	1
1000625	1	0.8254554	0	1
1000626	1	0.9135338	0	0
1000627	2	0.8622236	1	1
1000628	2	0.2801864	0	1
1000629	1	0.9366489	0	0
1000630	2	0.9380204	0	0
1000631	2	0.7909005	0	0
1000632	1	0.0516379	2	0
1000632	2	0.2550914	0	1
1000633	1	0.7062283	2	1
1000634	1	0.104055	4	0
1000635	1	0.9555191	0	0
1000636	1	0.622981	2	1
1000637	1	0.9724548	0	0
1000638	2	0.4524742	0	1
1000639	1	0.6829168	0	1
1000640	1	0.8148102	0	0
1000640	2	0.8924138	0	0
1000641	1	0.6349469	2	1
1000642	2	0.8380845	0	1
1000643	1	0.6358145	0	1
1000644	1	0.5187632	2	1
1000645	1	0.7602424	4	1
1000646	2	0.3074901	3	0
1000647	1	0.7010806	0	1
1000647	2	0.8926175	0	0

Patient ID	Side	Propensity score	Number of tranfusions	Matched
1000648	2	0.5071085	2	1
1000649	1	0.6807669	0	1
1000650	1	0.3935633	2	1
1000651	1	0.8963693	0	0
1000652	1	0.8380845	0	0
1000653	1	0.9442706	0	0
1000654	2	0.3416895	6	0
1000655	2	0.8303496	2	1
1000655	1	0.8583781	0	0
1000656	1	0.5585162	2	1
1000657	2	0.1936355	1	0
1000658	2	0.914542	0	0
1000659	1	0.6339748	0	1
1000660	1	0.184472	4	0
1000661	2	0.6909467	0	1
1000662	2	0.9796864	0	0
1000663	2	0.8585083	0	0
1000664	1	0.7451044	0	0
1000665	2	0.4353576	0	1
1000666	1	0.8838318	0	1
1000667	1	0.9683244	0	0
1000668	2	0.817672	0	0
1000669	1	0.6093145	2	1
1000670	2	0.3470842	2	0
1000671	1	0.9288847	0	0
1000672	1	0.7059057	0	1
1000673	2	0.2204541	2	0
1000674	1	0.9568595	0	0
1000675	2	0.5716151	0	1
1000676	2	0.3145485	2	0
1000677	2	0.6288589	0	1
1000678	1	0.9256307	0	0
1000679	1	0.9348254	0	0
1000680	1	0.9679403	0	0
1000681	1	0.7854294	2	1
1000681	2	0.7854294	0	0
1000682	2	0.8684553	0	0
1000683	1	0.8624214	0	0
1000684	2	0.8948156	0	0

Patient ID	Side	Propensity score	Number of tranfusions	Matched
1000685	2	0.9827961	0	0
1000686	2	0.7671505	0	0
1000687	1	0.9277478	0	0
1000688	1	0.7638791	0	1
1000689	2	0.9490811	2	1
1000690	2	0.169584	3	0
1000691	1	0.750596	0	0
1000692	1	0.5405086	0	1
1000693	2	0.372895	0	1
1000694	2	0.246825	4	0
1000695	1	0.6210378	0	1
1000696	1	0.8143077	0	1
1000697	1	0.9680528	0	0
1000698	1	0.9395485	0	0
1000699	2	0.1888762	2	0
1000700	2	0.3238886	2	0
1000701	2	0.8248143	0	1
1000702	1	0.8861318	0	1
1000703	2	0.9898134	0	0
1000704	1	0.4419562	0	1
1000705	2	0.5308863	0	1
1000706	1	0.9555191	0	0
1000707	1	0.6805081	2	1
1000708	2	0.2972515	2	1
1000709	1	0.436372	0	1
1000710	1	0.1476607	2	1
1000711	1	0.1474643	2	0
1000712	2	0.9040454	0	0
1000713	2	0.7158331	0	0
1000714	1	0.3250186	3	1
1000715	2	0.2809175	2	0
1000716	2	0.6326291	0	1
1000717	1	0.1267823	2	0
1000718	1	0.9180547	0	0
1000718	2	0.9180547	0	0
1000719	2	0.9273635	0	0
1000720	1	0.769303	0	0
1000721	2	0.096392	5	0
1000722	2	0.2416322	0	1

Patient ID	Side	Propensity score	Number of tranfusions	Matched
1000723	1	0.8131129	0	0
1000724	1	0.3588556	26	1
1000725	1	0.9530739	0	0
1000726	1	0.9278036	0	0
1000727	2	0.9603292	0	0
1000728	1	0.5120277	7	1
1000729	1	0.2458921	2	0
1000730	1	0.8061834	0	0
1000731	2	0.5985009	0	1
1000732	1	0.1289325	8	0
1000733	1	0.8803395	0	0
1000734	2	0.4909714	0	1
1000734	1	0.7837581	0	0
1000735	1	0.0124547	12	0
1000736	1	0.6973977	2	1
1000737	2	0.3382911	0	1
1000738	2	0.7564785	0	0
1000739	2	0.9376553	2	1
1000740	1	0.1306264	0	1
1000741	1	0.5006112	5	1
1000741	2	0.5006112	3	1
1000742	2	0.3179415	2	0
1000743	1	0.2996569	0	1
1000744	1	0.9712601	0	0
1000745	2	0.6637491	1	1
1000746	2	0.8133922	4	1
1000747	2	0.9070476	0	0
1000748	2	0.3185485	2	0
1000748	1	0.4378419	2	1
1000749	2	0.9457731	0	1
1000749	1	0.9557483	0	0
1000750	1	0.8782911	0	1
1000751	1	0.7317822	3	1
1000752	1	0.8906026	0	0
1000753	1	0.8997165	0	0
1000754	2	0.8277611	0	1
1000755	1	0.9024512	0	0
1000756	1	0.9068288	0	0
1000757	1	0.8590341	0	0

Patient ID	Side	Propensity score	Number of tranfusions	Matched
1000758	1	0.7113339	0	0
1000759	2	0.4905409	0	1
1000760	1	0.6538066	0	1
1000760	2	0.6538066	0	0
1000761	2	0.9364763	0	0
1000762	2	0.8747627	0	1
1000763	1	0.7613885	2	1
1000764	1	0.6552167	0	1
1000765	2	0.5532528	1	1
1000766	2	0.5623317	1	1
1000767	1	0.6889592	2	1
1000768	2	0.9611736	0	1
1000769	1	0.8356678	0	0
1000770	2	0.7827674	0	0
1000771	2	0.2449849	2	1
1000772	1	0.9493857	0	0
1000773	2	0.9607372	0	1
1000774	1	0.8381982	0	0
1000775	2	0.8071992	0	0
1000776	2	0.7838778	0	0
1000777	1	0.963065	0	0
1000778	1	0.3752376	2	1
1000779	2	0.7795526	0	0
1000780	2	0.5052486	0	1
1000781	1	0.8615083	0	0
1000782	2	0.2227954	0	1
1000783	2	0.9557483	0	0
1000784	2	0.8159437	0	0
1000785	2	0.8512704	0	0
1000786	2	0.0523019	2	0
1000787	1	0.7613885	0	1
1000788	2	0.9134025	0	0
1000789	1	0.4362131	4	1
1000790	2	0.9243652	0	0
1000791	2	0.5493338	3	1
1000792	1	0.5238844	2	1
1000793	1	0.8541505	0	0
1000794	1	0.8448089	0	0
1000795	1	0.9568595	0	0

Patient ID	Side	Propensity score	Number of tranfusions	Matched
1000796	2	0.8963693	0	0
1000797	2	0.6339748	0	1
1000798	1	0.2024319	3	0
1000799	2	0.3036823	0	1
1000800	1	0.7113339	0	0
1000801	1	0.6339748	0	1
1000802	2	0.5307699	0	1
1000803	1	0.6073469	0	1
1000804	1	0.9256307	0	0
1000805	1	0.6389688	0	0
1000806	2	0.214718	4	0
1000806	1	0.5067756	5	1
1000807	2	0.6210378	2	1
1000808	1	0.6468954	2	1
1000809	1	0.8380845	0	0
1000810	1	0.1717555	2	0
1000811	1	0.7292071	0	0
1000812	2	0.6584128	2	1
1000813	2	0.2604478	2	1
1000814	1	0.9829916	0	0
1000815	1	0.2126324	5	0
1000816	1	0.1582627	4	0
1000817	2	0.782809	0	0
1000818	2	0.7747317	0	0
1000818	1	0.759674	2	1
1000819	2	0.6103809	2	1
1000820	1	0.6363631	2	1
1000821	1	0.7853797	0	1
1000821	2	0.7853797	0	1
1000822	2	0.714073	2	1
1000822	1	0.5948547	0	1
1000823	1	0.8003601	0	1
1000824	2	0.6445818	0	1
1000825	1	0.6846067	0	1
1000826	1	0.4921914	0	1
1000827	1	0.1548002	2	0
1000828	1	0.4353576	2	1
1000829	2	0.9380204	0	0
1000830	2	0.530162	0	1
1000831	2	0.7175789	0	1

Patient ID	Side	Propensity score	Number of tranfusions	Matched
1000832	1	0.8180251	0	1
1000833	2	0.7355201	0	1
1000833	1	0.9078887	0	0
1000834	1	0.3372054	4	0
1000835	1	0.9395485	0	0
1000836	1	0.9366737	0	0
1000837	1	0.0865572	6	0
1000838	1	0.6290658	0	1
1000839	1	0.823929	2	1
1000840	2	0.2828303	2	1
1000841	2	0.914542	0	0
1000841	1	0.7699598	0	1
1000842	2	0.7010806	0	0
1000843	1	0.467087	1	1
1000843	2	0.467087	0	1
1000844	2	0.7845558	0	1
1000844	1	0.905477	0	0
1000845	1	0.5724167	0	0
1000846	2	0.9627395	0	0
1000847	1	0.623601	2	1
1000847	2	0.7089199	0	1
1000848	1	0.9775355	0	0
1000849	1	0.7490443	0	1
1000850	1	0.0854696	4	0
1000851	2	0.4937716	0	1
1000852	1	0.8009974	4	1
1000853	1	0.9366489	0	0
1000854	2	0.8151238	0	0
1000855	2	0.5591499	4	1
1000856	1	0.5290465	0	1
1000857	2	0.9163173	0	0
1000858	1	0.559449	0	1
1000858	2	0.6544533	0	1
1000859	2	0.7783576	2	1
1000860	1	0.9366489	0	0
1000860	2	0.9366489	0	0
1000861	1	0.9718675	0	0
1000862	2	0.7751945	0	0
1000863	2	0.8720679	0	0

Patient ID	Side	Propensity score	Number of tranfusions	Matched
1000864	1	0.1525709	1	0
1000865	2	0.6182663	0	1
1000866	2	0.6579239	0	0
1000867	1	0.8683234	4	1
1000868	2	0.965012	0	0
1000869	2	0.9397372	0	0
1000870	1	0.7373941	0	1
1000871	2	0.8410779	0	0
1000872	2	0.8380845	0	0
1000873	1	0.0976792	4	0
1000874	1	0.774531	0	1
1000875	1	0.957775	0	0
1000876	1	0.7010806	0	1
1000877	2	0.689499	0	0
1000878	1	0.7570091	0	0
1000878	2	0.8242864	0	0
1000879	2	0.9797031	0	0
1000880	1	0.8622236	0	1
1000880	2	0.9568595	0	0
1000881	1	0.9653663	0	0
1000882	2	0.9207713	0	0
1000883	1	0.9827961	0	0
1000884	1	0.5875321	4	1
1000885	1	0.1404493	6	1
1000886	2	0.9471208	0	0
1000887	1	0.8604233	2	1
1000888	2	0.7010806	0	1
1000889	1	0.9043188	3	1
1000890	2	0.872855	0	0
1000891	2	0.6360197	0	1
1000892	1	0.6586223	0	1
1000893	1	0.678968	0	1
1000894	1	0.7672793	0	0
1000895	2	0.9037011	0	0
1000896	2	0.8076437	0	0
1000897	2	0.9898134	0	0
1000898	2	0.9637995	0	0
1000899	1	0.1689041	0	1
1000900	1	0.9197193	0	0
1000901	2	0.882295	0	0

Patient ID	Side	Propensity score	Number of tranfusions	Matched
1000902	1	0.8851865	0	0
1000903	1	0.3138444	3	0
1000904	1	0.8410779	0	0
1000905	1	0.6723223	2	1
1000906	1	0.7483358	0	0
1000907	1	0.6605499	2	1
1000908	2	0.6775226	2	1
1000908	1	0.6775226	4	1
1000909	2	0.2286018	1	1
1000910	1	0.9611736	0	0
1000911	2	0.4100285	2	1
1000912	1	0.064427	2	0
1000913	1	0.026063	2	0
1000914	2	0.6556102	0	1
1000915	2	0.7526721	0	1
1000916	2	0.1267193	5	0
1000917	1	0.8512704	0	0
1000918	1	0.0117575	3	0
1000919	1	0.7783576	2	1
1000920	1	0.9514443	0	0
1000921	1	0.9482519	0	0
1000922	1	0.9397372	0	0
1000923	1	0.8644041	0	0
1000924	1	0.5076226	0	1
1000925	1	0.8512704	0	0
1000926	1	0.1887592	6	0
1000927	1	0.9082494	0	0
1000928	2	0.9811312	0	0
1000929	2	0.1979759	0	1
1000930	1	0.4808866	2	1
1000931	2	0.8644041	0	0
1000932	2	0.905477	0	0
1000933	1	0.4209728	1	1
1000934	1	0.8704304	0	0
1000935	2	0.4701007	0	1
1000936	1	0.9145093	0	0
1000937	1	0.1548002	2	0
1000938	1	0.918583	0	0
1000938	2	0.6873339	0	1
1000939	2	0.8489008	0	0

Patient ID	Side	Propensity score	Number of tranfusions	Matched
1000940	2	0.9684105	0	0
1000941	1	0.8792738	1	1
1000941	2	0.6596535	4	1
1000942	2	0.7783576	0	0
1000943	1	0.1819592	0	1
1000944	1	0.8410779	0	0
1000945	1	0.4796653	0	1
1000946	1	0.2072138	6	0
1000947	2	0.112277	5	1
1000948	1	0.691281	0	0
1000949	2	0.7853797	0	0
1000950	1	0.922837	0	0
1000951	1	0.9712601	0	0
1000952	1	0.9809673	0	0
1000953	2	0.5405086	0	1
1000953	1	0.7877594	0	1
1000954	2	0.2526517	0	1
1000955	1	0.4534992	0	1
1000956	2	0.6427094	0	0
1000957	1	0.1339634	2	0
1000958	1	0.7479218	0	0
1000959	1	0.9119742	0	0
1000960	2	0.963065	0	0
1000961	2	0.096053	3	0
1000962	1	0.8854344	0	0
1000963	1	0.882295	0	1
1000964	1	0.6182663	2	1
1000965	1	0.9684105	0	0
1000966	2	0.7089199	1	1
1000967	1	0.6006756	2	1
1000968	2	0.9557483	0	0
1000969	1	0.9366489	0	0
1000970	2	0.8082396	2	1
1000971	2	0.1114849	2	0
1000972	1	0.9623818	0	0
1000973	2	0.9380204	0	0
1000974	1	0.8148102	0	0
1000975	2	0.8923432	0	0
1000976	1	0.3501185	2	1

Patient ID	Side	Propensity score	Number of tranfusions	Matched
1000977	2	0.2037196	2	0
1000978	1	0.9555191	0	0
1000979	2	0.9781832	0	0
1000980	2	0.4206422	0	1
1000981	2	0.8869002	0	0
1000982	1	0.9145093	0	0
1000983	1	0.7064988	0	1
1000984	1	0.9297823	0	0
1000985	2	0.9460049	0	0
1000986	1	0.2467381	2	1
1000987	1	0.9483179	0	0
1000987	2	0.9453826	0	0
1000988	1	0.5307699	0	1
1000989	1	0.6517544	0	0
1000989	2	0.6517544	0	0
1000990	1	0.864351	0	0
1000991	2	0.3798259	3	1
1000992	2	0.1588929	6	0
1000992	1	0.1867977	3	0
1000993	2	0.7055906	0	1
1000994	1	0.6298494	0	1
1000995	1	0.9468601	0	0
1000996	1	0.6576642	0	1
1000996	2	0.8652821	0	0
1000997	1	0.8987589	0	0
1000998	1	0.908652	0	0
1000998	2	0.9267618	0	0
1000999	1	0.6210378	0	0
1001000	1	0.8967518	0	0
1001001	1	0.5967073	0	1
1001002	1	0.6544533	0	1
1001003	1	0.7010806	0	0
1001003	2	0.7010806	2	1
1001004	2	0.8002475	0	0
1001005	1	0.626568	0	1
1001006	2	0.806943	2	1
1001007	1	0.7211949	4	1
1001008	2	0.4452557	2	1
1001009	2	0.8387457	0	0

Patient ID	Side	Propensity score	Number of tranfusions	Matched
1001010	1	0.1020288	0	1
1001011	2	0.9718675	0	0
1001012	1	0.5745877	1	1
1001013	2	0.8624214	0	0
1001014	1	0.1362193	0	1
1001015	2	0.9236301	2	1
1001015	1	0.9236301	1	1
1001016	2	0.8242614	0	0
1001017	1	0.7526756	4	1
1001018	2	0.9512951	0	0
1001019	1	0.9398549	0	0
1001020	2	0.2797864	2	1
1001021	1	0.8531178	1	1
1001022	1	0.5056091	8	1
1001022	2	0.402726	5	1
1001023	2	0.9366737	0	0
1001024	1	0.9069528	0	0
1001025	2	0.3751552	0	1
1001026	1	0.5122697	2	1
1001027	1	0.5307699	0	1
1001028	2	0.3525382	1	0
1001029	2	0.3711177	5	1
1001030	1	0.8199402	0	1
1001031	1	0.8363182	0	0
1001032	2	0.9684105	2	1
1001033	1	0.8261372	0	1
1001034	1	0.9151533	0	0
1001035	2	0.9743555	0	0
1001036	2	0.7010806	0	0
1001037	1	0.1581925	5	0
1001038	2	0.9648087	0	1
1001039	1	0.9653663	0	0
1001040	1	0.8160688	2	1
1001041	1	0.4739665	0	1
1001042	2	0.0532364	0	1
1001043	2	0.2550914	3	1
1001044	2	0.9568595	0	0
1001045	1	0.7878527	0	1
1001045	2	0.9119415	0	0

Patient ID	Side	Propensity score	Number of tranfusions	Matched
1001046	2	0.8084822	0	0
1001047	2	0.9419123	0	0
1001048	2	0.9380204	1	1
1001049	2	0.9395485	0	0
1001050	2	0.921033	2	1
1001050	1	0.7025956	2	1
1001051	2	0.8356678	0	0
1001052	2	0.9653663	0	0
1001053	2	0.6656533	0	1
1001054	2	0.7845374	0	1
1001055	1	0.3302219	0	1
1001056	2	0.9568595	0	0
1001057	2	0.8986861	0	0
1001058	1	0.7211949	0	0
1001059	1	0.9623802	0	0
1001060	1	0.810253	0	0
1001061	2	0.7968503	3	1
1001062	1	0.9527564	0	0
1001063	1	0.8464121	0	0
1001064	1	0.9180547	0	0
1001065	2	0.6179632	0	1
1001066	1	0.9119742	0	1
1001067	1	0.3821907	1	0
1001068	1	0.8603249	0	0
1001069	2	0.7643871	0	0
1001070	1	0.7175789	2	1
1001070	2	0.7175789	0	0
1001071	1	0.957775	0	0
1001072	1	0.974966	0	0
1001073	1	0.8684553	0	0
1001074	2	0.9145093	0	0
1001075	2	0.0121565	8	0
1001076	1	0.9569282	0	0
1001077	1	0.4900505	0	1
1001078	1	0.2062671	2	1
1001079	1	0.8063357	0	0
1001080	2	0.7797947	2	1
1001081	1	0.9000514	0	0
1001082	1	0.4971275	0	1

Patient ID	Side	Propensity score	Number of tranfusions	Matched
1001082	2	0.4971275	0	1
1001083	1	0.8831506	0	0
1001084	2	0.8721251	0	0
1001084	1	0.85503	0	0
1001085	1	0.4044706	2	1
1001086	2	0.8970562	0	0
1001087	1	0.8267231	0	0
1001088	1	0.56959	0	1
1001089	1	0.5949637	2	1
1001090	1	0.6292635	2	1
1001091	1	0.9372068	0	0
1001092	1	0.5482672	2	1
1001093	1	0.9862315	0	0
1001094	2	0.2896238	1	0
1001095	2	0.0791264	8	0
1001096	2	0.3588556	0	1
1001097	2	0.8763473	0	1
1001097	1	0.7903502	0	0
1001098	1	0.8531178	0	0
1001099	1	0.8247183	0	0
1001100	1	0.0433764	4	0
1001101	2	0.8380845	0	0
1001102	1	0.2620691	23	0
1001102	2	0.4010291	2	1
1001103	1	0.9684105	0	0
1001104	2	0.3466773	2	1
1001105	1	0.4065954	2	1
1001106	2	0.6915566	0	0
1001107	1	0.9743555	0	0
1001108	2	0.9486418	0	0
1001109	2	0.6973171	2	1
1001110	1	0.9565483	0	0
1001111	1	0.8956693	0	0
1001112	2	0.0156117	12	0
1001113	1	0.8622236	0	0
1001113	2	0.9568595	0	1
1001114	2	0.5005724	5	1
1001115	1	0.8143077	0	1
1001116	1	0.7010806	0	0

Patient ID	Side	Propensity score	Number of tranfusions	Matched
1001117	2	0.8772783	0	0
1001118	1	0.396171	0	1
1001119	1	0.9406696	2	1
1001120	2	0.6624998	0	0
1001121	2	0.9119742	0	0
1001122	1	0.8298282	0	0
1001123	1	0.9121083	0	1
1001124	1	0.9163173	0	0
1001124	2	0.622981	0	1
1001125	1	0.8196841	1	1
1001126	2	0.8583781	0	0
1001127	2	0.8611213	0	0
1001128	1	0.0272167	14	0
1001129	2	0.8075833	0	1
1001130	2	0.9274384	0	0
1001131	2	0.8462635	2	1
1001131	1	0.9383534	0	0
1001132	2	0.8443209	0	0
1001133	1	0.71377	0	1
1001134	2	0.8143077	0	0
1001135	2	0.9068288	0	0
1001136	2	0.5826166	1	1
1001137	1	0.6062345	0	1
1001138	2	0.8287051	0	1
1001139	1	0.321832	3	0
1001140	2	0.9471208	0	0
1001141	2	0.9398549	0	0
1001142	2	0.2637527	1	0
1001143	1	0.6775226	0	1
1001144	1	0.8424176	0	0
1001144	2	0.5764288	0	1
1001145	2	0.8611213	0	1
1001146	1	0.8298011	0	0
1001147	2	0.9386415	0	0
1001148	1	0.1886882	4	0
1001149	2	0.6210378	0	1
1001150	2	0.56959	2	1
1001151	1	0.817672	0	0
1001152	1	0.9384815	2	1
1001153	1	0.9481907	0	1

Patient ID	Side	Propensity score	Number of tranfusions	Matched
1001153	2	0.9481907	0	0
1001154	2	0.8838318	0	0
1001155	1	0.7864738	0	0
1001156	1	0.796731	0	0
1001156	2	0.6265182	0	1
1001157	2	0.8698594	2	1
1001158	1	0.7059077	1	1
1001159	2	0.1863027	2	0
1001160	1	0.8082807	2	1
1001161	1	0.8464121	0	0
1001162	2	0.4050491	0	1
1001163	1	0.9387129	1	1
1001164	1	0.3176977	0	1
1001165	1	0.7115915	2	1
1001166	2	0.8590341	0	0
1001167	2	0.2179855	3	1
1001168	2	0.0563326	6	0
1001169	1	0.7610734	0	0
1001170	1	0.7113339	0	0
1001171	1	0.4733407	1	1
1001172	1	0.7861992	0	1
1001173	1	0.8268883	0	0
1001174	2	0.8381982	0	0
1001175	2	0.8512704	0	0
1001176	2	0.2024319	2	1
1001176	1	0.2024319	5	0
1001177	2	0.4861681	0	1
1001178	1	0.6867087	1	1
1001179	2	0.4774967	2	1
1001180	2	0.5092125	0	1
1001180	1	0.5623317	0	1
1001181	2	0.9380204	0	0
1001182	1	0.1715304	4	1
1001183	2	0.096199	2	0
1001184	1	0.3588556	7	1
1001185	1	0.5955561	0	1
1001186	1	0.7838286	0	0
1001187	2	0.412128	2	1
1001188	1	0.3068618	1	1

Patient ID	Side	Propensity score	Number of tranfusions	Matched
1001189	1	0.619632	1	1
1001190	1	0.8464121	0	1
1001190	2	0.8464121	0	0
1001191	1	0.9557483	0	1
1001192	1	0.8531178	0	0
1001193	1	0.9256307	0	0
1001194	1	0.4998259	0	1
1001195	1	0.1564605	2	0
1001196	2	0.5025039	2	1
1001197	1	0.6763113	1	1
1001198	1	0.2286018	2	1
1001199	2	0.0953166	4	0
1001200	1	0.2444667	2	0
1001201	2	0.7717639	1	1
1001202	2	0.9555191	0	0
1001203	2	0.6465287	0	1
1001204	2	0.3575094	4	1
1001205	2	0.7317822	0	1
1001206	2	0.6538066	0	1
1001207	1	0.7175789	0	0
1001207	2	0.6210378	0	0
1001208	1	0.0814313	0	1
1001209	1	0.4534675	2	1
1001210	1	0.87418	0	0
1001211	2	0.6901166	0	0
1001212	1	0.9128584	0	0
1001213	2	0.8963693	0	0
1001213	1	0.9366489	0	0
1001214	1	0.4415582	0	1
1001215	1	0.7175789	0	0
1001216	1	0.3683397	0	1
1001217	2	0.1159226	7	0
1001218	1	0.9097797	0	0
1001219	2	0.693725	0	0
1001220	1	0.8323885	0	0
1001221	1	0.9366489	0	1
1001222	1	0.56959	3	1
1001223	1	0.7062283	0	1
1001224	1	0.8143077	0	0

Patient ID	Side	Propensity score	Number of tranfusions	Matched
1001225	1	0.6210378	0	0
1001226	1	0.3236863	2	0
1001227	1	0.8033172	0	0
1001227	2	0.8349239	0	1
1001228	2	0.9557483	0	0
1001229	1	0.9603292	0	0
1001230	1	0.1510397	4	1
1001231	1	0.2082911	2	0
1001232	2	0.957775	0	0
1001233	1	0.9364664	0	0
1001234	2	0.810253	0	0
1001235	1	0.9557483	0	0
1001236	1	0.6362959	0	1
1001237	1	0.467087	1	1
1001238	1	0.2486238	4	0
1001239	1	0.230755	2	1
1001239	2	0.3833727	4	1
1001240	1	0.5307699	2	1
1001241	1	0.7512119	0	0
1001242	1	0.7609577	0	0
1001243	1	0.6103809	0	1
1001244	1	0.3539359	2	0
1001245	2	0.0892451	9	0
1001246	1	0.9119742	0	0
1001247	1	0.6461803	2	1
1001248	2	0.3546558	0	1
1001249	1	0.4817285	2	1
1001250	1	0.9521768	0	0
1001250	2	0.9684105	0	0
1001251	2	0.5067756	2	1
1001251	1	0.7699598	2	1
1001252	2	0.600304	0	1
1001253	1	0.6593094	0	0
1001254	1	0.4077469	0	1
1001255	1	0.8588787	0	0
1001256	1	0.3375722	2	1
1001257	1	0.9128584	0	0
1001258	1	0.3212566	0	1
1001259	1	0.2236856	0	1

Patient ID	Side	Propensity score	Number of tranfusions	Matched
1001260	2	0.5122697	2	1
1001261	2	0.9796864	0	1
1001262	2	0.7511338	0	0
1001263	1	0.4008986	0	1
1001264	1	0.3926625	3	0
1001265	1	0.9063035	0	0
1001266	2	0.9419123	0	0
1001267	1	0.573879	0	1
1001268	1	0.810253	0	0
1001269	1	0.9607372	0	0
1001270	1	0.156684	2	0
1001271	1	0.9395485	0	0
1001272	2	0.4802569	2	1
1001273	1	0.9573394	0	0
1001274	1	0.6631602	2	1
1001275	2	0.8532444	0	0
1001276	2	0.7451044	0	1
1001277	1	0.6443724	0	0
1001278	2	0.8380845	0	0
1001279	1	0.8033238	0	0
1001280	2	0.9550222	0	0
1001281	2	0.5542496	2	1
1001282	1	0.4415582	2	1
1001283	1	0.2862755	2	0
1001284	2	0.9119415	0	0
1001285	1	0.4044706	0	1
1001286	1	0.9735961	0	0
1001287	2	0.8700188	0	0
1001287	1	0.9398549	0	0
1001288	2	0.3264199	2	0
1001289	1	0.714073	0	1
1001290	1	0.8375368	0	1
1001291	1	0.7162491	0	0
1001292	1	0.3779718	3	0
1001293	1	0.7317822	0	1
1001294	2	0.6964176	0	1
1001295	1	0.6696718	2	1
1001296	2	0.2710625	6	1
1001297	2	0.5621231	3	1

Patient ID	Side	Propensity score	Number of tranfusions	Matched
1001297	1	0.8159437	0	0
1001298	1	0.8589752	0	0
1001299	1	0.623785	2	1
1001300	1	0.9827961	0	0
1001301	1	0.8004269	0	0
1001302	2	0.8381982	0	0
1001303	1	0.2693425	0	1
1001304	1	0.8143077	0	0
1001305	2	0.6872183	0	1
1001306	2	0.1780778	8	0
1001307	1	0.5065332	1	1
1001308	2	0.7834761	0	0
1001309	1	0.716825	0	0
1001310	1	0.9145093	0	0
1001311	1	0.9043188	0	0
1001312	1	0.9380204	0	0
1001313	2	0.7338336	0	1
1001313	1	0.7451044	0	0
1001314	2	0.9555191	0	0
1001315	2	0.3504041	2	0
1001316	1	0.9684105	0	0
1001317	2	0.9366489	0	0
1001318	2	0.4435362	0	1
1001319	1	0.2523237	3	0
1001320	2	0.9195277	2	1
1001321	1	0.9680528	0	0
1001322	1	0.7698659	1	1
1001323	2	0.1666741	6	1
1001323	1	0.4317516	2	1
1001324	1	0.5482672	0	1
1001325	2	0.5960043	5	1
1001326	1	0.6790591	0	1
1001327	2	0.882295	0	0
1001328	1	0.755473	0	0
1001329	1	0.4745131	0	1
1001330	2	0.1575875	0	1
1001331	1	0.8531178	0	0
1001332	1	0.9039453	0	0
1001333	1	0.4274528	4	0

Patient ID	Side	Propensity score	Number of tranfusions	Matched
1001333	2	0.2772692	17	0
1001334	2	0.9348254	0	0
1001335	2	0.9457731	0	0
1001336	2	0.6271352	0	1
1001337	2	0.9070476	0	0
1001338	1	0.8866693	2	1
1001339	2	0.8778271	0	0
1001340	1	0.8987589	0	0
1001341	1	0.4634502	2	1
1001342	2	0.9555191	0	0
1001343	2	0.5195146	4	1
1001344	2	0.0490667	17	0
1001345	2	0.8268883	0	0
1001346	1	0.9395485	0	0
1001346	2	0.8143077	0	0
1001347	1	0.0086243	3	0
1001348	1	0.7204169	3	1
1001349	2	0.7484937	0	0
1001350	2	0.6273714	0	1
1001351	2	0.7299379	0	0
1001352	1	0.5446812	0	1
1001353	2	0.8199402	0	0
1001354	1	0.1570563	2	0
1001355	1	0.140816	3	0
1001356	1	0.2707317	0	1
1001357	2	0.9197811	4	1
1001358	1	0.2314453	3	0
1001359	1	0.2416322	3	1
1001360	1	0.473773	1	1
1001361	2	0.8719391	2	1
1001362	1	0.6368605	1	1
1001363	1	0.475834	1	1
1001364	2	0.7054055	2	1
1001365	2	0.9398549	0	0
1001366	2	0.2204541	1	0
1001366	1	0.3212566	2	1
1001367	1	0.511364	6	1
1001368	2	0.4006762	0	1
1001369	1	0.7010806	0	1

Patient ID	Side	Propensity score	Number of tranfusions	Matched
1001370	2	0.6449332	0	1
1001371	1	0.5395649	0	1
1001372	1	0.9000514	0	0
1001373	2	0.7699598	0	0
1001374	2	0.7490443	2	1
1001375	1	0.9195277	0	1
1001376	2	0.5199525	0	1
1001377	1	0.3911606	1	0
1001378	2	0.9342051	0	1
1001379	2	0.1455373	2	1
1001380	1	0.3617395	2	1
1001381	2	0.9366489	0	0
1001382	1	0.3887759	13	1
1001382	2	0.3887759	11	0
1001383	1	0.1858658	6	0
1001384	1	0.7783576	0	1
1001385	1	0.8335385	0	1
1001386	1	0.5308739	4	1
1001387	2	0.0984957	4	0
1001388	1	0.9639877	0	0
1001389	1	0.4203475	3	1
1001390	2	0.9555191	0	0
1001391	2	0.2353372	2	0
1001391	1	0.2353372	2	1
1001392	1	0.8420542	0	1
1001393	1	0.8748827	0	0
1001394	1	0.246825	1	1
1001395	1	0.2836684	2	1
1001396	1	0.2236856	4	1
1001397	1	0.473773	1	1
1001398	2	0.5737743	4	1
1001399	2	0.6206773	0	1
1001400	2	0.1929442	4	0
1001401	2	0.5687177	0	1
1001402	1	0.4320913	4	1
1001403	2	0.9454951	0	0
1001403	1	0.9454951	0	0
1001404	1	0.3305501	0	1
1001405	2	0.9557483	0	0

Patient ID	Side	Propensity score	Number of tranfusions	Matched
1001406	1	0.1888976	2	0
1001407	2	0.8938577	0	0
1001408	2	0.9395485	0	0
1001409	2	0.4745131	0	1
1001410	1	0.9000514	0	0
1001411	2	0.0295375	2	0
1001412	1	0.6797838	0	0
1001412	2	0.6797838	0	1
1001413	2	0.6210378	2	1
1001414	1	0.6468954	2	1
1001415	2	0.9568595	0	0
1001416	1	0.4824428	0	1
1001417	1	0.1553156	6	0
1001418	2	0.9040454	0	0
1001419	2	0.7559605	0	1
1001420	1	0.8549261	0	1
1001421	2	0.9380204	0	0
1001422	2	0.9380204	0	0
1001423	1	0.8910959	0	0
1001424	2	0.7346398	0	0
1001425	1	0.7022124	0	0
1001426	1	0.1238085	2	0
1001427	1	0.8578785	0	0
1001428	2	0.8615083	0	0
1001428	1	0.9555191	0	0
1001429	1	0.7441412	0	0
1001430	1	0.1304628	1	0
1001431	1	0.6124737	0	1
1001432	1	0.918583	0	0
1001433	1	0.5585162	0	1
1001434	2	0.559449	0	1
1001435	2	0.1583484	7	0
1001436	1	0.0833389	4	0
1001437	2	0.810253	0	0
1001438	1	0.4349635	2	1
1001439	1	0.9366489	0	0
1001440	2	0.2182333	3	0
1001441	2	0.6586223	0	0
1001442	2	0.9266772	0	0

Patient ID	Side	Propensity score	Number of tranfusions	Matched
1001443	1	0.7375254	4	1
1001444	1	0.6909467	0	1
1001445	1	0.9568595	0	0
1001446	2	0.8622236	0	0
1001447	1	0.4783902	0	1
1001448	2	0.7907302	0	0
1001449	1	0.838091	0	0
1001450	2	0.9512951	0	0
1001451	1	0.7451837	0	0
1001452	1	0.0963621	6	0
1001453	1	0.8765468	0	0
1001454	1	0.2250382	0	1
1001455	1	0.7733651	7	1
1001455	2	0.6673521	2	1
1001456	1	0.965269	0	0
1001457	1	0.6461811	2	1
1001458	2	0.554448	7	1
1001459	1	0.0272167	3	0
1001460	1	0.6235333	0	1
1001461	1	0.263743	0	1
1001461	2	0.378616	0	1
1001462	1	0.9623802	0	0
1001463	2	0.2117173	0	1
1001464	1	0.678968	0	0
1001465	1	0.6504241	2	1
1001466	2	0.9197811	4	1
1001467	1	0.9557483	0	0
1001468	1	0.4545422	4	1
1001469	1	0.8066371	0	1
1001470	1	0.8700188	3	1
1001471	1	0.7451044	4	1
1001472	1	0.8418481	0	0
1001473	2	0.551797	0	1
1001474	2	0.4775784	0	1
1001475	1	0.4139172	3	1
1001476	1	0.5050592	1	1
1001477	1	0.7817874	0	1
1001478	2	0.5912416	0	1
1001479	1	0.8242614	0	0
1001480	2	0.9653663	0	0

Patient ID	Side	Propensity score	Number of tranfusions	Matched
1001481	1	0.9512951	0	0
1001481	2	0.8464121	0	0
1001482	2	0.7010806	1	1
1001483	2	0.6124737	0	1
1001484	1	0.1205094	1	0
1001485	1	0.945728	0	0
1001485	2	0.9668489	0	0
1001486	2	0.9502168	0	0
1001487	1	0.7222876	2	1
1001488	2	0.5687177	0	1
1001489	1	0.6807669	2	1
1001490	1	0.9119742	0	1
1001491	1	0.9623802	0	0
1001492	2	0.3741679	3	1
1001493	2	0.9714107	0	0
1001494	1	0.7994878	0	0
1001495	1	0.9000485	0	0
1001496	2	0.2595311	0	1
1001497	1	0.0095464	7	0
1001498	1	0.8967878	0	0
1001498	2	0.9149639	0	0
1001499	1	0.9197193	0	0
1001500	2	0.1680335	1	1
1001501	2	0.8247183	0	1
1001502	1	0.9262035	0	1
1001503	2	0.4221659	2	1
1001504	1	0.4209728	4	1
1001505	2	0.8252838	0	0
1001506	1	0.4950411	2	1
1001506	2	0.7869124	2	1
1001507	2	0.1969915	2	1
1001508	2	0.748557	0	1
1001509	1	0.8762677	0	1
1001510	1	0.4868262	2	1
1001511	2	0.7701729	1	1
1001512	1	0.1473102	1	0
1001513	2	0.7638791	0	0
1001514	1	0.5041998	0	1
1001515	1	0.7838778	0	1

Patient ID	Side	Propensity score	Number of tranfusions	Matched
1001516	1	0.5482672	2	1
1001517	1	0.7672793	2	1
1001518	1	0.6363631	0	0
1001519	1	0.9481907	0	0
1001520	2	0.8801035	0	0
1001521	1	0.0981781	5	0
1001522	1	0.9482519	0	0
1001522	2	0.9482519	0	0
1001523	1	0.8923432	0	0
1001524	2	0.2992102	2	1
1001524	1	0.2992102	2	1
1001525	1	0.897856	0	0
1001526	2	0.765812	0	0
1001527	2	0.9557483	0	0
1001528	1	0.3104382	2	1
1001529	1	0.755473	0	0
1001530	1	0.9034399	0	0
1001531	1	0.4967465	0	1
1001532	2	0.7537837	0	0
1001533	1	0.4327061	2	0
1001534	1	0.6579239	0	0
1001535	1	0.6368605	0	1
1001536	1	0.642379	0	0
1001537	2	0.8622236	0	0
1001538	2	0.2082397	2	0
1001539	1	0.9653663	0	0
1001540	1	0.622981	0	1
1001541	1	0.8038026	0	0
1001542	2	0.9684105	0	0
1001543	1	0.6349469	0	0
1001544	2	0.678968	0	0
1001545	1	0.7062283	1	1
1001546	1	0.7836873	0	0
1001547	1	0.3133725	3	0
1001548	2	0.2024319	2	0
1001549	2	0.5587219	0	1
1001550	2	0.9530739	0	0
1001551	2	0.9493857	0	0
1001552	2	0.599822	0	1

Patient ID	Side	Propensity score	Number of tranfusions	Matched
1001552	1	0.599822	1	1
1001553	2	0.8006195	0	0
1001554	1	0.6179632	0	1
1001555	1	0.2525808	0	1
1001556	2	0.0476047	4	0
1001557	1	0.7910648	3	1
1001558	2	0.1712995	1	0
1001559	1	0.7292201	0	0
1001560	1	0.8652821	0	0
1001561	2	0.5935675	0	1
1001562	2	0.8386947	0	0
1001563	1	0.7717639	3	1
1001564	1	0.8464121	2	1
1001565	1	0.7199393	2	1
1001566	1	0.7628868	0	1
1001567	2	0.9767195	0	0
1001568	1	0.8933589	0	0
1001569	2	0.8583781	0	0
1001570	1	0.9395485	0	0
1001571	2	0.9653663	0	0
1001572	2	0.8633056	0	0
1001573	1	0.9323972	0	0
1001574	1	0.6909467	0	1
1001575	1	0.3811998	2	1
1001576	1	0.3575094	2	1
1001577	2	0.1268756	4	0
1001578	2	0.3106769	5	1
1001578	1	0.0933591	0	1
1001579	2	0.8457341	0	0
1001580	2	0.925054	0	0
1001581	2	0.6522921	0	0
1001582	1	0.242314	4	0
1001583	1	0.9713844	0	0
1001583	2	0.9763465	0	0
1001584	2	0.4493395	0	1
1001585	2	0.3125997	5	1
1001586	1	0.5900283	0	1
1001587	2	0.8390119	2	1
1001588	1	0.9366489	0	0

Patient ID	Side	Propensity score	Number of tranfusions	Matched
1001589	2	0.9743555	0	0
1001590	1	0.9166842	0	0
1001591	1	0.3127286	0	1
1001592	2	0.679337	0	0
1001593	1	0.0542758	2	0
1001594	1	0.8381982	0	0
1001595	2	0.4529615	1	1
1001596	1	0.2841837	0	1
1001597	1	0.5575969	4	1
1001598	1	0.8462212	0	0
1001599	1	0.8622236	0	0
1001600	2	0.3458062	4	1
1001601	1	0.9166842	0	0
1001602	1	0.5002404	0	1
1001603	2	0.6179632	0	1
1001604	2	0.636353	0	0
1001605	1	0.3961032	3	1
1001606	1	0.3243744	0	1
1001607	2	0.4882489	2	1
1001608	2	0.5427269	5	1
1001608	1	0.3989192	5	1
1001609	2	0.1457462	4	0
1001610	2	0.321832	2	1
1001610	1	0.467087	2	1
1001611	1	0.6618387	0	1
1001612	2	0.3031601	0	1
1001612	1	0.755473	0	0
1001613	2	0.8342077	0	0
1001614	2	0.9372068	0	0
1001615	1	0.6516405	2	1
1001616	1	0.4625525	8	1
1001617	1	0.2865402	2	0
1001618	2	0.0147431	5	0
1001619	2	0.9482519	0	0
1001620	1	0.9050807	0	0
1001621	2	0.9557838	0	0
1001622	1	0.9163173	0	0
1001623	1	0.559449	0	1
1001624	1	0.8061834	0	0

Patient ID	Side	Propensity score	Number of tranfusions	Matched
1001625	2	0.8604233	0	1
1001626	2	0.9570311	0	0
1001627	1	0.1438045	3	0
1001628	1	0.8410208	0	0
1001629	2	0.9163947	0	0
1001630	1	0.9247656	0	0
1001631	1	0.8700188	0	1
1001632	2	0.7743031	0	0
1001633	1	0.5747471	0	1
1001634	1	0.8137449	0	0
1001635	1	0.2337935	0	1
1001636	2	0.7717639	0	0
1001637	1	0.7602424	0	1
1001638	2	0.2260023	13	0
1001639	2	0.5307699	1	1
1001640	1	0.1110761	2	0
1001641	1	0.8317252	0	0
1001642	2	0.7211463	0	1
1001643	1	0.9493857	0	0
1001644	1	0.6565705	0	1
1001645	2	0.7999699	1	1
1001646	2	0.4556627	1	1
1001646	1	0.7171406	0	0
1001647	1	0.0995094	6	0
1001648	1	0.6019842	0	1
1001649	2	0.6462418	0	1
1001650	2	0.7994878	0	0
1001651	2	0.5460748	2	1
1001652	1	0.7471596	3	1
1001653	1	0.6576642	0	0
1001654	1	0.0156407	3	0
1001655	1	0.2996909	0	1
1001656	2	0.3868049	1	1
1001657	1	0.9825048	0	0
1001658	1	0.9398549	0	0
1001659	1	0.467087	1	1
1001660	1	0.242314	2	1
1001661	1	0.0986374	2	0
1001662	1	0.8468805	3	1

Patient ID	Side	Propensity score	Number of tranfusions	Matched
1001663	1	0.9482519	0	0
1001664	2	0.5041998	0	1
1001665	1	0.914542	0	0
1001666	1	0.918583	0	0
1001667	1	0.9569966	0	0
1001668	1	0.9557483	0	0
1001669	1	0.9139531	0	0
1001670	1	0.7128229	2	1
1001671	1	0.2067997	3	0
1001671	2	0.473773	2	1
1001672	2	0.9568595	0	0
1001673	1	0.9512951	0	0
1001674	2	0.7858006	1	1
1001675	2	0.8143077	0	0
1001676	1	0.4115368	0	1
1001677	2	0.7317822	1	1
1001678	2	0.4320913	0	1
1001679	2	0.9653663	0	0
1001680	1	0.7600489	0	0
1001681	1	0.1903923	0	1
1001682	2	0.7906248	0	1
1001682	1	0.7862898	0	0
1001683	1	0.8729871	0	0
1001684	1	0.9557483	0	0
1001685	2	0.1625248	6	0
1001686	2	0.4660686	4	1
1001687	1	0.796731	0	1
1001688	1	0.4415582	7	1
1001689	1	0.4151808	0	1
1001690	1	0.7317822	2	1
1001691	1	0.9712601	0	0
1001692	2	0.3250186	2	1
1001693	2	0.9557483	0	0
1001694	1	0.9364788	0	0
1001695	1	0.9366737	0	0
1001696	2	0.6506863	0	1
1001696	1	0.810253	0	0
1001697	2	0.5308739	0	1
1001698	1	0.9256307	0	0

Patient ID	Side	Propensity score	Number of tranfusions	Matched
1001699	2	0.622981	2	1
1001700	2	0.7089199	0	1
1001701	1	0.6090651	2	1
1001702	1	0.832713	0	0
1001703	2	0.8317252	0	0
1001704	2	0.9150608	0	0
1001705	2	0.9166842	0	0
1001706	1	0.0713808	16	0
1001707	1	0.3588556	0	1
1001708	1	0.65763	0	0
1001709	2	0.9174228	0	0
1001710	2	0.9366489	0	0
1001711	2	0.8047536	0	1
1001712	2	0.9070476	0	0
1001713	1	0.7656533	0	0
1001714	1	0.4012625	0	1
1001714	2	0.7462821	0	0
1001715	2	0.9718675	2	1
1001716	2	0.7353651	0	1
1001717	1	0.6273714	0	1
1001717	2	0.898414	0	0
1001718	2	0.9653663	0	0
1001719	1	0.757626	0	1
1001720	2	0.9559048	0	0
1001721	1	0.4524742	0	1
1001721	2	0.810253	0	0
1001722	2	0.473773	0	1
1001723	1	0.6605499	0	1
1001724	2	0.642379	0	0
1001725	1	0.2742822	0	1
1001726	1	0.3057131	6	0
1001727	2	0.9150608	0	0
1001728	1	0.410127	0	1
1001729	1	0.7113339	0	0
1001730	2	0.9222556	0	0
1001731	2	0.6805081	0	1
1001732	2	0.8825185	0	0
1001733	2	0.0948138	4	0
1001734	1	0.5575969	7	1
1001734	2	0.7751945	0	0

Patient ID	Side	Propensity score	Number of tranfusions	Matched
1001735	2	0.7484937	0	0
1001736	1	0.3539359	4	1
1001737	2	0.5904373	2	1
1001738	1	0.7471596	0	0
1001739	1	0.9095091	0	0
1001739	2	0.8684553	0	0
1001740	2	0.4811878	2	1
1001741	2	0.9568595	0	0
1001742	1	0.8380357	5	1
1001743	2	0.7139026	1	1
1001743	1	0.6435435	0	1
1001744	1	0.2669081	0	1
1001745	1	0.8684553	0	0
1001746	2	0.8965559	0	0
1001747	2	0.5985411	0	1
1001747	1	0.6926273	0	0
1001748	2	0.9154188	0	0
1001748	1	0.9532798	0	0
1001749	1	0.8458476	0	0
1001750	2	0.4270264	0	1
1001750	1	0.5394653	0	1
1001751	1	0.7730314	0	1
1001752	1	0.7836873	0	0
1001753	2	0.8159437	0	0
1001754	1	0.9482519	0	0
1001755	1	0.4440603	0	1
1001756	2	0.2487272	2	0
1001757	2	0.475834	2	1
1001758	1	0.9256307	0	0
1001759	2	0.7010806	2	1
1001760	2	0.9684105	0	0
1001761	1	0.2898611	0	1
1001762	1	0.7175789	0	1
1001763	1	0.0441022	2	0
1001764	2	0.914542	0	0
1001765	1	0.5740564	0	1
1001766	1	0.5740564	0	0
1001767	1	0.3067493	0	1
1001768	1	0.6660834	2	1

Patient ID	Side	Propensity score	Number of tranfusions	Matched
1001769	2	0.7761444	0	0
1001770	2	0.3282734	3	1
1001771	2	0.8604372	0	0
1001772	2	0.8590341	2	1
1001773	2	0.9398549	0	0
1001773	1	0.9398549	0	0
1001774	2	0.2550914	6	1
1001775	1	0.833655	0	0
1001776	1	0.9512951	0	0
1001777	1	0.6565705	0	1
1001777	2	0.9095091	0	0
1001778	1	0.9203308	0	0
1001779	2	0.6210378	2	1
1001780	1	0.8720679	0	1
1001780	2	0.9278036	0	0
1001781	1	0.9269953	0	0
1001782	1	0.2479462	2	0
1001783	1	0.9555191	0	0
1001784	2	0.1640049	2	0
1001785	1	0.4567948	2	1
1001786	2	0.8335876	2	1
1001787	1	0.8830779	0	0
1001788	1	0.8583781	0	0
1001789	1	0.3321937	0	1
1001790	1	0.3994056	2	0
1001791	2	0.7004812	2	1
1001792	1	0.8923432	0	0
1001793	1	0.7759103	2	1
1001794	1	0.5161757	0	1
1001795	2	0.7451044	0	0
1001796	1	0.7250152	0	1
1001797	2	0.8386175	0	0
1001798	2	0.5116775	0	1
1001799	1	0.7245996	0	0
1001800	2	0.9639877	0	0
1001801	1	0.473773	2	1
1001802	2	0.2286018	0	1
1001803	2	0.9366737	0	0
1001804	1	0.0676972	6	0
1001805	1	0.5091986	0	1

Patient ID	Side	Propensity score	Number of tranfusions	Matched
1001806	1	0.8583781	4	1
1001807	1	0.4745131	0	1
1001808	1	0.6299819	3	1
1001809	2	0.6743881	0	1
1001810	2	0.5427269	1	1
1001811	1	0.9004994	0	0
1001811	2	0.8892304	0	0
1001812	1	0.5312783	2	1
1001813	2	0.4415582	2	0
1001814	1	0.9512951	0	0
1001815	1	0.9366489	0	0
1001816	2	0.5312783	0	1
1001817	1	0.928416	0	0
1001818	2	0.9359804	0	0
1001819	1	0.7586846	0	1
1001820	2	0.8380845	0	0
1001821	1	0.5057742	2	1
1001822	2	0.0986374	10	0
1001823	1	0.1653839	1	0
1001824	1	0.9732879	0	0
1001825	2	0.7846398	0	0
1001826	1	0.6343521	2	1
1001826	2	0.8629828	0	0
1001827	2	0.7211949	0	0
1001828	2	0.378616	0	1
1001829	2	0.4778322	0	1
1001830	2	0.7317822	1	1
1001831	2	0.5542496	4	1
1001832	1	0.56959	1	1
1001833	2	0.6103809	0	1
1001834	2	0.4584335	1	1
1001834	1	0.5740564	1	1
1001835	2	0.6872183	3	1
1001836	1	0.6850716	2	1
1001837	1	0.2727914	0	1
1001838	2	0.0452781	6	0
1001839	1	0.7250152	0	0
1001840	2	0.9010969	0	0
1001841	2	0.7797947	2	1
1001842	1	0.8293664	5	1

Patient ID	Side	Propensity score	Number of tranfusions	Matched
1001843	1	0.7622421	0	0
1001844	2	0.8412643	0	0
1001844	1	0.7014829	0	0
1001845	2	0.8474815	2	1
1001846	2	0.805438	4	1
1001847	1	0.8923432	0	0
1001848	2	0.9512951	0	0
1001849	1	0.9555191	0	0
1001849	2	0.9555191	0	0
1001850	1	0.7994878	2	1
1001850	2	0.7994878	0	1
1001851	2	0.4144451	0	1
1001852	1	0.9317789	0	0
1001853	2	0.914542	0	0
1001854	1	0.9557483	0	0
1001855	1	0.5453769	0	1
1001856	2	0.559449	0	1
1001857	1	0.336925	2	0
1001858	2	0.8465141	7	1
1001859	1	0.7624756	0	0
1001860	2	0.4960584	2	1
1001861	2	0.4271558	0	1
1001862	1	0.5079025	0	1
1001863	1	0.9410451	0	0
1001864	2	0.7317822	0	1
1001864	1	0.8196841	0	0
1001865	1	0.7609577	0	1
1001866	2	0.7582886	2	1
1001867	2	0.3677125	3	1
1001868	2	0.467087	2	1
1001869	1	0.5238844	2	1
1001870	1	0.635016	0	1
1001871	2	0.8143077	4	1
1001872	2	0.9482519	0	0
1001873	2	0.0626577	2	0
1001873	1	0.0626577	6	0
1001874	2	0.3602291	2	0
1001875	2	0.05819	11	0
1001876	1	0.4506037	0	1

Patient ID	Side	Propensity score	Number of tranfusions	Matched
1001876	2	0.622981	0	0
1001877	2	0.9119953	2	1
1001878	1	0.8720679	0	0
1001879	2	0.2888674	0	1
1001880	2	0.810253	0	0
1001881	1	0.4415582	0	1
1001882	2	0.7734786	2	1
1001883	1	0.965012	0	0
1001884	1	0.8926175	0	0
1001885	2	0.9490839	0	0
1001886	1	0.7861992	0	0
1001887	2	0.9145093	0	0
1001888	1	0.714073	1	1
1001889	1	0.9120077	0	0
1001890	1	0.3068618	0	1
1001891	1	0.6586223	0	0
1001892	2	0.488126	0	1
1001893	1	0.9482519	0	0
1001894	1	0.6258056	0	1
1001895	1	0.8556517	0	1
1001895	2	0.7789016	0	0
1001896	1	0.7518332	0	0
1001897	1	0.559449	0	0
1001898	1	0.5797566	4	1
1001899	2	0.914542	0	0
1001899	1	0.8622236	0	0
1001900	2	0.2550914	2	0
1001901	2	0.8448089	2	1
1001902	1	0.7613885	0	1
1001903	1	0.1902203	6	0
1001904	2	0.7994878	2	1
1001905	2	0.8505259	0	0
1001906	2	0.9075149	0	1
1001907	2	0.1563017	2	0
1001908	2	0.0539563	4	0
1001908	1	0.0291376	3	0
1001909	2	0.759277	1	1
1001910	1	0.6977639	0	0
1001911	2	0.7797947	2	1
1001912	2	0.9481091	0	0

Patient ID	Side	Propensity score	Number of tranfusions	Matched
1001912	1	0.9766817	0	0
1001913	2	0.1091269	2	0
1001914	2	0.4900505	0	1
1001915	1	0.882295	0	0
1001916	1	0.6388981	0	0
1001917	2	0.9017812	0	0
1001918	1	0.9538296	0	0
1001919	1	0.0854696	3	0
1001920	1	0.6339748	0	1
1001921	2	0.2595311	6	1
1001922	2	0.3414572	0	1
1001923	1	0.8987589	0	0
1001924	2	0.9684105	0	0
1001925	2	0.3009225	1	1
1001926	2	0.9684361	0	0
1001927	2	0.9827961	0	0
1001928	1	0.7853797	0	1
1001929	1	0.4516916	2	1
1001930	2	0.9569282	0	0
1001931	2	0.9160318	0	0
1001932	2	0.4910648	15	1
1001933	2	0.3545529	0	1
1001934	1	0.9166842	0	0
1001935	1	0.6785855	0	1
1001936	2	0.6674342	0	1
1001937	1	0.6538066	0	0
1001938	2	0.8889729	0	0
1001939	1	0.3065288	1	1
1001940	1	0.5824782	0	0
1001941	1	0.9835454	0	0
1001942	1	0.8004269	0	1
1001943	2	0.500115	2	1
1001944	1	0.6271352	2	1
1001945	1	0.3048106	9	0
1001946	1	0.2992102	2	1
1001947	1	0.9555191	0	0
1001948	2	0.742346	1	1
1001949	2	0.7511338	0	0
1001950	2	0.9040454	0	0

Patient ID	Side	Propensity score	Number of tranfusions	Matched
1001950	1	0.9040454	0	0
1001951	2	0.824614	0	0
1001952	2	0.8858747	0	0
1001953	2	0.5558016	2	1
1001954	1	0.9471623	0	0
1001955	1	0.7783576	0	0
1001956	1	0.5451828	4	1
1001956	2	0.5596484	1	1
1001957	1	0.8543689	0	1
1001958	2	0.2334442	0	1
1001959	2	0.7059057	4	1
1001959	1	0.7059057	4	1
1001960	2	0.8380845	4	1
1001961	2	0.622981	0	1
1001962	2	0.4900505	1	1
1001963	1	0.6179632	2	1
1001964	2	0.2929249	0	1
1001965	2	0.4045151	0	1
1001966	1	0.9557838	0	0
1001966	2	0.9639877	0	0
1001967	1	0.5059837	0	1
1001968	2	0.298614	3	1
1001969	1	0.5460748	2	1
1001970	1	0.2024319	0	1
1001971	1	0.5904373	0	1
1001972	1	0.2853555	2	0
1001972	2	0.4820374	3	1
1001973	2	0.8095856	0	0
1001974	1	0.9766817	0	0
1001975	2	0.5472813	0	1
1001976	2	0.156684	2	0
1001977	2	0.6732838	4	1
1001978	2	0.1649355	4	0
1001979	2	0.4085545	2	1
1001980	1	0.423887	2	1
1001981	1	0.9471208	0	0
1001981	2	0.9471208	0	0
1001982	2	0.4349635	2	1
1001983	1	0.2799763	2	1

Patient ID	Side	Propensity score	Number of tranfusions	Matched
1001984	2	0.9431953	0	0
1001985	2	0.397871	5	1
1001986	2	0.6763938	1	1
1001987	2	0.2206332	4	1
1001988	1	0.4316847	2	1
1001989	2	0.9796864	0	0
1001990	2	0.2511445	2	1
1001991	1	0.9297823	0	0
1001992	1	0.6565705	3	1
1001993	2	0.9359804	0	0
1001994	2	0.1136335	4	0
1001995	2	0.3290067	0	1
1001996	1	0.8909839	0	0
1001997	1	0.039919	3	0
1001998	1	0.7803655	0	0
1001999	1	0.6768638	0	1
1002000	2	0.5038861	0	1
1002001	2	0.7004812	0	0
1002002	2	0.8984905	0	0
1002003	1	0.8380845	0	0
1002004	2	0.5463858	2	1
1002005	1	0.9366489	0	0
1002006	1	0.6315521	0	1
1002007	1	0.8574622	0	1
1002008	1	0.6271352	4	1
1002009	2	0.6288589	2	1
1002010	2	0.8462635	0	1
1002011	1	0.5962059	0	1
1002011	2	0.7357013	0	0
1002012	2	0.8003601	0	1
1002013	1	0.5557157	2	1
1002014	2	0.1290914	1	0
1002015	2	0.0154635	5	0
1002016	2	0.8410779	0	0
1002016	1	0.9493857	2	1
1002017	2	0.9557483	0	0
1002018	1	0.7032168	0	1
1002019	1	0.1125386	0	1
1002020	1	0.9639877	0	0

Patient ID	Side	Propensity score	Number of tranfusions	Matched
1002020	2	0.9639877	0	0
1002021	1	0.3065288	2	0
1002022	1	0.8719391	0	1
1002023	2	0.9493857	0	0
1002024	2	0.8892304	0	0
1002024	1	0.8892304	2	1
1002025	1	0.9684105	0	0
1002026	1	0.6292635	0	1
1002027	2	0.5196691	0	1
1002028	2	0.0900471	12	0
1002029	1	0.8082396	0	1
1002030	1	0.8987589	0	0
1002031	1	0.9557483	0	0
1002032	2	0.9486418	0	0
1002033	2	0.7671505	0	0
1002033	1	0.4817529	0	1
1002034	1	0.8458476	0	0
1002035	2	0.7668433	0	0
1002036	2	0.9555191	0	0
1002037	1	0.739302	0	0
1002037	2	0.7783576	0	0
1002038	1	0.6631602	0	1
1002039	1	0.3479885	0	1
1002040	2	0.6765335	0	1
1002041	1	0.1659664	2	1
1002042	2	0.678968	2	1
1002043	2	0.693725	0	0
1002044	1	0.5045323	2	1
1002045	1	0.6502628	0	0
1002046	2	0.8427618	0	0
1002047	1	0.3155679	1	1
1002048	1	0.9417723	2	1
1002048	2	0.7284777	0	0
1002049	1	0.3560552	0	1
1002050	1	0.588538	11	1
1002051	1	0.711776	0	0
1002052	2	0.0787412	7	0
1002053	2	0.1456953	2	0
1002054	1	0.810253	0	1
1002055	2	0.5038861	0	1

Patient ID	Side	Propensity score	Number of tranfusions	Matched
1002056	1	0.3298604	3	1
1002057	1	0.7511338	0	1
1002058	2	0.5687177	0	1
1002059	1	0.7877594	0	1
1002060	1	0.1698545	2	0
1002061	2	0.8871914	0	0
1002062	2	0.5482672	0	1
1002063	2	0.6343875	0	0
1002064	1	0.3797956	2	0
1002065	1	0.9684105	0	0
1002066	1	0.8823384	2	1
1002066	2	0.7797947	0	0
1002067	1	0.9555191	0	0
1002068	1	0.178546	0	1
1002069	2	0.6624998	0	0
1002070	2	0.6502628	0	0
1002071	1	0.4942396	2	1
1002071	2	0.4796653	0	1
1002072	2	0.7990947	3	1
1002072	1	0.9162551	0	0
1002073	2	0.79514	0	0
1002074	2	0.8857165	2	1
1002075	1	0.8082396	1	1
1002076	2	0.3117949	0	1
1002077	2	0.56959	4	1
1002077	1	0.7175789	0	0
1002078	2	0.143729	1	1
1002079	2	0.8494875	0	0
1002080	1	0.8722887	0	0
1002081	2	0.9706023	0	0
1002082	1	0.9234281	0	0
1002083	2	0.2341951	3	0
1002084	1	0.9266286	0	0
1002085	2	0.3279283	0	1
1002086	1	0.8892304	0	0
1002087	1	0.81357	0	0
1002088	2	0.0901058	2	1
1002089	1	0.9471208	0	0
1002090	2	0.4006762	1	1

Patient ID	Side	Propensity score	Number of tranfusions	Matched
1002091	1	0.8545766	0	1
1002092	1	0.739302	0	0
1002093	2	0.9055791	2	1
1002094	2	0.559449	2	1
1002095	1	0.8380845	0	0
1002096	1	0.9398549	0	0
1002097	1	0.9380204	1	1
1002098	2	0.112277	0	1
1002099	2	0.7053579	0	1
1002099	1	0.5601314	0	1
1002100	1	0.0524178	7	0
1002101	1	0.9115528	0	1
1002102	1	0.6585594	0	0
1002103	2	0.2268098	0	1
1002104	1	0.9555191	0	0
1002105	1	0.5591499	0	1
1002106	1	0.530162	2	1
1002107	1	0.8180251	0	0
1002108	1	0.552885	0	1
1002109	2	0.7346398	0	1
1002110	2	0.7613885	0	1
1002110	1	0.928416	0	0
1002111	1	0.3602076	8	0
1002112	2	0.882295	0	0
1002113	2	0.8464121	0	1
1002114	2	0.9557838	0	0
1002115	1	0.6618387	0	1
1002116	2	0.6082057	0	1
1002117	2	0.8350394	0	0
1002118	1	0.8531178	1	1
1002119	2	0.3748982	4	1
1002120	1	0.5307699	0	1
1002121	2	0.3273235	0	1
1002122	1	0.5740564	0	1
1002123	1	0.1838743	0	1
1002124	2	0.6179632	2	1
1002125	2	0.3564382	4	1
1002126	1	0.6343521	0	1
1002127	1	0.7606473	0	0

Patient ID	Side	Propensity score	Number of tranfusions	Matched
1002128	2	0.8303496	0	1
1002129	1	0.5482672	0	1
1002130	1	0.5864279	2	1
1002131	1	0.8004269	0	0
1002132	1	0.8390119	0	0
1002132	2	0.8390119	0	0
1002133	2	0.6510919	0	0
1002134	2	0.8870113	0	0
1002135	2	0.9639877	0	0
1002136	1	0.8937287	0	0
1002137	2	0.3798259	2	0
1002138	1	0.905477	0	0
1002139	2	0.664922	0	0
1002140	2	0.9468601	0	0
1002140	1	0.9468601	0	0
1002141	2	0.1112753	2	0
1002142	2	0.839206	0	0
1002143	1	0.8160213	0	0
1002144	1	0.3068618	0	1
1002145	1	0.8838318	0	0
1002146	2	0.9802742	0	0
1002147	2	0.9278036	0	0
1002148	2	0.683496	1	1
1002149	2	0.8604312	4	1
1002150	2	0.9366489	0	0
1002151	2	0.9611736	0	0
1002152	1	0.3798259	1	0
1002153	1	0.0834036	1	0
1002154	2	0.9067072	0	0
1002155	2	0.2898611	2	1
1002156	2	0.8815563	0	0
1002157	2	0.9512951	0	0
1002158	2	0.517856	0	1
1002159	2	0.8615083	0	0
1002159	1	0.8583781	0	0
1002160	2	0.8143077	0	0
1002161	2	0.4586092	2	1
1002162	1	0.8390119	0	1
1002163	1	0.8572502	0	1

Patient ID	Side	Propensity score	Number of tranfusions	Matched
1002164	1	0.5041998	0	1
1002165	2	0.8196993	0	0
1002166	2	0.7089199	4	1
1002167	2	0.6361699	0	0
1002168	1	0.3236385	2	1
1002169	1	0.9050807	0	1
1002170	1	0.9278036	0	0
1002170	2	0.8720679	0	0
1002171	1	0.2847618	2	0
1002172	1	0.9366489	0	0
1002173	2	0.7672793	0	1
1002174	2	0.6098851	4	1
1002175	2	0.4963301	3	1
1002176	2	0.280292	0	1
1002177	2	0.5411458	9	1
1002178	1	0.887552	1	1
1002179	1	0.7519121	0	0
1002180	2	0.4766895	0	1
1002181	1	0.4817285	1	1
1002182	1	0.8956693	0	1
1002183	1	0.9568595	0	0
1002184	2	0.8979311	0	0
1002185	1	0.6292635	2	1
1002186	1	0.8182057	0	0
1002187	1	0.8410779	0	0
1002188	1	0.759674	0	1
1002189	1	0.7299379	0	0
1002190	1	0.4209728	0	1
1002191	1	0.9402535	0	0
1002192	1	0.8683234	0	1
1002193	2	0.2164462	2	0
1002194	1	0.0158738	15	0
1002195	2	0.7730775	2	1
1002196	1	0.5602598	0	0
1002197	1	0.7069964	0	1
1002198	2	0.7929086	0	1
1002198	1	0.9623818	0	0
1002199	2	0.710155	0	1
1002199	1	0.4461258	0	1

Patient ID	Side	Propensity score	Number of tranfusions	Matched
1002200	2	0.8558245	0	1
1002201	1	0.7520779	0	0
1002201	2	0.6167231	0	1
1002202	2	0.6591243	0	0
1002203	1	0.9145093	0	0
1002204	2	0.6506863	0	0
1002205	2	0.9482519	0	0
1002206	1	0.5321255	6	1
1002207	1	0.608804	0	1
1002208	1	0.882295	0	0
1002208	2	0.882295	0	0
1002209	1	0.7064988	2	1
1002209	2	0.7815353	0	1
1002210	1	0.4209728	4	1
1002211	1	0.7271124	0	0
1002212	1	0.7559605	0	1
1002213	1	0.5329441	2	1
1002214	2	0.8583781	0	0
1002215	1	0.9481907	0	0
1002216	2	0.0413491	2	0
1002217	1	0.4414355	2	1
1002218	2	0.9163173	0	0
1002219	2	0.8819084	2	1
1002220	2	0.5797566	0	1
1002221	2	0.1011862	2	0
1002222	1	0.5380587	3	1
1002222	2	0.5380587	2	1
1002223	1	0.815839	0	1
1002224	2	0.5685832	0	1
1002225	1	0.6210378	2	1
1002226	1	0.6477159	0	1
1002227	1	0.9454951	0	0
1002228	1	0.467087	0	1
1002229	2	0.7602424	0	0
1002230	2	0.6339748	2	1
1002231	2	0.5350229	4	1
1002232	2	0.132855	7	0
1002233	1	0.8199402	2	1
1002234	1	0.9555191	0	0

Patient ID	Side	Propensity score	Number of tranfusions	Matched
1002235	1	0.8531178	0	0
1002236	2	0.733567	2	1
1002237	2	0.2342159	5	0
1002238	1	0.2135796	4	0
1002239	2	0.9781832	0	0
1002239	1	0.91231	0	0
1002240	2	0.347983	1	1
1002241	2	0.9148016	0	0
1002242	1	0.6909467	0	0
1002243	2	0.2562112	2	1
1002244	1	0.8470115	0	1
1002245	2	0.8627176	0	0
1002246	1	0.7696973	0	1
1002247	1	0.9467011	0	0
1002248	1	0.9277758	0	0
1002248	2	0.8512213	2	1
1002249	2	0.3769238	2	1
1002249	1	0.3514923	3	1
1002250	1	0.8350394	0	0
1002251	1	0.1787831	15	1
1002252	2	0.921033	0	1
1002253	2	0.8531178	0	0
1002254	2	0.5594074	2	1
1002255	2	0.4254257	1	1
1002255	1	0.4254257	0	1
1002256	1	0.1742363	8	0
1002257	2	0.8479683	0	0
1002258	2	0.9128584	0	0
1002259	2	0.6258193	3	1
1002260	1	0.7119912	0	0
1002261	2	0.8103819	0	0
1002262	2	0.031317	4	0
1002263	2	0.4638417	2	1
1002263	1	0.4638417	2	1
1002264	2	0.8464121	2	1
1002265	1	0.7655532	0	1
1002266	2	0.2204023	5	0
1002267	2	0.6745713	0	1
1002268	1	0.7904238	0	1

Patient ID	Side	Propensity score	Number of tranfusions	Matched
1002269	2	0.5898075	0	1
1002270	2	0.8151135	0	0
1002270	1	0.8823384	1	1
1002271	1	0.6909467	0	0
1002272	2	0.8084822	0	1
1002273	2	0.6006756	1	1
1002274	2	0.9530739	0	0
1002275	2	0.6807669	2	1
1002276	1	0.9366489	0	0
1002277	2	0.1708466	2	0
1002278	2	0.467087	2	1
1002279	1	0.5889319	2	1
1002279	2	0.5889319	5	1
1002280	1	0.5057742	3	1
1002281	2	0.8583781	2	1
1002282	2	0.8328427	0	0
1002283	1	0.1416489	6	0
1002284	2	0.0984798	8	0
1002285	1	0.9145093	0	0
1002286	2	0.9569966	0	0
1002287	2	0.2632131	1	0
1002288	1	0.7341151	0	0
1002289	1	0.678968	0	1
1002290	1	0.1736644	0	1
1002291	1	0.6909467	0	0
1002292	2	0.8199402	0	0
1002293	1	0.9482519	0	0
1002294	2	0.9568595	0	0
1002294	1	0.9568595	0	0
1002295	2	0.1080909	0	1
1002296	2	0.0428715	2	0
1002297	1	0.8043698	2	1
1002298	2	0.2620691	2	0
1002299	2	0.5221845	0	1
1002300	1	0.9694243	0	0
1002301	2	0.9288847	0	0
1002302	2	0.8871914	0	0
1002303	2	0.9684105	0	0
1002304	2	0.8700188	0	0
1002305	1	0.9195277	0	0

Patient ID	Side	Propensity score	Number of tranfusions	Matched
1002306	1	0.9120077	0	1
1002307	1	0.0691505	0	1
1002308	1	0.1698545	2	1
1002309	2	0.6288589	2	1
1002310	2	0.9471208	0	1
1002311	1	0.0475722	7	0
1002312	1	0.9743555	2	1
1002313	2	0.8464121	0	0
1002314	2	0.9349987	0	0
1002315	2	0.3830412	0	1
1002316	1	0.436086	3	1
1002316	2	0.7724198	0	1
1002317	2	0.9145093	0	0
1002317	1	0.8979311	0	0
1002318	2	0.973199	0	0
1002319	1	0.4044706	2	1
1002320	1	0.6062345	0	1
1002321	2	0.4882084	0	1
1002322	2	0.9377042	0	0
1002323	1	0.9718675	0	0
1002324	1	0.4139172	0	1
1002325	1	0.9723309	0	0
1002326	1	0.6698383	3	1
1002327	2	0.5466941	0	1
1002328	2	0.4170868	0	1
1002329	1	0.51449	6	1
1002330	2	0.6094672	3	1
1002331	2	0.882295	0	0
1002332	1	0.3250186	0	1
1002333	1	0.0882048	4	0
1002333	2	0.3065288	5	1
1002334	2	0.6538066	0	0
1002335	2	0.9163173	0	0
1002336	2	0.6538066	0	0
1002337	2	0.9684105	0	0
1002338	1	0.3379328	0	1
1002339	1	0.9684105	0	0
1002340	1	0.6283384	0	1
1002340	2	0.6283384	2	1
1002341	1	0.467087	0	1

Patient ID	Side	Propensity score	Number of tranfusions	Matched
1002342	2	0.0137876	9	0
1002343	1	0.837696	0	1
1002344	2	0.6006756	1	1
1002344	1	0.8684553	0	0
1002345	2	0.2799763	0	1
1002346	1	0.2055755	11	0
1002347	1	0.8003601	2	1
1002348	2	0.8515652	0	0
1002349	1	0.1998581	2	0
1002350	1	0.7490443	0	1
1002351	1	0.7705859	0	0
1002352	1	0.7317822	0	1
1002353	1	0.1466258	6	1
1002354	1	0.9486173	0	0
1002355	1	0.678968	1	1
1002356	2	0.9453069	0	0
1002357	2	0.9448927	0	0
1002358	1	0.8823384	0	0
1002359	1	0.5119561	2	1
1002360	1	0.8726236	0	1
1002361	2	0.1114724	4	0
1002362	2	0.0285863	3	0
1002363	1	0.4624865	2	1
1002364	2	0.9081043	0	0
1002364	1	0.6210992	0	0
1002365	1	0.7759103	0	1
1002366	2	0.9395485	0	0
1002367	2	0.3575094	2	0
1002368	2	0.6852827	0	1
1002369	1	0.8817283	0	0
1002370	2	0.5225074	4	1
1002371	2	0.6175798	2	1
1002372	1	0.1136335	5	0
1002373	1	0.9558169	0	0
1002374	2	0.7035234	0	0
1002375	1	0.5991044	3	1
1002376	1	0.3250186	0	1
1002377	1	0.7205255	2	1
1002378	1	0.885538	0	0

Patient ID	Side	Propensity score	Number of tranfusions	Matched
1002379	1	0.8381982	0	0
1002380	1	0.8265822	0	0
1002381	2	0.9781832	0	0
1002382	2	0.9460524	0	0
1002383	1	0.2783999	0	1
1002384	2	0.6658357	2	1
1002385	1	0.9077942	2	1
1002386	1	0.1208212	4	0
1002386	2	0.1756432	2	0
1002387	1	0.4367024	0	1
1002388	2	0.7268058	2	1
1002389	1	0.9555191	0	0
1002390	1	0.0944502	3	0
1002391	1	0.9247656	0	0
1002392	1	0.7010806	0	0
1002393	2	0.3889718	2	0
1002394	2	0.6790591	3	1
1002395	2	0.6271352	5	1
1002396	1	0.8583781	0	0
1002396	2	0.8583781	0	1
1002397	1	0.1859022	2	0
1002398	1	0.4254257	1	1
1002399	2	0.9472872	10	1
1002399	1	0.8626183	0	1
1002400	1	0.559449	3	1
1002401	2	0.8450363	0	0
1002402	2	0.9775355	0	0
1002403	2	0.423243	2	0
1002404	1	0.7999699	0	1
1002405	2	0.6888362	0	1
1002406	1	0.9366489	0	0
1002407	1	0.8111391	0	0
1002408	2	0.8061834	0	1
1002409	1	0.2658993	2	0
1002410	2	0.9718334	0	0
1002411	2	0.8401003	0	0
1002412	1	0.2550914	2	1
1002413	1	0.1509816	0	1
1002414	1	0.882295	0	0
1002415	2	0.9415828	0	0

Patient ID	Side	Propensity score	Number of tranfusions	Matched
1002416	1	0.3065288	2	0
1002417	2	0.9743555	0	0
1002418	2	0.9472042	0	0
1002419	2	0.8505259	0	0
1002420	1	0.3048106	1	0
1002421	1	0.624642	2	1
1002422	1	0.9039453	0	0
1002423	2	0.7338336	4	1
1002424	1	0.5904373	0	1
1002425	2	0.1753001	4	0
1002426	1	0.5412269	0	0
1002427	1	0.2214288	9	0
1002428	2	0.941524	0	0
1002429	1	0.4779756	3	1
1002429	2	0.4779756	0	1
1002430	1	0.1928174	2	0
1002431	1	0.3102176	0	1
1002432	1	0.9653663	0	0
1002433	2	0.9546245	0	0
1002434	1	0.810253	0	0
1002435	1	0.2067997	2	0
1002436	2	0.9142818	0	0
1002437	1	0.6805081	1	1
1002438	1	0.7010806	0	1
1002439	1	0.2801864	4	0
1002440	2	0.9684105	0	0
1002440	1	0.9684105	0	0
1002441	1	0.9120077	0	0
1002442	1	0.9555191	0	0
1002443	2	0.8632497	0	0
1002444	2	0.3730224	6	1
1002445	1	0.6626588	1	1
1002446	2	0.9277478	0	0
1002447	2	0.0273609	12	0
1002448	2	0.8242614	0	0
1002449	1	0.3250186	4	1
1002450	2	0.7010806	1	1
1002451	2	0.9256307	0	0
1002452	1	0.3074901	4	0

Patient ID	Side	Propensity score	Number of tranfusions	Matched
1002453	1	0.3564382	1	1
1002454	2	0.3571163	0	1
1002455	1	0.9342051	0	0
1002456	2	0.1548002	1	0
1002457	1	0.9694243	0	0
1002458	1	0.5402498	0	1
1002459	1	0.6733983	2	1
1002460	1	0.714073	0	0
1002461	2	0.56959	0	1
1002462	2	0.9171911	0	0
1002463	2	0.4244193	0	1
1002464	2	0.3486697	0	1
1002465	1	0.1466258	2	0
1002466	2	0.7516189	0	0
1002467	2	0.7512119	0	0
1002468	2	0.9284884	0	0
1002469	1	0.559449	0	1
1002470	1	0.8410779	0	0
1002471	2	0.7853797	0	0
1002472	1	0.6765335	4	1
1002473	1	0.3250186	0	1
1002474	1	0.6361699	0	0
1002475	1	0.0698281	6	0
1002476	1	0.6365851	0	0
1002477	2	0.8684553	0	0
1002478	2	0.2932329	6	1
1002479	1	0.9283309	0	0
1002480	2	0.0900471	1	1
1002481	2	0.8683234	0	0
1002481	1	0.8615083	0	0
1002482	2	0.7624756	0	0
1002483	2	0.8689915	0	1
1002484	2	0.9646522	0	0
1002485	1	0.8583781	0	1
1002486	1	0.9835454	0	0
1002487	2	0.8143077	0	0
1002488	2	0.8986861	3	1
1002489	2	0.9016513	0	1
1002490	1	0.8196841	0	0

Patient ID	Side	Propensity score	Number of tranfusions	Matched
1002490	2	0.9348254	0	0
1002491	1	0.7853797	2	1
1002492	2	0.774531	2	1
1002493	2	0.1238236	2	0
1002494	2	0.1920066	2	0
1002495	1	0.5791046	0	1
1002496	1	0.335518	0	1
1002497	1	0.473773	2	1
1002498	1	0.9018355	0	0
1002499	2	0.2876948	2	1
1002500	2	0.5307699	0	1
1002501	2	0.3250186	0	1
1002502	1	0.9050807	0	0
1002503	2	0.9457731	0	0
1002503	1	0.9024512	0	0
1002504	1	0.9163173	0	0
1002504	2	0.9163173	0	0
1002505	2	0.6363631	0	1
1002506	2	0.1808962	2	1
1002507	2	0.4774967	12	1
1002508	2	0.0328589	14	0
1002509	2	0.4144451	2	1
1002510	1	0.5038861	0	1
1002511	2	0.2852021	0	1
1002512	2	0.8531178	0	1
1002513	2	0.0739778	14	0
1002514	2	0.7663047	0	0
1002515	2	0.8317252	0	0
1002515	1	0.8234012	0	0
1002516	1	0.8380845	0	1
1002517	2	0.7505902	2	1
1002518	2	0.0698281	4	0
1002519	1	0.5687177	2	1
1002519	2	0.6837346	0	1
1002520	2	0.572865	0	0
1002521	1	0.242314	2	0
1002522	1	0.2236856	0	1
1002523	1	0.1859853	3	0
1002524	2	0.8143077	0	0

Patient ID	Side	Propensity score	Number of tranfusions	Matched
1002525	1	0.9265721	0	1
1002526	1	0.31865	3	0
1002527	1	0.8751222	0	0
1002527	2	0.9472872	0	0
1002528	2	0.8597681	0	0
1002528	1	0.8597681	0	0
1002529	2	0.5493338	0	1
1002530	1	0.5698571	2	1
1002531	1	0.9512951	0	0
1002532	2	0.6743881	4	1
1002532	1	0.6743881	2	1
1002533	2	0.2729049	4	1
1002534	1	0.865247	0	0
1002535	1	0.9266286	0	0
1002536	1	0.9684105	0	0
1002537	1	0.6909467	0	0
1002538	1	0.1578028	1	1
1002539	1	0.1925025	5	0
1002540	1	0.8208233	0	0
1002541	2	0.5408134	3	1
1002541	1	0.5198111	0	1
1002542	1	0.9898134	0	0
1002543	1	0.0755955	2	0
1002544	2	0.806943	2	1
1002545	1	0.8541505	0	0
1002546	1	0.87502	0	0
1002547	1	0.6003014	5	1
1002548	2	0.9675624	0	0
1002549	2	0.7266501	0	0
1002550	2	0.683496	0	1
1002551	2	0.1405075	2	0
1002552	1	0.2860811	2	1
1002553	2	0.2932256	0	1
1002554	2	0.7064988	0	1
1002555	1	0.9796864	0	0
1002556	2	0.1133417	2	1
1002556	1	0.3250186	1	1
1002557	2	0.8043698	1	1
1002558	1	0.755473	0	0

Patient ID	Side	Propensity score	Number of tranfusions	Matched
1002559	1	0.9031482	0	0
1002560	1	0.756538	0	1
1002561	1	0.4882489	0	1
1002562	2	0.8159437	0	0
1002563	1	0.9639877	0	0
1002564	2	0.8242614	0	0
1002565	2	0.9366489	0	0
1002566	2	0.7656533	2	1
1002567	2	0.9532798	0	0
1002568	2	0.5276367	2	1
1002569	2	0.9342051	1	1
1002570	2	0.6637491	2	1
1002571	1	0.9364763	0	0
1002572	2	0.0742841	4	0
1002573	2	0.4037822	1	1
1002574	2	0.8566947	0	0
1002574	1	0.8565918	0	1
1002575	1	0.8987589	0	0
1002576	1	0.810253	0	0
1002577	2	0.5309452	0	1
1002578	2	0.6095358	0	1
1002579	1	0.9555191	0	0
1002580	1	0.9039453	0	0
1002580	2	0.8120872	0	0
1002581	1	0.831569	0	1
1002581	2	0.831569	1	1
1002582	1	0.653528	0	1
1002583	1	0.5585162	0	1
1002583	2	0.5585162	0	1
1002584	2	0.8844555	0	0
1002585	2	0.7838778	2	1
1002586	1	0.1486094	2	0
1002587	2	0.9743555	0	0
1002588	1	0.1410131	7	0
1002589	1	0.9134939	0	0
1002590	1	0.423243	2	1
1002591	1	0.9166842	0	0
1002591	2	0.9811312	0	0
1002592	1	0.7877594	0	1

Patient ID	Side	Propensity score	Number of tranfusions	Matched
1002593	1	0.1020669	0	1
1002594	2	0.9743555	0	0
1002595	1	0.7948355	0	0
1002595	2	0.9321146	0	0
1002596	1	0.678968	0	0
1002597	2	0.2067997	2	1
1002598	2	0.9568595	0	0
1002599	1	0.8159437	0	0
1002600	1	0.8925282	0	0
1002601	2	0.9284884	0	0
1002601	1	0.9284884	0	0
1002602	1	0.9530621	0	0
1002603	1	0.1758676	2	0
1002604	2	0.9377239	0	0
1002605	2	0.882295	0	0
1002606	2	0.6210378	2	1
1002607	1	0.4735617	0	1
1002608	1	0.3464321	0	1
1002609	1	0.6715935	0	1
1002610	1	0.9119742	0	0
1002611	2	0.3711177	1	0
1002612	1	0.9673688	0	0
1002613	1	0.8652821	0	0
1002614	2	0.2707317	0	1
1002615	1	0.9395485	0	0
1002616	2	0.4912046	6	1
1002617	2	0.7622421	0	0
1002618	2	0.9781832	0	0
1002619	1	0.3935633	3	0
1002620	1	0.0283976	10	0
1002621	2	0.9680528	0	0
1002622	1	0.8926175	0	0
1002623	1	0.6653261	0	1
1002624	2	0.3620051	0	1
1002625	1	0.8968255	0	0
1002626	1	0.2193855	2	0
1002627	2	0.0526381	2	0
1002628	1	0.5904373	2	1
1002628	2	0.5904373	2	1

Patient ID	Side	Propensity score	Number of tranfusions	Matched
1002629	1	0.588538	2	1
1002630	1	0.3455079	2	1
1002631	2	0.9406359	0	0
1002632	1	0.7464717	0	0
1002633	2	0.4044706	0	1
1002634	1	0.3250186	6	1
1002634	2	0.3250186	2	1
1002635	2	0.8720679	0	0
1002636	2	0.4367024	0	1
1002637	1	0.6842546	0	0
1002638	2	0.0742841	2	0
1002639	1	0.8128405	0	0
1002639	2	0.8128405	0	0
1002640	2	0.6628244	0	1
1002641	1	0.739302	4	1
1002642	1	0.9366489	0	0
1002643	1	0.9070476	0	0
1002644	1	0.8744204	4	1
1002645	1	0.2673095	2	1
1002646	1	0.918583	0	0
1002647	2	0.61813	2	1
1002648	2	0.2206332	4	0
1002649	1	0.6631602	0	0
1002650	1	0.969645	0	0
1002651	2	0.8722887	0	0
1002652	1	0.0201546	4	0
1002653	1	0.8558245	2	1
1002654	2	0.1779538	20	0
1002655	1	0.7717639	0	1
1002655	2	0.9342051	0	0
1002656	2	0.5687177	2	1
1002657	2	0.4802082	2	1
1002658	1	0.642688	0	1
1002658	2	0.9684105	0	0
1002659	2	0.9624106	0	0
1002660	2	0.8462212	0	0
1002660	1	0.9006122	4	1
1002661	1	0.1624789	13	0
1002662	1	0.9653663	0	0

Patient ID	Side	Propensity score	Number of tranfusions	Matched
1002663	2	0.0272167	17	0
1002664	2	0.678968	2	1
1002665	1	0.9512951	0	0
1002666	2	0.4209728	0	1
1002666	1	0.473773	2	1
1002667	2	0.3689615	2	1
1002668	2	0.6842505	0	1
1002669	2	0.6264614	0	1
1002670	2	0.5983813	2	1
1002671	2	0.6075645	2	1
1002672	1	0.6210378	0	1
1002673	1	0.9278036	0	0
1002674	1	0.8979311	0	0
1002675	2	0.6935708	0	0
1002676	1	0.9743555	0	0
1002676	2	0.9743555	0	0
1002677	2	0.9005415	2	1
1002678	2	0.9413932	0	0
1002679	2	0.9471208	0	0
1002680	2	0.7199393	0	0
1002681	1	0.9796864	0	0
1002682	2	0.928416	0	0
1002683	1	0.7175789	0	0
1002684	1	0.9569966	0	0
1002685	1	0.467087	0	1
1002686	1	0.9172093	0	0
1002687	1	0.4872598	2	1
1002688	2	0.2204541	2	0
1002689	1	0.2283806	0	1
1002690	2	0.56959	1	1
1002691	1	0.3385494	2	1
1002692	2	0.7315358	0	1
1002693	1	0.8182057	0	0
1002694	1	0.9457731	0	0
1002695	1	0.7459386	2	1
1002696	1	0.9000514	0	0
1002697	1	0.8895731	0	0
1002697	2	0.9383534	0	0
1002698	2	0.8708875	0	0

Patient ID	Side	Propensity score	Number of tranfusions	Matched
1002699	1	0.7783576	0	0
1002700	2	0.8448089	0	0
1002701	2	0.8265822	0	0
1002701	1	0.9568595	0	0
1002702	1	0.8652821	0	0
1002703	2	0.5153931	0	1
1002704	1	0.4203475	0	1
1002705	2	0.6210378	0	1
1002706	2	0.6343521	0	0
1002707	2	0.172388	0	1
1002708	2	0.4440603	0	1
1002709	2	0.9603292	0	0
1002710	2	0.978361	0	0
1002710	1	0.978361	0	0
1002711	1	0.9342051	0	0
1002712	2	0.9538277	0	0
1002713	1	0.5584911	0	1
1002713	2	0.8176521	0	0
1002714	2	0.9550222	0	0
1002715	1	0.8182057	0	0
1002716	1	0.8247408	5	1
1002717	2	0.3057131	5	0
1002718	1	0.1871392	2	0
1002719	2	0.0331518	6	0
1002720	1	0.8354647	0	0
1002721	2	0.1172352	3	0
1002722	2	0.8348097	0	1
1002723	1	0.6658357	4	1
1002724	1	0.0604472	3	0
1002725	2	0.5745877	0	1
1002726	1	0.7215596	0	1
1002727	1	0.714073	0	1
1002727	2	0.9398549	0	0
1002728	2	0.9684105	0	0
1002729	2	0.8926175	0	0
1002730	1	0.2591388	3	0
1002731	1	0.5352928	1	1
1002732	2	0.9120077	0	0
1002733	2	0.9559048	0	0
1002734	1	0.2237398	2	1

Patient ID	Side	Propensity score	Number of tranfusions	Matched
1002735	1	0.4615932	2	1
1002736	1	0.8143077	0	0
1002737	1	0.9339128	0	0
1002738	1	0.4435972	2	1
1002739	1	0.4439202	5	1
1002740	1	0.9041181	0	0
1002741	1	0.7613885	1	1
1002741	2	0.7240763	0	0
1002742	2	0.1859022	3	1
1002743	1	0.8823384	0	0
1002744	1	0.467087	0	1
1002745	1	0.1806238	0	1
1002746	2	0.9395485	0	0
1002747	2	0.8683234	0	1
1002747	1	0.559449	0	1
1002748	2	0.9128584	0	0
1002749	1	0.8699323	0	0
1002750	1	0.817672	0	0
1002751	2	0.9173771	0	0
1002752	2	0.9119742	0	0
1002753	1	0.321832	2	1
1002754	1	0.6432483	0	1
1002754	2	0.4888588	0	1
1002755	2	0.7610734	0	0
1002755	1	0.9380204	0	0
1002756	1	0.3951615	0	1
1002757	1	0.8923432	0	0
1002758	1	0.8242864	0	0
1002759	1	0.7838778	0	0
1002760	2	0.7834761	0	0
1002760	1	0.7834761	0	0
1002761	2	0.6739856	0	0
1002762	2	0.7451044	0	0
1002763	1	0.0457419	4	0
1002764	1	0.7537837	0	0
1002765	2	0.1020288	1	1
1002766	2	0.5716151	0	1
1002767	2	0.380564	4	1
1002768	2	0.9197811	0	1

Patient ID	Side	Propensity score	Number of tranfusions	Matched
1002769	1	0.041006	3	0
1002770	2	0.8186172	0	1
1002771	2	0.9530739	0	0
1002772	1	0.9145093	0	0
1002773	2	0.0640174	11	0
1002774	1	0.6586223	0	0
1002775	1	0.9197193	0	0
1002776	2	0.6308935	4	1
1002777	2	0.1255288	5	0
1002778	2	0.3601328	2	0
1002778	1	0.9207713	0	0
1002779	1	0.5542496	3	1
1002780	1	0.969645	0	0
1002781	1	0.56959	2	1
1002782	2	0.9043188	0	0
1002783	1	0.1483754	0	1
1002784	2	0.3133725	0	1
1002785	1	0.2550914	0	1
1002786	1	0.4724157	4	1
1002787	2	0.7683856	0	1
1002788	2	0.2992102	3	1
1002789	1	0.8708168	0	0
1002790	2	0.2262191	3	0
1002791	1	0.8242614	0	0
1002792	1	0.9043188	0	0
1002793	1	0.8199402	0	1
1002794	1	0.9301074	0	0
1002795	1	0.6576294	0	0
1002796	1	0.8150505	0	0
1002797	2	0.9653663	0	0
1002798	2	0.5810471	0	0
1002799	1	0.8678728	0	0
1002799	2	0.8982738	0	0
1002800	2	0.9653663	0	0
1002801	1	0.9366489	0	1
1002802	1	0.7451044	2	1
1002803	1	0.4983625	0	1
1002804	1	0.2350999	0	1
1002805	1	0.248998	2	1
1002806	1	0.3250186	2	1

Patient ID	Side	Propensity score	Number of tranfusions	Matched
1002807	1	0.5740564	0	1
1002808	1	0.6062345	0	1
1002809	2	0.8684553	0	0
1002810	2	0.6339748	0	1
1002811	1	0.9099468	0	0
1002811	2	0.948741	0	0
1002812	1	0.4715458	0	1
1002813	2	0.561905	2	1
1002814	2	0.8551245	0	1
1002815	1	0.8151238	0	0
1002816	2	0.9281372	0	0
1002817	2	0.9121083	0	0
1002818	1	0.8766375	5	1
1002819	2	0.8684553	0	0
1002819	1	0.9256307	0	0
1002820	2	0.0444392	4	0
1002821	1	0.683496	0	1
1002822	1	0.7556597	2	1
1002823	2	0.3602291	0	1
1002824	2	0.9743555	0	0
1002825	2	0.6939692	0	1
1002826	1	0.3940368	0	1
1002827	2	0.9684105	0	0
1002828	2	0.2799763	4	1
1002829	1	0.7783576	0	0
1002830	1	0.5964152	0	1
1002831	1	0.8448089	0	0
1002832	1	0.242314	2	1
1002833	2	0.3665214	6	1
1002834	1	0.8923432	0	0
1002835	2	0.9472872	0	0
1002836	1	0.8770538	1	1
1002837	2	0.8143077	0	0
1002838	2	0.7998903	2	1
1002839	1	0.9380204	0	0
1002840	1	0.273316	2	0
1002841	1	0.7863965	0	0
1002842	2	0.7399326	0	0
1002843	1	0.965933	0	0
1002844	2	0.3250186	2	1

Patient ID	Side	Propensity score	Number of tranfusions	Matched
1002845	2	0.7470569	0	0
1002846	1	0.2604887	0	1
1002847	1	0.7564785	0	0
1002848	2	0.4549305	0	1
1002849	1	0.882295	0	0
1002850	2	0.6801305	0	0
1002851	1	0.5411458	2	1
1002852	2	0.9402535	0	0
1002853	1	0.678968	0	1
1002854	2	0.9706023	0	0
1002855	1	0.5347764	2	1
1002856	1	0.2534086	0	1
1002857	2	0.8591355	0	0
1002858	1	0.7558137	0	0
1002859	1	0.8887281	0	1
1002860	2	0.864351	0	0
1002861	1	0.9712601	0	0
1002862	2	0.5347764	0	1
1002863	1	0.9029307	0	0
1002864	2	0.6419219	5	1
1002865	1	0.8234012	0	0
1002866	2	0.7451044	0	0
1002867	1	0.7751945	0	0
1002867	2	0.9380204	0	0
1002868	2	0.7454145	0	1
1002869	1	0.8380845	0	0
1002870	1	0.8199402	0	0
1002871	2	0.8380845	0	0
1002872	2	0.473773	2	1
1002873	1	0.9171911	0	0
1002873	2	0.9765373	0	0
1002874	1	0.2807186	2	0
1002875	2	0.8819084	0	0
1002876	2	0.9653663	0	0
1002877	1	0.8859565	0	0
1002878	2	0.9380204	0	0
1002879	1	0.9443064	0	0
1002880	1	0.7007701	2	1
1002881	2	0.8253745	2	1

Patient ID	Side	Propensity score	Number of tranfusions	Matched
1002882	1	0.6121385	3	1
1002883	1	0.9380204	2	1
1002884	2	0.3795239	1	1
1002885	1	0.9207713	0	0
1002886	2	0.9512951	0	0
1002887	1	0.5395649	2	1
1002888	2	0.8381982	0	0
1002889	2	0.5937697	1	1
1002890	1	0.3503485	4	1
1002891	1	0.8065736	0	0
1002892	1	0.9623802	0	0
1002893	2	0.806943	0	0
1002894	1	0.3451385	0	1
1002895	1	0.8651406	0	0
1002896	1	0.6248208	0	0
1002897	2	0.6116308	0	1
1002898	2	0.7583805	0	0
1002899	1	0.6339748	2	1
1002900	1	0.7556121	7	1
1002901	2	0.640985	4	1
1002902	1	0.7428126	0	0
1002903	2	0.5740564	0	0
1002903	1	0.5740564	0	1
1002904	1	0.1460613	2	0
1002905	1	0.8578383	0	0
1002906	1	0.9568595	0	0
1002907	1	0.7730314	0	1
1002908	1	0.5737743	0	1
1002909	1	0.9366248	0	0
1002910	1	0.9395485	0	0
1002911	2	0.1460613	4	0
1002912	2	0.9796864	0	0
1002913	1	0.4634502	0	1
1002914	2	0.1381117	3	0
1002915	2	0.4710574	7	1
1002916	2	0.810253	0	0
1002917	1	0.6857437	0	1
1002918	1	0.9653663	0	0
1002919	2	0.7808636	0	0

Patient ID	Side	Propensity score	Number of tranfusions	Matched
1002919	1	0.7808636	0	0
1002920	1	0.9307857	0	0
1002921	2	0.9527564	0	0
1002922	2	0.9684105	0	0
1002923	1	0.7059057	0	1
1002924	2	0.5663911	4	1
1002925	1	0.1020288	2	1
1002926	1	0.7145118	3	1
1002927	1	0.925054	0	0
1002928	1	0.9603292	0	0
1002929	1	0.2550914	2	1
1002930	2	0.6210378	1	1
1002931	1	0.2982801	2	1
1002932	2	0.7853797	0	0
1002933	1	0.8671572	0	1
1002934	1	0.559449	0	1
1002935	1	0.1319859	3	0
1002936	2	0.3886761	0	1
1002937	1	0.8949718	0	1
1002938	2	0.0900138	1	0
1002939	2	0.7441412	0	0
1002939	1	0.9068288	0	0
1002940	1	0.8683234	0	0
1002941	2	0.8494818	0	0
1002942	1	0.2222123	2	0
1002943	1	0.242314	3	1
1002943	2	0.242314	3	0
1002944	2	0.2685936	4	1
1002945	2	0.7199393	4	1
1002946	2	0.7564785	0	0
1002947	2	0.8532444	0	0
1002948	2	0.9493857	0	1
1002949	2	0.3504041	0	1
1002950	1	0.9075149	0	1
1002951	2	0.9809673	0	0
1002952	2	0.6075645	0	1
1002952	1	0.6075645	2	1
1002953	2	0.7613885	0	1
1002954	1	0.740117	0	0
1002955	2	0.9454951	0	0

Patient ID	Side	Propensity score	Number of tranfusions	Matched
1002956	2	0.2875349	0	1
1002957	2	0.3391415	1	0
1002958	1	0.1024967	10	0
1002959	2	0.1546398	2	0
1002960	1	0.8726236	1	1
1002961	2	0.4375777	0	1
1002962	2	0.9372145	0	0
1002963	1	0.8381982	0	0
1002964	1	0.8061834	0	1
1002965	2	0.7451837	0	0
1002966	1	0.318691	1	0
1002967	2	0.0086626	8	0
1002968	1	0.1445133	7	0
1002969	2	0.2998132	0	1
1002970	1	0.5957898	1	1
1002970	2	0.5957898	0	1
1002971	2	0.5234918	8	1
1002972	2	0.2997649	4	1
1002972	1	0.7587227	2	1
1002973	1	0.3858944	5	1
1002974	2	0.2341951	2	0
1002975	2	0.2325698	2	0
1002976	2	0.321832	2	0
1002977	2	0.714073	0	0
1002978	2	0.9395485	2	1
1002979	1	0.6349469	0	0
1002980	1	0.8151238	0	0
1002981	1	0.3250186	5	1
1002982	2	0.321832	4	0
1002983	1	0.4226732	4	0
1002984	1	0.9482975	0	0
1002985	2	0.4802569	0	1
1002986	1	0.7451044	0	0
1002987	2	0.837696	2	1
1002987	1	0.837696	0	0
1002988	1	0.6451731	0	0
1002989	2	0.9276642	0	0
1002990	2	0.6637491	2	1
1002991	1	0.2445827	0	1

Patient ID	Side	Propensity score	Number of tranfusions	Matched
1002992	1	0.8552614	0	0
1002993	2	0.8049161	0	0
1002994	1	0.2458921	2	0
1002995	2	0.9656805	0	0
1002995	1	0.9613422	0	0
1002996	1	0.0574686	2	0
1002997	2	0.6177862	0	1
1002998	2	0.8390119	0	0
1002999	2	0.4324012	0	1
1003000	1	0.8651406	0	0
1003001	2	0.5416342	0	1
1003002	1	0.9653663	0	0
1003003	1	0.8512081	3	1
1003004	1	0.894368	2	1
1003005	1	0.6456687	0	1
1003006	2	0.0741431	4	0
1003007	1	0.4516916	6	1
1003008	2	0.9197193	0	0
1003009	2	0.8590019	0	0
1003009	1	0.9077942	0	1
1003010	1	0.8104353	0	0
1003010	2	0.8268883	0	0
1003011	1	0.8438831	2	1
1003012	1	0.9555191	0	0
1003013	2	0.9668489	0	0
1003014	2	0.4882084	1	1
1003015	2	0.9472872	0	0
1003016	1	0.0121565	14	0
1003017	1	0.0770133	2	0
1003018	2	0.2741648	0	1
1003019	1	0.4900505	0	1
1003020	2	0.2939608	2	0
1003021	2	0.7858006	0	0
1003021	1	0.7858006	0	0
1003022	1	0.9017812	0	0
1003023	1	0.7451044	0	0
1003024	1	0.0320789	15	0
1003025	1	0.5591499	0	1
1003026	1	0.9040454	0	0
1003027	1	0.6321596	0	1

Patient ID	Side	Propensity score	Number of tranfusions	Matched
1003028	1	0.808008	0	0
1003029	2	0.8479526	0	1
1003029	1	0.8479526	2	1
1003030	1	0.8496354	0	1
1003031	1	0.5827996	1	1
1003032	2	0.6808684	2	1
1003033	1	0.4924099	0	1
1003034	1	0.965012	0	0
1003034	2	0.9811312	0	0
1003035	1	0.9568595	0	0
1003036	1	0.8486877	0	0
1003036	2	0.8843186	0	0
1003037	2	0.8287051	0	0
1003038	2	0.6723223	0	1
1003039	1	0.9050807	0	0
1003040	1	0.473773	1	1
1003041	1	0.0289247	3	0
1003042	1	0.0675463	9	0
1003043	1	0.9624106	0	0
1003044	1	0.8651406	0	0
1003045	2	0.4626784	0	1
1003046	1	0.8483376	0	0
1003047	2	0.1366643	1	0
1003048	2	0.1087145	7	0
1003048	1	0.1981528	4	1
1003049	1	0.9512951	0	0
1003050	1	0.1132905	0	1
1003051	2	0.7326774	0	1
1003052	1	0.5808195	0	0
1003053	1	0.6396647	0	1
1003054	2	0.3697271	0	1
1003055	1	0.8483376	0	1
1003056	2	0.9095091	0	0
1003057	2	0.8983983	0	1
1003058	1	0.9827961	0	0
1003059	1	0.8464121	0	0
1003059	2	0.9512951	0	0
1003060	2	0.4177947	7	1
1003061	1	0.4475346	0	1

Patient ID	Side	Propensity score	Number of tranfusions	Matched
1003062	2	0.9197193	0	0
1003063	1	0.9405539	0	0
1003064	1	0.9653663	0	0
1003065	1	0.7696973	0	1
1003066	2	0.9380691	0	0
1003067	2	0.9380204	0	1
1003068	1	0.9380204	0	1
1003069	1	0.817672	0	0
1003070	1	0.3528668	3	0
1003071	1	0.4415582	2	0
1003072	1	0.9288847	0	1
1003073	1	0.2231884	4	0
1003074	2	0.3133725	1	1
1003075	2	0.9796864	0	0
1003076	2	0.9380204	2	1
1003077	1	0.9222556	0	0
1003078	1	0.9611736	0	0
1003079	2	0.9555191	0	0
1003080	2	0.8651406	0	0
1003081	1	0.9623802	0	0
1003082	1	0.9395485	0	0
1003083	1	0.950773	0	0
1003084	2	0.9317789	0	0
1003085	1	0.4397827	4	1
1003086	1	0.5262407	0	1
1003087	1	0.5920472	0	1
1003088	2	0.2240943	0	1
1003089	1	0.9119742	0	0
1003090	2	0.9521189	0	0
1003091	1	0.4435972	0	1
1003092	2	0.4942396	0	1
1003093	2	0.5309048	4	1
1003094	1	0.2860811	0	1
1003095	1	0.7564785	0	0
1003096	1	0.9186629	3	1
1003097	2	0.4390605	0	1
1003098	2	0.9527564	0	0
1003099	1	0.3790332	2	1
1003100	2	0.8317252	0	0
1003101	2	0.7910648	2	1

Patient ID	Side	Propensity score	Number of tranfusions	Matched
1003102	1	0.7655532	2	1
1003103	2	0.810253	0	0
1003104	2	0.8622236	0	0
1003105	1	0.9262035	0	0
1003106	1	0.6299819	0	0
1003107	1	0.3995178	2	1
1003108	1	0.9071183	0	0
1003109	1	0.7294804	3	1
1003110	2	0.8893129	0	0
1003111	2	0.5211484	2	1
1003111	1	0.5211484	2	1
1003112	2	0.3658852	0	1
1003113	2	0.9639877	0	0
1003114	1	0.2236856	0	1
1003115	1	0.2923339	2	0
1003116	1	0.6665623	4	1
1003117	1	0.8048824	0	0
1003118	2	0.9684105	0	0
1003119	1	0.5904373	0	1
1003120	1	0.6084571	0	0
1003121	2	0.0974903	2	0
1003122	1	0.6349469	0	1
1003123	1	0.9353824	0	0
1003124	2	0.3399404	4	1
1003125	1	0.3013975	2	1
1003126	2	0.9555191	0	0
1003127	2	0.0330376	14	0
1003128	2	0.810253	0	0
1003129	1	0.6544533	3	1
1003130	1	0.9555191	0	1
1003131	1	0.7077939	0	1
1003132	1	0.5196408	0	1
1003133	1	0.6477159	0	0
1003134	2	0.5740564	0	0
1003135	1	0.2182333	4	0
1003136	1	0.4044706	0	1
1003137	1	0.7146404	1	1
1003138	2	0.9271049	0	0
1003139	1	0.4376201	1	0
1003140	2	0.9898134	0	0

Patient ID	Side	Propensity score	Number of tranfusions	Matched
1003141	1	0.9775355	0	0
1003142	1	0.8199402	0	0
1003143	2	0.87026	4	1
1003144	1	0.1304628	2	0
1003145	1	0.6210378	0	0
1003146	1	0.8342246	0	0
1003147	2	0.8268883	0	0
1003148	1	0.7634533	2	1
1003149	1	0.1329063	3	0
1003150	1	0.9557483	0	0
1003151	1	0.2517358	4	1
1003152	2	0.6544533	2	1
1003153	2	0.8626183	3	1
1003154	2	0.622981	2	1
1003155	1	0.481933	0	1
1003156	2	0.7010806	0	1
1003157	1	0.7567775	0	0
1003158	1	0.4927622	0	1
1003159	1	0.3285485	0	1
1003160	1	0.6308935	1	1
1003161	1	0.7138674	1	1
1003162	1	0.9680528	0	0
1003163	1	0.5737743	0	1
1003164	1	0.9119742	0	0
1003165	1	0.6516405	0	1
1003166	2	0.9145093	0	0
1003167	2	0.7283342	0	1
1003168	2	0.467087	0	1
1003169	2	0.6966127	0	1
1003170	2	0.467087	2	1
1003171	1	0.8358088	0	0
1003172	1	0.622981	2	1
1003173	1	0.7240763	0	0
1003174	1	0.7541507	0	0
1003175	2	0.3458062	0	1
1003176	1	0.8531178	0	0
1003177	2	0.6210378	0	0
1003178	2	0.8569584	0	1
1003179	2	0.5347764	0	1

Patient ID	Side	Propensity score	Number of tranfusions	Matched
1003180	2	0.8291816	0	0
1003181	1	0.7145118	0	1
1003182	1	0.2458921	2	0
1003183	1	0.0488324	3	0
1003184	1	0.3282734	2	0
1003185	2	0.9366737	0	0
1003185	1	0.9366737	0	1
1003186	2	0.1267193	2	0
1003187	1	0.8615083	0	0
1003187	2	0.9120077	0	0
1003188	1	0.1764928	2	0
1003189	1	0.6732364	1	1
1003190	2	0.1488857	4	0
1003191	1	0.4619625	0	1
1003191	2	0.4619625	1	1
1003192	2	0.8023827	0	0
1003193	1	0.9718675	0	0
1003194	2	0.6182663	1	1
1003195	1	0.8583781	0	1
1003195	2	0.8583781	0	0
1003196	1	0.6538066	4	1
1003197	2	0.1738388	4	1
1003197	1	0.56959	0	1
1003198	1	0.8830277	0	0
1003199	2	0.6172903	2	1
1003200	2	0.9555191	0	0
1003201	1	0.8923432	0	1
1003202	1	0.2024319	3	0
1003203	1	0.9653663	0	0
1003204	1	0.957775	0	0
1003205	2	0.9145093	0	0
1003206	1	0.7994878	0	1
1003207	1	0.3250186	0	1
1003208	1	0.7852786	2	1
1003209	1	0.9222556	0	0
1003210	2	0.9219838	0	0
1003211	1	0.368443	0	1
1003212	1	0.5234918	2	1
1003213	2	0.1702515	4	0
1003214	1	0.9133362	0	0

Patient ID	Side	Propensity score	Number of tranfusions	Matched
1003215	2	0.9557483	0	0
1003216	1	0.9380204	0	0
1003217	1	0.9266286	0	0
1003218	2	0.2554295	0	1
1003219	2	0.9743555	0	0
1003220	1	0.588538	2	1
1003221	1	0.9000485	0	1
1003222	2	0.9395485	0	0
1003222	1	0.8143077	0	0
1003223	1	0.242314	2	0
1003224	2	0.5057742	3	1
1003225	1	0.793514	0	1
1003226	2	0.3609962	2	1
1003227	1	0.8309514	0	0
1003228	2	0.79758	26	1
1003229	2	0.0475925	4	0
1003230	1	0.1474643	2	0
1003231	2	0.9145093	0	0
1003231	1	0.823929	0	0
1003232	1	0.8949718	1	1
1003233	1	0.3466033	0	1
1003234	2	0.4615932	2	1
1003235	2	0.8418481	0	1
1003236	2	0.9053989	0	0
1003237	2	0.9684105	0	0
1003238	1	0.2525808	0	1
1003238	2	0.559449	0	1
1003239	2	0.8297242	0	0
1003240	1	0.678968	0	0
1003241	1	0.3774182	2	1
1003242	2	0.9089601	0	0
1003242	1	0.9467011	0	0
1003243	2	0.3749073	0	1
1003243	1	0.3749073	0	1
1003244	1	0.7865697	2	1
1003245	1	0.7577626	0	0
1003245	2	0.8476354	0	0
1003246	1	0.9607499	4	1
1003247	1	0.7339152	0	0
1003248	2	0.7275832	3	1

Patient ID	Side	Propensity score	Number of tranfusions	Matched
1003249	1	0.4051959	11	1
1003250	1	0.7053579	5	1
1003251	2	0.2426323	5	0
1003252	1	0.1067472	4	0
1003253	2	0.8196841	0	1
1003254	1	0.7175789	0	0
1003254	2	0.7175789	0	0
1003255	2	0.963065	0	0
1003255	1	0.963065	0	0
1003256	2	0.1715548	9	0
1003257	2	0.6114708	10	1
1003258	1	0.8505259	0	0
1003259	2	0.4044706	0	1
1003259	1	0.6544533	0	1
1003260	1	0.9339128	0	0
1003261	1	0.1719784	3	0
1003262	2	0.2216177	1	0
1003263	1	0.7911339	0	1
1003264	2	0.2648933	3	1
1003265	1	0.9568595	0	0
1003266	1	0.1223756	7	0
1003267	1	0.9712601	0	0
1003268	1	0.8512175	0	0
1003269	1	0.9128584	0	0
1003270	2	0.4941244	0	1
1003271	2	0.9743555	0	0
1003272	1	0.9457731	0	0
1003273	2	0.6437349	0	1
1003274	2	0.7451044	0	0
1003274	1	0.5542496	0	1
1003275	1	0.8989661	0	0
1003276	2	0.6826809	8	1
1003277	1	0.6370454	0	0
1003278	1	0.0951333	5	1
1003279	2	0.9395485	0	0
1003280	1	0.2932512	2	0
1003281	1	0.8684553	0	0
1003282	1	0.242314	7	0
1003283	1	0.6339748	2	1

Patient ID	Side	Propensity score	Number of tranfusions	Matched
1003284	2	0.678968	0	0
1003285	1	0.1698545	3	0
1003286	1	0.555441	2	1
1003287	2	0.2236856	1	1
1003288	1	0.5312783	0	1
1003289	1	0.8125943	0	1
1003290	1	0.928416	0	0
1003290	2	0.928416	0	0
1003291	2	0.9471208	0	0
1003292	1	0.3250186	2	1
1003293	1	0.6872183	0	1
1003294	2	0.8464121	0	1
1003295	2	0.5619304	0	1
1003296	2	0.1766384	2	0
1003297	1	0.9017256	0	0
1003298	1	0.9301074	0	0
1003299	1	0.2095772	0	1
1003300	1	0.9119742	0	0
1003301	2	0.8614129	0	0
1003302	2	0.6586223	4	1
1003303	2	0.8569771	2	1
1003304	2	0.9743555	0	0
1003305	1	0.843828	0	0
1003306	2	0.804426	0	1
1003306	1	0.481972	0	1
1003307	1	0.3504041	2	0
1003308	1	0.8234012	0	0
1003309	1	0.2473879	2	0
1003310	2	0.810253	0	0
1003311	1	0.0416819	2	0
1003312	2	0.9775355	0	0
1003313	1	0.6805081	2	1
1003314	1	0.321832	0	1
1003315	2	0.9684105	0	1
1003316	1	0.9557483	0	0
1003317	1	0.9538277	0	0
1003318	2	0.897856	0	0
1003319	1	0.1229046	2	0
1003320	2	0.8119574	0	0
1003321	2	0.2939064	2	0

Patient ID	Side	Propensity score	Number of tranfusions	Matched
1003321	1	0.2209456	5	0
1003322	1	0.2473207	3	0
1003323	2	0.8684553	0	0
1003324	2	0.8151238	0	0
1003325	1	0.8923432	0	0
1003326	1	0.9796864	0	0
1003327	2	0.9718675	0	0
1003328	2	0.321832	1	1
1003329	2	0.810253	0	0
1003330	2	0.8380845	0	1
1003331	1	0.6743881	2	1
1003332	1	0.8590341	0	0
1003333	1	0.0936681	4	0
1003334	2	0.8464121	0	0
1003335	2	0.6822033	0	0
1003336	1	0.559449	2	1
1003337	1	0.8082396	1	1
1003338	1	0.7032168	2	1
1003339	2	0.8143077	0	1
1003340	2	0.9291345	0	0
1003341	1	0.8892304	0	0
1003342	2	0.3278905	0	1
1003343	1	0.9301074	0	0
1003344	1	0.3753152	4	1
1003345	1	0.7010806	0	0
1003346	2	0.6618387	3	1
1003347	2	0.1338009	2	0
1003348	1	0.8143077	2	1
1003348	2	0.8143077	0	0
1003349	1	0.9408087	0	0
1003350	1	0.9862315	0	0
1003351	1	0.3314213	6	1
1003352	1	0.5309048	0	1
1003353	1	0.6618387	0	0
1003354	1	0.7597614	0	0
1003355	2	0.0986374	13	0
1003356	2	0.4593293	2	1
1003357	1	0.8150505	0	0
1003358	1	0.837696	0	0
1003359	1	0.823929	0	0

Patient ID	Side	Propensity score	Number of tranfusions	Matched
1003360	2	0.4337504	0	1
1003360	1	0.2770845	0	1
1003361	2	0.7974147	2	1
1003362	2	0.9336253	0	0
1003363	1	0.5012139	0	1
1003364	2	0.5998099	2	1
1003364	1	0.7149794	0	0
1003365	1	0.1180535	10	1
1003366	2	0.0560851	2	1
1003367	1	0.234669	3	0
1003368	1	0.410127	3	1
1003369	1	0.5281954	0	1
1003370	2	0.6534875	0	0
1003371	2	0.9043188	0	1
1003372	2	0.9372068	0	0
1003373	1	0.8268883	0	0
1003374	2	0.5057742	2	1
1003375	2	0.522026	2	1
1003376	2	0.8464121	0	0
1003377	2	0.9550222	0	0
1003378	1	0.7059057	0	1
1003379	2	0.4524742	0	1
1003380	2	0.9471644	0	0
1003381	2	0.9527564	0	0
1003382	1	0.7299379	0	0
1003382	2	0.7699598	0	1
1003383	2	0.683496	2	1
1003384	2	0.2578129	2	0
1003385	2	0.2833378	5	0
1003386	1	0.6618387	0	0
1003387	1	0.6544533	0	1
1003388	2	0.6210378	0	1
1003388	1	0.7564785	0	1
1003389	2	0.5257468	0	1
1003390	2	0.8963693	0	0
1003391	1	0.9380204	0	0
1003392	2	0.1718138	2	0
1003393	1	0.6014247	2	1
1003393	2	0.5328374	0	1

Patient ID	Side	Propensity score	Number of tranfusions	Matched
1003394	1	0.1764928	5	0
1003395	2	0.714073	0	0
1003396	1	0.9240073	0	0
1003397	1	0.7637279	0	0
1003398	2	0.3893244	0	1
1003399	2	0.7731047	2	1
1003400	1	0.7370072	2	1
1003401	1	0.9482519	0	0
1003402	2	0.7628868	3	1
1003403	2	0.2595311	2	1
1003404	2	0.9395485	0	0
1003405	1	0.4044706	2	1
1003405	2	0.7010806	2	1
1003406	1	0.4044706	0	1
1003406	2	0.4044706	0	1
1003407	1	0.4415582	1	0
1003408	1	0.9290496	1	1
1003408	2	0.9149639	0	0
1003409	1	0.8836279	0	0
1003410	1	0.8189673	0	0
1003411	2	0.8380845	2	1
1003412	1	0.5038861	3	1
1003413	2	0.4395469	0	1
1003414	2	0.5152384	1	1
1003415	2	0.622981	2	1
1003416	2	0.6006756	0	1
1003417	2	0.5952844	0	1
1003418	2	0.2150401	0	1
1003419	2	0.9086713	0	0
1003420	1	0.4524742	2	1
1003420	2	0.6586223	0	0
1003421	1	0.8464121	0	1
1003422	1	0.8823384	0	1
1003423	2	0.3504041	1	0
1003423	1	0.3504041	2	0
1003424	2	0.3138182	5	0
1003425	1	0.7146748	1	1
1003426	1	0.449342	2	1
1003427	1	0.5745877	1	1

Patient ID	Side	Propensity score	Number of tranfusions	Matched
1003428	1	0.933683	0	0
1003429	1	0.8450363	0	1
1003430	1	0.1303028	13	0
1003431	1	0.714073	0	0
1003432	1	0.6210378	2	1
1003433	1	0.0751356	5	0
1003434	1	0.420392	4	0
1003435	2	0.8675035	0	0
1003436	1	0.4927622	0	1
1003437	1	0.5795027	1	1
1003438	2	0.9448927	0	0
1003439	2	0.6468291	2	1
1003440	1	0.2050429	2	1
1003441	2	0.9557838	0	0
1003442	2	0.8303496	0	0
1003443	1	0.7559605	4	1
1003444	2	0.4106748	0	1
1003445	1	0.0986374	3	0
1003446	1	0.467087	2	1
1003447	2	0.2830849	3	1
1003448	1	0.659002	0	0
1003449	1	0.3563927	3	1
1003450	2	0.9166842	0	0
1003450	1	0.8963693	0	0
1003451	2	0.2645873	1	0
1003452	1	0.4236214	0	1
1003453	2	0.7613885	0	1
1003454	2	0.1548002	3	0
1003455	1	0.8111391	0	0
1003455	2	0.8949718	0	0
1003456	2	0.3250186	2	0
1003457	1	0.6889592	0	0
1003458	2	0.3019094	12	0
1003459	2	0.6105611	6	1
1003460	2	0.9653663	0	0
1003461	2	0.924686	0	0
1003462	1	0.7834761	0	0
1003463	1	0.599822	2	1
1003464	1	0.8410779	0	0

Patient ID	Side	Propensity score	Number of tranfusions	Matched
1003465	2	0.6368605	0	1
1003466	2	0.467087	0	1
1003467	1	0.3028421	0	1
1003468	2	0.6174811	6	1
1003469	2	0.7317822	6	1
1003470	1	0.9829916	0	0
1003471	1	0.6538603	2	1
1003472	2	0.9350258	0	0
1003473	1	0.6292635	0	1
1003474	2	0.8234012	0	0
1003475	1	0.9128413	0	0
1003476	1	0.8303496	2	1
1003476	2	0.8303496	2	1
1003477	1	0.3451287	4	1
1003478	2	0.7837581	0	0
1003479	2	0.3436485	0	1
1003480	1	0.7797947	2	1
1003481	1	0.228877	2	0
1003482	1	0.9180547	0	0
1003483	2	0.9281372	1	1
1003484	2	0.2286018	2	0
1003485	2	0.473773	1	1
1003486	2	0.9380204	2	1
1003487	2	0.9569966	0	0
1003488	1	0.9371699	0	0
1003489	2	0.9371651	0	0
1003490	1	0.9289944	0	0
1003491	2	0.8143077	0	0
1003492	1	0.9086713	0	0
1003493	2	0.1990663	0	1
1003494	1	0.1445053	2	0
1003495	1	0.5211484	2	1
1003496	2	0.810253	0	0
1003497	1	0.6056686	0	1
1003498	1	0.2772692	2	0
1003499	2	0.693725	0	0
1003500	1	0.8722887	0	0
1003501	1	0.5904373	0	1
1003502	1	0.8926764	0	0
1003503	2	0.2271049	2	1

Patient ID	Side	Propensity score	Number of tranfusions	Matched
1003504	1	0.1908818	12	0
1003505	2	0.833655	0	1
1003506	1	0.4791337	0	1
1003507	2	0.2431445	0	1
1003507	1	0.4942396	0	1
1003508	1	0.4044706	0	1
1003509	2	0.0621982	9	0
1003510	1	0.1799348	5	1
1003511	2	0.9134939	0	0
1003512	1	0.2637527	0	1
1003513	1	0.924686	0	0
1003514	1	0.7281684	2	1
1003515	2	0.2402883	0	1
1003516	2	0.6349469	0	0
1003517	2	0.0525045	7	0
1003518	2	0.8583781	0	0
1003519	1	0.5747471	2	1
1003520	1	0.8926411	0	0
1003521	1	0.7838778	0	1
1003522	2	0.755473	0	0
1003523	1	0.1548002	1	0
1003524	1	0.467087	0	1
1003525	1	0.4817617	3	1
1003526	1	0.9180171	0	0
1003527	2	0.2429393	1	0
1003528	2	0.0734251	2	0
1003529	1	0.3711177	1	0
1003530	1	0.4375402	2	1
1003531	2	0.321832	0	1
1003532	1	0.1525709	2	0
1003532	2	0.3612392	0	1
1003533	2	0.6743881	0	0
1003534	2	0.3498713	7	0
1003535	1	0.2797864	0	1
1003536	2	0.8111391	0	0
1003537	1	0.882295	0	0
1003538	2	0.9457731	0	0
1003539	2	0.1896968	0	1
1003539	1	0.1896968	2	1

Patient ID	Side	Propensity score	Number of tranfusions	Matched
1003540	1	0.9568595	0	1
1003541	1	0.5516044	3	1
1003542	1	0.678968	0	0
1003543	2	0.321832	0	1
1003544	1	0.7759587	0	0
1003545	2	0.9499006	0	0
1003546	2	0.9694243	0	0
1003547	2	0.3666436	0	1
1003548	1	0.846667	1	1
1003549	2	0.3474476	2	0
1003550	2	0.9557483	0	0
1003551	2	0.2576619	2	0
1003552	1	0.5057742	0	1
1003552	2	0.8724139	0	0
1003553	2	0.41621	2	1
1003554	1	0.9775355	0	0
1003555	2	0.9398549	0	0
1003556	1	0.6723223	2	1
1003557	1	0.9568595	0	0
1003558	2	0.8648404	0	0
1003559	1	0.7743031	0	0
1003560	2	0.5546064	3	1
1003561	1	0.9166842	0	0
1003562	1	0.918583	0	0
1003563	2	0.8307439	0	0
1003564	1	0.2488727	1	0
1003565	2	0.846667	0	1
1003565	1	0.7838778	2	1
1003566	2	0.9468601	0	0
1003567	1	0.1912525	2	0
1003568	2	0.7858006	1	1
1003569	2	0.3531793	0	1
1003570	2	0.0884485	4	1
1003571	1	0.9684105	0	0
1003572	1	0.2624929	1	0
1003573	2	0.7656533	0	1
1003574	1	0.8271991	0	0
1003575	2	0.683496	1	1
1003576	2	0.882295	0	0

Patient ID	Side	Propensity score	Number of tranfusions	Matched
1003577	1	0.7610734	0	0
1003578	2	0.9402535	0	0
1003579	1	0.6544533	2	1
1003580	2	0.4303197	2	0
1003581	1	0.6892153	0	0
1003582	1	0.8838318	0	0
1003583	1	0.1527036	7	0
1003584	1	0.6565705	2	1
1003584	2	0.6506863	0	0
1003585	1	0.947474	0	0
1003586	2	0.4878789	0	1
1003587	2	0.9163947	0	0
1003588	1	0.926904	2	1
1003589	1	0.9197193	2	1
1003590	1	0.7312732	2	1
1003591	2	0.3116332	4	0
1003592	2	0.1548002	1	0
1003593	2	0.5025039	2	1
1003594	2	0.8958698	0	0
1003595	1	0.8003601	7	1
1003596	1	0.8984905	0	0
1003597	2	0.9364763	0	1
1003598	2	0.6339748	1	1
1003599	1	0.7299379	0	1
1003600	1	0.7429692	0	0
1003601	2	0.721094	2	1
1003602	1	0.3007151	7	1
1003603	1	0.5166902	2	1
1003604	2	0.1512063	5	0
1003605	1	0.9366489	0	0
1003606	1	0.7010806	0	0
1003607	1	0.6506863	2	1
1003608	1	0.8190085	0	0
1003609	1	0.9243086	0	0
1003609	2	0.9637995	0	0
1003610	2	0.5623317	3	1
1003611	2	0.9145093	0	0
1003612	1	0.5542496	0	1
1003613	1	0.8835036	0	0

Patient ID	Side	Propensity score	Number of tranfusions	Matched
1003614	1	0.9258477	0	0
1003615	2	0.3650076	0	1
1003616	1	0.7862898	0	0
1003617	2	0.2511445	0	1
1003618	1	0.3115666	2	1
1003619	1	0.7663047	0	0
1003620	2	0.8531178	0	0
1003621	2	0.8306983	0	1
1003622	1	0.9684105	0	0
1003623	1	0.9481907	0	0
1003624	1	0.112128	6	1
1003624	2	0.56959	3	1
1003625	1	0.9395485	0	1
1003626	2	0.5713235	2	1
1003627	2	0.4491468	3	1
1003628	1	0.1641638	4	0
1003629	2	0.7838778	0	0
1003630	2	0.9568595	0	0
1003631	2	0.4722031	1	1
1003632	2	0.8583781	1	1
1003633	1	0.7348097	0	0
1003634	1	0.3504041	3	0
1003635	1	0.2775909	6	0
1003636	1	0.6343521	0	1
1003636	2	0.9448927	0	0
1003637	1	0.9024512	0	0
1003637	2	0.9557483	0	0
1003638	1	0.9380204	0	0
1003639	2	0.8923432	1	1
1003640	1	0.5824782	0	0
1003641	2	0.9366489	0	0
1003642	2	0.2111481	2	0
1003643	1	0.1659664	8	1
1003644	1	0.8143077	0	0
1003645	2	0.4272192	2	0
1003646	2	0.4353576	3	1
1003647	2	0.9623802	0	0
1003648	1	0.8652821	0	0
1003649	1	0.8979311	0	0

Patient ID	Side	Propensity score	Number of tranfusions	Matched
1003650	2	0.9505225	0	0
1003651	1	0.4545422	0	1
1003652	1	0.7010806	0	0
1003653	1	0.9269953	0	0
1003654	2	0.473773	2	1
1003655	1	0.8881328	0	0
1003656	2	0.7838286	0	0
1003657	1	0.9119742	0	0
1003658	2	0.8380845	0	0
1003659	1	0.9467011	0	0
1003659	2	0.9398549	0	0
1003660	2	0.7861992	2	1
1003661	1	0.9222556	0	0
1003662	1	0.0948138	2	0
1003663	1	0.6370063	0	1
1003664	2	0.8963693	0	0
1003665	2	0.5490619	2	1
1003666	2	0.0372303	4	0
1003667	2	0.7089199	2	1
1003668	1	0.4006762	0	1
1003669	2	0.4224298	2	0
1003670	2	0.2198282	6	0
1003671	1	0.9353824	0	1
1003672	2	0.7145358	0	0
1003673	2	0.6517544	0	0
1003674	2	0.4455333	0	1
1003674	1	0.8348097	2	1
1003675	2	0.5081023	2	1
1003676	2	0.898414	0	0
1003677	1	0.9684105	0	0
1003678	2	0.9775355	0	0
1003678	1	0.9775355	0	0
1003679	1	0.3592359	3	1
1003680	1	0.683496	0	1
1003681	2	0.3326873	0	1
1003682	2	0.2967845	2	1
1003683	2	0.4144451	0	1
1003683	1	0.6124737	3	1
1003684	2	0.9145093	0	0

Patient ID	Side	Propensity score	Number of tranfusions	Matched
1003685	2	0.5641194	0	1
1003686	1	0.7175789	2	1
1003687	1	0.5307699	0	1
1003688	1	0.882295	0	0
1003689	1	0.6339748	4	1
1003690	2	0.5025039	0	1
1003691	2	0.5478821	0	1
1003692	1	0.7911524	0	0
1003693	1	0.9732879	0	0
1003694	2	0.3871764	0	1
1003695	2	0.3966366	0	1
1003696	2	0.9584746	0	0
1003697	1	0.3504041	2	0
1003698	1	0.9637995	0	0
1003699	2	0.2055755	2	0
1003699	1	0.3528668	4	0
1003700	1	0.9682459	0	0
1003701	2	0.9313657	0	0
1003701	1	0.9775355	0	0
1003702	1	0.7526756	2	1
1003703	1	0.7803655	0	0
1003704	2	0.7808636	0	0
1003705	2	0.8244078	0	0
1003705	1	0.6074143	0	1
1003706	1	0.6517544	0	0
1003707	1	0.6743881	0	1
1003708	1	0.2858255	1	1
1003709	2	0.6925109	0	1
1003709	1	0.790189	0	0
1003710	2	0.9527564	0	0
1003711	2	0.1760836	3	0
1003712	2	0.8187	0	0
1003713	2	0.9555191	0	0
1003714	2	0.1859022	2	0
1003715	1	0.8187	0	0
1003715	2	0.7571858	2	1
1003716	2	0.8632497	0	0
1003717	2	0.3122272	3	0
1003718	1	0.2344216	3	0

Patient ID	Side	Propensity score	Number of tranfusions	Matched
1003719	1	0.6368605	2	1
1003720	2	0.8003601	0	0
1003721	1	0.473773	0	1
1003722	2	0.9653663	0	0
1003723	1	0.9145093	0	0
1003724	1	0.5041998	3	1
1003725	1	0.1050111	6	0
1003726	1	0.9296347	0	0
1003727	1	0.9197193	0	0
1003728	2	0.9684105	0	0
1003729	2	0.561905	3	1
1003730	1	0.6201626	0	1
1003731	2	0.5621231	1	1
1003732	1	0.5679204	2	1
1003732	2	0.5679204	0	1
1003733	1	0.683496	0	1
1003734	1	0.4223626	0	1
1003735	2	0.8357238	0	0
1003736	2	0.2024851	5	0
1003737	1	0.9119742	0	0
1003738	2	0.7941505	0	0
1003739	2	0.2599114	4	0
1003740	1	0.437895	2	0
1003741	1	0.2024319	2	0
1003742	1	0.8793763	0	1
1003743	1	0.898414	0	0
1003744	2	0.9365586	0	0
1003745	1	0.0228245	4	0
1003746	1	0.8888902	0	0
1003747	1	0.8453464	0	0
1003747	2	0.8180251	6	1
1003748	2	0.4378303	0	1
1003749	1	0.9277478	0	0
1003749	2	0.9603292	0	0
1003750	1	0.9380204	0	0
1003751	2	0.9366737	0	0
1003751	1	0.9684105	0	0
1003752	2	0.4516916	0	1
1003753	2	0.9684105	0	0
1003754	2	0.5396611	0	1

Patient ID	Side	Propensity score	Number of tranfusions	Matched
1003755	1	0.9623802	0	0
1003756	2	0.56959	2	1
1003757	2	0.8531178	0	0
1003758	1	0.6305341	2	1
1003759	1	0.8851865	0	0
1003760	2	0.551494	2	1
1003761	2	0.9603292	0	0
1003762	2	0.7089199	2	1
1003763	2	0.7527626	0	0
1003764	2	0.5858861	0	1
1003765	2	0.346694	3	0
1003766	1	0.8589752	0	0
1003767	2	0.9603292	0	0
1003768	2	0.8464121	0	0
1003769	1	0.9471644	0	0
1003770	1	0.407293	0	1
1003771	2	0.5716151	0	1
1003772	1	0.7913258	0	0
1003773	2	0.7992524	0	1
1003774	2	0.8720679	0	0
1003775	2	0.0704406	9	0
1003776	2	0.779352	0	0
1003777	1	0.6449332	0	0
1003778	2	0.765845	0	0
1003779	1	0.1616691	4	0
1003780	1	0.8199402	0	1
1003781	1	0.5578053	0	1
1003782	1	0.2427046	2	0
1003783	1	0.9207713	0	0
1003784	2	0.7785966	0	1
1003785	2	0.9330271	0	0
1003786	1	0.7531048	0	0
1003787	1	0.0275699	5	0
1003788	2	0.4633183	0	1
1003789	1	0.9211168	0	0
1003790	2	0.4518385	1	1
1003790	1	0.5797566	0	1
1003791	1	0.563008	0	0
1003791	2	0.2666018	0	1

Patient ID	Side	Propensity score	Number of tranfusions	Matched
1003792	2	0.5575969	0	1
1003792	1	0.4006762	0	1
1003793	2	0.8720679	0	1
1003794	1	0.7819304	0	0
1003794	2	0.9639877	0	0
1003795	2	0.9395485	0	0
1003796	1	0.156403	1	0
1003797	2	0.7925557	3	1
1003798	2	0.6876798	0	1
1003799	1	0.517856	0	1
1003800	1	0.678968	2	1
1003801	2	0.833655	0	0
1003802	2	0.7004812	0	0
1003803	2	0.8626183	0	1
1003804	1	0.7867695	0	1
1003805	2	0.7451044	2	1
1003806	2	0.1773797	1	0
1003807	1	0.3305501	2	1
1003808	1	0.7511338	0	0
1003809	1	0.3977177	2	1
1003810	2	0.8464121	0	0
1003811	1	0.9766817	0	1
1003812	1	0.9532798	0	0
1003813	1	0.9197193	0	0
1003814	1	0.7265486	0	1
1003815	1	0.4774967	1	1
1003816	1	0.2525808	2	1
1003817	2	0.7317822	2	1
1003818	2	0.693725	0	1
1003819	1	0.4597755	0	1
1003820	1	0.8909839	0	0
1003821	1	0.0516379	2	0
1003822	1	0.5482672	4	1
1003823	1	0.8974619	0	0
1003824	2	0.88063	0	0
1003825	2	0.9605683	0	0
1003826	1	0.865247	0	0
1003827	1	0.6637512	0	1
1003828	1	0.9128584	0	0
1003829	2	0.4502311	2	1

Patient ID	Side	Propensity score	Number of tranfusions	Matched
1003829	1	0.4254257	0	1
1003830	1	0.321832	4	0
1003831	2	0.9288847	0	0
1003832	1	0.8520059	0	0
1003833	1	0.9482519	0	0
1003834	1	0.5372857	0	1
1003835	1	0.8987589	0	0
1003836	2	0.506164	0	1
1003837	2	0.9095091	0	0
1003838	1	0.8380845	0	0
1003839	2	0.3774182	0	1
1003840	2	0.0476974	2	0
1003841	2	0.0878338	7	0
1003842	1	0.6265182	2	1
1003843	2	0.204323	3	0
1003844	1	0.5201238	2	1
1003845	1	0.0273303	2	0
1003846	1	0.678968	0	0
1003847	2	0.3048106	2	1
1003848	1	0.7143803	0	0
1003849	2	0.8347511	0	1
1003850	2	0.9211168	0	0
1003851	1	0.5405086	2	1
1003851	2	0.7877594	4	1
1003852	1	0.651317	1	1
1003853	2	0.9389041	0	0
1003854	1	0.9395485	0	0
1003855	1	0.7861992	0	0
1003856	2	0.9512951	0	0
1003857	2	0.5211484	0	1
1003858	1	0.4059943	0	1
1003859	1	0.915615	0	1
1003860	2	0.3399404	2	0
1003861	2	0.2000821	2	0
1003862	1	0.6288589	0	1
1003863	1	0.9684105	0	0
1003863	2	0.9684105	0	0
1003864	2	0.2710625	3	0
1003865	2	0.8838318	0	0
1003866	2	0.7956537	0	0

Patient ID	Side	Propensity score	Number of tranfusions	Matched
1003867	2	0.8719391	0	0
1003868	2	0.6084539	0	0
1003869	2	0.872855	0	0
1003870	1	0.7374049	2	1
1003871	1	0.3504041	4	0
1003871	2	0.4733407	4	1
1003872	2	0.2992102	2	1
1003873	1	0.3356613	2	1
1003873	2	0.380564	5	0
1003874	2	0.5685832	1	1
1003875	2	0.8508557	2	1
1003876	1	0.9197193	0	0
1003877	1	0.7199393	0	1
1003878	1	0.9718675	0	0
1003879	1	0.5307699	0	1
1003880	2	0.4752627	0	1
1003881	1	0.0438309	3	0
1003882	2	0.1698545	0	1
1003882	1	0.2550914	0	1
1003883	2	0.4109022	0	1
1003884	2	0.6174811	2	1
1003885	1	0.3935633	4	0
1003886	2	0.8987589	0	1
1003886	1	0.8987589	0	0
1003887	1	0.7468932	0	0
1003888	1	0.0147431	0	1
1003889	2	0.3322061	0	1
1003890	2	0.5312783	3	1
1003890	1	0.1506232	3	0
1003891	2	0.38985	4	0
1003892	1	0.3774182	0	1
1003893	1	0.9039453	0	0
1003894	1	0.1359229	2	0
1003895	1	0.6405191	0	1
1003895	2	0.5102741	0	1
1003896	1	0.8565127	1	1
1003897	2	0.8770538	0	1
1003898	1	0.833655	0	0
1003899	2	0.9796864	0	0
1003900	1	0.8267231	0	0

Patient ID	Side	Propensity score	Number of tranfusions	Matched
1003901	2	0.678968	0	1
1003902	2	0.4055494	3	1
1003903	2	0.6014652	2	1
1003904	1	0.6723223	0	1
1003905	2	0.7004812	3	1
1003906	2	0.4221659	3	1
1003907	1	0.87418	0	0
1003908	1	0.8101415	0	0
1003909	2	0.3749073	0	1
1003910	2	0.8380845	0	0
1003911	2	0.8199402	0	0
1003912	1	0.9382355	0	0
1003913	1	0.3250186	2	0
1003914	1	0.5826166	1	1
1003915	1	0.3932348	3	0
1003916	1	0.8982696	0	0
1003917	1	0.7515005	0	0
1003917	2	0.7809963	0	0
1003918	2	0.3711177	2	1
1003919	1	0.622981	0	1
1003920	1	0.5403834	3	1
1003921	2	0.158659	2	0
1003922	2	0.2821948	2	1
1003923	1	0.4638417	3	1
1003924	1	0.6500681	0	0
1003924	2	0.8380845	0	0
1003925	1	0.8652821	0	0
1003926	2	0.9145093	0	0
1003927	2	0.9366489	0	0
1003928	2	0.9086713	0	0
1003929	1	0.9743555	0	0
1003929	2	0.9862315	0	0
1003930	1	0.9278036	0	0
1003931	2	0.8595092	0	0
1003932	1	0.7445363	0	0
1003933	1	0.7515005	0	0
1003934	1	0.7776973	1	1
1003935	2	0.080481	8	0
1003936	1	0.3504041	4	0

Patient ID	Side	Propensity score	Number of tranfusions	Matched
1003937	2	0.0795248	2	0
1003938	2	0.9343481	0	0
1003939	2	0.8375368	2	1
1003940	1	0.8143077	3	1
1003941	1	0.4921493	2	1
1003942	2	0.8064643	0	0
1003942	1	0.8064643	0	0
1003943	1	0.511364	2	1
1003944	1	0.6321596	2	1
1003945	1	0.1495744	2	0
1003946	1	0.2860811	0	1
1003947	1	0.4209728	1	1
1003948	1	0.8746868	2	1
1003949	1	0.0754001	2	0
1003950	1	0.8823384	0	1
1003951	2	0.9206523	0	0
1003952	1	0.7797947	0	1
1003953	1	0.893461	0	0
1003954	2	0.819938	0	1
1003955	1	0.5598815	2	1
1003956	1	0.4104241	0	1
1003957	2	0.0280013	4	0
1003958	1	0.1754709	4	0
1003959	1	0.8983983	2	1
1003960	1	0.8043698	2	1
1003961	2	0.0256407	4	0
1003962	2	0.3504041	2	0
1003963	2	0.5218534	0	1
1003964	2	0.805438	0	1
1003965	2	0.9743555	0	0
1003966	2	0.56959	0	1
1003967	1	0.8722887	0	0
1003968	1	0.473773	2	1
1003968	2	0.3250186	4	1
1003969	2	0.1859022	2	0
1003970	1	0.1548002	4	0
1003971	2	0.9684105	0	0
1003972	2	0.5085712	2	1
1003973	2	0.9408087	0	0

Patient ID	Side	Propensity score	Number of tranfusions	Matched
1003974	1	0.5147179	0	1
1003975	1	0.7577799	0	0
1003976	1	0.3250186	2	0
1003977	2	0.3028421	0	1
1003978	1	0.5186767	2	1
1003979	2	0.1320813	6	0
1003980	1	0.1970638	1	0
1003981	2	0.7597614	0	0
1003982	2	0.6185063	0	1
1003983	1	0.7374109	0	0
1003984	1	0.8349239	0	0
1003985	2	0.8840578	0	0
1003986	2	0.0504265	1	0
1003987	2	0.4519534	1	1
1003988	1	0.473773	2	1
1003989	2	0.8143077	0	0
1003990	1	0.8186172	0	0
1003991	1	0.8887281	0	1
1003992	2	0.5557157	0	1
1003993	2	0.6370454	0	0
1003994	1	0.4676726	0	1
1003995	2	0.8143077	0	0
1003996	1	0.8757317	2	1
1003997	1	0.599822	0	1
1003998	2	0.8744204	0	1
1003999	2	0.7440412	0	0
1004000	2	0.9611736	0	0
1004001	1	0.9555191	0	0
1004002	2	0.5737743	1	1
1004003	1	0.8199402	0	0
1004004	2	0.9493857	0	0
1004005	1	0.9009214	0	0
1004006	1	0.8652123	0	0
1004007	1	0.7471596	0	0
1004007	2	0.7471596	0	0
1004008	2	0.4906955	1	1
1004009	1	0.7602424	0	0
1004010	2	0.693725	0	0
1004011	1	0.473773	2	1
1004012	2	0.7284777	0	0

Patient ID	Side	Propensity score	Number of tranfusions	Matched
1004013	1	0.5057742	2	1
1004014	2	0.0963621	8	0
1004015	1	0.941524	0	0
1004016	2	0.9557483	0	0
1004017	1	0.9342051	1	1
1004018	1	0.6271352	3	1
1004019	1	0.2117264	4	1
1004020	2	0.407293	3	1
1004021	2	0.7858006	0	1
1004021	1	0.941524	0	0
1004022	2	0.1635913	1	0
1004023	1	0.3028421	0	1
1004024	2	0.9471208	0	0
1004025	1	0.9482724	0	0
1004026	1	0.8303496	0	0
1004027	2	0.9454951	0	0
1004028	2	0.8583781	0	0
1004029	1	0.7010806	0	0
1004030	1	0.9366489	0	0
1004031	1	0.58239	0	0
1004032	2	0.8976958	0	0
1004033	2	0.3968271	0	1
1004034	2	0.4033335	2	1
1004035	2	0.8004348	1	1
1004036	2	0.9119742	0	1
1004037	2	0.6071733	1	1
1004038	2	0.7511338	0	0
1004039	1	0.7999699	1	1
1004040	2	0.8021703	0	0
1004041	2	0.4733407	1	1
1004041	1	0.2639768	2	0
1004042	2	0.9145093	0	0
1004043	1	0.0262742	18	0
1004044	2	0.7795189	0	0
1004045	1	0.3395415	0	1
1004046	1	0.454003	0	1
1004047	1	0.6565705	0	1
1004048	2	0.4820497	4	1
1004049	2	0.1698545	2	0
1004050	2	0.0331062	5	0

Patient ID	Side	Propensity score	Number of tranfusions	Matched
1004051	1	0.8684553	0	0
1004052	1	0.5050592	2	1
1004053	2	0.3648859	2	1
1004054	2	0.6860728	3	1
1004055	2	0.9637995	0	0
1004056	2	0.881481	0	0
1004057	1	0.8420542	0	1
1004058	2	0.9041181	0	0
1004059	1	0.507064	2	1
1004060	2	0.8923432	2	1
1004061	1	0.9135338	0	0
1004062	1	0.9743555	0	0
1004063	1	0.8968255	0	0
1004064	1	0.6909467	4	1
1004065	1	0.5740564	0	0
1004066	1	0.7175789	2	1
1004067	1	0.818326	0	0
1004068	1	0.8920939	0	0
1004068	2	0.8920939	0	0
1004069	2	0.8199402	0	0
1004070	1	0.965012	0	0
1004071	2	0.7613885	0	1
1004072	2	0.2808335	2	0
1004073	1	0.6889592	0	1
1004074	1	0.1015416	0	1
1004075	1	0.898414	0	0
1004076	2	0.5261353	0	1
1004077	1	0.8033172	0	1
1004077	2	0.9646522	0	0
1004078	1	0.7613885	2	1
1004079	1	0.838091	0	0
1004080	2	0.8719391	0	1
1004080	1	0.8719391	0	1
1004081	1	0.9454951	0	0
1004082	2	0.473773	1	1
1004083	1	0.9547941	0	0
1004084	1	0.0437246	5	0
1004085	1	0.7949521	0	0
1004085	2	0.8071553	0	0

Patient ID	Side	Propensity score	Number of tranfusions	Matched
1004086	2	0.8772783	0	0
1004087	1	0.79514	0	1
1004088	2	0.907451	1	1
1004089	1	0.7885835	4	1
1004090	1	0.6341578	0	0
1004091	1	0.6805989	0	0
1004092	2	0.6618387	0	1
1004093	2	0.8583781	0	0
1004094	2	0.9082494	0	0
1004095	1	0.0075015	7	0
1004096	2	0.7436793	0	0
1004096	1	0.9163947	0	0
1004097	1	0.2204541	1	0
1004098	2	0.8143077	0	0
1004099	2	0.8622236	0	0
1004100	2	0.9555191	0	0
1004101	1	0.378616	1	1
1004102	1	0.248998	2	0
1004102	2	0.248998	0	1
1004103	2	0.4859977	0	1
1004104	1	0.9151533	0	0
1004105	2	0.954536	0	0
1004106	2	0.9186629	0	0
1004107	1	0.4006762	2	1
1004108	2	0.9557483	0	0
1004109	2	0.5411458	0	1
1004110	2	0.9163947	0	0
1004111	2	0.2799763	0	1
1004112	1	0.7624756	0	0
1004113	1	0.8651406	0	0
1004114	1	0.1901215	8	0
1004115	1	0.9342051	0	0
1004116	1	0.7955295	0	0
1004116	2	0.9607372	0	1
1004117	1	0.7663047	2	1
1004118	1	0.701194	0	0
1004119	1	0.918583	0	1
1004120	1	0.6498169	2	1
1004121	2	0.7357013	0	0

Patient ID	Side	Propensity score	Number of tranfusions	Matched
1004122	1	0.5853952	1	1
1004122	2	0.1105052	2	0
1004123	1	0.8192388	0	0
1004124	1	0.980466	0	0
1004125	1	0.8282737	0	0
1004126	1	0.4796653	0	1
1004127	1	0.6743881	0	1
1004128	1	0.5308087	0	1
1004128	2	0.7484937	2	1
1004129	2	0.4995343	0	1
1004130	1	0.9481907	0	0
1004131	2	0.0532364	7	0
1004132	1	0.7097606	0	1
1004133	1	0.9150608	0	0
1004134	2	0.467087	2	1
1004135	2	0.5056041	2	1
1004136	1	0.608248	0	0
1004137	1	0.9408087	6	1
1004138	1	0.7010806	0	0
1004139	1	0.0942148	3	0
1004140	2	0.9684105	0	0
1004141	2	0.9024512	0	0
1004142	2	0.4174627	3	1
1004143	2	0.1024967	5	0
1004144	2	0.32247	2	0
1004145	2	0.5975077	2	1
1004146	1	0.9471623	0	0
1004147	1	0.410127	3	1
1004148	2	0.7451044	0	0
1004149	1	0.9684105	0	0
1004150	2	0.9557838	0	0
1004151	2	0.6182663	0	1
1004152	1	0.599822	0	1
1004153	2	0.97214	0	0
1004154	2	0.6888522	0	0
1004155	1	0.8771753	0	0
1004156	2	0.1748169	4	0
1004157	2	0.7294804	0	1
1004158	2	0.0538607	9	0
1004159	1	0.2994416	1	0

Patient ID	Side	Propensity score	Number of tranfusions	Matched
1004160	1	0.1645321	6	0
1004161	2	0.8159437	5	1
1004162	2	0.9145093	0	0
1004163	2	0.8892304	0	1
1004164	1	0.6618387	0	0
1004165	1	0.810253	0	0
1004166	2	0.9454951	2	1
1004167	1	0.7143803	0	0
1004168	2	0.4440603	0	1
1004169	2	0.9607372	0	0
1004170	1	0.7204169	0	1
1004171	1	0.3657055	3	1
1004172	1	0.5099006	2	1
1004173	1	0.6092499	0	1
1004174	2	0.6872183	2	1
1004175	1	0.7861992	0	1
1004176	1	0.931036	0	0
1004177	2	0.5623317	0	1
1004178	2	0.9684361	0	0
1004179	2	0.2404424	0	1
1004180	2	0.3464321	4	1
1004180	1	0.4998259	2	1
1004181	1	0.41679	4	1
1004182	2	0.2171266	1	0
1004183	2	0.3308833	2	1
1004184	2	0.7624756	0	0
1004185	1	0.7994878	4	1
1004186	1	0.1460613	2	0
1004187	2	0.4515096	2	1
1004188	2	0.9568595	0	0
1004189	1	0.8838318	0	0
1004190	2	0.8531178	0	0
1004191	1	0.8349296	0	0
1004192	2	0.2992102	2	1
1004193	2	0.9145093	0	0
1004194	2	0.5359595	0	1
1004195	1	0.8585083	0	0
1004196	2	0.3504041	3	0
1004197	2	0.5085712	4	1

Patient ID	Side	Propensity score	Number of tranfusions	Matched
1004198	1	0.346036	0	1
1004199	1	0.9555191	0	0
1004200	2	0.6093747	0	0
1004200	1	0.4575979	2	1
1004201	1	0.3442346	4	1
1004202	1	0.9121083	0	0
1004203	2	0.2992102	0	1
1004204	2	0.5740564	2	1
1004205	1	0.56959	0	1
1004206	1	0.6339748	0	0
1004207	2	0.7451044	0	1
1004208	1	0.9207713	0	0
1004209	1	0.9827961	0	0
1004210	1	0.8622236	0	0
1004211	2	0.5593951	3	1
1004212	1	0.3961032	0	1
1004213	2	0.9639877	0	0
1004214	1	0.8905035	0	0
1004215	2	0.7993932	0	0
1004216	1	0.9145093	0	0
1004217	1	0.527902	2	1
1004217	2	0.8678662	0	0
1004218	1	0.8733999	1	1
1004219	2	0.7317822	5	1
1004220	1	0.5002404	0	1
1004221	1	0.7838778	1	1
1004222	1	0.2992102	4	1
1004223	1	0.9656597	0	0
1004224	2	0.6921092	0	0
1004225	1	0.793514	3	1
1004226	2	0.8604233	0	0
1004227	2	0.8930209	0	0
1004228	1	0.9119742	0	0
1004229	1	0.7216224	2	1
1004230	1	0.7717639	0	1
1004231	1	0.6103809	0	1
1004231	2	0.6103809	2	1
1004232	2	0.1635913	2	0
1004233	2	0.9718675	0	0
1004234	2	0.7346398	0	0

Patient ID	Side	Propensity score	Number of tranfusions	Matched
1004235	1	0.9659986	0	0
1004236	2	0.7159442	0	0
1004237	1	0.3711177	2	1
1004238	2	0.6166569	2	1
1004239	2	0.9603292	0	0
1004239	1	0.7836873	0	0
1004240	2	0.9684361	0	0
1004241	1	0.843697	0	1
1004242	2	0.6103809	2	1
1004243	1	0.4414355	0	1
1004244	1	0.9555547	0	1
1004245	2	0.7717639	0	0
1004246	1	0.1933552	1	0
1004247	1	0.2828303	0	1
1004248	2	0.678968	0	1
1004249	1	0.9256307	0	0
1004249	2	0.9000514	2	1
1004250	2	0.0453375	1	0
1004251	1	0.6129037	3	1
1004252	1	0.0630246	10	0
1004253	1	0.8744045	6	1
1004254	2	0.9675201	0	0
1004255	1	0.620867	0	1
1004256	2	0.7788128	0	0
1004257	2	0.3311657	0	1
1004258	2	0.5099006	2	1
1004259	1	0.9395485	0	0
1004260	2	0.0619076	11	0
1004261	1	0.9557838	0	0
1004262	1	0.810253	0	0
1004262	2	0.810253	0	0
1004263	2	0.0742841	2	0
1004264	2	0.1867323	7	0
1004265	2	0.4393737	0	1
1004266	1	0.5843855	0	0
1004267	1	0.3468367	4	1
1004268	1	0.8626183	0	0
1004269	2	0.2657531	4	1
1004269	1	0.2657531	0	1

Patient ID	Side	Propensity score	Number of tranfusions	Matched
1004270	2	0.5165051	3	1
1004270	1	0.5165051	2	1
1004271	1	0.9366489	0	0
1004272	2	0.6083256	0	0
1004273	2	0.9512951	0	0
1004274	1	0.9684105	0	0
1004275	2	0.6500681	0	0
1004275	1	0.693147	0	0
1004276	2	0.7053579	1	1
1004277	2	0.8892304	0	0
1004278	2	0.7399099	0	1
1004279	1	0.5863623	0	1
1004280	2	0.0242407	2	0
1004281	2	0.7317822	0	1
1004282	1	0.5211484	2	1
1004283	1	0.8783149	2	1
1004284	2	0.8450363	0	0
1004285	2	0.8869002	0	0
1004286	2	0.2807186	3	0
1004287	1	0.9730576	0	0
1004288	2	0.6271352	0	1
1004288	1	0.6210378	0	0
1004289	1	0.6014247	0	1
1004290	2	0.9366737	0	0
1004291	2	0.0304421	9	0
1004292	1	0.5290465	0	1
1004293	1	0.7240763	0	0
1004294	2	0.9348254	0	0
1004294	1	0.8838318	0	0
1004295	1	0.6299819	0	0
1004296	2	0.8720679	0	0
1004297	2	0.0973926	2	0
1004298	1	0.4254257	2	1
1004299	1	0.9458349	0	0
1004300	2	0.8871914	0	0
1004301	1	0.7039941	0	1
1004302	1	0.9482519	0	0
1004303	1	0.8380845	0	0
1004304	1	0.9653663	0	0
1004305	1	0.8590341	0	1

Patient ID	Side	Propensity score	Number of tranfusions	Matched
1004306	1	0.56959	0	1
1004307	2	0.0517778	2	0
1004308	2	0.9557483	0	0
1004309	1	0.3305501	2	1
1004310	2	0.9684105	0	0
1004310	1	0.9684105	0	0
1004311	2	0.8690648	2	1
1004312	1	0.8467754	2	1
1004313	2	0.7373435	0	0
1004314	2	0.9775355	0	0
1004315	2	0.9558169	0	0
1004316	1	0.3236385	4	1
1004317	1	0.8719391	2	1
1004317	2	0.6242512	0	1
1004318	2	0.4047476	1	1
1004319	1	0.7565187	0	1
1004320	2	0.6822033	0	1
1004321	2	0.9481907	0	0
1004322	2	0.6210378	1	1
1004323	1	0.9366489	0	0
1004324	1	0.6195415	1	1
1004325	1	0.9050807	0	0
1004326	1	0.8251629	0	0
1004327	2	0.1466258	11	0
1004328	2	0.8190085	0	0
1004329	1	0.1384197	8	0
1004330	1	0.9639877	0	0
1004331	1	0.6631602	0	0
1004332	1	0.6384172	0	0
1004333	2	0.8823384	4	1
1004334	2	0.3009225	0	1
1004335	1	0.9718675	0	0
1004336	1	0.8959568	0	0
1004337	1	0.8464121	2	1
1004338	1	0.0961444	3	0
1004339	1	0.8003601	0	0
1004340	2	0.6136546	9	1
1004341	2	0.9027108	1	1
1004342	2	0.9106365	0	0

Patient ID	Side	Propensity score	Number of tranfusions	Matched
1004343	1	0.5421411	0	1
1004344	1	0.6489229	2	1
1004345	1	0.9123386	0	0
1004346	2	0.865544	0	0
1004347	1	0.9555191	0	1
1004347	2	0.9555191	0	0
1004348	1	0.9348254	2	1
1004349	2	0.8591355	0	0
1004349	1	0.9402535	0	0
1004350	1	0.9623802	0	0
1004351	1	0.8652821	0	0
1004352	2	0.4359649	0	1
1004353	1	0.473773	0	1
1004354	2	0.4882489	0	1
1004355	1	0.3620975	4	0
1004355	2	0.2314156	2	0
1004356	1	0.6210378	0	1
1004357	1	0.9611736	0	0
1004358	1	0.9481907	0	0
1004359	2	0.547057	0	1
1004360	2	0.8722887	2	1
1004361	1	0.5740564	0	1
1004362	1	0.5797566	1	1
1004363	2	0.6179632	5	1
1004364	1	0.9555191	0	0
1004365	2	0.9827961	0	0
1004366	2	0.8143077	0	0
1004367	2	0.3282734	1	0
1004368	2	0.7751945	0	0
1004369	1	0.815375	0	0
1004370	2	0.3145485	1	0
1004371	2	0.9395485	0	1
1004372	1	0.9013558	0	0
1004373	1	0.8180251	0	0
1004374	2	0.7511399	0	1
1004375	1	0.6362959	3	1
1004376	2	0.9557483	0	0
1004377	2	0.7412045	1	1
1004378	2	0.6021064	0	1

Patient ID	Side	Propensity score	Number of tranfusions	Matched
1004379	1	0.9039453	0	0
1004380	2	0.6687954	0	1
1004381	1	0.6765335	0	1
1004382	2	0.9512951	0	0
1004383	1	0.9380204	0	0
1004384	2	0.8349239	0	1
1004385	1	0.5716151	6	1
1004386	2	0.755473	1	1
1004387	1	0.6586223	0	1
1004388	1	0.3602291	2	1
1004389	1	0.3006817	0	1
1004390	2	0.4912657	0	1
1004390	1	0.7660496	0	0
1004391	2	0.6370063	2	1
1004392	2	0.9512951	0	0
1004393	2	0.2550914	3	1
1004394	1	0.7892495	0	1
1004395	1	0.7613885	2	1
1004395	2	0.473773	2	1
1004396	1	0.3132114	4	1
1004397	2	0.8684553	0	0
1004398	1	0.7348097	0	0
1004399	1	0.9796864	0	0
1004399	2	0.9796864	4	1
1004400	2	0.9264762	0	0
1004401	1	0.9366489	0	0
1004402	1	0.8873877	0	0
1004403	2	0.3539359	0	1
1004404	1	0.6363631	2	1
1004404	2	0.767398	0	0
1004405	2	0.5623317	2	1
1004406	2	0.914542	0	0
1004406	1	0.8622236	0	0
1004407	1	0.7609577	0	0
1004408	2	0.9160318	0	0
1004409	1	0.2799763	2	1
1004410	1	0.9146145	0	0
1004411	2	0.6850716	1	1
1004412	2	0.9639877	2	1

Patient ID	Side	Propensity score	Number of tranfusions	Matched
1004413	1	0.1226307	6	1
1004414	1	0.8197724	0	0
1004415	1	0.5411458	0	1
1004416	2	0.0630246	2	0
1004417	1	0.5591499	2	1
1004418	2	0.8572502	0	0
1004419	2	0.8182057	0	0
1004420	2	0.467087	3	1
1004421	1	0.1048877	1	1
1004422	2	0.9471208	0	0
1004423	2	0.6210378	0	1
1004424	1	0.8176421	0	0
1004425	2	0.0341027	7	0
1004426	2	0.0370335	5	0
1004427	1	0.5487077	0	1
1004428	2	0.8199402	0	1
1004429	2	0.3798259	6	0
1004430	1	0.9364664	0	0
1004431	1	0.8923432	0	0
1004432	1	0.9395485	0	0
1004433	2	0.9546245	0	1
1004434	1	0.2126324	4	0
1004435	1	0.5450325	4	1
1004436	2	0.4270143	0	1
1004437	2	0.9256307	0	1
1004438	1	0.9222556	0	0
1004439	1	0.2992102	0	1
1004440	1	0.8997165	0	0
1004441	1	0.8380845	0	0
1004442	2	0.410127	0	1
1004443	1	0.8548512	2	1
1004444	1	0.9493857	0	0
1004445	2	0.9680528	0	0
1004446	2	0.8233393	0	1
1004447	1	0.8349239	6	1
1004448	2	0.7022124	0	0
1004449	1	0.2550914	2	0
1004450	2	0.9051527	0	0
1004451	2	0.2397506	9	1
1004452	1	0.7454769	0	0

Patient ID	Side	Propensity score	Number of tranfusions	Matched
1004453	1	0.9119742	0	0
1004454	1	0.5271277	2	1
1004455	1	0.1267193	4	0
1004456	2	0.9191106	0	0
1004457	2	0.5602688	2	1
1004458	2	0.5371487	1	1
1004458	1	0.3529911	4	0
1004459	1	0.5928773	0	1
1004460	1	0.599822	1	1
1004461	1	0.8143077	2	1
1004462	1	0.3065288	2	1
1004463	2	0.5743375	0	0
1004464	2	0.8254554	0	0
1004465	1	0.928416	0	0
1004466	2	0.2770792	3	1
1004467	2	0.7146748	0	1
1004468	2	0.15243	17	0
1004469	2	0.612785	0	1
1004469	1	0.7751945	0	0
1004470	1	0.85848	0	0
1004471	1	0.8305005	5	1
1004472	2	0.882295	0	0
1004473	1	0.8251629	0	0
1004474	1	0.750596	2	1
1004475	1	0.1859022	1	1
1004476	1	0.2473207	4	1
1004477	1	0.4900505	0	1
1004478	2	0.4337701	0	1
1004479	1	0.9862315	0	0
1004480	1	0.605145	2	1
1004481	2	0.9546245	0	0
1004482	1	0.1461907	2	0
1004483	2	0.834521	0	0
1004484	2	0.2550914	0	1
1004485	1	0.4653615	2	1
1004486	1	0.1582627	4	0
1004487	2	0.1055341	10	0
1004488	2	0.7671505	0	0
1004489	2	0.8448089	0	1

Patient ID	Side	Propensity score	Number of tranfusions	Matched
1004490	2	0.9684105	0	0
1004491	1	0.8393267	0	0
1004492	1	0.703726	0	0
1004493	2	0.5737743	0	0
1004494	2	0.5571656	0	1
1004495	1	0.8348297	0	1
1004496	2	0.7233331	0	0
1004497	2	0.755473	0	0
1004498	1	0.4631742	2	1
1004498	2	0.835932	0	0
1004499	1	0.4209728	0	1
1004500	1	0.9551294	0	0
1004501	2	0.7389681	2	1
1004502	1	0.6339748	2	1
1004503	1	0.1230849	2	0
1004504	2	0.5572492	0	1
1004505	1	0.8857348	0	1
1004506	1	0.9398549	0	0
1004507	1	0.9330049	0	0
1004508	2	0.6145392	0	1
1004509	1	0.6909467	4	1
1004510	1	0.6089534	0	1
1004510	2	0.6571131	1	1
1004511	1	0.2957018	2	1
1004512	2	0.5291469	0	1
1004513	1	0.5711182	0	1
1004514	2	0.9569966	0	0
1004515	2	0.8003601	0	1
1004516	2	0.2139115	2	0
1004517	1	0.7525656	0	0
1004518	1	0.8066371	0	0
1004519	1	0.8987589	0	0
1004520	1	0.2236856	0	1
1004521	1	0.7266501	0	1
1004521	2	0.833655	0	1
1004522	2	0.3305501	0	1
1004523	2	0.9419123	0	0
1004524	1	0.2513183	4	0
1004525	2	0.3636044	2	1

Patient ID	Side	Propensity score	Number of tranfusions	Matched
1004526	2	0.785459	2	1
1004527	1	0.2714662	0	1
1004528	1	0.623601	1	1
1004529	1	0.5251476	2	1
1004530	1	0.6339748	0	0
1004531	2	0.0845825	9	0
1004532	2	0.9557483	0	0
1004533	1	0.7340729	0	1
1004534	2	0.6909467	0	1
1004535	2	0.1698545	3	1
1004536	2	0.9568595	0	0
1004536	1	0.9568595	0	0
1004537	1	0.5591499	2	1
1004538	2	0.9211168	0	0
1004539	1	0.3602291	4	1
1004540	1	0.7471596	0	1
1004541	2	0.3530725	6	0
1004542	1	0.9070476	0	0
1004543	1	0.865544	0	0
1004544	2	0.7577626	0	1
1004545	2	0.6693853	0	1
1004546	2	0.678968	2	1
1004547	2	0.739302	0	1
1004548	2	0.6172903	2	1
1004549	2	0.9482724	0	0
1004550	2	0.0511104	12	0
1004551	1	0.9256307	0	0
1004552	1	0.7759587	0	0
1004553	2	0.407293	0	1
1004554	1	0.1350739	5	0
1004555	1	0.6580393	0	0
1004556	1	0.7485968	0	0
1004556	2	0.5099006	0	1
1004557	2	0.6889592	0	0
1004558	1	0.3050708	5	1
1004559	2	0.5424572	0	1
1004560	1	0.9339128	0	0
1004561	2	0.0901058	0	1
1004562	1	0.2334151	3	1

Patient ID	Side	Propensity score	Number of tranfusions	Matched
1004563	1	0.3352106	9	1
1004564	1	0.0121565	17	0
1004565	1	0.8709506	0	0
1004566	2	0.9148346	0	0
1004567	2	0.9743555	0	1
1004568	1	0.8881255	0	0
1004569	1	0.7803655	0	0
1004570	1	0.2004336	2	0
1004571	2	0.6265182	1	1
1004572	1	0.7317822	0	1
1004573	2	0.0567909	0	1
1004574	2	0.6165074	1	1
1004575	2	0.8445213	0	0
1004576	2	0.9706023	0	0
1004577	2	0.3739897	0	1
1004578	2	0.473773	2	1
1004579	1	0.2710625	2	1
1004580	2	0.3361404	2	0
1004581	2	0.7403527	0	0
1004582	2	0.2605082	0	1
1004583	1	0.0097832	15	0
1004584	2	0.9377717	0	0
1004585	2	0.7049937	6	1
1004586	2	0.9568595	0	0
1004587	1	0.9366489	0	0
1004588	2	0.4044706	0	1
1004589	1	0.5472813	0	1
1004590	1	0.8644041	0	0
1004590	2	0.784673	0	0
1004591	2	0.9285828	0	0
1004592	2	0.5797566	2	1
1004593	2	0.9380204	0	0
1004594	1	0.8483376	0	0
1004595	1	0.4722031	4	1
1004596	2	0.7107625	0	0
1004597	1	0.1308471	6	1
1004598	2	0.8887281	2	1
1004599	2	0.8303496	0	0
1004600	2	0.8143077	0	0

Patient ID	Side	Propensity score	Number of tranfusions	Matched
1004600	1	0.8143077	0	0
1004601	1	0.3117434	4	0
1004602	1	0.4524742	2	1
1004603	1	0.599822	2	1
1004604	1	0.678347	0	0
1004605	1	0.8859565	0	0
1004606	1	0.9262035	0	0
1004607	1	0.9364763	0	0
1004608	1	0.5041998	0	1
1004609	1	0.6210378	2	1
1004610	2	0.6506863	1	1
1004611	2	0.7007701	0	1
1004612	1	0.8464121	0	1
1004613	1	0.4236214	2	1
1004614	2	0.8095856	0	0
1004615	1	0.8464121	2	1
1004616	2	0.8199402	2	1
1004617	1	0.8583781	2	1
1004618	1	0.8538887	2	1
1004619	1	0.733567	0	1
1004620	1	0.9395485	0	0
1004621	1	0.9366489	0	0
1004622	2	0.4349635	0	1
1004622	1	0.561905	0	1
1004623	2	0.8390119	0	0
1004624	2	0.744074	0	0
1004625	1	0.8143077	0	1
1004626	2	0.5057742	2	1
1004627	2	0.8667763	0	0
1004628	2	0.5057742	0	1
1004629	1	0.9397847	0	0
1004630	1	0.921033	0	1
1004631	1	0.7034586	0	0
1004632	1	0.7211949	0	0
1004633	1	0.9653663	0	0
1004634	2	0.8196841	1	1
1004635	2	0.3724687	0	1
1004635	1	0.3955377	2	1
1004636	2	0.0377839	4	0

Patient ID	Side	Propensity score	Number of tranfusions	Matched
1004637	1	0.9557483	0	0
1004638	1	0.6368605	0	1
1004639	1	0.9639877	0	0
1004640	1	0.9623818	0	0
1004641	1	0.4188789	0	1
1004642	2	0.4584335	2	1
1004642	1	0.3461101	2	1
1004643	1	0.7252052	0	0
1004644	2	0.3057131	6	0
1004645	2	0.7084306	0	1
1004646	1	0.619632	0	1
1004647	2	0.8143077	2	1
1004648	1	0.8722887	0	0
1004649	2	0.0386468	2	0
1004650	2	0.8871914	0	0
1004651	2	0.0372303	2	0
1004652	2	0.5427976	0	1
1004653	2	0.9557483	0	0
1004654	1	0.1810321	4	0
1004655	1	0.6135979	0	1
1004656	1	0.559449	0	0
1004656	2	0.6790591	2	1
1004657	1	0.1795676	0	1
1004658	2	0.8583781	2	1
1004659	1	0.9684105	0	0
1004660	1	0.3833727	0	1
1004661	1	0.9569282	0	0
1004662	1	0.0864653	12	0
1004663	2	0.9376553	0	0
1004664	2	0.3212566	1	1
1004665	1	0.9163173	0	0
1004666	1	0.5601314	0	0
1004667	1	0.2523237	3	0
1004668	1	0.9128584	0	0
1004669	1	0.7783576	0	0
1004670	1	0.8066371	0	0
1004671	1	0.9380204	1	1
1004672	1	0.2802136	0	1
1004673	2	0.9557483	0	0

Patient ID	Side	Propensity score	Number of tranfusions	Matched
1004674	2	0.3697271	0	1
1004675	1	0.6062345	0	0
1004676	1	0.8349296	0	0
1004676	2	0.8349296	0	0
1004677	2	0.4135625	0	1
1004678	2	0.4436663	3	1
1004679	1	0.9482519	0	0
1004680	1	0.8514921	0	0
1004681	2	0.263354	0	1
1004682	2	0.9205213	0	0
1004683	1	0.9457731	0	0
1004684	1	0.9306221	0	0
1004684	2	0.9306221	0	0
1004685	2	0.810253	2	1
1004686	1	0.6084539	0	0
1004687	1	0.8003601	2	1
1004688	2	0.8741737	0	1
1004689	1	0.8604233	2	1
1004690	1	0.2341392	4	0
1004691	1	0.8591355	0	0
1004691	2	0.8830779	0	0
1004692	1	0.2206332	4	0
1004692	2	0.2206332	0	1
1004693	2	0.9366737	0	0
1004694	1	0.9611477	0	0
1004695	1	0.0742841	3	0
1004696	2	0.2062671	2	1
1004696	1	0.2062671	0	1
1004697	2	0.4473092	2	1
1004698	1	0.2974002	2	1
1004698	2	0.4044706	2	1
1004699	2	0.891645	0	0
1004700	2	0.9145093	0	0
1004701	1	0.7511338	0	0
1004701	2	0.6757735	0	1
1004702	2	0.8412643	0	0
1004703	1	0.1474643	2	0
1004704	1	0.2260023	9	0
1004705	2	0.559449	0	0

Patient ID	Side	Propensity score	Number of tranfusions	Matched
1004706	1	0.9236301	0	1
1004707	1	0.7511338	2	1
1004708	1	0.9364763	4	1
1004709	2	0.3250186	3	0
1004710	1	0.2153045	4	0
1004711	1	0.678968	0	0
1004712	2	0.9512951	0	0
1004713	1	0.8684553	0	0
1004714	1	0.2370855	0	1
1004715	2	0.4417876	2	1
1004716	1	0.045735	11	0
1004717	1	0.1842791	4	0
1004718	2	0.6631602	0	1
1004719	2	0.4493408	0	1
1004720	2	0.9285819	0	0
1004721	1	0.779352	0	0
1004722	1	0.3250186	2	1
1004723	1	0.5983813	1	1
1004724	2	0.7530555	0	0
1004725	2	0.6435435	0	1
1004726	2	0.5165051	2	1
1004727	1	0.2801864	4	1
1004728	2	0.7992524	0	0
1004729	1	0.3875718	0	1
1004730	1	0.3514752	1	0
1004731	2	0.9639877	0	0
1004732	1	0.8987589	0	1
1004733	1	0.8880704	0	0
1004733	2	0.9486418	0	0
1004734	1	0.7362633	0	0
1004735	1	0.6581681	0	0
1004736	1	0.6762563	0	1
1004737	1	0.8838318	0	0
1004738	1	0.6370063	0	0
1004739	1	0.5843988	0	0
1004740	2	0.2550362	1	1
1004741	1	0.6210378	0	0
1004742	1	0.6400206	0	0
1004743	1	0.5312783	2	1

Patient ID	Side	Propensity score	Number of tranfusions	Matched
1004743	2	0.2317343	3	1
1004744	1	0.9584746	0	0
1004745	1	0.2067997	6	1
1004746	1	0.7567775	0	0
1004747	1	0.6822033	2	1
1004748	1	0.3091972	4	0
1004749	1	0.9471208	0	0
1004750	1	0.3992271	0	1
1004751	2	0.526943	0	1
1004752	1	0.8392869	0	0
1004753	1	0.1466258	4	0
1004754	1	0.6618387	0	0
1004755	2	0.0531731	5	0
1004756	2	0.407293	0	1
1004757	1	0.739302	2	1
1004758	2	0.8604372	0	0
1004759	1	0.873031	0	0
1004760	1	0.6506863	2	1
1004761	2	0.0887985	4	0
1004762	1	0.8512175	0	1
1004763	1	0.3361404	4	1
1004764	2	0.8160213	3	1
1004764	1	0.8912083	0	0
1004765	2	0.4070335	1	1
1004766	1	0.9557483	0	0
1004767	2	0.5116775	0	1
1004768	1	0.3477466	2	0
1004768	2	0.4715458	2	1
1004769	1	0.2992102	0	1
1004770	2	0.755473	0	0
1004771	1	0.9680528	0	0
1004772	2	0.9796864	0	0
1004773	1	0.639349	1	1
1004774	2	0.9163947	0	0
1004775	1	0.6027397	0	1
1004775	2	0.7174632	0	0
1004776	1	0.5122697	5	1
1004777	2	0.6437801	5	1
1004778	1	0.9653663	0	0

Patient ID	Side	Propensity score	Number of tranfusions	Matched
1004779	2	0.5438708	0	0
1004780	2	0.9557483	0	0
1004781	1	0.3057131	4	0
1004782	1	0.8268883	0	0
1004783	2	0.4037822	14	1
1004784	2	0.4040934	4	0
1004785	2	0.6882286	0	1
1004786	1	0.9680528	0	0
1004787	1	0.87418	0	1
1004788	1	0.6210378	1	1
1004789	2	0.8722887	0	0
1004790	2	0.9585671	0	0
1004791	1	0.6781573	0	0
1004792	2	0.2336602	2	1
1004793	1	0.511364	0	1
1004794	2	0.4229888	0	1
1004795	1	0.9756276	0	0
1004796	1	0.933683	0	0
1004797	2	0.7563266	0	0
1004797	1	0.8963693	0	0
1004798	1	0.6790591	2	1
1004798	2	0.6790591	0	1
1004799	1	0.8322218	0	0
1004800	1	0.9380204	0	0
1004801	1	0.3656997	0	1
1004802	2	0.8590341	2	1
1004803	2	0.4524742	0	1
1004804	2	0.2681206	3	0
1004804	1	0.3790332	0	1
1004805	2	0.882295	0	0
1004806	1	0.5990015	0	1
1004807	1	0.7641478	0	1
1004808	2	0.9398549	0	0
1004809	2	0.4655844	0	1
1004810	1	0.9756276	0	0
1004811	2	0.5016053	1	1
1004812	2	0.194294	0	1
1004812	1	0.4521564	0	1
1004813	2	0.9124406	0	0
1004814	2	0.2686254	2	0

Patient ID	Side	Propensity score	Number of tranfusions	Matched
1004815	2	0.9119742	0	0
1004816	2	0.481933	2	1
1004817	2	0.5162569	0	1
1004817	1	0.7484937	0	1
1004818	1	0.6956104	2	1
1004819	2	0.8152938	0	1
1004820	1	0.475834	0	1
1004821	2	0.3516253	2	1
1004822	2	0.2714662	1	0
1004823	1	0.5860069	6	1
1004824	1	0.925874	0	0
1004825	2	0.8061834	0	0
1004826	2	0.9653663	0	0
1004827	2	0.1799348	2	1
1004827	1	0.1799348	0	1
1004828	2	0.7511338	0	0
1004829	1	0.3798259	2	0
1004830	1	0.2595311	0	1
1004831	1	0.4451098	0	1
1004832	2	0.5762173	0	1
1004833	2	0.4220721	0	1
1004834	1	0.8684553	0	0
1004834	2	0.8684553	0	0
1004835	2	0.9222556	0	0
1004836	1	0.9395485	3	1
1004837	2	0.9119742	0	1
1004838	1	0.4092281	0	1
1004839	1	0.7643871	0	0
1004840	1	0.744074	0	0
1004841	2	0.9366489	0	0
1004841	1	0.9366489	0	0
1004842	1	0.9653663	0	0
1004843	2	0.8515652	1	1
1004844	2	0.011622	8	0
1004845	2	0.9380204	0	1
1004845	1	0.9380204	0	1
1004846	1	0.9119742	0	0
1004847	1	0.8583781	0	0
1004848	2	0.2739091	2	0
1004849	1	0.4379569	0	1

Patient ID	Side	Propensity score	Number of tranfusions	Matched
1004850	2	0.882295	0	0
1004850	1	0.882295	0	0
1004851	2	0.3521751	0	1
1004852	1	0.9557483	0	0
1004853	2	0.8895731	0	0
1004854	1	0.8199402	2	1
1004855	2	0.0205467	3	0
1004856	1	0.8830779	2	1
1004857	1	0.075208	2	0
1004858	1	0.1764874	4	0
1004859	2	0.9550222	0	0
1004860	1	0.678968	0	1
1004860	2	0.678968	2	1
1004861	2	0.7564785	0	0
1004862	2	0.321832	2	1
1004863	2	0.5165051	2	1
1004864	1	0.3699846	0	1
1004865	2	0.8143077	0	0
1004866	1	0.6174883	0	1
1004867	2	0.6174811	2	1
1004868	1	0.8819084	0	1
1004869	2	0.823929	0	1
1004870	1	0.1474643	2	0
1004871	2	0.9571506	0	0
1004872	2	0.2469185	0	1
1004873	2	0.928416	0	0
1004874	2	0.7892495	0	1
1004875	1	0.4282856	2	0
1004876	1	0.6881271	2	1
1004877	1	0.8892304	0	0
1004878	2	0.6250799	0	1
1004879	2	0.4268092	2	1
1004880	2	0.9366489	0	0
1004881	1	0.9098565	0	0
1004882	2	0.7451044	0	1
1004883	2	0.810253	0	0
1004884	1	0.8242614	0	0
1004885	2	0.9415828	0	0
1004886	2	0.3028421	0	1

Patient ID	Side	Propensity score	Number of tranfusions	Matched
1004887	1	0.9264305	2	1
1004888	2	0.9699928	0	0
1004889	1	0.4802569	2	1
1004890	2	0.9377717	0	0
1004891	2	0.1136335	3	0
1004892	2	0.5796341	0	1
1004893	1	0.8720679	0	0
1004893	2	0.8720679	0	0
1004894	2	0.125103	2	0
1004895	2	0.5287857	0	1
1004896	1	0.0437246	5	0
1004897	2	0.8309514	1	1
1004898	1	0.2801864	2	1
1004899	1	0.4209728	0	1
1004899	2	0.4274472	4	1
1004900	1	0.4006762	1	1
1004901	2	0.9145093	0	0
1004902	2	0.2067997	3	0
1004903	1	0.9639877	0	0
1004904	1	0.8410779	0	0
1004904	2	0.7567775	0	0
1004905	2	0.9364763	0	0
1004906	2	0.9459836	0	0
1004907	2	0.4452966	0	1
1004908	1	0.6103809	7	1
1004909	2	0.8512175	0	0
1004910	1	0.9653663	0	0
1004911	2	0.8380845	0	0
1004912	1	0.7062283	2	1
1004913	2	0.9557483	0	0
1004914	1	0.8569584	0	0
1004915	2	0.035823	22	1
1004916	2	0.3065288	4	0
1004917	1	0.9684105	0	0
1004918	1	0.4353576	4	1
1004919	2	0.8651406	0	0
1004920	2	0.559449	4	1
1004921	2	0.1842078	8	1
1004922	2	0.9243086	0	0

Patient ID	Side	Propensity score	Number of tranfusions	Matched
1004923	1	0.7680654	1	1
1004924	2	0.380564	4	0
1004925	1	0.9339128	0	0
1004926	2	0.839206	0	0
1004926	1	0.9366737	0	0
1004927	1	0.8933949	0	0
1004928	2	0.8380845	0	0
1004929	1	0.2517358	0	1
1004930	2	0.7783576	0	1
1004931	1	0.7845558	2	1
1004932	2	0.3650894	0	1
1004933	1	0.1675961	0	1
1004934	1	0.8486744	0	0
1004935	1	0.8909839	0	0
1004936	1	0.3305501	0	1
1004936	2	0.5057742	0	1
1004937	1	0.9288847	0	0
1004938	1	0.750596	0	1
1004938	2	0.750596	4	1
1004939	1	0.389276	2	1
1004940	1	0.9546245	0	0
1004940	2	0.9623802	0	0
1004941	1	0.5162569	0	1
1004942	2	0.9211168	0	0
1004943	2	0.6244454	0	1
1004944	1	0.7749288	0	0
1004945	1	0.801863	0	0
1004946	1	0.6805081	2	1
1004947	1	0.6143042	0	1
1004947	2	0.7705859	0	0
1004948	2	0.948741	0	0
1004949	2	0.6210378	0	0
1004950	1	0.5623317	0	1
1004951	1	0.7865697	2	1
1004952	2	0.1137119	7	0
1004953	2	0.8652821	0	0
1004953	1	0.8652821	0	0
1004954	2	0.9089601	0	0
1004955	1	0.9380204	0	0

Patient ID	Side	Propensity score	Number of tranfusions	Matched
1004956	2	0.4188789	0	1
1004957	2	0.2509839	3	0
1004958	1	0.8474815	0	1
1004959	2	0.3999621	0	1
1004960	1	0.8508557	0	1
1004961	1	0.2550914	2	0
1004962	1	0.9555191	0	0
1004963	1	0.7466034	0	0
1004964	1	0.7317822	1	1
1004965	2	0.8776536	0	1
1004966	1	0.9482724	0	0
1004967	1	0.6349469	0	1
1004968	2	0.6339748	2	1
1004969	2	0.9021338	0	0
1004970	1	0.683496	2	1
1004971	1	0.2449849	0	1
1004972	1	0.7511338	0	1
1004972	2	0.7511338	1	1
1004973	1	0.8103819	0	0
1004974	2	0.2044553	1	0
1004975	2	0.810253	0	0
1004976	2	0.475834	1	1
1004977	1	0.623785	0	1
1004978	1	0.925874	0	0
1004979	2	0.9395485	0	0
1004980	2	0.5870586	0	1
1004981	1	0.7240763	0	0
1004982	2	0.6679912	2	1
1004982	1	0.5767474	0	1
1004983	1	0.1525709	2	1
1004984	1	0.9623802	0	0
1004985	1	0.4415582	2	1
1004985	2	0.622981	0	0
1004986	1	0.8572502	2	1
1004987	1	0.9623802	0	0
1004988	2	0.8071236	0	0
1004989	1	0.1275878	2	0
1004989	2	0.9247459	0	0
1004990	2	0.9163173	0	0

Patient ID	Side	Propensity score	Number of tranfusions	Matched
1004991	2	0.5238844	13	1
1004992	1	0.9454951	0	0
1004993	1	0.6308935	0	1
1004994	1	0.5391658	8	1
1004995	2	0.1727274	2	0
1004996	1	0.2421212	0	1
1004997	2	0.6939031	3	1
1004998	1	0.167182	2	0
1004999	1	0.0649558	4	0
1005000	1	0.9706023	0	0
1005001	1	0.8487278	0	0
1005001	2	0.8624214	0	0
1005002	1	0.765845	0	0
1005003	1	0.4663877	0	1
1005004	1	0.8923432	0	0
1005005	1	0.7138674	2	1
1005006	1	0.5841954	0	0
1005007	2	0.434783	0	1
1005008	1	0.2884745	3	1
1005009	1	0.6103809	0	1
1005010	2	0.7222826	0	1
1005011	1	0.0986374	4	0
1005012	2	0.843932	1	1
1005013	1	0.9684105	0	0
1005014	1	0.7445205	0	1
1005015	2	0.349112	2	1
1005016	1	0.810253	0	0
1005016	2	0.810253	0	0
1005017	1	0.3307663	0	1
1005018	2	0.9040454	0	0
1005019	1	0.0761348	6	0
1005020	1	0.6210378	0	0
1005021	2	0.9454951	0	0
1005022	1	0.0394142	3	0
1005023	2	0.2467381	0	1
1005024	1	0.4139172	0	1
1005025	1	0.8102122	0	0
1005026	2	0.8924138	0	0
1005027	1	0.7511399	5	1

Patient ID	Side	Propensity score	Number of tranfusions	Matched
1005028	2	0.7622421	0	1
1005029	2	0.5211484	5	1
1005029	1	0.5740564	4	1
1005030	2	0.9471208	0	0
1005031	1	0.7836873	0	0
1005032	2	0.1898562	4	1
1005033	1	0.7786686	0	0
1005034	1	0.2140578	3	0
1005035	2	0.9555191	0	0
1005036	1	0.8892304	0	0
1005037	2	0.9000514	0	0
1005038	1	0.898414	0	0
1005039	1	0.7609577	0	0
1005040	2	0.3936187	0	1
1005041	1	0.8531178	0	0
1005041	2	0.9163173	0	0
1005042	2	0.7974147	2	1
1005043	2	0.8512767	0	0
1005044	1	0.9408087	0	1
1005045	2	0.9611736	0	0
1005046	1	0.0147431	15	1
1005047	2	0.8870113	0	0
1005048	1	0.8023827	0	1
1005049	1	0.9120077	0	0
1005050	2	0.3904594	2	0
1005051	1	0.7263637	2	1
1005052	2	0.8380845	0	0
1005053	2	0.9557483	0	0
1005054	2	0.4104498	2	1
1005055	2	0.9186629	0	0
1005056	1	0.9512951	0	0
1005057	1	0.9135031	0	0
1005058	2	0.5937697	1	1
1005059	1	0.2801864	2	0
1005060	1	0.4452966	0	1
1005060	2	0.9256307	4	1
1005061	2	0.5312783	2	1
1005062	2	0.810253	0	0
1005063	2	0.25952	1	1

Patient ID	Side	Propensity score	Number of tranfusions	Matched
1005064	1	0.3855807	0	1
1005065	1	0.1548002	6	0
1005066	1	0.9694243	0	0
1005067	1	0.6465287	2	1
1005068	1	0.4006762	3	1
1005069	2	0.8103819	0	0
1005070	2	0.6964539	0	1
1005071	2	0.1296389	4	1
1005072	2	0.7567775	0	0
1005073	2	0.948741	0	0
1005074	1	0.9653663	0	0
1005075	2	0.9718675	0	0
1005076	2	0.8654763	0	0
1005077	1	0.4551236	0	1
1005078	2	0.9611736	0	0
1005079	2	0.9568595	0	0
1005080	1	0.8462635	0	0
1005081	2	0.8368876	0	0
1005082	2	0.9555191	0	0
1005083	1	0.9119742	2	1
1005084	1	0.143219	5	0
1005085	1	0.4305716	3	0
1005086	1	0.1825326	4	0
1005087	2	0.9527564	0	0
1005088	2	0.9684105	4	1
1005089	2	0.8182057	1	1
1005090	2	0.6852601	2	1
1005091	1	0.8817283	0	0
1005091	2	0.8817283	0	1
1005092	2	0.4545422	2	1
1005092	1	0.5312783	2	1
1005093	1	0.8177543	0	0
1005094	1	0.9366489	0	0
1005095	1	0.0670476	5	0
1005096	1	0.9145093	0	0
1005097	1	0.1548002	2	0
1005098	1	0.4864515	3	1
1005099	1	0.5307699	0	1
1005100	1	0.5935675	0	1

Patient ID	Side	Propensity score	Number of tranfusions	Matched
1005101	1	0.1778219	10	0
1005102	2	0.1091269	2	0
1005103	1	0.0914561	4	0
1005104	1	0.9898134	0	0
1005105	2	0.755473	2	1
1005106	1	0.0795015	7	0
1005107	1	0.5308739	0	1
1005108	1	0.8622236	0	0
1005109	2	0.1270491	2	0
1005110	1	0.6909467	1	1
1005111	1	0.8624214	0	0
1005112	1	0.8143077	0	0
1005113	1	0.4353576	2	0
1005114	2	0.0448526	3	0
1005114	1	0.1232325	2	0
1005115	1	0.8021703	0	0
1005116	1	0.0565318	0	1
1005117	2	0.5591499	0	1
1005118	1	0.2067997	0	1
1005119	1	0.1962029	0	1
1005120	1	0.6321596	2	1
1005121	1	0.9457731	0	0
1005122	2	0.7483358	0	0
1005123	2	0.5359595	0	1
1005124	1	0.8355938	0	0
1005125	2	0.5435919	0	1
1005126	1	0.739302	0	1
1005127	2	0.87418	0	1
1005128	1	0.5623317	2	1
1005129	2	0.9163173	0	0
1005130	1	0.8208233	0	0
1005131	1	0.817672	0	0
1005132	2	0.9481091	0	1
1005133	2	0.7010806	0	0
1005134	2	0.5078036	0	1
1005135	1	0.5056137	0	1
1005136	1	0.8541505	1	1
1005137	1	0.710155	0	1
1005138	1	0.7578386	0	0

Patient ID	Side	Propensity score	Number of tranfusions	Matched
1005139	2	0.9039453	0	0
1005140	2	0.9148016	0	0
1005141	2	0.9565483	0	0
1005142	1	0.8722887	0	1
1005143	2	0.6210378	0	1
1005144	2	0.914542	2	1
1005145	1	0.8722887	0	0
1005146	1	0.1032112	5	0
1005147	1	0.3464321	2	1
1005148	1	0.3784805	11	1
1005149	2	0.321832	3	1
1005150	2	0.3935633	3	0
1005151	1	0.7838778	2	1
1005151	2	0.8151675	2	1
1005152	2	0.925054	0	0
1005153	1	0.6349469	0	0
1005154	2	0.7797947	0	0
1005155	1	0.8159437	1	1
1005156	1	0.9134025	0	0
1005157	1	0.810253	0	0
1005158	2	0.4282856	2	1
1005159	1	0.3649532	2	1
1005160	1	0.3501185	2	1
1005161	1	0.8182057	0	0
1005162	2	0.8095856	0	0
1005163	1	0.5920472	0	1
1005163	2	0.9348254	0	0
1005164	1	0.6808008	0	0
1005165	1	0.4133672	0	1
1005166	1	0.714073	0	0
1005167	2	0.805528	0	0
1005168	1	0.8380845	0	0
1005169	1	0.9796864	0	0
1005170	2	0.1404862	2	0
1005171	1	0.5623317	0	1
1005172	1	0.7628868	11	1
1005173	1	0.8348097	0	1
1005174	2	0.2341951	2	0
1005175	1	0.3711177	3	0

Patient ID	Side	Propensity score	Number of tranfusions	Matched
1005175	2	0.6021064	0	1
1005176	2	0.8180251	0	0
1005177	2	0.3636044	0	1
1005178	1	0.7797947	0	1
1005179	1	0.6093747	0	0
1005180	1	0.4144451	2	1
1005181	1	0.473773	2	1
1005182	1	0.501405	4	1
1005183	2	0.1338009	2	0
1005184	1	0.9366489	0	0
1005185	1	0.5294019	0	1
1005186	2	0.9029307	0	1
1005187	2	0.7672793	0	0
1005188	2	0.8767001	0	0
1005189	1	0.817672	0	0
1005190	1	0.4518385	0	1
1005191	2	0.2768618	0	1
1005192	1	0.8543689	10	1
1005193	2	0.6618387	0	1
1005194	1	0.3212566	0	1
1005195	2	0.473773	0	1
1005196	1	0.1754709	3	0
1005197	2	0.7128839	2	1
1005198	2	0.9226706	0	0
1005199	1	0.9207713	0	0
1005200	1	0.5623317	2	1
1005201	1	0.923833	0	0
1005202	1	0.4003731	6	0
1005203	1	0.8267231	0	0
1005204	1	0.3049089	3	0
1005205	1	0.4232768	2	0
1005206	1	0.2794593	3	0
1005207	2	0.6174927	0	1
1005208	1	0.3858023	0	1
1005209	1	0.4144451	0	1
1005210	1	0.5138417	2	1
1005211	2	0.1384197	2	0
1005212	2	0.2972059	1	1
1005213	1	0.6349469	0	1

Patient ID	Side	Propensity score	Number of tranfusions	Matched
1005214	2	0.4700425	2	1
1005215	2	0.8348297	2	1
1005216	1	0.5413551	2	1
1005217	1	0.3838588	0	1
1005218	1	0.4174139	0	1
1005219	2	0.8684553	0	0
1005220	2	0.9366489	0	0
1005221	2	0.6676309	0	1
1005221	1	0.8485192	0	0
1005222	2	0.9557483	0	0
1005222	1	0.9557483	0	0
1005223	2	0.1859022	4	0
1005224	1	0.8801035	0	0
1005225	1	0.5359595	0	1
1005226	1	0.9145093	0	0
1005227	2	0.4592113	0	1
1005227	1	0.7609577	5	1
1005228	1	0.5935675	0	1
1005229	1	0.8143077	0	0
1005230	2	0.3718861	0	1
1005231	2	0.6909467	0	0
1005232	2	0.8143077	0	0
1005233	1	0.3268703	2	1
1005234	1	0.9357085	0	0
1005235	1	0.8202391	0	0
1005236	2	0.423243	0	1
1005237	2	0.9573394	0	0
1005238	2	0.755473	0	1
1005238	1	0.755473	2	1
1005239	1	0.8114735	0	0
1005240	1	0.3067821	4	0
1005241	1	0.9898134	0	0
1005242	1	0.892649	2	1
1005243	1	0.7956537	0	0
1005244	1	0.7010806	0	0
1005245	2	0.9551294	0	0
1005246	1	0.4733407	0	1
1005247	2	0.9074583	0	1
1005248	2	0.678968	0	0

Patient ID	Side	Propensity score	Number of tranfusions	Matched
1005249	1	0.8549261	0	1
1005250	1	0.9395485	0	0
1005251	2	0.9555191	0	0
1005252	1	0.2695111	4	1
1005252	2	0.2458921	2	0
1005253	2	0.481933	0	1
1005254	1	0.8752306	0	0
1005255	2	0.0649558	4	0
1005256	1	0.6944648	0	0
1005257	1	0.7530076	0	0
1005258	2	0.4833765	1	1
1005259	2	0.8052972	0	1
1005260	1	0.321832	0	1
1005261	1	0.8251629	0	0
1005262	1	0.9571506	0	0
1005263	2	0.6826809	2	1
1005264	1	0.9568595	2	1
1005265	1	0.9239004	0	0
1005266	2	0.9898134	0	0
1005267	1	0.6790591	2	1
1005268	1	0.8280681	0	0
1005269	2	0.7059057	2	1
1005270	2	0.9653663	0	0
1005271	2	0.808008	0	0
1005272	1	0.3028421	4	1
1005273	2	0.378616	2	1
1005274	1	0.6687502	2	1
1005275	1	0.8622236	0	0
1005276	2	0.7138674	0	0
1005276	1	0.9163173	0	0
1005277	1	0.1456555	2	0
1005277	2	0.2610932	4	1
1005278	1	0.7751945	0	0
1005279	2	0.4037822	0	1
1005280	2	0.7451044	0	1
1005281	2	0.0567909	6	1
1005282	2	0.9735961	0	0
1005283	2	0.9353824	0	0
1005284	1	0.6504241	2	1

Patient ID	Side	Propensity score	Number of tranfusions	Matched
1005285	1	0.8268883	0	0
1005286	1	0.7277866	2	1
1005287	1	0.8671892	1	1
1005288	2	0.8671572	0	0
1005289	2	0.8488542	0	0
1005290	1	0.5783081	7	1
1005291	2	0.1743616	2	1
1005292	1	0.1741835	3	0
1005293	2	0.7838778	0	1
1005293	1	0.7838778	0	1
1005294	2	0.8172835	0	0
1005295	2	0.9732879	0	0
1005296	2	0.1683917	11	0
1005297	2	0.9471208	0	0
1005298	2	0.5067485	0	1
1005299	2	0.801863	0	0
1005300	2	0.0409759	1	0
1005301	1	0.9607372	0	0
1005302	1	0.9119742	0	0
1005303	2	0.81959	0	0
1005304	1	0.8380845	2	1
1005305	2	0.9141047	0	0
1005306	1	0.9611736	0	0
1005307	1	0.9527564	0	0
1005308	1	0.7415499	1	1
1005309	1	0.5308739	5	1
1005310	2	0.7010806	0	1
1005311	1	0.7797947	0	1
1005312	2	0.3658852	0	1
1005313	2	0.321832	1	0
1005314	2	0.9611736	0	0
1005315	1	0.8722887	0	0
1005316	2	0.2163809	4	0
1005317	2	0.3065288	2	1
1005318	1	0.6210378	0	1
1005319	1	0.928416	0	0
1005320	1	0.1743547	3	0
1005320	2	0.4424432	6	0
1005321	2	0.9197193	0	0

Patient ID	Side	Propensity score	Number of tranfusions	Matched
1005322	1	0.9142818	0	0
1005323	1	0.9265721	0	0
1005324	2	0.7060583	2	1
1005325	2	0.1630668	2	0
1005326	2	0.8159437	0	0
1005327	1	0.321832	2	1
1005328	1	0.8503392	0	0
1005329	1	0.9380204	0	0
1005330	2	0.8955071	0	0
1005331	2	0.0895369	4	0
1005332	1	0.810253	0	0
1005332	2	0.810253	0	0
1005333	1	0.258208	0	1
1005334	1	0.8739372	0	1
1005335	1	0.3514923	0	1
1005336	2	0.8892304	0	0
1005336	1	0.9380204	0	0
1005337	2	0.6565705	2	1
1005338	1	0.7597614	0	0
1005339	2	0.1717555	3	0
1005340	2	0.7668612	0	1
1005341	1	0.9342051	0	0
1005342	2	0.519588	15	1
1005343	1	0.9211168	0	0
1005344	1	0.5099006	4	1
1005345	2	0.810253	0	0
1005346	1	0.9653663	0	0
1005347	2	0.9315427	0	0
1005348	2	0.7128839	0	1
1005349	2	0.8450363	0	1
1005350	1	0.9081043	0	0
1005351	1	0.8060101	0	0
1005352	1	0.7909005	0	0
1005353	2	0.3285376	0	1
1005354	1	0.3212566	2	0
1005355	2	0.2517358	0	1
1005356	1	0.965012	0	0
1005357	2	0.9383534	0	0
1005358	2	0.6508769	2	1

Patient ID	Side	Propensity score	Number of tranfusions	Matched
1005359	2	0.6179632	0	1
1005360	2	0.81357	0	0
1005360	1	0.7904238	0	1
1005361	1	0.8684553	0	1
1005362	1	0.2539187	0	1
1005363	1	0.8260662	2	1
1005364	1	0.8196841	0	1
1005365	2	0.7317822	0	1
1005366	2	0.8604233	0	0
1005367	2	0.6271352	2	1
1005368	2	0.2753559	5	1
1005369	1	0.81357	0	0
1005370	1	0.9827961	0	0
1005370	2	0.9680528	0	0
1005371	2	0.6129012	1	1
1005372	1	0.9482519	0	0
1005373	1	0.5350229	2	1
1005374	1	0.4946648	0	1
1005375	2	0.6343521	0	0
1005376	1	0.5312783	0	1
1005377	1	0.5685832	9	1
1005378	1	0.2992102	2	1
1005379	2	0.8242614	0	0
1005380	2	0.5051612	2	1
1005380	1	0.5111606	0	1
1005381	1	0.6084539	0	0
1005382	1	0.8515652	0	0
1005383	2	0.4032728	0	1
1005384	2	0.1592934	3	0
1005385	1	0.678968	0	0
1005386	1	0.7994878	0	0
1005386	2	0.678968	0	0
1005387	1	0.4906955	1	1
1005388	2	0.9648087	2	1
1005389	2	0.8861318	0	0
1005390	1	0.8765468	0	0
1005391	1	0.3320819	0	1
1005392	2	0.8656695	0	0
1005393	1	0.6068991	5	1

Patient ID	Side	Propensity score	Number of tranfusions	Matched
1005394	1	0.8823384	2	1
1005395	1	0.559449	0	0
1005396	1	0.7471596	0	0
1005397	1	0.6179632	0	1
1005398	1	0.4831417	0	1
1005399	1	0.767398	0	0
1005400	2	0.1346475	4	0
1005401	2	0.4524742	5	1
1005401	1	0.4524742	2	1
1005402	2	0.1542122	0	1
1005403	1	0.1859022	2	0
1005403	2	0.1859022	0	1
1005404	2	0.6210378	0	1
1005405	2	0.7701729	0	0
1005406	1	0.467087	0	1
1005407	1	0.5308739	2	1
1005407	2	0.6544533	0	1
1005408	2	0.9366489	0	0
1005409	2	0.8793763	2	1
1005410	1	0.8987589	0	0
1005410	2	0.9684105	0	0
1005411	2	0.9743555	0	0
1005412	2	0.6807669	0	0
1005413	2	0.8348097	2	1
1005413	1	0.8348097	4	1
1005414	1	0.1442475	2	1
1005415	1	0.4706002	2	1
1005416	1	0.9376553	0	1
1005417	2	0.2550914	0	1
1005418	2	0.3212566	0	1
1005419	2	0.4387719	4	1
1005420	1	0.2550914	0	1
1005421	1	0.3477466	2	0
1005422	1	0.3065288	2	1
1005423	2	0.6631602	0	1
1005424	2	0.7138674	0	1
1005425	1	0.622981	1	1
1005426	1	0.7490443	0	0
1005427	2	0.9398549	0	0

Patient ID	Side	Propensity score	Number of tranfusions	Matched
1005428	2	0.9163173	0	0
1005429	1	0.608248	0	1
1005430	1	0.9197193	0	0
1005431	2	0.9276642	0	0
1005432	2	0.7783576	0	1
1005432	1	0.693725	0	0
1005433	2	0.8979311	0	0
1005434	1	0.5012173	0	1
1005435	1	0.8267231	0	0
1005436	2	0.9039453	0	0
1005437	2	0.9624106	0	0
1005438	2	0.9590093	0	0
1005439	2	0.7840198	0	0
1005440	2	0.8199402	2	1
1005440	1	0.5623317	2	1
1005441	1	0.8471798	1	1
1005442	2	0.9555191	0	0
1005443	2	0.3236385	2	1
1005444	1	0.1223756	2	0
1005445	1	0.0899884	2	1
1005446	1	0.4689873	0	1
1005447	2	0.7892969	0	0
1005448	2	0.8979311	2	1
1005449	1	0.6696168	3	1
1005450	2	0.7341295	0	0
1005451	2	0.8580465	0	0
1005451	1	0.8580465	0	0
1005452	2	0.9684105	0	0
1005453	1	0.3305501	2	1
1005454	2	0.2250138	4	0
1005455	1	0.8464121	0	0
1005456	1	0.766746	2	1
1005457	2	0.678968	0	0
1005458	2	0.0579512	2	0
1005459	2	0.7761444	0	0
1005459	1	0.765845	0	0
1005460	1	0.1859022	0	1
1005461	2	0.9482724	0	0
1005462	1	0.4516069	0	1

Patient ID	Side	Propensity score	Number of tranfusions	Matched
1005463	1	0.4011498	3	1
1005464	1	0.9530739	0	0
1005465	1	0.1878532	4	0
1005466	1	0.9731231	0	0
1005467	1	0.9639877	0	1
1005468	2	0.8615083	0	0
1005469	2	0.7613885	2	1
1005470	2	0.8143077	0	1
1005471	1	0.6361699	0	0
1005472	2	0.3770483	1	1
1005473	1	0.7698659	7	1
1005474	1	0.2972059	0	1
1005475	2	0.2945167	5	1
1005476	2	0.9557483	0	0
1005477	1	0.9481091	0	0
1005478	2	0.3865072	0	1
1005479	2	0.4254257	4	1
1005480	2	0.0517391	5	0
1005481	1	0.4733407	3	1
1005482	1	0.7348097	0	0
1005483	2	0.7600489	0	0
1005484	1	0.4415582	1	0
1005485	1	0.4735617	2	1
1005486	1	0.7838778	0	0
1005487	1	0.9431339	0	0
1005488	2	0.9124995	0	0
1005489	1	0.4793986	2	1
1005490	1	0.557265	0	1
1005490	2	0.7263637	0	1
1005491	2	0.7723468	0	0
1005492	1	0.9051418	0	0
1005493	2	0.4722031	2	1
1005494	2	0.4240038	0	1
1005495	2	0.5641194	2	1
1005496	1	0.9481907	0	0
1005497	2	0.678968	0	0
1005497	1	0.678968	0	0
1005498	2	0.4398131	7	0
1005499	1	0.8048824	0	0

Patient ID	Side	Propensity score	Number of tranfusions	Matched
1005499	2	0.944025	0	0
1005500	1	0.97214	0	0
1005501	1	0.9775355	0	0
1005502	1	0.6826809	0	1
1005503	2	0.6159782	2	1
1005504	1	0.7511338	0	0
1005505	1	0.8583395	0	0
1005506	1	0.9512951	0	0
1005507	1	0.5805676	0	1
1005508	1	0.8675035	0	0
1005509	2	0.3641067	2	0
1005510	2	0.6331858	0	0
1005511	1	0.941524	0	0
1005512	2	0.882295	0	0
1005512	1	0.882295	0	0
1005513	2	0.6339748	0	0
1005514	2	0.053804	6	0
1005515	1	0.8182057	0	0
1005516	1	0.8703418	0	1
1005517	1	0.9350175	0	0
1005518	1	0.7010806	0	0
1005519	1	0.8303496	0	1
1005520	2	0.9121083	0	0
1005521	1	0.678968	0	0
1005522	1	0.4518385	3	1
1005523	1	0.9197193	0	0
1005524	1	0.681442	0	0
1005525	1	0.599822	2	1
1005526	2	0.6619618	0	0
1005527	1	0.9000514	0	0
1005528	1	0.5406237	0	1
1005529	2	0.3503538	0	1
1005530	1	0.6846067	2	1
1005531	1	0.9119742	0	0
1005532	2	0.7861992	0	1
1005533	2	0.7835842	0	1
1005534	1	0.7451044	2	1
1005535	2	0.7644362	0	0
1005536	1	0.9166842	0	0

Patient ID	Side	Propensity score	Number of tranfusions	Matched
1005537	2	0.8733991	1	1
1005538	1	0.8569584	2	1
1005539	1	0.8244078	0	0
1005540	1	0.2801864	4	1
1005541	2	0.9684105	0	0
1005542	2	0.8492618	0	0
1005543	1	0.7010806	2	1
1005544	2	0.3961032	0	1
1005545	1	0.693725	0	0
1005546	2	0.750596	0	1
1005547	1	0.7515005	0	0
1005548	1	0.4209728	0	1
1005549	2	0.9380204	0	0
1005550	2	0.9680528	0	0
1005551	2	0.8626183	4	1
1005552	1	0.7526632	0	1
1005553	2	0.6790591	2	1
1005554	2	0.6535684	0	1
1005555	1	0.8244275	0	0
1005556	1	0.0696045	5	0
1005557	1	0.801863	0	0
1005557	2	0.6822033	0	0
1005558	1	0.7505902	0	1
1005559	2	0.56959	0	1
1005560	1	0.6444111	0	0
1005561	2	0.9402535	0	0
1005562	2	0.1337972	15	0
1005563	1	0.0901954	9	0
1005564	1	0.757626	2	1
1005565	2	0.9718675	0	0
1005566	2	0.8583781	0	0
1005566	1	0.8583781	2	1
1005567	1	0.8720679	2	1
1005568	2	0.7264832	4	1
1005569	2	0.0084265	5	0
1005570	1	0.921033	2	1
1005571	2	0.1606193	3	0
1005572	2	0.7877594	2	1
1005573	2	0.9653663	0	0

Patient ID	Side	Propensity score	Number of tranfusions	Matched
1005574	1	0.0211833	5	0
1005575	2	0.4261515	2	1
1005576	1	0.0447226	12	0
1005577	2	0.2028052	6	1
1005578	2	0.8254169	0	1
1005579	1	0.9569282	0	0
1005580	1	0.965012	0	0
1005581	1	0.823929	0	0
1005582	1	0.2286018	0	1
1005582	2	0.8151675	0	1
1005583	1	0.7853797	0	1
1005584	2	0.8887281	2	1
1005585	2	0.3236385	0	1
1005586	2	0.8652821	0	0
1005587	1	0.7994878	0	0
1005588	2	0.8630536	0	0
1005589	1	0.9557483	0	0
1005590	1	0.1859022	3	0
1005591	2	0.9573737	0	0
1005591	1	0.9573394	0	0
1005592	1	0.9366248	0	0
1005593	2	0.3251336	2	0
1005594	2	0.3798259	4	1
1005594	1	0.3798259	2	0
1005595	1	0.8895731	0	0
1005595	2	0.8590019	0	0
1005596	1	0.5057742	2	1
1005597	1	0.5854051	0	1
1005598	1	0.9557483	0	0
1005599	1	0.0228245	1	0
1005600	1	0.9340456	0	0
1005601	2	0.8151715	2	1
1005602	1	0.9380204	0	0
1005603	2	0.8271991	0	0
1005603	1	0.8187	0	0
1005604	1	0.8412643	0	1
1005604	2	0.9366248	0	0
1005605	2	0.8651406	0	0
1005606	2	0.8771182	0	0

Patient ID	Side	Propensity score	Number of tranfusions	Matched
1005607	1	0.9493857	0	0
1005608	2	0.9557483	0	0
1005609	1	0.5025039	0	1
1005610	2	0.4556785	0	1
1005611	2	0.9809673	0	0
1005612	1	0.9395485	0	0
1005613	2	0.7077223	2	1
1005614	1	0.8583781	0	1
1005615	1	0.5308739	2	1
1005616	1	0.9765373	0	0
1005617	1	0.0526381	7	0
1005618	1	0.9315427	0	1
1005619	1	0.755473	0	1
1005620	1	0.2974002	2	1
1005621	2	0.5740564	0	0
1005622	1	0.8464121	4	1
1005623	2	0.0746281	4	0
1005624	1	0.8583781	0	1
1005625	1	0.2807186	1	0
1005626	1	0.0621669	9	0
1005627	1	0.8908069	0	0
1005628	1	0.5985009	2	1
1005629	2	0.7115915	0	1
1005630	2	0.5312783	0	1
1005631	2	0.9471208	0	0
1005632	1	0.6210378	2	1
1005633	1	0.6457154	0	0
1005634	1	0.711776	0	1
1005635	1	0.8267231	0	0
1005635	2	0.8182057	0	0
1005636	1	0.8946468	0	1
1005637	2	0.9016513	0	0
1005638	2	0.8311099	0	0
1005638	1	0.8311099	0	0
1005639	1	0.3387799	0	1
1005640	1	0.2681206	2	0
1005641	1	0.4921914	0	1
1005642	2	0.7763584	0	0
1005643	1	0.8381982	0	0

Patient ID	Side	Propensity score	Number of tranfusions	Matched
1005644	1	0.2760632	2	0
1005644	2	0.3885172	2	1
1005645	1	0.1756432	1	0
1005646	2	0.9471208	0	0
1005647	2	0.6368605	1	1
1005648	2	0.4044706	2	1
1005648	1	0.5308739	2	1
1005649	1	0.3028421	2	1
1005650	1	0.766746	0	1
1005651	1	0.3158548	0	1
1005652	1	0.948741	2	1
1005653	2	0.9186629	0	0
1005654	1	0.9265721	0	1
1005655	1	0.5422639	2	1
1005656	2	0.2204023	3	0
1005657	2	0.2783631	0	1
1005658	1	0.9405123	0	1
1005659	2	0.9684361	0	0
1005660	1	0.242314	2	0
1005661	2	0.8199402	2	1
1005662	2	0.701194	0	1
1005663	2	0.9023173	2	1
1005664	2	0.8380845	0	0
1005665	1	0.330969	1	1
1005666	2	0.6273714	0	1
1005666	1	0.6510919	0	0
1005667	2	0.9119742	0	1
1005668	1	0.1312381	3	0
1005669	1	0.8120355	0	0
1005670	1	0.622981	0	1
1005671	1	0.6210378	2	1
1005672	1	0.9278036	0	0
1005673	2	0.1640022	6	0
1005674	1	0.2945167	2	1
1005675	2	0.3464321	0	1
1005676	1	0.1349551	1	0
1005677	1	0.693725	2	1
1005678	2	0.4144451	4	1
1005679	2	0.3183732	2	0

Patient ID	Side	Propensity score	Number of tranfusions	Matched
1005680	1	0.473773	0	1
1005681	2	0.7199393	0	1
1005682	1	0.8242614	0	0
1005683	2	0.8926175	0	0
1005684	2	0.4772099	1	1
1005685	1	0.8471816	3	1
1005686	2	0.9457731	0	0
1005687	1	0.8380845	0	0
1005688	1	0.9359804	0	0
1005689	2	0.6755275	3	1
1005690	2	0.0687678	8	1
1005691	2	0.5482672	2	1
1005692	1	0.7451044	2	1
1005693	2	0.2027784	0	1
1005694	2	0.9215279	0	0
1005695	2	0.380564	0	1
1005696	2	0.9395485	0	0
1005697	2	0.5655465	0	1
1005698	2	0.7346398	2	1
1005699	2	0.6743881	0	1
1005700	1	0.8071992	0	0
1005701	2	0.7032168	5	1
1005701	1	0.7032168	4	1
1005702	1	0.9505225	0	0
1005703	2	0.9512951	0	0
1005704	2	0.7010806	0	0
1005705	1	0.9706023	0	0
1005706	2	0.9766437	0	0
1005707	1	0.9718675	0	0
1005708	1	0.9237913	0	0
1005709	2	0.8987589	0	0
1005710	1	0.9180547	0	1
1005711	2	0.8006195	0	0
1005712	1	0.3618611	0	1
1005712	2	0.4909714	1	1
1005713	1	0.8926175	0	0
1005714	2	0.599822	0	1
1005715	1	0.5621231	0	1
1005716	1	0.9724548	0	0

Patient ID	Side	Propensity score	Number of tranfusions	Matched
1005717	2	0.957775	0	0
1005718	1	0.1675961	2	1
1005719	2	0.8781879	0	0
1005720	2	0.4754415	0	1
1005721	1	0.9653663	0	0
1005722	1	0.3522707	2	0
1005722	2	0.5062472	2	1
1005723	2	0.4254257	0	1
1005724	2	0.9166842	0	0
1005725	2	0.7264057	0	0
1005726	1	0.9527564	0	0
1005727	2	0.8151238	0	0
1005728	1	0.1887592	1	0
1005729	1	0.5769122	0	1
1005730	2	0.8986861	0	1
1005731	1	0.7724354	2	1
1005732	2	0.9163173	0	0
1005733	2	0.321832	2	1
1005734	1	0.8464121	0	1
1005735	2	0.0592548	2	0
1005736	2	0.7191814	0	0
1005737	2	0.1067472	3	0
1005738	1	0.4515805	0	1
1005739	1	0.8199402	0	0
1005740	1	0.7551894	0	1
1005741	2	0.9482724	0	0
1005742	1	0.1268756	4	0
1005743	2	0.9330049	0	0
1005744	1	0.1642707	0	1
1005745	2	0.6368605	4	1
1005746	1	0.6761712	4	1
1005747	2	0.8143077	0	0
1005748	1	0.6006756	3	1
1005748	2	0.7751945	0	0
1005749	1	0.5408182	4	1
1005749	2	0.3574027	4	1
1005750	2	0.9827961	0	0
1005751	2	0.2204023	4	0
1005752	1	0.3946473	1	1

Patient ID	Side	Propensity score	Number of tranfusions	Matched
1005753	2	0.742346	0	1
1005754	1	0.5057742	0	1
1005755	1	0.678968	0	1
1005756	1	0.8767222	4	1
1005757	2	0.9073451	0	0
1005758	2	0.9186629	0	0
1005759	1	0.8684553	0	0
1005760	2	0.8700188	0	0
1005761	1	0.9197193	0	0
1005762	1	0.678968	0	0
1005763	2	0.9639877	0	0
1005764	2	0.0532364	8	0
1005765	1	0.9402091	2	1
1005766	1	0.9883079	0	0
1005767	1	0.6846067	0	1
1005768	1	0.5152629	0	1
1005769	1	0.8926175	0	0
1005770	2	0.6822933	0	0
1005771	1	0.7751945	0	1
1005772	1	0.8410779	0	0
1005773	1	0.7236026	0	0
1005773	2	0.7609577	0	0
1005774	2	0.7992524	1	1
1005775	2	0.4164292	2	1
1005776	2	0.9342051	0	1
1005777	2	0.9068288	0	0
1005778	1	0.7128351	0	1
1005779	2	0.8375368	2	1
1005780	1	0.9743555	0	0
1005781	1	0.7010806	0	1
1005782	1	0.8410779	0	1
1005783	2	0.3387008	2	0
1005784	2	0.9163173	0	0
1005785	2	0.56959	2	1
1005785	1	0.56959	2	1
1005786	2	0.5602598	0	0
1005787	1	0.622981	0	1
1005788	2	0.9835454	0	0
1005789	1	0.1915684	2	0

Patient ID	Side	Propensity score	Number of tranfusions	Matched
1005790	1	0.4807361	2	1
1005791	1	0.3474476	3	0
1005792	1	0.7412045	0	1
1005793	2	0.9186629	0	0
1005793	1	0.9186629	0	0
1005794	1	0.6215086	2	1
1005795	2	0.8070162	0	0
1005796	2	0.4980487	0	1
1005797	1	0.9471208	0	0
1005798	1	0.380564	2	1
1005799	2	0.6693853	2	1
1005800	2	0.2550914	0	1
1005801	2	0.4592113	0	1
1005802	2	0.9735961	0	0
1005803	2	0.5687177	4	1
1005804	2	0.7699598	0	0
1005805	2	0.9128413	0	0
1005806	2	0.1506212	7	0
1005806	1	0.5221845	2	1
1005807	2	0.4983625	0	1
1005808	2	0.8569584	0	1
1005808	1	0.9186629	0	0
1005809	1	0.5005986	5	1
1005810	1	0.6465287	0	1
1005811	1	0.9040454	0	0
1005811	2	0.9040454	0	0
1005812	1	0.4375402	0	1
1005813	1	0.8622236	0	0
1005814	1	0.8987589	0	0
1005815	1	0.7668959	4	1
1005816	2	0.378616	0	1
1005817	1	0.321832	1	0
1005818	2	0.2801864	2	1
1005819	2	0.8143077	0	1
1005820	2	0.9127291	0	0
1005821	2	0.8053637	0	0
1005822	2	0.381759	2	0
1005822	1	0.381759	0	1
1005823	1	0.8492618	0	0

Patient ID	Side	Propensity score	Number of tranfusions	Matched
1005824	2	0.8462635	0	0
1005824	1	0.8720679	0	0
1005825	2	0.2711451	3	0
1005826	2	0.1891314	3	0
1005827	2	0.5211484	0	1
1005828	1	0.5251476	0	1
1005829	1	0.8464121	2	1
1005830	2	0.8342246	0	0
1005831	2	0.9197193	0	0
1005832	2	0.8626183	0	0
1005833	2	0.7310801	0	1
1005834	2	0.473773	0	1
1005835	1	0.3800545	0	1
1005836	1	0.7317822	3	1
1005836	2	0.8955071	0	1
1005837	2	0.3361404	4	0
1005838	1	0.8464121	0	0
1005839	1	0.6695935	0	1
1005840	1	0.034543	7	0
1005841	1	0.7701729	0	0
1005842	2	0.9290496	0	1
1005843	1	0.8909839	0	0
1005844	2	0.9191106	0	0
1005845	2	0.9546245	0	0
1005846	2	0.321832	2	1
1005847	1	0.6395683	1	1
1005848	2	0.3090635	5	0
1005849	2	0.8733664	0	1
1005850	2	0.5958125	2	1
1005851	1	0.475834	2	1
1005851	2	0.3250186	4	1
1005852	2	0.6805081	0	1
1005853	1	0.1885288	4	1
1005854	2	0.6103809	0	1
1005855	2	0.7904238	4	1
1005855	1	0.7904238	1	1
1005856	1	0.5920472	0	1
1005857	2	0.6299819	0	0
1005858	2	0.1791882	0	1

Patient ID	Side	Propensity score	Number of tranfusions	Matched
1005859	2	0.8684553	0	0
1005860	1	0.5649148	2	1
1005861	2	0.7807217	0	0
1005862	2	0.407293	0	1
1005863	1	0.8091414	1	1
1005864	1	0.6698383	0	1
1005865	1	0.4735617	4	1
1005866	1	0.6443419	1	1
1005867	1	0.9119742	0	0
1005868	1	0.8312791	0	0
1005869	2	0.8556398	0	0
1005870	1	0.4475346	3	1
1005871	2	0.2108666	1	0
1005872	1	0.3961032	6	1
1005873	2	0.8149367	0	0
1005874	1	0.5307699	4	1
1005875	1	0.85848	0	1
1005875	2	0.9637995	0	0
1005876	2	0.2826716	14	0
1005877	2	0.5057742	0	1
1005878	1	0.9222556	0	0
1005879	1	0.7205255	0	1
1005880	2	0.8830779	0	1
1005881	2	0.6456687	0	0
1005882	1	0.5449822	0	1
1005883	2	0.5025039	2	1
1005884	2	0.3250186	1	1
1005885	2	0.8143077	1	1
1005886	1	0.8061834	2	1
1005887	1	0.0567909	2	0
1005888	1	0.948741	0	0
1005889	1	0.653267	0	0
1005890	1	0.1074888	3	0
1005891	1	0.8319608	1	1
1005892	2	0.7819624	0	0
1005893	2	0.810253	0	0
1005894	1	0.4614544	0	1
1005895	1	0.1460613	5	0
1005896	1	0.0948138	2	0

Patient ID	Side	Propensity score	Number of tranfusions	Matched
1005897	2	0.843828	0	1
1005898	2	0.6210378	0	1
1005899	2	0.9121083	0	0
1005900	2	0.7451044	0	1
1005901	1	0.5575569	2	1
1005902	1	0.1111439	3	0
1005903	2	0.6822033	0	1
1005904	2	0.4375777	2	1
1005904	1	0.6449332	0	1
1005905	2	0.8460801	0	0
1005906	1	0.7853797	0	1
1005907	2	0.9380204	0	0
1005908	1	0.6506863	0	1
1005909	1	0.0306747	97	0
1005910	1	0.2591388	4	0
1005911	2	0.6006756	0	1
1005912	2	0.8380845	0	1
1005913	1	0.6565705	3	1
1005914	1	0.7838778	0	0
1005914	2	0.8151675	0	1
1005915	1	0.0617476	2	0
1005916	1	0.9128584	0	0
1005916	2	0.9128584	0	0
1005917	2	0.9086713	0	0
1005918	1	0.5122697	0	1
1005919	1	0.7849514	2	1
1005920	1	0.7059127	3	1
1005921	2	0.8242614	0	0
1005921	1	0.9163173	0	0
1005922	2	0.948741	0	1
1005923	1	0.693725	0	0
1005924	2	0.5576199	0	1
1005925	1	0.2972059	0	1
1005926	1	0.8765468	0	1
1005927	1	0.3070275	0	1
1005928	1	0.8152938	1	1
1005929	1	0.6248119	0	1
1005930	2	0.1743616	0	1
1005931	1	0.3628151	0	1

Patient ID	Side	Propensity score	Number of tranfusions	Matched
1005932	1	0.5234918	2	1
1005933	1	0.9145093	0	0
1005934	1	0.8604233	0	1
1005935	2	0.113951	3	0
1005936	2	0.7258901	0	1
1005937	1	0.9342051	0	0
1005938	1	0.3770483	1	0
1005939	2	0.422429	4	1
1005940	1	0.138256	8	0
1005940	2	0.2116794	4	1
1005941	2	0.7470569	0	1
1005942	1	0.6577749	0	0
1005943	2	0.2315262	4	0
1005944	2	0.9530739	0	0
1005945	2	0.7001598	0	0
1005946	1	0.4452966	2	1
1005946	2	0.9095091	4	1
1005947	2	0.6175179	2	1
1005948	1	0.9348254	0	0
1005948	2	0.9467011	0	0
1005949	2	0.8801035	0	0
1005950	1	0.7656533	0	1
1005951	1	0.6411311	0	1
1005952	1	0.4006431	9	1
1005953	2	0.9568595	0	0
1005954	1	0.6370063	2	1
1005955	2	0.8492533	0	0
1005956	2	0.8610562	0	1
1005957	1	0.9366489	0	0
1005957	2	0.9366489	0	0
1005958	2	0.8267231	0	0
1005959	1	0.426527	2	1
1005960	2	0.4570379	0	1
1005961	1	0.0884485	0	1
1005962	2	0.8287051	0	0
1005963	1	0.5740564	4	1
1005964	1	0.6319291	2	1
1005965	2	0.2228526	5	1
1005966	1	0.3062646	2	0

Patient ID	Side	Propensity score	Number of tranfusions	Matched
1005967	1	0.6210378	0	1
1005968	2	0.8720679	0	0
1005969	2	0.6992193	0	1
1005970	2	0.6062345	2	1
1005971	1	0.8590341	2	1
1005971	2	0.8590341	2	1
1005972	2	0.4584335	2	1
1005973	2	0.87418	2	1
1005974	1	0.8065736	0	0
1005974	2	0.8065736	0	0
1005975	2	0.837696	0	1
1005976	1	0.9348254	2	1
1005977	2	0.5416342	4	1
1005978	1	0.5307699	2	1
1005979	1	0.9195277	0	1
1005980	2	0.4144451	0	1
1005981	1	0.6544893	2	1
1005982	2	0.3749073	2	1
1005983	1	0.7007701	0	0
1005984	2	0.6909467	1	1
1005985	2	0.9684105	0	0
1005986	1	0.6585441	0	1
1005986	2	0.714073	0	0
1005987	1	0.9568595	0	0
1005988	2	0.8071553	0	0
1005989	1	0.9598494	0	0
1005990	2	0.8576828	0	1
1005991	1	0.6210378	0	1
1005992	2	0.7060583	0	1
1005993	1	0.9555191	1	1
1005994	2	0.6931869	4	1
1005995	2	0.5312783	0	1
1005996	2	0.1011862	0	1
1005997	2	0.0822351	0	1
1005998	1	0.622981	2	1
1005999	2	0.6834791	2	1
1006000	2	0.9568595	0	0
1006000	1	0.9568595	0	0
1006001	1	0.4379569	3	1

Patient ID	Side	Propensity score	Number of tranfusions	Matched
1006002	1	0.9120077	0	0
1006002	2	0.9555191	0	0
1006003	1	0.9555191	0	0
1006004	1	0.8846375	0	1
1006005	2	0.7836873	0	0
1006006	1	0.941524	0	0
1006007	2	0.2598204	0	1
1006008	1	0.9712601	0	0
1006009	1	0.6909467	2	1
1006010	1	0.3961032	5	1
1006011	2	0.7565391	0	0
1006012	1	0.9472872	0	0
1006013	1	0.7004812	0	1
1006014	2	0.8170872	0	0
1006015	2	0.1635913	3	0
1006016	2	0.5472813	4	1
1006017	2	0.2541205	0	1
1006017	1	0.4528818	0	1
1006018	1	0.7974401	0	1
1006019	1	0.748557	0	0
1006020	1	0.7451837	0	1
1006021	1	0.0698281	4	0
1006022	1	0.6736824	0	1
1006023	1	0.7478824	0	0
1006024	2	0.020908	29	0
1006025	1	0.6951561	2	1
1006026	2	0.3504041	2	0
1006027	2	0.911543	2	1
1006028	1	0.5116775	2	1
1006029	2	0.8410779	0	0
1006030	1	0.7454145	2	1
1006031	2	0.7115915	2	1
1006032	1	0.9070476	0	0
1006033	2	0.1232652	0	1
1006034	2	0.5543528	0	0
1006035	1	0.4417876	2	0
1006036	2	0.9568595	0	0
1006037	1	0.8926175	0	0
1006038	1	0.9119742	0	0

Patient ID	Side	Propensity score	Number of tranfusions	Matched
1006039	2	0.8267231	0	0
1006039	1	0.8182057	0	0
1006040	1	0.7838778	0	0
1006041	2	0.9297823	0	0
1006042	2	0.8987589	2	1
1006043	2	0.0815882	1	1
1006044	1	0.3065288	0	1
1006045	1	0.2067997	2	1
1006046	1	0.8652821	0	0
1006047	1	0.8540021	0	1
1006048	1	0.9264762	0	0
1006049	2	0.9457731	0	0
1006050	1	0.6822033	0	0
1006050	2	0.8838318	0	0
1006051	2	0.8033172	0	0
1006052	1	0.8859565	0	0
1006053	1	0.3451464	4	1
1006054	1	0.9573394	0	0
1006055	1	0.6688298	0	1
1006056	2	0.6820516	0	1
1006057	1	0.8622236	2	1
1006058	1	0.9222556	0	0
1006059	1	0.4353576	0	1
1006060	1	0.3028421	0	1
1006061	1	0.4349635	0	1
1006062	1	0.9163947	0	0
1006063	1	0.6343521	0	0
1006064	1	0.8926175	0	0
1006065	1	0.0659134	7	0
1006066	1	0.2024319	4	0
1006067	2	0.5493338	3	1
1006067	1	0.4796653	0	1
1006068	1	0.5745877	0	0
1006069	2	0.8624214	0	0
1006070	2	0.0834714	1	0
1006071	1	0.9195277	0	0
1006072	1	0.2550914	4	1
1006073	1	0.914542	0	0
1006074	2	0.6973977	0	1

Patient ID	Side	Propensity score	Number of tranfusions	Matched
1006075	2	0.7511338	10	1
1006076	2	0.8703418	2	1
1006076	1	0.8703418	0	0
1006077	1	0.969153	0	0
1006077	2	0.9486418	0	0
1006078	1	0.7881868	6	1
1006079	1	0.2330678	2	1
1006080	1	0.8363182	0	0
1006081	1	0.5965624	2	1
1006082	1	0.7836873	0	0
1006083	1	0.1466258	3	0
1006084	1	0.2992102	0	1
1006085	2	0.3666436	2	1
1006086	1	0.8453464	0	0
1006087	2	0.756538	2	1
1006087	1	0.4671674	3	1
1006088	1	0.8392869	0	0
1006089	1	0.3250186	7	1
1006089	2	0.3250186	3	1
1006090	1	0.5540145	0	1
1006091	2	0.2924066	2	0
1006092	2	0.2150401	0	1
1006093	1	0.5575969	0	1
1006093	2	0.6774199	0	1
1006094	2	0.639349	0	1
1006095	1	0.2706053	0	1
1006095	2	0.5131808	0	1
1006096	2	0.9099573	0	0
1006097	2	0.7331558	2	1
1006098	1	0.5585162	4	1
1006099	1	0.4915451	2	1
1006100	2	0.8659212	0	0
1006100	1	0.8503392	0	0
1006101	2	0.8035296	0	1
1006102	2	0.2804494	0	1
1006103	1	0.678968	0	1
1006104	1	0.8308958	0	0
1006105	2	0.364077	1	0
1006106	1	0.9827961	0	0

Patient ID	Side	Propensity score	Number of tranfusions	Matched
1006107	1	0.8242864	0	0
1006108	2	0.6554563	0	1
1006108	1	0.4754649	0	1
1006109	1	0.7861992	0	0
1006110	2	0.8838318	2	1
1006111	1	0.7877594	0	1
1006112	2	0.8477782	0	0
1006113	1	0.882295	0	0
1006114	2	0.7994878	0	1
1006115	1	0.4190377	2	0
1006116	2	0.733989	0	0
1006117	1	0.9146721	2	1
1006117	2	0.87418	2	1
1006118	2	0.5052486	0	1
1006119	2	0.9281372	0	0
1006120	1	0.9380204	0	1
1006121	1	0.5371472	2	1
1006122	1	0.8908884	0	0
1006123	2	0.8427618	0	0
1006124	2	0.6370454	0	0
1006125	2	0.9339128	0	0
1006126	2	0.7835842	2	1
1006127	1	0.9380204	0	0
1006128	1	0.7064988	0	1
1006129	1	0.7459386	0	1
1006129	2	0.6109409	0	1
1006130	1	0.7829055	0	0
1006131	2	0.3501185	2	0
1006132	1	0.2862857	2	0
1006133	2	0.1838996	0	1
1006134	1	0.4965861	0	1
1006135	1	0.9086713	0	1
1006136	1	0.8838318	0	0
1006136	2	0.9546245	0	0
1006137	2	0.4846757	6	1
1006137	1	0.6621338	0	0
1006138	1	0.7610734	0	0
1006139	1	0.8143077	0	0
1006140	2	0.882295	0	0

Patient ID	Side	Propensity score	Number of tranfusions	Matched
1006141	2	0.1136335	2	0
1006142	2	0.9827961	0	0
1006143	2	0.7103589	0	1
1006144	1	0.2992102	3	0
1006145	1	0.2457842	2	1
1006146	1	0.8652821	0	1
1006146	2	0.8622236	0	0
1006147	2	0.2714662	3	0
1006148	1	0.380564	3	0
1006149	1	0.1924715	5	0
1006150	1	0.1741835	4	0
1006151	2	0.9571506	0	0
1006151	1	0.9862315	0	0
1006152	1	0.7996714	0	1
1006152	2	0.8827056	0	0
1006153	2	0.3711177	4	1
1006153	1	0.1357266	3	0
1006154	1	0.7138674	0	0
1006155	1	0.8410779	0	0
1006156	1	0.678968	2	1
1006157	1	0.242314	4	0
1006158	1	0.751755	0	0
1006159	2	0.4419562	0	1
1006160	2	0.1946929	0	1
1006161	2	0.7252052	0	0
1006162	2	0.7994878	0	1
1006163	2	0.410127	2	1
1006164	1	0.7897282	2	1
1006165	1	0.6687235	0	1
1006166	1	0.8380845	0	0
1006167	2	0.5014506	2	1
1006168	1	0.188642	9	0
1006169	2	0.7987674	0	1
1006170	1	0.4539692	0	1
1006170	2	0.8300317	0	0
1006171	1	0.9040454	0	0
1006172	1	0.9365586	0	0
1006173	2	0.7485968	0	0
1006174	1	0.7097606	0	1

Patient ID	Side	Propensity score	Number of tranfusions	Matched
1006175	1	0.6982953	0	0
1006176	1	0.3048106	1	0
1006177	1	0.9186629	0	0
1006178	1	0.9653663	0	0
1006179	2	0.2330678	0	1
1006179	1	0.340764	0	1
1006180	2	0.8926175	0	0
1006181	1	0.8114685	0	0
1006181	2	0.9086713	0	0
1006182	1	0.5307699	0	1
1006183	2	0.9179326	0	0
1006183	1	0.8149367	0	0
1006184	1	0.559449	0	1
1006185	2	0.9555191	2	1
1006186	2	0.678968	0	0
1006187	2	0.9417866	0	1
1006188	2	0.9471208	0	0
1006189	2	0.8923432	0	0
1006190	2	0.9775355	0	0
1006191	1	0.810253	0	0
1006192	2	0.7709754	0	0
1006193	1	0.7087258	14	1
1006194	1	0.8340393	0	0
1006195	1	0.5379262	0	1
1006196	2	0.0441993	8	0
1006197	1	0.9395485	0	1
1006198	2	0.3009225	0	1
1006199	2	0.0950119	1	0
1006200	1	0.739302	0	0
1006201	1	0.9781832	0	0
1006202	1	0.0951333	0	1
1006203	2	0.9151895	0	0
1006204	2	0.965012	0	0
1006205	2	0.8182057	0	0
1006205	1	0.559449	0	1
1006206	2	0.5482672	3	1
1006207	2	0.8349239	2	1
1006207	1	0.9050807	2	1
1006208	2	0.4431884	1	0

Patient ID	Side	Propensity score	Number of tranfusions	Matched
1006209	1	0.3045899	2	0
1006210	1	0.9145093	0	0
1006211	1	0.6056686	0	1
1006212	1	0.678968	3	1
1006213	2	0.974966	0	0
1006213	1	0.9623802	0	0
1006214	1	0.9372068	0	0
1006214	2	0.963065	0	0
1006215	1	0.0524343	4	0
1006216	2	0.7838286	0	0
1006217	1	0.8464121	4	1
1006218	1	0.0554122	4	0
1006219	2	0.7362633	0	0
1006220	1	0.8182057	0	1
1006221	2	0.6361699	0	0
1006222	2	0.6349469	0	0
1006223	1	0.3707763	1	1
1006224	1	0.6790886	3	1
1006224	2	0.9436676	0	0
1006225	2	0.7699598	0	0
1006226	1	0.9277478	0	0
1006227	1	0.8531178	0	1
1006228	2	0.0667093	4	0
1006229	1	0.7639143	1	1
1006230	1	0.6682116	2	1
1006231	2	0.6909467	0	0
1006232	1	0.7010806	5	1
1006233	1	0.8186172	0	0
1006234	1	0.0986374	6	0
1006235	2	0.8486877	0	0
1006236	2	0.8143077	0	0
1006237	1	0.7122629	0	0
1006238	2	0.5623317	0	1
1006239	2	0.9568595	0	0
1006239	1	0.9568595	0	0
1006240	1	0.9603292	0	0
1006241	1	0.9380204	0	1
1006242	2	0.9197193	0	0
1006243	1	0.6743881	0	1

Patient ID	Side	Propensity score	Number of tranfusions	Matched
1006244	1	0.9781832	0	0
1006245	2	0.714073	2	1
1006246	1	0.4316847	0	1
1006247	2	0.8963693	0	0
1006248	1	0.9639877	0	0
1006249	2	0.1598218	2	0
1006250	1	0.6631602	2	1
1006251	1	0.56959	0	1
1006252	2	0.4802569	2	1
1006252	1	0.2067997	2	0
1006253	1	0.242314	2	0
1006254	2	0.2862372	2	1
1006255	1	0.9040454	0	0
1006256	1	0.5170605	0	1
1006257	1	0.2025311	2	0
1006258	2	0.8067023	0	0
1006259	2	0.9743555	2	1
1006260	1	0.1449856	7	0
1006261	1	0.7266501	0	0
1006262	2	0.5745877	0	1
1006263	2	0.7853797	0	0
1006264	2	0.6179632	2	1
1006265	2	0.6307332	17	1
1006266	2	0.3798259	0	1
1006266	1	0.3798259	2	0
1006267	1	0.5740564	0	1
1006268	1	0.9121083	0	0
1006269	2	0.9557483	0	1
1006270	1	0.490862	0	1
1006270	2	0.490862	2	1
1006271	2	0.9611736	0	0
1006272	2	0.9119742	2	1
1006273	2	0.9278036	0	0
1006274	2	0.552885	0	0
1006275	1	0.702928	0	0
1006276	1	0.3517092	0	1
1006277	1	0.9634501	0	0
1006278	1	0.5557157	0	1
1006279	1	0.4722031	3	1

Patient ID	Side	Propensity score	Number of tranfusions	Matched
1006279	2	0.4415582	2	0
1006280	1	0.6210378	2	1
1006281	1	0.9166842	0	0
1006281	2	0.9743555	0	0
1006282	1	0.1886882	4	0
1006283	1	0.1873399	2	0
1006284	1	0.6672447	0	1
1006284	2	0.6672447	2	1
1006285	1	0.3028421	0	1
1006286	2	0.7941224	0	0
1006287	1	0.2525808	0	1
1006288	2	0.162369	2	0
1006289	1	0.8684553	0	0
1006290	2	0.2550914	4	0
1006291	1	0.5460748	0	1
1006292	2	0.9145093	0	0
1006293	2	0.8963693	0	0
1006294	2	0.3637847	0	1
1006295	2	0.9774165	0	0
1006296	1	0.3008999	0	1
1006296	2	0.9219838	0	0
1006297	1	0.9381146	0	0
1006298	1	0.9119742	0	0
1006299	1	0.6292635	0	1
1006300	2	0.8569584	0	0
1006301	1	0.9493857	0	0
1006302	2	0.7593779	2	1
1006303	2	0.3952608	0	1
1006304	2	0.1267193	5	0
1006305	1	0.7451044	0	0
1006306	1	0.0699262	5	0
1006307	2	0.3998955	2	1
1006308	2	0.8380845	0	0
1006309	2	0.9363302	0	0
1006310	2	0.8908782	0	0
1006311	1	0.6797838	2	1
1006312	2	0.7151394	0	0
1006313	2	0.1267823	1	0
1006314	1	0.8311099	0	0

Patient ID	Side	Propensity score	Number of tranfusions	Matched
1006315	2	0.6370454	0	0
1006316	1	0.3994056	1	0
1006317	2	0.380564	2	0
1006318	2	0.9068288	0	0
1006319	2	0.7451044	0	0
1006319	1	0.678968	0	0
1006320	1	0.8215458	0	0
1006321	1	0.4763374	0	1
1006322	1	0.8103819	0	0
1006323	2	0.3082673	4	1
1006324	1	0.9694243	0	0
1006325	1	0.7010806	0	0
1006326	2	0.622145	0	1
1006327	2	0.4256155	3	1
1006328	1	0.7451044	0	1
1006329	2	0.9276642	0	0
1006330	2	0.0503414	14	0
1006331	1	0.1583994	6	1
1006332	1	0.5307699	2	1
1006333	1	0.8726236	0	1
1006334	2	0.81959	0	0
1006335	1	0.9527564	0	0
1006336	2	0.2550914	2	0
1006337	2	0.9395485	2	1
1006338	1	0.9568595	0	0
1006339	1	0.311248	0	1
1006340	1	0.7010806	0	0
1006341	2	0.5922712	1	1
1006342	2	0.6723137	2	1
1006342	1	0.9555547	0	0
1006343	2	0.2807186	2	0
1006344	2	0.903371	0	0
1006344	1	0.887552	0	1
1006345	1	0.8751926	0	0
1006346	2	0.733567	4	1
1006347	2	0.4037822	1	1
1006348	1	0.8851865	0	0
1006348	2	0.9637995	0	0
1006349	2	0.2941618	0	1

Patient ID	Side	Propensity score	Number of tranfusions	Matched
1006350	2	0.3315825	0	1
1006351	2	0.7858006	0	1
1006352	1	0.9775355	0	0
1006353	1	0.8021703	0	0
1006354	2	0.8372281	0	0
1006355	2	0.8380845	0	0
1006356	1	0.6820516	0	0
1006357	2	0.9639877	0	0
1006358	1	0.8143077	0	0
1006359	2	0.9000514	0	0
1006360	1	0.8380845	0	0
1006361	2	0.4144451	0	1
1006361	1	0.622981	0	1
1006362	2	0.5551746	2	1
1006363	2	0.5422639	0	1
1006364	1	0.6850716	2	1
1006365	1	0.2199693	2	0
1006366	1	0.6842546	0	0
1006367	2	0.2486238	2	0
1006367	1	0.3601328	0	1
1006368	1	0.659002	0	0
1006369	1	0.475834	0	1
1006370	2	0.1223756	2	0
1006371	2	0.8944559	2	1
1006372	2	0.7032189	0	1
1006373	2	0.3471566	3	0
1006374	2	0.5460748	2	1
1006375	2	0.0389533	9	0
1006376	2	0.3844546	0	1
1006377	2	0.7717354	0	1
1006378	1	0.0347258	5	0
1006379	2	0.3028421	0	1
1006380	2	0.5438708	0	0
1006381	1	0.3236385	2	1
1006382	2	0.8763473	0	0
1006383	2	0.2768551	1	1
1006384	2	0.481933	1	1
1006385	1	0.5307699	0	1
1006386	1	0.4257735	0	1

Patient ID	Side	Propensity score	Number of tranfusions	Matched
1006387	1	0.3550394	5	1
1006388	2	0.2550914	3	1
1006389	2	0.5255339	0	1
1006390	1	0.8464121	0	0
1006391	1	0.894368	0	1
1006392	2	0.7019633	0	0
1006393	1	0.5433206	2	1
1006394	1	0.6565705	3	1
1006395	2	0.7974147	0	1
1006396	2	0.432904	2	0
1006396	1	0.4329454	2	0
1006397	2	0.2658993	1	0
1006398	1	0.2091801	2	0
1006398	2	0.4458629	3	1
1006399	1	0.7999699	0	0
1006400	2	0.7240763	0	0
1006401	2	0.6271352	0	1
1006402	2	0.9557483	0	0
1006403	1	0.5762173	4	1
1006404	1	0.7392867	0	1
1006405	1	0.9472872	0	0
1006406	2	0.9186629	0	0
1006407	1	0.8484094	0	0
1006407	2	0.8450363	0	1
1006408	2	0.7211949	0	1
1006409	2	0.0447451	3	0
1006410	1	0.5422639	2	1
1006411	1	0.7010806	0	0
1006412	2	0.9718675	0	0
1006413	2	0.5057742	0	1
1006414	1	0.9395485	0	0
1006415	2	0.632993	2	1
1006416	2	0.3904005	0	1
1006417	1	0.9364763	0	0
1006418	1	0.2197414	1	0
1006419	2	0.6945592	0	1
1006420	1	0.9377239	0	0
1006421	1	0.8684553	0	0
1006422	1	0.9796864	0	0

Patient ID	Side	Propensity score	Number of tranfusions	Matched
1006423	2	0.9684105	0	0
1006424	2	0.2645873	2	0
1006425	1	0.693725	0	0
1006426	1	0.8143077	2	1
1006427	1	0.8531178	0	0
1006428	1	0.9366489	0	0
1006429	2	0.6624998	0	1
1006430	1	0.7280526	0	1
1006431	1	0.1886882	2	0
1006432	1	0.9652634	0	0
1006433	1	0.9653663	0	0
1006434	2	0.710155	2	1
1006435	1	0.3246864	0	1
1006436	2	0.9366489	0	0
1006437	2	0.9269953	0	0
1006438	1	0.51449	2	1
1006439	2	0.6696718	0	1
1006440	2	0.2109563	6	0
1006441	2	0.8381147	0	0
1006442	1	0.9395485	0	0
1006443	2	0.3907733	0	1
1006444	2	0.5025039	1	1
1006445	2	0.9684105	0	0
1006446	2	0.9835454	0	0
1006447	2	0.9735961	0	0
1006447	1	0.9634501	0	0
1006448	1	0.7994878	0	0
1006449	1	0.8615083	0	0
1006450	2	0.7994878	2	1
1006451	2	0.7097598	2	1
1006452	1	0.8983983	0	0
1006452	2	0.9163173	0	0
1006453	2	0.8590341	0	0
1006454	2	0.5202207	2	1
1006455	1	0.6349469	2	1
1006456	2	0.9639877	0	0
1006457	2	0.7308096	0	1
1006458	2	0.8143077	0	0
1006459	2	0.6780845	0	1

Patient ID	Side	Propensity score	Number of tranfusions	Matched
1006460	2	0.5808195	0	1
1006461	1	0.9653663	0	0
1006462	2	0.88625	0	0
1006463	1	0.6664204	0	1
1006464	1	0.5482672	2	1
1006465	1	0.1355864	2	1
1006466	1	0.9639877	0	0
1006467	1	0.7994878	0	0
1006468	2	0.5585162	0	1
1006469	1	0.143682	0	1
1006470	2	0.6832323	0	1
1006471	2	0.6586223	1	1
1006472	1	0.475834	0	1
1006473	1	0.6021064	0	1
1006474	1	0.3158548	0	1
1006474	2	0.6343521	0	0
1006475	2	0.7138674	5	1
1006476	2	0.8199402	0	1
1006477	1	0.9457731	0	0
1006478	2	0.8590341	0	1
1006479	1	0.0147431	4	0
1006480	2	0.8512175	0	0
1006481	1	0.948741	0	1
1006482	2	0.9467011	0	0
1006483	1	0.5704284	1	1
1006484	1	0.7479218	0	0
1006485	1	0.9550222	0	0
1006486	2	0.8464121	0	1
1006487	1	0.4399924	7	0
1006488	1	0.4666292	0	1
1006489	2	0.2067997	0	1
1006489	1	0.3028421	0	1
1006490	1	0.2742545	1	1
1006491	2	0.4185981	5	1
1006492	1	0.887552	0	0
1006493	2	0.7418761	4	1
1006494	2	0.9311332	0	0
1006495	1	0.8199402	0	0
1006496	2	0.3305501	0	1

Patient ID	Side	Propensity score	Number of tranfusions	Matched
1006497	1	0.9313657	0	0
1006498	1	0.4000652	0	1
1006499	1	0.7035156	0	0
1006500	1	0.8002475	0	0
1006501	1	0.9706023	0	0
1006502	1	0.5482672	2	1
1006503	1	0.2745754	0	1
1006503	2	0.5135	0	1
1006504	1	0.8298365	0	0
1006505	2	0.6842546	0	0
1006505	1	0.8848014	0	1
1006506	2	0.6167231	0	1
1006507	2	0.6729171	2	1
1006508	1	0.9339128	0	0
1006509	1	0.7992524	2	1
1006510	2	0.9380691	0	0
1006511	2	0.6021064	1	1
1006512	2	0.8569584	2	1
1006513	1	0.5308739	0	1
1006514	2	0.6260713	2	1
1006515	1	0.5796341	0	1
1006516	2	0.5998099	2	1
1006517	2	0.3908383	0	1
1006518	1	0.6544533	0	1
1006519	2	0.8242864	0	0
1006520	2	0.7807217	0	0
1006521	1	0.8812618	0	0
1006521	2	0.87502	0	0
1006522	1	0.9168845	0	0
1006523	2	0.1223756	2	0
1006524	1	0.7151394	4	1
1006525	1	0.9499555	0	0
1006526	1	0.8931346	0	0
1006527	2	0.6339748	2	1
1006528	1	0.9555191	0	0
1006528	2	0.9555191	0	0
1006529	2	0.730823	2	1
1006529	1	0.730823	2	1
1006530	1	0.9398549	0	0

Patient ID	Side	Propensity score	Number of tranfusions	Matched
1006531	1	0.3221141	3	0
1006532	2	0.8671572	0	0
1006533	1	0.8958698	0	0
1006534	2	0.6633168	6	1
1006535	2	0.882295	0	0
1006536	2	0.1808962	0	1
1006537	1	0.6441329	0	1
1006538	2	0.9568595	0	0
1006539	1	0.9380204	0	0
1006540	2	0.6845789	3	1
1006540	1	0.8036016	0	1
1006541	1	0.9372897	0	0
1006542	2	0.475834	4	1
1006543	1	0.8656695	0	0
1006544	2	0.9222176	0	0
1006545	1	0.3545529	0	1
1006546	2	0.2604478	0	1
1006547	1	0.9395485	0	0
1006548	2	0.4505445	0	1
1006549	2	0.8565502	2	1
1006550	1	0.8651406	0	0
1006551	1	0.8377627	0	0
1006552	1	0.9623802	0	0
1006553	2	0.9000514	0	0
1006554	1	0.796731	2	1
1006555	2	0.5057742	2	1
1006556	2	0.9520807	0	0
1006557	2	0.410127	4	1
1006558	1	0.1435748	3	0
1006559	2	0.5094025	0	1
1006560	2	0.9568595	0	0
1006561	2	0.8784045	2	1
1006561	1	0.899455	0	0
1006562	1	0.7139026	0	0
1006563	2	0.5957808	0	1
1006564	2	0.1277829	2	0
1006565	1	0.9482519	0	0
1006566	1	0.7377456	0	0
1006567	2	0.8610562	0	0

Patient ID	Side	Propensity score	Number of tranfusions	Matched
1006568	2	0.6576642	0	1
1006569	1	0.9471623	0	0
1006570	1	0.5119561	0	1
1006571	1	0.0884485	0	1
1006572	2	0.9366737	0	0
1006573	1	0.9527564	0	0
1006574	1	0.1008832	1	0
1006575	2	0.8287051	0	0
1006576	1	0.810253	0	0
1006577	1	0.7010806	2	1
1006578	2	0.4715458	0	1
1006579	1	0.7613885	2	1
1006580	1	0.4586092	1	1
1006581	2	0.3952807	4	1
1006582	2	0.3637847	0	1
1006583	1	0.6852827	2	1
1006584	2	0.5076168	6	1
1006584	1	0.6211852	3	1
1006585	1	0.7699598	0	0
1006586	1	0.1460613	2	0
1006587	2	0.2828303	0	1
1006588	1	0.8987589	0	0
1006588	2	0.7025956	0	0
1006589	2	0.9380204	0	0
1006590	2	0.9145093	0	0
1006591	1	0.4144451	0	1
1006592	1	0.8309514	0	1
1006593	2	0.9366489	0	0
1006594	1	0.7609577	0	0
1006595	2	0.4031475	3	0
1006596	1	0.1635525	17	1
1006597	2	0.5935675	0	1
1006597	1	0.8380845	3	1
1006598	2	0.8459021	0	0
1006599	2	0.833655	2	1
1006600	2	0.5357667	0	1
1006601	1	0.5025039	0	1
1006602	1	0.8234012	0	0
1006603	1	0.5207967	0	1

Patient ID	Side	Propensity score	Number of tranfusions	Matched
1006604	1	0.6179632	0	1
1006605	2	0.8170857	0	0
1006606	1	0.8293664	0	1
1006607	1	0.9637995	0	0
1006608	2	0.7861992	0	0
1006609	1	0.1267193	4	0
1006610	1	0.5106366	0	1
1006611	1	0.9581994	0	0
1006612	2	0.8583781	0	1
1006612	1	0.8583781	0	0
1006613	1	0.6539285	6	1
1006614	1	0.9380204	0	0
1006615	1	0.9718675	0	0
1006616	1	0.8892304	0	0
1006617	1	0.2024319	3	0
1006618	1	0.6723223	1	1
1006619	1	0.810253	0	0
1006620	1	0.817672	0	0
1006620	2	0.817672	0	0
1006621	1	0.8838318	0	0
1006622	2	0.9679403	3	1
1006623	1	0.5213943	0	1
1006624	2	0.6265182	0	1
1006625	2	0.7445205	0	0
1006626	1	0.0712174	11	0
1006627	1	0.467087	0	1
1006628	2	0.2363879	2	0
1006629	1	0.8159437	0	0
1006630	2	0.02241	18	0
1006631	2	0.7018522	0	0
1006632	1	0.8462212	0	0
1006633	1	0.559449	2	1
1006634	2	0.473773	2	1
1006635	1	0.8196841	0	1
1006636	1	0.8622153	0	1
1006637	2	0.9120077	2	1
1006638	1	0.8749832	0	0
1006639	2	0.1134464	2	0
1006640	1	0.9468601	0	0

Patient ID	Side	Propensity score	Number of tranfusions	Matched
1006641	1	0.678968	0	1
1006642	1	0.8720679	0	0
1006642	2	0.9398549	0	0
1006643	2	0.321832	0	1
1006644	1	0.8665466	0	0
1006645	1	0.8859565	0	0
1006646	1	0.2598204	6	1
1006646	2	0.2598204	2	1
1006647	2	0.4571578	0	1
1006648	2	0.882295	0	0
1006649	2	0.2658993	2	0
1006650	1	0.9653663	0	0
1006650	2	0.9653663	0	0
1006651	2	0.7836873	0	0
1006652	2	0.6506863	0	0
1006653	1	0.5575569	4	1
1006654	1	0.5839917	0	1
1006655	2	0.0273303	2	0
1006656	1	0.7840198	0	0
1006656	2	0.4913575	2	1
1006657	1	0.3602291	0	1
1006658	1	0.8937287	0	1
1006659	2	0.622981	0	1
1006659	1	0.467087	0	1
1006660	2	0.8980585	0	0
1006661	1	0.3751264	0	1
1006662	1	0.8924138	0	0
1006663	2	0.8604233	0	1
1006664	1	0.9277478	0	0
1006665	1	0.8372364	0	0
1006665	2	0.9065205	0	0
1006666	1	0.9395485	0	0
1006667	1	0.8782153	0	0
1006668	1	0.810253	0	0
1006669	2	0.6805081	0	1
1006670	1	0.9256307	0	0
1006671	2	0.9134292	0	0
1006672	2	0.9607372	4	1
1006673	2	0.2130473	8	0

Patient ID	Side	Propensity score	Number of tranfusions	Matched
1006674	1	0.8531178	0	0
1006675	2	0.6447928	9	1
1006676	1	0.8143077	0	1
1006677	2	0.168978	2	1
1006678	2	0.3501185	2	0
1006679	1	0.1460613	2	0
1006680	1	0.9680528	2	1
1006681	1	0.5307699	2	1
1006682	1	0.0626167	6	1
1006683	1	0.6971362	0	0
1006684	2	0.9424865	0	0
1006685	2	0.0089122	18	0
1006686	1	0.8027986	0	0
1006686	2	0.9624106	0	0
1006687	1	0.5904373	6	1
1006688	1	0.4774967	0	1
1006688	2	0.5329441	2	1
1006689	1	0.8410779	0	0
1006690	2	0.7838286	0	0
1006691	1	0.2468794	0	1
1006692	2	0.9243652	0	0
1006693	1	0.9197193	2	1
1006694	1	0.2137922	2	0
1006695	1	0.6631602	8	1
1006696	1	0.6179632	2	1
1006697	1	0.1183616	0	1
1006698	2	0.9005453	0	1
1006699	1	0.8103819	0	0
1006699	2	0.9380204	2	1
1006700	1	0.2637527	2	1
1006700	2	0.5737743	0	1
1006701	1	0.9050807	0	0
1006702	1	0.3282734	2	0
1006703	1	0.5478821	0	1
1006704	1	0.8326454	0	0
1006705	1	0.914542	0	1
1006706	1	0.9743555	0	0
1006707	1	0.8359879	0	0
1006708	1	0.621019	2	1

Patient ID	Side	Propensity score	Number of tranfusions	Matched
1006709	1	0.2550914	1	0
1006710	2	0.7025956	0	1
1006711	1	0.9512951	0	0
1006711	2	0.9512951	0	0
1006712	2	0.9256307	0	0
1006713	1	0.6374629	0	0
1006713	2	0.919453	0	0
1006714	1	0.9493857	0	1
1006715	2	0.5238844	0	1
1006716	2	0.8061576	3	1
1006717	2	0.9119742	2	1
1006718	1	0.8627645	0	0
1006719	2	0.3833727	2	1
1006720	2	0.2204541	4	0
1006721	1	0.9639877	0	0
1006722	2	0.1708543	2	0
1006723	2	0.56959	2	1
1006724	2	0.8182057	0	0
1006724	1	0.8926175	0	1
1006725	2	0.9079289	0	0
1006726	2	0.9639877	0	0
1006726	1	0.9639877	1	1
1006727	1	0.7785966	0	0
1006728	2	0.4733407	2	1
1006728	1	0.810253	0	0
1006729	1	0.321832	4	0
1006729	2	0.6618387	0	0
1006730	2	0.2350999	1	1
1006731	1	0.8584276	0	0
1006732	1	0.810253	0	1
1006733	2	0.9802742	0	0
1006734	2	0.182517	2	1
1006735	1	0.8462635	0	0
1006736	1	0.2829048	3	0
1006736	2	0.3556132	2	1
1006737	1	0.2550914	0	1
1006738	1	0.5057742	0	1
1006739	1	0.7413953	0	0
1006740	2	0.808008	0	0

Patient ID	Side	Propensity score	Number of tranfusions	Matched
1006741	2	0.8412643	0	0
1006742	2	0.9512951	0	0
1006743	2	0.9163173	0	0
1006744	1	0.4209728	1	1
1006745	2	0.9380204	0	0
1006746	2	0.1663991	0	1
1006747	1	0.7641478	4	1
1006748	2	0.7907302	0	0
1006749	2	0.7471596	0	0
1006749	1	0.6618387	2	1
1006750	1	0.9551294	0	0
1006751	1	0.8500797	2	1
1006752	2	0.755473	2	1
1006753	2	0.3065288	2	1
1006754	1	0.9637995	0	0
1006754	2	0.9555191	0	0
1006755	1	0.7010806	0	0
1006756	1	0.4410523	3	0
1006757	2	0.5329441	0	1
1006758	2	0.2035356	2	0
1006759	1	0.6498169	0	1
1006760	2	0.711776	0	0
1006761	2	0.3798259	0	1
1006762	1	0.5696323	0	1
1006763	2	0.5795027	6	1
1006764	2	0.8987589	0	0
1006765	1	0.9639877	0	0
1006766	2	0.9086713	0	0
1006767	2	0.2102932	3	0
1006768	1	0.3525196	4	0
1006769	1	0.7062283	0	1
1006770	1	0.774531	0	0
1006771	1	0.6723223	0	1
1006771	2	0.755473	0	1
1006772	2	0.5724973	2	1
1006773	2	0.6308935	2	1
1006774	1	0.3915087	1	0
1006775	2	0.7004812	0	1
1006776	1	0.8651406	0	0

Patient ID	Side	Propensity score	Number of tranfusions	Matched
1006777	1	0.7838778	1	1
1006778	1	0.4185981	2	1
1006779	1	0.8531178	0	0
1006780	2	0.5238844	1	1
1006781	1	0.8532444	2	1
1006782	2	0.8464121	0	0
1006783	1	0.3531793	1	1
1006784	2	0.4415582	2	1
1006785	1	0.6288589	2	1
1006786	2	0.2499274	0	1
1006787	1	0.8665093	0	0
1006788	1	0.8550696	2	1
1006789	1	0.7892495	0	0
1006790	1	0.2992102	2	1
1006791	1	0.4509937	2	1
1006792	2	0.1715548	18	0
1006793	1	0.9557483	0	0
1006794	2	0.9623818	0	0
1006795	1	0.3192709	2	0
1006796	1	0.8420542	0	1
1006797	2	0.81357	0	0
1006798	2	0.615287	0	1
1006799	1	0.7175289	0	0
1006800	2	0.5904373	2	1
1006801	2	0.7668433	0	1
1006802	1	0.3931496	2	0
1006803	2	0.1516064	3	0
1006804	1	0.6586223	0	1
1006805	1	0.85848	2	1
1006806	2	0.6757735	0	1
1006807	2	0.7834102	0	0
1006808	1	0.6506863	0	1
1006808	2	0.6506863	0	1
1006809	1	0.710155	0	1
1006810	2	0.7271796	3	1
1006811	2	0.7053579	4	1
1006812	2	0.9735961	0	0
1006813	1	0.9684361	0	0
1006814	2	0.7559605	2	1

Patient ID	Side	Propensity score	Number of tranfusions	Matched
1006815	2	0.9128413	0	0
1006816	1	0.8709951	0	0
1006817	2	0.8503392	0	0
1006818	2	0.8143077	0	0
1006819	1	0.7230118	0	0
1006820	1	0.8268883	0	0
1006821	1	0.8864646	0	0
1006822	2	0.7641478	0	0
1006823	2	0.9568595	0	0
1006824	2	0.9684105	0	0
1006825	2	0.8628989	0	0
1006826	2	0.8569584	0	0
1006826	1	0.8312791	0	0
1006827	2	0.0147431	6	0
1006828	2	0.8143077	0	0
1006829	1	0.7836873	0	1
1006830	1	0.8583781	0	1
1006831	1	0.9684105	0	0
1006832	2	0.8652821	1	1
1006833	1	0.9527564	0	0
1006834	1	0.6586223	0	0
1006835	1	0.9570311	0	0
1006836	2	0.6743881	2	1
1006837	1	0.3028421	2	1
1006838	1	0.8033172	0	0
1006838	2	0.8349239	0	0
1006839	2	0.8159437	0	1
1006840	2	0.559449	0	0
1006841	1	0.9219838	0	0
1006842	1	0.65763	0	0
1006843	2	0.7103589	0	0
1006844	1	0.2801864	2	0
1006845	1	0.2595311	0	1
1006846	1	0.8143077	0	0
1006847	2	0.8380845	0	0
1006848	1	0.2559329	0	1
1006849	2	0.1353537	6	0
1006850	2	0.8043698	0	1
1006851	1	0.5935675	0	1

Patient ID	Side	Propensity score	Number of tranfusions	Matched
1006852	1	0.1288039	2	0
1006853	2	0.8247183	0	0
1006854	2	0.685284	2	1
1006855	2	0.3007648	0	1
1006856	2	0.750596	0	0
1006857	2	0.2953252	2	1
1006858	2	0.7054591	3	1
1006859	1	0.5435919	0	1
1006860	1	0.9398549	0	0
1006861	2	0.526943	3	1
1006862	2	0.8963693	0	0
1006863	2	0.7567775	0	0
1006864	2	0.242314	3	0
1006865	1	0.4549305	2	1
1006866	1	0.7941224	0	1
1006867	1	0.678968	0	0
1006868	1	0.8887281	0	0
1006869	2	0.8084737	1	1
1006870	1	0.6504241	2	1
1006871	2	0.6939999	2	1
1006872	2	0.8043698	1	1
1006872	1	0.8726236	0	0
1006873	1	0.7032168	4	1
1006874	1	0.8345919	0	1
1006874	2	0.9499006	0	0
1006875	2	0.0670476	6	0
1006876	1	0.4715458	0	1
1006877	2	0.8303496	0	0
1006878	1	0.5740564	0	0
1006879	2	0.9735961	0	0
1006880	2	0.068379	2	0
1006881	2	0.6248208	0	0
1006882	2	0.3471566	1	0
1006883	1	0.3028421	3	1
1006884	1	0.2102779	3	1
1006884	2	0.2006496	6	0
1006885	1	0.1744498	6	0
1006886	1	0.2087721	3	0
1006887	1	0.7204169	0	0

Patient ID	Side	Propensity score	Number of tranfusions	Matched
1006888	1	0.3252061	1	0
1006889	2	0.5312783	0	1
1006890	2	0.7496943	0	0
1006891	1	0.1661733	2	0
1006892	1	0.8450363	0	0
1006893	2	0.8923432	0	0
1006894	1	0.6909467	0	0
1006895	2	0.9557838	0	0
1006895	1	0.8979584	0	0
1006896	1	0.925874	0	0
1006897	1	0.4070335	1	1
1006898	1	0.1083004	2	1
1006899	1	0.8604372	0	0
1006900	1	0.9718675	0	0
1006901	2	0.9024512	0	0
1006902	2	0.8707224	0	0
1006903	2	0.5222317	0	1
1006903	1	0.2023194	2	0
1006904	2	0.2416096	0	1
1006905	2	0.5795027	2	1
1006906	1	0.714073	0	1
1006906	2	0.714073	0	0
1006907	2	0.240539	10	1
1006908	2	0.3837169	2	1
1006909	1	0.8267231	0	0
1006910	1	0.2024319	4	1
1006911	1	0.8287529	2	1
1006912	1	0.6179632	0	1
1006913	1	0.0875032	4	0
1006913	2	0.3305501	3	1
1006914	2	0.7673	0	0
1006915	1	0.467087	0	1
1006916	2	0.9557838	0	0
1006917	1	0.7881868	2	1
1006918	2	0.8667763	0	0
1006919	1	0.620406	0	1
1006920	1	0.2260023	0	1
1006921	1	0.362906	6	1
1006922	2	0.806943	0	1

Patient ID	Side	Propensity score	Number of tranfusions	Matched
1006923	1	0.7010806	0	1
1006923	2	0.6299819	0	1
1006924	2	0.6384172	0	0
1006925	1	0.9471644	0	0
1006926	2	0.1836016	5	1
1006927	1	0.7471596	0	0
1006928	1	0.2844035	3	0
1006929	1	0.8381982	0	0
1006930	1	0.9653663	0	0
1006931	1	0.8572502	0	0
1006931	2	0.7751945	2	1
1006932	2	0.5623317	2	1
1006933	2	0.9557483	0	0
1006934	2	0.9486418	0	0
1006935	2	0.7511338	0	1
1006936	1	0.8483376	0	0
1006937	2	0.7317822	0	1
1006938	1	0.796731	0	0
1006939	2	0.656765	0	1
1006940	1	0.5797566	0	1
1006941	1	0.1460613	2	0
1006942	1	0.9639877	0	0
1006943	1	0.4951376	2	1
1006944	2	0.3084504	0	1
1006945	2	0.6669915	0	1
1006946	2	0.9898134	0	0
1006946	1	0.9898134	0	0
1006947	1	0.8311099	0	0
1006948	2	0.0532364	1	0
1006949	2	0.4335273	4	1
1006950	2	0.7257291	0	0
1006951	2	0.8719391	0	0
1006952	2	0.8486201	0	0
1006953	2	0.9527564	0	0
1006954	1	0.7429692	0	0
1006955	2	0.9732879	0	0
1006956	1	0.8349239	0	0
1006957	1	0.9366489	3	1
1006958	1	0.6321596	0	1

Patient ID	Side	Propensity score	Number of tranfusions	Matched
1006959	2	0.8851865	0	0
1006960	2	0.4104307	0	1
1006961	1	0.5614328	0	0
1006961	2	0.8297242	0	0
1006962	1	0.9557483	0	0
1006963	2	0.6321596	2	1
1006964	1	0.8720679	0	0
1006965	2	0.2473207	0	1
1006966	2	0.6820516	4	1
1006967	1	0.7139026	0	1
1006967	2	0.7139026	2	1
1006968	1	0.8390119	0	0
1006969	1	0.8719391	0	0
1006970	2	0.7059057	4	1
1006970	1	0.7807217	0	0
1006971	1	0.7370072	0	1
1006972	2	0.9611736	0	0
1006973	1	0.0750504	2	1
1006974	1	0.833655	0	1
1006975	2	0.8247858	4	1
1006976	2	0.9568595	0	0
1006977	2	0.559449	0	1
1006977	1	0.8143077	0	0
1006978	2	0.0913976	4	0
1006978	1	0.0913976	3	0
1006979	2	0.8074818	0	0
1006980	2	0.3773102	2	0
1006980	1	0.3773102	2	0
1006981	1	0.4044706	0	1
1006981	2	0.5308739	0	1
1006982	2	0.4238313	5	1
1006982	1	0.137072	7	0
1006983	2	0.9472042	0	1
1006984	2	0.7836873	0	0
1006985	2	0.9459836	0	0
1006986	2	0.2972059	0	1
1006987	1	0.9555547	2	1
1006988	2	0.6179632	4	1
1006989	2	0.8740013	2	1

Patient ID	Side	Propensity score	Number of tranfusions	Matched
1006990	1	0.7610734	0	0
1006991	1	0.8156355	2	1
1006992	2	0.0884485	2	1
1006993	2	0.3305501	0	1
1006994	2	0.4190954	5	0
1006995	1	0.200341	0	1
1006996	1	0.4621709	0	1
1006997	1	0.6432543	2	1
1006998	1	0.6579239	0	0
1006999	2	0.8964472	0	0
1007000	2	0.5786815	0	1
1007001	2	0.2799763	2	1
1007001	1	0.3250186	3	1
1007002	1	0.9796864	0	0
1007003	1	0.7783576	0	0
1007004	2	0.9766817	0	0
1007005	2	0.6174811	0	1
1007006	2	0.8199402	0	0
1007007	1	0.7783576	0	0
1007008	1	0.7663047	2	1
1007009	2	0.1067797	6	0
1007010	2	0.0911258	11	0
1007011	2	0.2944816	0	1
1007012	1	0.1995049	7	1
1007013	2	0.3610225	0	1
1007014	2	0.8187	0	0
1007015	1	0.5181515	0	1
1007016	2	0.833655	0	0
1007017	2	0.8544433	0	0
1007018	1	0.9113517	0	0
1007019	1	0.9069528	0	0
1007020	1	0.473773	2	1
1007021	2	0.0526129	7	0
1007022	1	0.2197414	3	0
1007023	2	0.9555191	0	0
1007024	2	0.712234	3	1
1007024	1	0.4592113	1	1
1007025	2	0.7798196	0	0
1007026	1	0.6210378	0	1

Patient ID	Side	Propensity score	Number of tranfusions	Matched
1007027	2	0.9653663	0	0
1007028	2	0.1289196	3	0
1007029	2	0.9424865	0	0
1007030	2	0.5290465	2	1
1007031	1	0.9568595	0	0
1007032	1	0.755473	0	1
1007033	2	0.7751945	0	1
1007034	1	0.5912416	0	1
1007035	2	0.969153	0	0
1007036	1	0.026457	4	0
1007037	2	0.3302219	0	1
1007038	2	0.9084513	2	1
1007039	2	0.9431339	0	0
1007040	1	0.6443724	0	0
1007041	2	0.8505259	0	0
1007042	2	0.8929252	0	0
1007043	1	0.9898134	0	0
1007044	1	0.5623317	0	1
1007045	2	0.793514	1	1
1007046	2	0.8244078	0	0
1007047	2	0.5207995	7	1
1007048	1	0.4693145	5	1
1007049	1	0.3455151	0	1
1007050	2	0.6594796	2	1
1007051	2	0.1486094	2	0
1007052	2	0.9366489	0	0
1007053	1	0.8143077	0	1
1007054	1	0.6787576	0	1
1007055	1	0.5711182	0	1
1007056	1	0.8143077	0	0
1007057	2	0.8061834	0	0
1007058	2	0.1583994	0	1
1007059	1	0.810253	0	0
1007060	1	0.9623818	0	0
1007061	1	0.6617085	4	1
1007062	1	0.9271049	0	0
1007063	1	0.4592113	1	1
1007064	1	0.7264057	0	0
1007065	1	0.2334442	0	1

Patient ID	Side	Propensity score	Number of tranfusions	Matched
1007066	1	0.6156725	2	1
1007067	2	0.9364664	0	0
1007068	1	0.5795027	0	1
1007069	1	0.8003601	0	1
1007070	2	0.9481907	0	0
1007071	1	0.4282856	0	1
1007072	2	0.7994878	0	0
1007073	1	0.1698545	5	0
1007074	2	0.8590341	0	0
1007075	1	0.9766817	2	1
1007076	1	0.9568595	0	0
1007077	2	0.1776719	3	0
1007078	1	0.8382619	0	0
1007079	1	0.5482672	1	1
1007080	2	0.3048106	0	1
1007081	2	0.5166094	3	1
1007082	2	0.9457731	0	0
1007083	2	0.8926175	0	0
1007084	2	0.2055755	2	0
1007085	2	0.6167163	5	1
1007086	1	0.8825185	0	0
1007087	1	0.3697271	3	1
1007088	1	0.7182463	0	0
1007089	1	0.0483913	3	0
1007090	2	0.9039453	0	0
1007091	2	0.0086626	6	0
1007092	1	0.8235636	0	0
1007093	2	0.7341019	0	0
1007094	1	0.8464121	0	0
1007095	2	0.6210378	1	1
1007096	2	0.4796653	0	1
1007097	2	0.8531178	0	0
1007098	1	0.824614	0	0
1007098	2	0.8967338	0	0
1007099	2	0.3514441	3	0
1007100	2	0.1791882	2	0
1007101	2	0.6175915	0	1
1007102	2	0.7613885	2	1
1007103	1	0.7138674	0	1
1007104	2	0.9723309	0	0

Patient ID	Side	Propensity score	Number of tranfusions	Matched
1007105	2	0.5482672	0	1
1007106	1	0.2442493	1	0
1007107	1	0.9472872	0	1
1007108	1	0.7587011	2	1
1007108	2	0.7956537	0	0
1007109	1	0.8143077	0	0
1007110	2	0.9512951	0	0
1007111	1	0.9505225	0	0
1007112	1	0.4126884	5	1
1007113	2	0.8306983	0	0
1007114	1	0.8585083	0	0
1007115	1	0.8892304	0	0
1007116	2	0.8016836	0	0
1007117	2	0.882295	0	0
1007118	1	0.7139026	0	1
1007119	1	0.6179632	0	1
1007120	2	0.7451044	0	0
1007121	1	0.7062283	0	1
1007122	1	0.1698545	3	0
1007123	2	0.9197193	0	0
1007124	1	0.7233331	0	0
1007125	2	0.1817046	2	1
1007126	2	0.8892304	0	0
1007127	1	0.840731	0	0
1007127	2	0.3554994	0	1
1007128	2	0.9720847	0	0
1007129	1	0.9395485	0	0
1007130	2	0.7840198	0	0
1007130	1	0.9472042	2	1
1007131	2	0.851423	0	0
1007132	1	0.4637274	2	1
1007133	2	0.6758651	10	1
1007134	1	0.7941224	0	0
1007135	1	0.5623317	0	1
1007136	2	0.8293664	2	1
1007137	2	0.51449	0	1
1007138	2	0.914542	0	0
1007139	2	0.4181951	0	1
1007140	2	0.8892304	0	0
1007141	2	0.7999699	0	1

Patient ID	Side	Propensity score	Number of tranfusions	Matched
1007142	1	0.6807524	0	0
1007143	2	0.7564785	0	0
1007144	1	0.559449	0	0
1007144	2	0.6790591	2	1
1007145	1	0.8267231	0	0
1007146	1	0.6319291	0	1
1007147	1	0.1530738	2	0
1007148	1	0.6449332	2	1
1007149	2	0.8720679	0	0
1007150	2	0.1624789	2	0
1007151	2	0.9611736	0	0
1007151	1	0.8963693	0	0
1007152	2	0.6271352	1	1
1007153	1	0.7772751	2	1
1007154	1	0.8066371	0	0
1007155	1	0.9743555	0	1
1007156	1	0.3082673	4	1
1007157	1	0.9653663	0	0
1007158	1	0.9682459	0	0
1007159	1	0.4667833	3	1
1007159	2	0.5943448	3	1
1007160	1	0.8143077	0	0
1007161	1	0.8684553	0	0
1007162	1	0.9756276	0	0
1007163	1	0.9082494	0	0
1007164	2	0.022764	6	0
1007165	1	0.8045401	2	1
1007166	1	0.9070476	0	0
1007167	1	0.3192288	7	0
1007168	2	0.8628989	0	0
1007169	1	0.9611736	0	0
1007170	2	0.9568595	0	0
1007171	1	0.8684553	0	0
1007171	2	0.8892304	0	0
1007172	1	0.683496	2	1
1007173	1	0.4701301	3	1
1007174	1	0.6867087	0	1
1007175	2	0.8380845	3	1
1007176	2	0.9041181	0	0

Patient ID	Side	Propensity score	Number of tranfusions	Matched
1007177	2	0.8604233	0	0
1007178	2	0.87418	0	0
1007179	2	0.7240914	0	0
1007180	2	0.552885	0	1
1007181	1	0.739302	0	1
1007182	1	0.8103819	0	1
1007183	1	0.8583781	0	0
1007184	2	0.2733145	4	0
1007185	1	0.7663047	0	1
1007186	1	0.8622236	0	0
1007187	2	0.8643851	0	0
1007188	1	0.6062345	0	1
1007189	1	0.687843	0	1
1007190	2	0.3770483	1	0
1007191	2	0.203955	2	0
1007192	2	0.6156725	3	1
1007193	1	0.9366489	0	0
1007194	2	0.8200548	0	0
1007195	2	0.9197193	0	0
1007196	2	0.6872183	0	1
1007197	2	0.8684553	0	0
1007198	2	0.9024512	0	0
1007199	2	0.2067997	2	0
1007200	2	0.9653663	0	0
1007201	1	0.3961032	0	1
1007202	1	0.6685409	4	1
1007203	2	0.9051527	0	0
1007204	1	0.0506237	3	0
1007205	2	0.9505225	0	0
1007206	2	0.7490443	1	1
1007207	1	0.5716151	4	1
1007208	2	0.3095169	0	1
1007208	1	0.112277	0	1
1007209	1	0.7910648	2	1
1007210	2	0.0272167	2	0
1007211	2	0.8003851	0	0
1007212	2	0.6477752	2	1
1007212	1	0.8569584	0	0
1007213	2	0.740117	0	0
1007214	1	0.9827961	0	0

Patient ID	Side	Propensity score	Number of tranfusions	Matched
1007215	1	0.6362959	3	1
1007216	1	0.8871914	0	0
1007217	1	0.6600526	0	0
1007218	2	0.6722247	0	0
1007219	1	0.6565705	4	1
1007219	2	0.3504041	4	0
1007220	1	0.0779573	6	0
1007221	1	0.6212756	0	0
1007222	1	0.9239261	0	0
1007223	2	0.7999699	0	1
1007224	1	0.5307699	0	1
1007225	1	0.4139172	3	1
1007226	2	0.4044706	0	1
1007227	1	0.8006195	0	1
1007228	2	0.8372281	0	0
1007229	2	0.4580208	10	1
1007230	1	0.9555547	0	0
1007231	2	0.9222556	0	0
1007232	2	0.9004673	0	1
1007233	1	0.5578053	0	1
1007234	2	0.0567909	4	0
1007235	2	0.622981	1	1
1007236	2	0.2145431	1	1
1007237	1	0.9718675	0	0
1007238	2	0.5673484	5	1
1007238	1	0.8363182	0	0
1007239	2	0.6842546	2	1
1007240	1	0.7451044	0	1
1007241	1	0.5904373	0	1
1007242	2	0.321832	0	1
1007243	2	0.1662811	0	1
1007244	2	0.2325698	3	1
1007245	1	0.801863	0	0
1007245	2	0.8610245	0	0
1007246	2	0.8604695	0	0
1007247	1	0.9568595	0	0
1007248	1	0.1170975	1	0
1007249	2	0.8066371	0	0
1007250	2	0.9653663	0	0

Patient ID	Side	Propensity score	Number of tranfusions	Matched
1007251	1	0.4614684	0	1
1007252	2	0.6174811	1	1
1007253	2	0.6637491	1	1
1007254	2	0.3028421	0	1
1007255	2	0.7103589	4	1
1007256	1	0.4109022	0	1
1007257	1	0.6143042	0	0
1007258	2	0.6349469	2	1
1007259	1	0.1680335	2	0
1007260	2	0.9410451	0	0
1007261	2	0.5797566	2	1
1007262	2	0.2413515	0	1
1007263	2	0.7528192	0	0
1007264	1	0.7113339	0	0
1007265	2	0.8590341	0	0
1007266	2	0.957775	0	0
1007267	1	0.9653663	0	0
1007268	2	0.9481907	2	1
1007269	1	0.911852	0	0
1007270	2	0.5806389	6	1
1007271	1	0.6339748	0	1
1007272	1	0.5449822	0	0
1007273	1	0.0785958	7	0
1007274	1	0.7717639	0	1
1007275	2	0.56959	2	1
1007275	1	0.755473	0	1
1007276	1	0.4044706	3	1
1007277	1	0.7346402	0	0
1007278	1	0.4869262	0	1
1007279	2	0.4164292	4	1
1007280	1	0.9395485	0	0
1007281	2	0.3112866	0	1
1007282	2	0.526943	3	1
1007283	1	0.3637847	2	1
1007284	2	0.410127	0	1
1007285	1	0.9555547	0	0
1007286	2	0.8199402	0	0
1007287	2	0.8569584	0	0
1007288	1	0.9653663	0	0

Patient ID	Side	Propensity score	Number of tranfusions	Matched
1007289	2	0.5985009	0	1
1007290	1	0.8111391	0	0
1007290	2	0.8905035	0	0
1007291	2	0.1268756	2	0
1007292	1	0.3177068	6	1
1007293	2	0.1548002	2	0
1007294	1	0.3556734	0	1
1007294	2	0.7860082	0	0
1007295	2	0.8724139	0	1
1007296	1	0.8531178	0	0
1007297	2	0.9557483	0	0
1007297	1	0.9557483	0	0
1007298	2	0.8800648	0	0
1007299	2	0.3826855	8	1
1007300	2	0.6112214	11	1
1007301	2	0.3520014	2	0
1007302	1	0.9191106	0	0
1007303	1	0.7451044	2	1
1007304	1	0.9512951	0	0
1007305	2	0.5195642	0	1
1007305	1	0.5049889	2	1
1007306	2	0.8381982	0	0
1007307	1	0.3908383	1	1
1007308	1	0.6401157	0	0
1007309	2	0.6339748	0	0
1007310	2	0.3637176	2	0
1007311	1	0.2067997	1	0
1007312	1	0.8583781	0	0
1007313	1	0.5217103	0	1
1007314	2	0.4037822	6	1
1007315	2	0.7717639	0	0
1007316	1	0.4349635	1	0
1007317	1	0.6500681	0	1
1007317	2	0.9639877	0	0
1007318	2	0.5964152	0	1
1007319	2	0.9504737	0	0
1007320	1	0.8270586	0	0
1007321	1	0.2056589	4	0
1007321	2	0.4785176	3	1

Patient ID	Side	Propensity score	Number of tranfusions	Matched
1007322	2	0.7346398	0	0
1007323	1	0.5623317	2	1
1007324	1	0.6143042	3	1
1007325	1	0.4524742	0	1
1007326	1	0.9482519	0	0
1007327	2	0.5935675	0	1
1007328	2	0.6090651	3	1
1007329	1	0.8196841	1	1
1007330	1	0.5041998	2	1
1007331	1	0.4071583	16	1
1007332	1	0.2445827	2	1
1007333	1	0.4353576	2	1
1007334	1	0.7190104	0	1
1007335	1	0.6598216	0	1
1007336	2	0.8793957	0	0
1007337	1	0.6506863	0	0
1007338	2	0.9776562	0	0
1007339	1	0.3874848	2	1
1007340	1	0.8923432	0	0
1007341	2	0.8923432	0	0
1007342	1	0.7838778	0	0
1007343	2	0.9611736	0	0
1007344	2	0.4422937	0	1
1007345	2	0.9568595	0	0
1007346	1	0.1791882	4	1
1007347	2	0.3351131	0	1
1007348	1	0.4135821	5	1
1007349	1	0.7699598	0	0
1007350	1	0.1947112	1	1
1007351	2	0.0972561	3	0
1007352	1	0.9663259	0	0
1007353	1	0.9557483	0	0
1007354	1	0.1969915	0	1
1007355	1	0.7451044	2	1
1007356	2	0.2341951	2	0
1007357	1	0.898628	0	0
1007358	2	0.801863	0	0
1007358	1	0.9457731	0	0
1007359	1	0.6368605	0	1
1007360	2	0.7196551	2	1

Patient ID	Side	Propensity score	Number of tranfusions	Matched
1007360	1	0.898628	0	0
1007361	2	0.5867526	1	1
1007362	1	0.9289634	0	0
1007363	2	0.8578785	0	1
1007364	1	0.7836873	2	1
1007365	1	0.0089759	4	0
1007366	2	0.4144451	2	1
1007367	1	0.9041181	0	0
1007367	2	0.9040454	0	0
1007368	1	0.7445205	2	1
1007369	2	0.6506863	0	0
1007370	2	0.7613885	0	1
1007371	1	0.3212566	3	1
1007372	2	0.6339748	0	0
1007373	2	0.8410779	0	0
1007374	2	0.1859022	1	0
1007375	1	0.8414306	0	0
1007376	2	0.678968	4	1
1007377	1	0.6349469	2	1
1007378	1	0.79514	0	0
1007379	2	0.8859565	0	0
1007380	2	0.3798259	2	0
1007380	1	0.6210378	0	1
1007381	2	0.639349	0	0
1007381	1	0.7479218	0	0
1007382	1	0.882295	0	0
1007383	2	0.9180547	0	0
1007384	1	0.9093815	0	0
1007385	2	0.1548002	7	0
1007386	2	0.9119742	0	0
1007387	2	0.9086713	1	1
1007387	1	0.7373435	0	0
1007388	1	0.8337712	0	0
1007389	2	0.6790591	0	1
1007390	1	0.0617327	10	0
1007391	1	0.9555191	0	0
1007392	2	0.6989718	3	1
1007393	2	0.9106365	0	0
1007394	1	0.9639877	0	0

Patient ID	Side	Propensity score	Number of tranfusions	Matched
1007395	1	0.8963693	0	0
1007396	1	0.1461907	7	0
1007397	2	0.5740564	2	1
1007398	2	0.8622236	0	0
1007399	1	0.7907302	0	1
1007400	1	0.6679504	0	1
1007401	1	0.8143077	0	0
1007402	1	0.7563266	0	0
1007403	1	0.3175601	2	0
1007404	1	0.9448927	0	0
1007405	2	0.8160213	0	1
1007406	2	0.8892304	0	0
1007406	1	0.9380204	0	0
1007407	1	0.5201238	2	1
1007408	2	0.8591355	0	0
1007409	2	0.8651406	0	0
1007409	1	0.9236301	0	1
1007410	1	0.9380204	0	0
1007411	2	0.8531178	0	0
1007412	2	0.1476202	0	1
1007413	2	0.6822033	0	0
1007414	1	0.2711709	2	1
1007415	1	0.7097606	0	1
1007416	1	0.9565483	0	0
1007417	1	0.6319744	0	1
1007418	1	0.9490839	0	1
1007419	2	0.9040454	0	0
1007420	1	0.9265721	0	0
1007421	2	0.8303496	0	0
1007421	1	0.8583781	0	0
1007422	2	0.467087	0	1
1007423	1	0.9457731	0	0
1007423	2	0.9348254	0	1
1007424	2	0.1150845	14	0
1007425	2	0.467087	6	1
1007426	2	0.286449	12	0
1007427	2	0.4535268	0	1
1007428	1	0.5234918	0	1
1007428	2	0.6743881	4	1
1007429	1	0.6293322	1	1

Patient ID	Side	Propensity score	Number of tranfusions	Matched
1007430	1	0.2034425	0	1
1007431	1	0.6275256	0	1
1007432	2	0.9684105	0	0
1007433	1	0.6339748	0	1
1007434	2	0.9086713	0	0
1007435	2	0.8987589	0	0
1007436	1	0.9366489	2	1
1007437	1	0.6743881	10	1
1007438	2	0.3503485	1	0
1007439	2	0.9787826	0	0
1007440	2	0.9395485	0	0
1007441	2	0.7338573	0	0
1007442	2	0.7845374	0	1
1007443	2	0.3504041	3	0
1007444	2	0.8870113	0	0
1007445	2	0.9653663	0	0
1007446	1	0.7751945	0	0
1007446	2	0.9380204	0	0
1007447	2	0.2473207	2	0
1007448	1	0.6246513	6	1
1007449	1	0.6443419	4	1
1007450	1	0.3164344	0	1
1007451	1	0.796731	0	1
1007452	2	0.0062568	4	0
1007453	2	0.8254554	2	1
1007454	1	0.132016	6	0
1007455	2	0.9256307	0	0
1007456	2	0.7789676	2	1
1007457	1	0.8066371	1	1
1007458	1	0.8311099	0	0
1007458	2	0.8311099	0	0
1007459	2	0.1780778	3	0
1007460	1	0.5453972	1	1
1007461	2	0.9119742	0	0
1007462	1	0.9557483	0	0
1007463	1	0.8349239	0	0
1007464	2	0.4349635	4	0
1007464	1	0.3115666	4	0
1007465	1	0.733567	0	1

Patient ID	Side	Propensity score	Number of tranfusions	Matched
1007466	1	0.4528818	0	1
1007467	2	0.3057131	13	0
1007468	1	0.774531	0	0
1007469	1	0.8923432	0	0
1007470	1	0.7370072	1	1
1007471	1	0.5227044	0	1
1007472	2	0.9139531	0	0
1007472	1	0.9639877	0	0
1007473	1	0.8548234	2	1
1007474	2	0.4964228	0	1
1007475	1	0.8574622	2	1
1007476	2	0.9095091	0	1
1007477	1	0.2936035	8	1
1007478	2	0.9315427	0	0
1007479	1	0.6084842	0	0
1007480	1	0.4209728	2	1
1007481	2	0.5797566	2	1
1007482	1	0.6544893	0	1
1007483	2	0.9653663	0	0
1007484	2	0.4796653	2	1
1007485	2	0.2830815	0	1
1007486	2	0.8904756	0	0
1007487	2	0.8001187	1	1
1007487	1	0.9568595	0	0
1007488	2	0.7089199	3	1
1007489	1	0.7797947	0	0
1007490	1	0.5077109	2	1
1007491	1	0.7613885	0	1
1007492	1	0.4185981	2	0
1007492	2	0.8926175	0	0
1007493	2	0.2884926	0	1
1007494	1	0.9743555	0	0
1007495	2	0.9107936	2	1
1007496	1	0.8528269	0	0
1007497	1	0.1275878	4	0
1007498	1	0.3045899	4	0
1007498	2	0.4229888	3	1
1007499	1	0.9436676	0	0
1007500	2	0.8591355	0	0
1007501	1	0.1715548	3	0

Patient ID	Side	Propensity score	Number of tranfusions	Matched
1007502	1	0.0532364	2	0
1007503	1	0.0986368	2	0
1007504	1	0.0900117	4	1
1007505	1	0.9457731	0	0
1007506	1	0.9605683	0	0
1007507	1	0.7861992	0	0
1007508	1	0.4785176	2	1
1007509	1	0.2431445	2	1
1007510	1	0.6710028	0	1
1007511	2	0.755473	0	1
1007512	1	0.321832	2	1
1007512	2	0.5985009	2	1
1007513	1	0.6019541	0	1
1007514	2	0.4353576	2	1
1007515	2	0.4722031	1	1
1007516	2	0.7609577	0	0
1007517	1	0.9095091	0	0
1007518	2	0.8984905	0	0
1007519	1	0.9166842	0	1
1007520	2	0.3908383	0	1
1007521	1	0.7624756	0	0
1007522	2	0.5447981	0	0
1007523	2	0.8159437	0	1
1007524	1	0.7613885	1	1
1007525	2	0.4763374	63	1
1007526	2	0.8531178	0	0
1007527	1	0.7138674	0	1
1007527	2	0.56959	0	1
1007528	1	0.5401787	3	1
1007529	2	0.7138674	0	1
1007530	2	0.3305501	2	1
1007531	1	0.3649516	1	1
1007532	1	0.883558	0	0
1007533	2	0.8652821	0	0
1007534	1	0.473773	2	1
1007535	2	0.9095091	0	0
1007536	2	0.8926175	0	0
1007537	1	0.3212566	3	0
1007537	2	0.3212566	5	0

Patient ID	Side	Propensity score	Number of tranfusions	Matched
1007538	1	0.9156797	2	1
1007538	2	0.9307857	0	0
1007539	2	0.9348254	0	0
1007539	1	0.9467011	0	0
1007540	2	0.7672793	0	0
1007541	2	0.9735961	0	0
1007542	1	0.3239959	0	1
1007542	2	0.8242864	0	0
1007543	1	0.9568595	0	0
1007544	1	0.9855258	0	0
1007545	2	0.9395485	0	0
1007546	2	0.4733407	0	1
1007547	1	0.3504041	2	1
1007548	2	0.8483376	4	1
1007549	2	0.6884223	0	1
1007550	2	0.6308935	2	1
1007551	2	0.2467381	2	1
1007552	1	0.8149367	0	0
1007552	2	0.8450363	0	0
1007553	1	0.7668433	1	1
1007554	1	0.9557483	0	0
1007555	1	0.2422995	3	1
1007556	2	0.9653663	0	0
1007557	2	0.9796864	0	0
1007558	1	0.2202349	4	0
1007559	1	0.5080693	3	1
1007559	2	0.4545422	2	1
1007560	2	0.0986374	4	0
1007561	2	0.6977639	2	1
1007562	2	0.6506863	0	0
1007562	1	0.810253	0	0
1007563	2	0.8234012	0	0
1007564	2	0.8722887	0	0
1007565	2	0.6051389	3	1
1007566	2	0.9197193	0	1
1007567	1	0.9527564	0	0
1007568	1	0.5211484	0	1
1007569	1	0.9119742	2	1
1007570	1	0.928416	0	0

Patient ID	Side	Propensity score	Number of tranfusions	Matched
1007571	2	0.2525808	6	1
1007572	2	0.4906955	0	1
1007573	1	0.6538066	4	1
1007574	1	0.56959	2	1
1007575	1	0.9568595	0	0
1007576	1	0.928416	0	0
1007577	2	0.9680528	0	0
1007578	2	0.9145093	0	0
1007579	1	0.4625525	0	1
1007580	2	0.6822033	1	1
1007581	2	0.5307699	0	1
1007582	1	0.0726617	30	0
1007583	1	0.5659327	0	1
1007584	1	0.9068288	0	0
1007584	2	0.9017812	0	0
1007585	2	0.9366489	0	0
1007586	1	0.8823384	0	0
1007587	2	0.9207713	0	0
1007588	2	0.0902146	5	0
1007589	1	0.7148756	0	1
1007590	1	0.120285	5	0
1007591	2	0.9571506	0	0
1007592	2	0.1112753	3	0
1007593	2	0.4757078	0	1
1007593	1	0.7472001	0	0
1007594	1	0.8893129	0	0
1007595	2	0.9262035	0	0
1007596	2	0.7010806	2	1
1007597	1	0.0900471	0	1
1007598	2	0.8380845	0	1
1007599	1	0.7613885	2	1
1007600	1	0.8791089	0	0
1007601	2	0.678968	2	1
1007602	2	0.0075074	13	0
1007603	2	0.204235	6	0
1007604	2	0.6202962	1	1
1007605	1	0.2314453	2	0
1007606	2	0.8151238	0	1
1007606	1	0.7004812	0	0

Patient ID	Side	Propensity score	Number of tranfusions	Matched
1007607	2	0.4998259	2	1
1007608	1	0.1962029	2	1
1007609	1	0.7035234	0	0
1007610	1	0.3065288	0	1
1007611	2	0.8512175	0	1
1007612	2	0.9811312	0	0
1007613	1	0.6447928	0	1
1007613	2	0.9472042	0	0
1007614	2	0.8143077	0	0
1007615	2	0.9163173	0	0
1007616	2	0.8857348	0	0
1007616	1	0.9639877	0	1
1007617	2	0.782809	0	0
1007617	1	0.782809	0	0
1007618	2	0.0682113	3	0
1007619	1	0.7115915	0	1
1007620	2	0.8186172	2	1
1007621	2	0.2620691	3	0
1007622	1	0.473773	0	1
1007623	1	0.0649953	4	0
1007624	1	0.2550914	2	0
1007625	1	0.9493857	0	0
1007626	1	0.4766895	4	1
1007627	2	0.1623618	8	0
1007628	1	0.9653663	0	0
1007629	1	0.2801864	0	1
1007630	1	0.467087	1	1
1007631	1	0.3504041	2	0
1007632	2	0.8349239	0	1
1007633	2	0.321832	0	1
1007634	2	0.1366643	3	0
1007635	1	0.4701007	0	1
1007636	2	0.8182057	0	0
1007637	2	0.9569282	0	0
1007638	1	0.6608236	0	0
1007639	1	0.7861992	0	0
1007640	2	0.56959	0	1
1007641	1	0.9145093	0	0
1007642	1	0.021923	6	0

Patient ID	Side	Propensity score	Number of tranfusions	Matched
1007643	2	0.9623818	0	0
1007644	2	0.9512951	0	0
1007645	2	0.8380845	0	0
1007646	1	0.6211123	0	0
1007647	2	0.3617646	2	1
1007648	1	0.9568595	2	1
1007649	2	0.6842546	0	0
1007650	1	0.5965624	6	1
1007651	1	0.9067378	0	0
1007652	2	0.9405592	1	1
1007653	2	0.7701146	0	0
1007654	1	0.3371508	2	0
1007655	2	0.7994878	0	0
1007655	1	0.7994878	0	0
1007656	1	0.9340456	0	0
1007657	2	0.4381453	2	0
1007658	1	0.8871914	0	0
1007659	1	0.5602688	0	1
1007660	1	0.0694331	4	0
1007661	2	0.4224298	3	0
1007662	2	0.7730314	2	1
1007663	2	0.5557157	2	1
1007664	2	0.3133725	7	0
1007665	2	0.4710574	0	1
1007666	2	0.1957609	2	0
1007667	1	0.755473	2	1
1007668	2	0.5875321	0	1
1007669	1	0.9366737	1	1
1007670	2	0.9557838	0	0
1007671	2	0.6271352	0	1
1007671	1	0.6271352	2	1
1007672	1	0.8390119	0	0
1007673	1	0.5541489	0	0
1007674	2	0.9611736	0	0
1007675	1	0.88625	0	0
1007676	1	0.599822	0	1
1007676	2	0.8355938	0	0
1007677	2	0.2341951	6	0
1007678	1	0.6832323	0	1

Patient ID	Side	Propensity score	Number of tranfusions	Matched
1007679	2	0.380564	0	1
1007680	2	0.806943	2	1
1007681	1	0.9399637	0	0
1007682	1	0.8923432	0	0
1007683	2	0.7861992	2	1
1007684	2	0.8767222	0	1
1007685	1	0.4942396	2	1
1007685	2	0.3302219	2	1
1007686	1	0.9380204	0	0
1007686	2	0.810253	0	0
1007687	2	0.6860194	2	1
1007688	1	0.4418074	1	0
1007689	1	0.9639877	0	0
1007690	2	0.8979584	0	0
1007691	1	0.0134826	6	0
1007692	1	0.9086713	0	0
1007693	1	0.9086713	0	0
1007694	1	0.914542	0	0
1007695	2	0.1440325	2	0
1007696	1	0.4851406	0	1
1007697	2	0.4144451	0	1
1007698	2	0.5435919	2	1
1007699	1	0.6213782	2	1
1007700	2	0.8531178	0	0
1007701	2	0.6468954	0	1
1007702	1	0.2328377	5	0
1007703	1	0.4139172	1	1
1007704	1	0.8880704	0	0
1007704	2	0.8580362	0	0
1007705	2	0.9031482	0	0
1007706	1	0.4912457	0	1
1007707	2	0.3395872	2	1
1007708	1	0.5405086	0	1
1007708	2	0.6631602	0	0
1007709	2	0.7451837	3	1
1007709	1	0.8583781	0	0
1007710	2	0.8388081	0	0
1007710	1	0.7340607	2	1
1007711	1	0.108706	4	0

Patient ID	Side	Propensity score	Number of tranfusions	Matched
1007712	2	0.0846775	4	0
1007713	1	0.2236856	0	1
1007714	1	0.9197193	0	0
1007715	2	0.678968	0	0
1007716	1	0.4454381	0	1
1007717	1	0.9653663	0	0
1007718	1	0.1055341	2	0
1007719	1	0.2550914	0	1
1007720	2	0.799333	0	0
1007721	1	0.6805081	0	1
1007722	2	0.2024319	2	0
1007723	2	0.957775	0	0
1007724	2	0.3404872	1	0
1007725	2	0.0502999	2	0
1007726	2	0.7113339	0	0
1007727	2	0.378616	0	1
1007728	1	0.851055	0	0
1007729	1	0.4374598	0	1
1007730	2	0.8410779	0	0
1007731	2	0.8349239	0	0
1007731	1	0.9050807	0	0
1007732	1	0.701194	4	1
1007732	2	0.8503392	0	0
1007733	1	0.5740564	0	1
1007734	2	0.817672	0	0
1007735	2	0.957775	0	0
1007736	1	0.7149794	0	0
1007737	1	0.7299379	0	0
1007738	1	0.928416	0	0
1007739	1	0.810253	0	0
1007740	1	0.6752185	0	1
1007741	1	0.357722	4	0
1007742	2	0.2906392	1	0
1007743	2	0.6544533	2	1
1007744	1	0.6544533	0	1
1007745	1	0.242314	2	0
1007746	1	0.7470569	2	1
1007747	1	0.109699	3	0
1007748	1	0.882295	1	1
1007749	1	0.5610185	4	1

Patient ID	Side	Propensity score	Number of tranfusions	Matched
1007750	2	0.0228245	6	0
1007751	2	0.905477	0	0
1007752	2	0.9557483	0	0
1007753	1	0.7797947	3	1
1007754	2	0.912419	0	0
1007755	2	0.8199402	0	0
1007756	2	0.1766384	6	0
1007757	1	0.1514817	1	0
1007758	2	0.9106365	0	0
1007759	1	0.683496	0	1
1007760	2	0.8007989	0	0
1007761	1	0.1715548	3	0
1007762	1	0.8483376	0	0
1007763	1	0.9623818	0	0
1007764	1	0.229397	2	0
1007764	2	0.6374046	0	0
1007765	1	0.7471596	0	0
1007766	2	0.9765373	0	0
1007767	1	0.9557838	0	0
1007768	1	0.5298555	0	1
1007769	1	0.9395485	0	1
1007770	1	0.8143077	0	0
1007771	2	0.9342051	0	0
1007772	2	0.473773	1	1
1007773	2	0.5685832	1	1
1007774	2	0.2852641	2	1
1007775	1	0.8512704	0	0
1007776	2	0.9106058	0	1
1007777	2	0.5202207	4	1
1007778	1	0.8082396	0	1
1007779	1	0.3282734	3	0
1007780	2	0.7185892	2	1
1007781	2	0.0602183	4	0
1007782	1	0.1956367	2	0
1007783	2	0.810253	0	0
1007784	1	0.5403612	0	1
1007785	2	0.9718675	0	0
1007786	1	0.9017256	0	0
1007787	1	0.7840198	0	0

Patient ID	Side	Propensity score	Number of tranfusions	Matched
1007788	2	0.9521768	0	0
1007789	2	0.3391898	3	0
1007790	2	0.7705859	0	0
1007791	2	0.3477466	2	0
1007792	2	0.923833	0	0
1007793	1	0.2117264	0	1
1007794	1	0.9180547	0	0
1007795	2	0.8061834	0	0
1007796	2	0.6370454	0	0
1007796	1	0.9454951	0	0
1007797	2	0.3416646	2	0
1007798	1	0.473773	0	1
1007799	1	0.467087	0	1
1007800	1	0.473773	2	1
1007801	2	0.9278036	0	0
1007802	1	0.6103809	2	1
1007803	2	0.3382911	6	1
1007804	2	0.7834102	1	1
1007805	1	0.833455	2	1
1007805	2	0.8610562	2	1
1007806	2	0.9145093	0	0
1007807	1	0.1322334	2	0
1007808	1	0.7473577	0	0
1007808	2	0.9827961	0	0
1007809	1	0.559449	0	1
1007810	2	0.8828985	0	0
1007811	2	0.5780596	2	1
1007811	1	0.5780596	2	1
1007812	1	0.4796653	0	1
1007813	2	0.363642	6	1
1007814	2	0.8684553	0	0
1007814	1	0.9256307	0	0
1007815	2	0.9827961	0	0
1007816	1	0.2666825	2	1
1007817	1	0.7942998	0	0
1007818	1	0.8926175	0	0
1007819	2	0.6292635	2	1
1007820	2	0.5432829	1	1
1007821	1	0.6339748	0	0
1007822	1	0.8143077	0	0

Patient ID	Side	Propensity score	Number of tranfusions	Matched
1007823	1	0.9033303	0	0
1007824	1	0.3602076	3	0
1007825	1	0.957775	0	0
1007826	2	0.3908383	2	1
1007827	1	0.3908383	2	1
1007828	1	0.9366489	0	0
1007829	2	0.075208	7	0
1007830	1	0.9512951	0	0
1007831	2	0.898414	0	0
1007832	2	0.9276642	0	0
1007833	1	0.9835454	0	0
1007834	1	0.9343481	0	0
1007835	1	0.0504265	2	1
1007836	2	0.5370622	0	1
1007837	2	0.9145093	0	0
1007838	2	0.3399404	0	1
1007839	2	0.8464121	0	1
1007840	2	0.8143077	0	0
1007841	1	0.9380204	0	0
1007842	1	0.8923432	0	0
1007843	2	0.182665	0	1
1007844	2	0.9482519	0	0
1007845	2	0.8611213	2	1
1007846	1	0.9442207	0	0
1007847	2	0.263354	2	0
1007848	2	0.2714662	2	1
1007849	1	0.6926273	0	0
1007850	1	0.4109022	2	1
1007850	2	0.4634502	2	1
1007851	2	0.9119742	2	1
1007852	1	0.1394752	6	1
1007853	2	0.2992102	2	1
1007853	1	0.1020288	2	1
1007854	2	0.9550222	0	0
1007855	2	0.5942621	0	1
1007856	2	0.5848193	8	1
1007857	2	0.3305501	0	1
1007858	1	0.3145463	4	0
1007859	2	0.7724198	3	1
1007860	2	0.7585755	0	1

Patient ID	Side	Propensity score	Number of tranfusions	Matched
1007861	1	0.8410779	0	0
1007862	1	0.8733999	0	1
1007863	2	0.9639877	0	0
1007864	1	0.3907341	2	0
1007865	1	0.7025956	0	1
1007866	2	0.1125386	1	1
1007867	1	0.7299379	0	0
1007868	2	0.9339128	0	0
1007869	1	0.7551894	5	1
1007870	1	0.3250186	1	1
1007871	1	0.5548267	0	1
1007872	1	0.56959	3	1
1007873	1	0.7878527	3	1
1007874	2	0.7204169	0	0
1007875	2	0.7025956	0	1
1007876	2	0.678968	2	1
1007877	1	0.9675201	0	0
1007878	1	0.0373787	11	0
1007879	2	0.6624998	6	1
1007880	1	0.6288589	0	1
1007881	1	0.6504241	2	1
1007882	2	0.8414306	0	0
1007883	1	0.4575924	17	1
1007884	1	0.1741835	1	0
1007885	2	0.8322218	0	0
1007886	2	0.8066371	0	0
1007887	2	0.7010806	0	0
1007888	1	0.8004348	0	0
1007889	2	0.8590341	0	0
1007889	1	0.7004812	0	0
1007890	1	0.9395485	0	0
1007891	1	0.5796341	1	1
1007891	2	0.5796341	0	1
1007892	1	0.3028421	0	1
1007892	2	0.3028421	0	1
1007893	1	0.9256307	0	0
1007894	2	0.8464121	1	1
1007895	1	0.9353824	4	1
1007896	2	0.3575682	2	0

Patient ID	Side	Propensity score	Number of tranfusions	Matched
1007897	2	0.6095605	3	1
1007898	2	0.8143077	0	0
1007899	1	0.8021703	0	0
1007900	2	0.6247168	0	1
1007900	1	0.8006502	0	0
1007901	1	0.511364	0	1
1007902	2	0.8293664	0	1
1007903	1	0.7266501	2	1
1007904	1	0.6838794	0	1
1007905	1	0.7533126	0	0
1007906	2	0.6790591	2	1
1007907	1	0.7451044	0	0
1007907	2	0.7451044	0	0
1007908	1	0.2997649	0	1
1007909	1	0.6637512	0	1
1007910	1	0.8569584	0	0
1007911	2	0.7299379	0	0
1007912	2	0.0256509	5	0
1007913	2	0.9482519	0	0
1007914	1	0.9030593	2	1
1007915	1	0.5683046	4	1
1007916	1	0.7317822	1	1
1007917	2	0.3871764	0	1
1007918	2	0.1609039	13	0
1007919	2	0.1055652	4	0
1007920	1	0.7430077	0	0
1007921	2	0.8787133	3	1
1007922	1	0.9732879	0	0
1007923	2	0.3557549	5	1
1007924	1	0.8246191	0	0
1007925	2	0.9301074	0	0
1007925	1	0.965012	0	0
1007926	2	0.286685	3	0
1007927	1	0.914542	0	0
1007928	2	0.8319608	0	1
1007929	1	0.7609577	0	0
1007930	1	0.7866617	0	1
1007931	1	0.7010806	0	1
1007932	1	0.2992102	3	0
1007933	2	0.4614834	7	1

Patient ID	Side	Propensity score	Number of tranfusions	Matched
1007934	1	0.9366489	0	0
1007935	2	0.2239528	2	0
1007936	1	0.9614843	0	0
1007937	2	0.9366489	0	0
1007938	1	0.8131905	1	1
1007939	2	0.4592113	0	1
1007940	2	0.9457731	2	1
1007941	2	0.7636876	0	1
1007942	1	0.3152109	2	1
1007943	2	0.2550914	2	0
1007944	2	0.8720679	0	0
1007945	1	0.6006756	1	1
1007946	1	0.9380204	0	0
1007947	1	0.8823384	0	1
1007948	2	0.6538066	2	1
1007949	2	0.2115551	0	1
1007950	2	0.5557157	0	1
1007951	1	0.9006674	0	0
1007952	2	0.105361	0	1
1007953	1	0.8082396	0	1
1007954	1	0.8410779	0	0
1007955	1	0.1045449	0	1
1007956	1	0.2799763	0	1
1007957	2	0.8905035	0	0
1007958	2	0.1012952	3	0
1007959	2	0.9380204	0	0
1007960	1	0.8512704	0	0
1007961	2	0.9684105	0	0
1007962	1	0.9706023	0	0
1007963	2	0.7743031	0	0
1007964	1	0.7861992	2	1
1007965	2	0.810253	0	0
1007966	1	0.4672155	1	1
1007967	1	0.4008269	0	1
1007968	2	0.8570977	0	0
1007969	2	0.8278654	2	1
1007970	2	0.5557157	2	1
1007971	1	0.7600489	0	0
1007972	1	0.9653663	0	0

Patient ID	Side	Propensity score	Number of tranfusions	Matched
1007973	2	0.0235413	4	0
1007974	1	0.6361699	0	0
1007975	1	0.7610734	0	0
1007976	2	0.0471805	1	0
1007977	1	0.7519121	0	0
1007978	1	0.1184539	11	0
1007979	1	0.2024319	5	0
1007980	1	0.8143077	0	0
1007981	1	0.9684105	0	0
1007982	1	0.0328704	2	0
1007983	1	0.7184489	0	0
1007984	2	0.898414	0	0
1007985	1	0.7317822	0	1
1007986	1	0.8137449	0	0
1007987	2	0.9402535	0	0
1007987	1	0.9639877	0	0
1007988	2	0.9277478	0	0
1007989	1	0.9131293	0	0
1007990	2	0.7663047	0	0
1007991	1	0.9380204	0	0
1007992	2	0.9627395	0	0
1007993	1	0.6544533	2	1
1007994	2	0.9006674	0	0
1007995	1	0.56959	0	1
1007996	1	0.2024319	3	0
1007997	1	0.8143077	0	0
1007998	1	0.8651406	0	0
1007999	1	0.9364763	0	0
1008000	2	0.9718675	0	0
1008001	1	0.2550914	4	0
1008002	2	0.5347764	0	1
1008003	1	0.3539359	3	0
1008004	2	0.6321596	2	1
1008005	2	0.6273714	0	1
1008006	2	0.5312783	3	1
1008007	1	0.3282734	8	1
1008008	1	0.8143077	0	0
1008009	1	0.1442475	0	1
1008010	1	0.9775355	0	0
1008011	2	0.56959	2	1

Patient ID	Side	Propensity score	Number of tranfusions	Matched
1008012	2	0.3514752	2	0
1008013	1	0.403486	0	1
1008014	1	0.4118643	6	1
1008015	2	0.4044858	1	1
1008016	1	0.8186131	0	0
1008017	2	0.8987589	0	0
1008018	2	0.9095091	0	0
1008019	1	0.8515652	0	1
1008020	2	0.7867695	2	1
1008021	2	0.7583805	0	0
1008022	1	0.8412643	2	1
1008023	2	0.9490839	0	0
1008024	2	0.56959	3	1
1008025	1	0.0436978	6	0
1008026	2	0.6765335	0	1
1008026	1	0.2992102	0	1
1008027	1	0.7010806	0	0
1008028	1	0.2495422	1	1
1008029	2	0.8733999	4	1
1008030	1	0.4509937	0	1
1008031	1	0.8143077	0	0
1008032	1	0.2822617	3	0
1008033	1	0.6143042	0	0
1008034	1	0.2000821	3	0
1008035	2	0.9898134	0	0
1008036	2	0.9603292	0	0
1008037	2	0.4813971	3	1
1008038	1	0.8410779	0	0
1008039	1	0.449652	0	1
1008040	1	0.810253	2	1
1008041	2	0.9256884	0	0
1008042	2	0.678968	0	1
1008043	2	0.9277758	0	0
1008044	1	0.9197193	0	1
1008045	2	0.588538	4	1
1008046	1	0.7153102	0	0
1008047	2	0.0989612	6	0
1008048	2	0.882295	0	0
1008048	1	0.7994878	0	0
1008049	2	0.8071992	0	0

Patient ID	Side	Propensity score	Number of tranfusions	Matched
1008050	2	0.9366489	0	0
1008051	1	0.6656533	0	1
1008052	1	0.8709424	0	0
1008053	1	0.3774182	2	1
1008054	2	0.0713257	4	0
1008055	1	0.8143077	0	0
1008056	2	0.8420542	2	1
1008057	2	0.7317822	0	1
1008058	1	0.8722229	0	0
1008059	2	0.5826166	2	1
1008060	1	0.8987589	0	0
1008060	2	0.9366737	0	0
1008061	2	0.9542825	0	0
1008062	1	0.8082396	2	1
1008063	2	0.8608724	0	0
1008064	1	0.0782198	4	0
1008065	2	0.6693853	2	1
1008066	1	0.6847599	1	1
1008067	1	0.9623802	0	0
1008067	2	0.9623802	0	0
1008068	2	0.7853797	2	1
1008069	1	0.9380204	0	0
1008070	2	0.8342246	0	0
1008071	2	0.9550222	0	0
1008072	1	0.6992193	2	1
1008073	2	0.1956367	3	0
1008074	2	0.8061834	0	0
1008075	1	0.1301977	0	1
1008076	1	0.6600526	0	0
1008077	2	0.9472872	0	0
1008078	1	0.846667	0	1
1008078	2	0.2784123	4	1
1008079	1	0.9796864	0	0
1008080	2	0.4580208	2	1
1008081	2	0.9163947	0	0
1008082	2	0.8505259	0	0
1008083	2	0.3774182	0	1
1008084	1	0.9340456	0	0
1008085	1	0.603985	4	1

Patient ID	Side	Propensity score	Number of tranfusions	Matched
1008086	1	0.0950477	2	0
1008087	1	0.2794593	2	0
1008088	1	0.9568595	2	1
1008089	1	0.9568595	0	0
1008090	1	0.8462635	0	0
1008091	1	0.9265721	0	0
1008092	2	0.7451044	0	0
1008093	1	0.5985009	2	1
1008094	2	0.0075015	4	0
1008095	1	0.8968255	0	0
1008096	1	0.9684105	0	0
1008097	1	0.2260023	3	1
1008097	2	0.6339748	4	1
1008098	1	0.9684105	0	0
1008099	1	0.3674133	2	1
1008100	2	0.547106	0	1
1008101	2	0.4584335	2	1
1008102	2	0.2472492	2	0
1008103	2	0.9527564	0	0
1008104	1	0.8003601	0	0
1008105	2	0.2925965	3	1
1008106	2	0.6958758	0	1
1008107	1	0.8823384	0	0
1008108	1	0.9000514	0	0
1008109	1	0.311248	3	1
1008110	2	0.8979311	0	0
1008111	1	0.7049051	1	1
1008112	2	0.5737743	2	1
1008113	1	0.9146721	0	1
1008113	2	0.9712601	0	0
1008114	1	0.6514034	2	1
1008115	1	0.6006756	5	1
1008116	1	0.9264762	0	0
1008116	2	0.9775355	0	0
1008117	2	0.4242065	0	1
1008118	2	0.8424735	4	1
1008118	1	0.6743881	0	1
1008119	1	0.0532364	2	0
1008120	1	0.9684361	0	0

Patient ID	Side	Propensity score	Number of tranfusions	Matched
1008121	2	0.6506863	0	0
1008122	2	0.9557483	0	0
1008123	2	0.9264762	0	0
1008123	1	0.9119953	0	1
1008124	1	0.6586223	0	0
1008125	2	0.3560629	2	1
1008126	2	0.8143077	2	1
1008127	2	0.3401774	2	0
1008127	1	0.5936798	2	1
1008128	1	0.56959	0	1
1008129	1	0.8923432	0	0
1008130	2	0.9653663	0	0
1008131	2	0.1303028	2	0
1008132	1	0.7845374	0	1
1008133	1	0.9550222	0	0
1008134	1	0.9493857	0	0
1008135	1	0.8342077	0	0
1008135	2	0.8938577	0	0
1008136	1	0.7838778	0	0
1008137	2	0.9336253	0	0
1008138	2	0.9142818	0	0
1008139	2	0.8583781	0	1
1008139	1	0.8583781	2	1
1008140	1	0.8729465	0	0
1008141	1	0.9380204	0	0
1008142	1	0.4796653	0	1
1008143	1	0.9555191	0	0
1008144	1	0.0361702	0	1
1008145	2	0.909169	0	0
1008146	2	0.3206316	2	0
1008147	2	0.263354	2	1
1008148	1	0.8569584	0	0
1008148	2	0.8569584	0	1
1008149	1	0.4530651	4	1
1008150	1	0.6210378	0	1
1008151	2	0.8569584	0	0
1008152	2	0.7059057	2	1
1008153	1	0.3477466	2	0
1008154	1	0.4132863	0	1

Patient ID	Side	Propensity score	Number of tranfusions	Matched
1008155	1	0.9543008	0	0
1008156	2	0.678968	0	0
1008157	1	0.6090651	5	1
1008158	2	0.9482519	0	0
1008159	1	0.8851865	0	0
1008160	1	0.7138674	2	1
1008161	2	0.122055	4	0
1008162	2	0.6820516	0	0
1008163	2	0.9398549	0	0
1008164	2	0.7854294	0	1
1008165	1	0.4631417	0	1
1008166	1	0.2067997	0	1
1008167	2	0.3897793	1	1
1008168	1	0.4254257	4	1
1008169	2	0.599822	0	1
1008169	1	0.599822	2	1
1008170	1	0.5202606	0	1
1008171	2	0.5057742	0	1
1008172	2	0.6298494	2	1
1008172	1	0.8380845	4	1
1008173	1	0.7853797	0	0
1008174	2	0.2952085	0	1
1008175	1	0.9611736	2	1
1008175	2	0.9684105	0	0
1008176	1	0.767398	0	0
1008177	1	0.9039453	0	0
1008177	2	0.9568595	0	0
1008178	1	0.8003601	0	0
1008179	1	0.2312134	0	1
1008180	2	0.2365066	2	1
1008181	1	0.0328589	2	0
1008182	1	0.8358088	0	0
1008183	2	0.9339128	0	0
1008184	1	0.9486418	0	0
1008185	2	0.8420542	2	1
1008186	2	0.1467373	0	1
1008187	1	0.3125997	0	1
1008188	2	0.9569282	0	0
1008189	1	0.5312783	2	1

Patient ID	Side	Propensity score	Number of tranfusions	Matched
1008190	1	0.9827961	0	0
1008191	2	0.3389104	2	0
1008192	2	0.9144774	0	0
1008193	2	0.9653663	0	0
1008194	1	0.88063	0	0
1008195	1	0.8464121	0	0
1008196	1	0.8651406	0	0
1008197	1	0.8151238	0	0
1008198	2	0.6586223	2	1
1008198	1	0.8724139	2	1
1008199	2	0.9398549	0	0
1008200	1	0.5021	4	1
1008201	1	0.0926708	1	1
1008202	2	0.5166272	0	1
1008203	2	0.1255288	1	0
1008204	2	0.4044299	3	0
1008205	1	0.7451044	0	0
1008206	2	0.4282673	2	0
1008207	1	0.0532364	2	0
1008208	2	0.6556749	0	1
1008209	2	0.4320413	2	1
1008210	2	0.7511338	0	0
1008211	2	0.511364	0	1
1008212	2	0.7494328	4	1
1008213	2	0.9811312	0	0
1008214	1	0.6565705	0	1
1008214	2	0.4733407	2	1
1008215	2	0.9768046	0	0
1008216	1	0.957775	0	0
1008217	1	0.7836873	0	0
1008218	1	0.9482519	0	0
1008219	1	0.9186629	0	0
1008220	1	0.242314	2	0
1008221	2	0.599822	0	1
1008222	1	0.9119742	0	0
1008223	2	0.9639877	0	0
1008224	1	0.8720679	0	0
1008225	2	0.6678334	0	1
1008226	2	0.9559048	0	0
1008227	1	0.8910959	0	0

Patient ID	Side	Propensity score	Number of tranfusions	Matched
1008228	2	0.914542	0	0
1008229	2	0.5579234	3	1
1008230	2	0.8023827	5	1
1008231	1	0.5930601	1	1
1008232	2	0.837696	0	1
1008232	1	0.8211572	0	0
1008233	1	0.9380691	0	0
1008233	2	0.8892304	0	0
1008234	2	0.1066127	12	0
1008235	2	0.8512704	0	0
1008236	1	0.467087	1	1
1008237	1	0.9555191	0	0
1008238	1	0.8857348	0	0
1008239	2	0.9684361	0	0
1008240	1	0.6846067	2	1
1008240	2	0.2283806	0	1
1008241	1	0.8823384	0	0
1008242	1	0.5901101	0	1
1008243	2	0.0224916	14	0
1008244	1	0.7978948	3	1
1008245	1	0.5453769	0	1
1008246	2	0.2685391	2	1
1008247	2	0.6696718	0	1
1008248	2	0.1011862	2	1
1008249	1	0.0120518	4	0
1008250	2	0.6435435	2	1
1008251	1	0.8823384	0	1
1008252	1	0.4415582	3	0
1008253	1	0.6089524	0	1
1008254	1	0.2282312	1	1
1008255	1	0.467087	1	1
1008256	2	0.6538066	0	1
1008257	2	0.2741648	3	1
1008258	1	0.9059667	0	1
1008258	2	0.7944445	0	0
1008259	1	0.599822	0	1
1008260	1	0.7164752	0	0
1008261	1	0.6339748	0	0
1008262	2	0.9342051	0	0

Patient ID	Side	Propensity score	Number of tranfusions	Matched
1008263	1	0.4673443	0	1
1008264	2	0.6109385	0	1
1008265	2	0.2384577	2	0
1008266	1	0.6909467	0	0
1008267	1	0.4437094	1	1
1008268	1	0.073114	4	0
1008269	1	0.5172633	0	1
1008269	2	0.425271	3	1
1008270	2	0.8303496	0	0
1008271	2	0.8720679	0	0
1008272	2	0.5575569	0	1
1008272	1	0.6031897	0	1
1008273	2	0.1690027	1	0
1008274	1	0.252009	3	0
1008275	1	0.8531178	0	0
1008276	2	0.5716151	4	1
1008277	2	0.7511338	0	0
1008278	1	0.918583	2	1
1008279	1	0.467087	4	1
1008280	2	0.8628565	0	0
1008281	2	0.4353576	0	1
1008282	2	0.6012156	1	1
1008283	1	0.4105438	0	1
1008284	2	0.5823425	0	0
1008285	2	0.5753093	0	1
1008286	1	0.9266286	0	0
1008287	1	0.9653663	0	0
1008288	1	0.9018397	0	0
1008289	1	0.7039941	2	1
1008290	1	0.7699598	4	1
1008291	2	0.8251629	0	0
1008292	2	0.5308863	0	1
1008293	2	0.7524539	0	0
1008294	2	0.9278036	0	0
1008295	1	0.8412643	0	0
1008295	2	0.9684105	0	0
1008296	2	0.3283026	2	0
1008297	2	0.9241018	0	0
1008297	1	0.9444106	0	0

Patient ID	Side	Propensity score	Number of tranfusions	Matched
1008298	1	0.5422639	2	1
1008299	2	0.3068618	2	1
1008300	1	0.9603292	0	0
1008301	1	0.693725	3	1
1008302	2	0.4417876	0	1
1008303	1	0.0176017	5	0
1008304	2	0.7149794	0	0
1008305	2	0.7348097	0	0
1008306	2	0.3311657	0	1
1008307	1	0.2690528	0	1
1008308	1	0.4522336	2	1
1008309	2	0.7564785	0	0
1008310	1	0.5303937	2	1
1008311	1	0.9197193	0	0
1008312	1	0.7451044	4	1
1008313	2	0.7783576	0	0
1008314	1	0.357722	3	0
1008314	2	0.8720679	0	0
1008315	1	0.4185981	2	0
1008316	1	0.2072138	1	0
1008317	1	0.3282734	0	1
1008318	2	0.3796384	0	1
1008318	1	0.5591499	1	1
1008319	1	0.6159767	0	1
1008320	2	0.8120872	0	0
1008321	2	0.1659552	0	1
1008322	2	0.416033	3	1
1008323	1	0.7175789	0	0
1008324	1	0.3811676	0	1
1008325	1	0.4825522	0	1
1008325	2	0.8036505	0	0
1008326	1	0.2711709	0	1
1008327	1	0.0331062	5	0
1008328	1	0.882295	0	0
1008328	2	0.678968	0	0
1008329	2	0.7785966	2	1
1008330	2	0.2860811	0	1
1008331	1	0.6006756	2	1
1008332	1	0.8720679	0	0

Patient ID	Side	Propensity score	Number of tranfusions	Matched
1008333	1	0.1349252	2	0
1008334	2	0.8963693	0	0
1008335	1	0.2696952	0	1
1008335	2	0.5575994	0	1
1008336	2	0.6506863	0	1
1008336	1	0.3760779	2	1
1008337	2	0.6265182	3	1
1008338	2	0.1566657	2	0
1008339	1	0.9568595	0	0
1008340	2	0.8186172	2	1
1008341	2	0.92748	0	0
1008341	1	0.92748	0	0
1008342	1	0.473773	0	1
1008343	2	0.8512704	0	0
1008343	1	0.9222556	0	0
1008344	1	0.3868049	2	1
1008345	1	0.5740564	0	1
1008346	1	0.6075645	2	1
1008347	1	0.1674851	0	1
1008348	2	0.9637995	0	0
1008349	2	0.3711177	2	0
1008350	1	0.3648572	0	1
1008351	1	0.6143042	0	0
1008352	2	0.0938651	1	0
1008353	1	0.447963	0	1
1008354	1	0.91067	0	0
1008355	1	0.8703418	0	1
1008356	1	0.806943	0	1
1008357	1	0.8583781	0	0
1008358	1	0.3427097	0	1
1008359	2	0.473773	2	1
1008360	1	0.9412272	0	0
1008360	2	0.9605683	0	0
1008361	2	0.3282734	2	0
1008362	2	0.9112114	0	0
1008363	2	0.2495422	3	0
1008364	2	0.1064048	4	0
1008365	1	0.5493338	0	1
1008366	2	0.916375	0	0

Patient ID	Side	Propensity score	Number of tranfusions	Matched
1008366	1	0.916375	0	0
1008367	1	0.467087	0	1
1008368	2	0.7699598	0	0
1008369	2	0.1570563	2	0
1008370	1	0.8345919	6	1
1008371	2	0.2350999	0	1
1008372	2	0.8817283	1	1
1008373	2	0.2214288	2	0
1008374	2	0.8926175	0	0
1008375	1	0.914542	0	0
1008376	1	0.5557157	1	1
1008377	1	0.9207713	0	0
1008378	2	0.6264054	0	1
1008379	2	0.6506863	0	1
1008380	1	0.2457842	3	1
1008381	2	0.9827961	0	0
1008382	1	0.1564605	6	0
1008383	1	0.4796653	1	1
1008384	2	0.321832	0	1
1008385	1	0.7785966	0	1
1008386	2	0.9395485	1	1
1008387	1	0.9039868	0	1
1008388	2	0.6807669	0	1
1008389	1	0.3001152	0	1
1008390	2	0.6804604	0	0
1008391	1	0.9775355	0	0
1008392	1	0.9557483	0	0
1008393	2	0.0554819	2	0
1008394	2	0.6349469	0	0
1008395	1	0.8798368	0	0
1008396	2	0.6311096	0	1
1008396	1	0.7245996	0	0
1008397	2	0.5716151	0	1
1008398	1	0.3068618	0	1
1008399	1	0.467087	0	1
1008400	2	0.9382355	0	0
1008401	1	0.9568595	0	0
1008402	1	0.4317516	0	1
1008402	2	0.4050255	0	1

Patient ID	Side	Propensity score	Number of tranfusions	Matched
1008403	2	0.8531178	0	0
1008404	2	0.9634501	4	1
1008405	1	0.663365	5	1
1008406	2	0.9557483	0	0
1008407	2	0.9387129	0	1
1008408	1	0.7797947	0	0
1008408	2	0.8143077	0	0
1008409	2	0.8569584	0	0
1008410	1	0.701459	0	0
1008411	1	0.6360197	2	1
1008412	1	0.701787	0	0
1008413	2	0.8160688	0	1
1008414	1	0.1803461	3	0
1008415	2	0.6564138	0	1
1008415	1	0.5167877	3	1
1008416	1	0.5585162	2	1
1008417	2	0.7564785	2	1
1008418	2	0.9402535	0	0
1008418	1	0.9639877	0	0
1008419	2	0.9835454	0	0
1008420	2	0.8931346	0	0
1008421	2	0.2972165	1	1
1008422	1	0.8023827	0	0
1008423	2	0.5049889	0	1
1008424	1	0.2710625	2	0
1008425	2	0.410127	2	1
1008426	1	0.4274115	0	1
1008427	1	0.8843137	0	0
1008428	2	0.6909467	0	1
1008429	2	0.0846227	5	0
1008430	1	0.7451044	0	0
1008431	1	0.122055	11	0
1008432	1	0.5085712	0	1
1008433	1	0.6489229	0	0
1008434	1	0.023686	5	0
1008435	2	0.9538277	0	0
1008436	2	0.5740564	1	1
1008437	2	0.599822	2	1
1008438	2	0.9243652	0	0
1008439	2	0.8979311	0	0

Patient ID	Side	Propensity score	Number of tranfusions	Matched
1008440	2	0.8909839	0	0
1008440	1	0.8683234	2	1
1008441	1	0.2171266	2	1
1008442	2	0.8720679	0	1
1008443	2	0.8584097	0	0
1008444	1	0.8630618	0	0
1008445	1	0.9675332	0	0
1008445	2	0.9743555	0	0
1008446	2	0.9568595	0	0
1008447	1	0.5029569	0	1
1008448	1	0.9482519	0	0
1008448	2	0.9482519	0	0
1008449	1	0.8987589	4	1
1008450	2	0.8182057	0	0
1008451	2	0.9197193	0	0
1008452	2	0.3784805	0	1
1008453	1	0.5936798	1	1
1008454	1	0.6291643	0	1
1008455	2	0.6368605	2	1
1008456	1	0.9607372	0	0
1008457	1	0.4298948	3	0
1008458	1	0.7299379	2	1
1008459	2	0.2396032	0	1
1008460	1	0.6121385	2	1
1008461	1	0.7398223	2	1
1008462	2	0.8924138	0	0
1008463	1	0.6544533	1	1
1008464	1	0.8923432	0	0
1008465	1	0.764659	0	0
1008466	1	0.8983983	0	0
1008467	2	0.8372281	0	0
1008468	2	0.3412865	2	1
1008469	1	0.8892025	0	0
1008470	2	0.2286018	1	1
1008470	1	0.7838778	0	0
1008471	2	0.8160688	0	0
1008472	1	0.2395199	2	1
1008473	1	0.0262188	10	0
1008474	1	0.2435291	8	0

Patient ID	Side	Propensity score	Number of tranfusions	Matched
1008474	2	0.4245174	0	1
1008475	2	0.7658537	0	0
1008476	2	0.1698545	0	1
1008477	1	0.6790591	4	1
1008478	1	0.9322666	2	1
1008478	2	0.9607499	0	1
1008479	2	0.7699598	0	1
1008480	1	0.8867568	0	1
1008481	1	0.9471208	2	1
1008482	2	0.9078221	2	1
1008483	2	0.8462635	0	0
1008483	1	0.9278036	0	0
1008484	1	0.8583781	1	1
1008484	2	0.8143077	0	0
1008485	2	0.8531178	0	0
1008486	1	0.8103819	0	0
1008487	1	0.9050807	0	0
1008488	1	0.5882164	0	1
1008489	1	0.622145	0	0
1008489	2	0.9071223	0	0
1008490	2	0.7511338	1	1
1008491	1	0.5797566	2	1
1008491	2	0.2801864	0	1
1008492	1	0.3045313	5	0
1008493	1	0.9103201	0	0
1008494	2	0.4921914	4	1
1008495	1	0.8720679	2	1
1008496	2	0.5948547	0	0
1008497	1	0.3342481	0	1
1008497	2	0.3833727	0	1
1008498	1	0.9675201	0	0
1008499	2	0.6130926	4	1
1008500	2	0.6468954	0	1
1008501	2	0.8131691	0	1
1008501	1	0.9493857	0	0
1008502	1	0.6375563	1	1
1008503	2	0.678968	0	0
1008504	1	0.6844349	0	0
1008505	1	0.6370063	0	1
1008506	2	0.9000514	0	0

Patient ID	Side	Propensity score	Number of tranfusions	Matched
1008506	1	0.8268883	0	0
1008507	1	0.4144451	4	1
1008508	1	0.2825076	19	1
1008509	2	0.7950629	0	1
1008510	1	0.5557687	0	1
1008511	1	0.8531178	0	0
1008512	1	0.9166842	0	0
1008513	2	0.6909467	0	0
1008514	1	0.9546245	0	0
1008515	2	0.2550914	2	1
1008516	2	0.9568595	0	0
1008517	1	0.6444111	0	0
1008518	1	0.9222556	0	0
1008519	2	0.6844686	2	1
1008520	2	0.6556749	0	1
1008520	1	0.7022124	0	0
1008521	2	0.0121565	11	0
1008522	1	0.4607009	4	1
1008522	2	0.6319291	2	1
1008523	1	0.885538	0	0
1008524	2	0.9555191	0	0
1008524	1	0.9555191	0	0
1008525	1	0.2341951	2	1
1008526	1	0.8590258	0	1
1008527	2	0.9067378	0	0
1008528	1	0.8893129	0	0
1008529	1	0.8843186	0	0
1008530	1	0.2992102	2	1
1008531	2	0.9637995	0	0
1008532	2	0.0565426	7	1
1008533	2	0.1394752	2	1
1008534	2	0.9024512	0	0
1008535	2	0.7790663	0	0
1008536	2	0.6210378	2	1
1008537	1	0.8823384	0	0
1008538	2	0.9339128	0	0
1008539	2	0.6790591	0	1
1008540	1	0.4735617	2	1
1008541	2	0.2592462	4	0

Patient ID	Side	Propensity score	Number of tranfusions	Matched
1008542	1	0.6319291	4	1
1008543	2	0.9623802	0	0
1008544	2	0.4006762	0	1
1008544	1	0.6977639	0	0
1008545	2	0.1396133	0	1
1008546	1	0.9684105	0	1
1008547	2	0.9405539	0	0
1008547	1	0.9656805	0	0
1008548	1	0.5436055	0	0
1008549	1	0.9262035	0	0
1008550	2	0.7511338	0	1
1008551	2	0.8251417	3	1
1008552	2	0.8380845	0	1
1008552	1	0.8380845	0	0
1008553	1	0.7258901	1	1
1008554	2	0.3915087	4	0
1008555	2	0.9133362	0	1
1008555	1	0.9133362	2	1
1008556	1	0.1913898	2	0
1008557	2	0.9637995	0	0
1008557	1	0.9637995	0	0
1008558	2	0.6462542	2	1
1008558	1	0.5056091	0	1
1008559	1	0.9380204	0	1
1008560	1	0.1257737	6	0
1008561	2	0.7315358	0	0
1008562	2	0.9684149	0	0
1008563	1	0.7520779	0	0
1008564	2	0.9145093	0	0
1008564	1	0.9145093	0	0
1008565	1	0.9754074	0	0
1008566	1	0.2341951	0	1
1008567	1	0.6210378	0	1
1008567	2	0.9000514	0	0
1008568	1	0.6790591	2	1
1008569	1	0.9342051	0	0
1008569	2	0.9342051	0	0
1008570	2	0.4346611	2	1
1008571	2	0.7341295	0	0

Patient ID	Side	Propensity score	Number of tranfusions	Matched
1008572	1	0.0558041	0	1
1008573	1	0.1205342	23	0
1008574	2	0.7731047	0	1
1008575	2	0.7010806	0	0
1008576	1	0.3065288	2	1
1008577	1	0.7454769	0	0
1008578	1	0.4209728	0	1
1008579	2	0.8923432	0	0
1008580	2	0.2240943	2	1
1008581	1	0.8838318	0	0
1008582	2	0.8979311	0	0
1008582	1	0.8979311	0	1
1008583	2	0.5930601	2	1
1008584	1	0.9555191	0	0
1008585	1	0.926904	2	1
1008586	1	0.963065	0	0
1008587	2	0.8719391	1	1
1008588	1	0.8740013	0	1
1008589	2	0.50196	0	1
1008590	2	0.7669336	2	1
1008591	2	0.8326454	0	0
1008592	1	0.9119742	0	0
1008593	2	0.8250997	0	1
1008594	2	0.683496	2	1
1008594	1	0.1395241	0	1
1008595	1	0.5607811	0	1
1008596	2	0.8380845	0	0
1008597	2	0.8740013	7	1
1008597	1	0.8541505	0	1
1008598	2	0.8923432	0	1
1008599	2	0.6454555	1	1
1008600	2	0.9482724	0	0
1008601	1	0.9070476	0	0
1008602	2	0.6402105	2	1
1008603	1	0.8151238	0	0
1008604	1	0.6210378	0	1
1008605	1	0.7613885	0	1
1008606	2	0.9315427	0	0
1008607	1	0.6629275	0	1

Patient ID	Side	Propensity score	Number of tranfusions	Matched
1008608	2	0.9448927	0	0
1008608	1	0.9448927	0	0
1008609	1	0.928416	0	0
1008610	1	0.8987589	0	0
1008611	2	0.9387549	0	0
1008612	1	0.8390119	0	0
1008613	1	0.9835454	0	0
1008614	2	0.4795538	0	1
1008615	2	0.8664521	0	0
1008616	1	0.9568595	0	0
1008617	1	0.9024935	0	0
1008618	2	0.6100065	0	1
1008619	1	0.2852989	2	1
1008619	2	0.2852989	0	1
1008620	2	0.2525808	2	1
1008621	1	0.9000514	0	0
1008622	1	0.9568595	0	0
1008623	1	0.6074143	3	1
1008624	2	0.6199751	0	1
1008625	2	0.6743881	0	1
1008626	1	0.5882676	0	1
1008627	2	0.3868049	2	1
1008628	2	0.7893384	0	0
1008629	1	0.8380845	0	0
1008630	1	0.7145118	0	1
1008631	1	0.6792615	0	0
1008632	1	0.4111889	5	1
1008633	1	0.7370072	2	1
1008634	2	0.5575969	2	1
1008635	2	0.9743555	0	0
1008636	1	0.8615083	0	0
1008637	1	0.9490839	0	0
1008638	1	0.9684105	0	0
1008639	1	0.0714496	6	0
1008640	1	0.6624998	0	0
1008641	1	0.7622421	0	0
1008642	2	0.8628989	0	0
1008643	2	0.8443047	0	0
1008644	2	0.4221659	3	1

Patient ID	Side	Propensity score	Number of tranfusions	Matched
1008645	1	0.755473	1	1
1008646	1	0.0781541	2	0
1008647	1	0.1136335	5	0
1008648	2	0.9898134	0	0
1008649	1	0.9366489	0	0
1008650	2	0.9068288	0	0
1008651	2	0.9557483	4	1
1008652	2	0.3992149	2	1
1008653	1	0.744074	0	0
1008654	2	0.9366248	0	0
1008654	1	0.9481907	0	0
1008655	1	0.7400366	2	1
1008656	1	0.3601328	2	1
1008657	2	0.8328993	0	0
1008658	1	0.1859022	5	0
1008659	2	0.857636	8	1
1008659	1	0.8848014	2	1
1008660	1	0.9706023	0	0
1008661	1	0.7899556	3	1
1008662	1	0.8548512	1	1
1008663	2	0.9366489	0	0
1008664	1	0.3275327	10	1
1008665	1	0.5002404	0	1
1008666	1	0.8923432	0	0
1008667	1	0.8303496	0	1
1008668	2	0.8531178	0	0
1008669	1	0.5628302	0	0
1008669	2	0.8569584	2	1
1008670	2	0.5621313	4	1
1008671	1	0.6506863	0	0
1008672	1	0.7258572	2	1
1008673	2	0.3761384	5	1
1008674	2	0.9281372	0	1
1008675	2	0.3683397	0	1
1008676	1	0.8965563	0	0
1008677	2	0.9835454	0	0
1008678	2	0.3400615	2	0
1008679	1	0.8807684	0	0
1008680	1	0.1708095	2	0

Patient ID	Side	Propensity score	Number of tranfusions	Matched
1008681	1	0.9862315	0	0
1008682	1	0.5312783	0	1
1008683	2	0.0509298	6	0
1008684	1	0.6839073	3	1
1008685	2	0.3520014	2	1
1008686	1	0.020908	14	0
1008687	2	0.9180547	2	1
1008688	2	0.6210378	2	1
1008689	1	0.8793957	0	0
1008690	1	0.9364664	0	0
1008691	1	0.6327443	0	0
1008692	1	0.7268211	0	1
1008693	1	0.8615083	0	0
1008694	2	0.6576642	1	1
1008695	1	0.8720679	0	0
1008696	2	0.9605683	0	0
1008697	1	0.9512951	0	0
1008698	2	0.7010806	0	0
1008699	1	0.7999699	2	1
1008700	2	0.8955071	2	1
1008701	1	0.9471623	0	0
1008702	1	0.8357238	0	0
1008702	2	0.728705	0	0
1008703	1	0.8268883	0	0
1008703	2	0.9163173	0	0
1008704	1	0.7010806	0	0
1008705	2	0.4593293	2	1
1008705	1	0.4006373	2	1
1008706	2	0.3432235	2	1
1008707	2	0.9372897	0	0
1008708	2	0.9482519	0	0
1008709	1	0.4271558	2	1
1008709	2	0.5591499	2	1
1008710	1	0.5716151	0	1
1008711	1	0.9546245	0	0
1008712	2	0.4603808	0	1
1008713	2	0.9166842	2	1
1008714	1	0.3880984	0	1
1008715	2	0.9835454	0	0

Patient ID	Side	Propensity score	Number of tranfusions	Matched
1008716	1	0.3065288	0	1
1008717	2	0.6375563	0	1
1008718	1	0.8532444	0	1
1008719	1	0.6790591	0	1
1008719	2	0.8703418	2	1
1008720	1	0.4426782	2	0
1008721	1	0.5990015	0	1
1008721	2	0.755473	0	1
1008722	2	0.9457731	0	0
1008723	2	0.7256267	0	1
1008724	1	0.8787343	0	1
1008725	2	0.755339	0	1
1008726	2	0.1223756	2	0
1008727	1	0.1681965	3	0
1008728	1	0.7004812	0	0
1008729	1	0.4456008	0	1
1008730	1	0.56959	0	1
1008731	2	0.9154188	0	0
1008732	2	0.9135031	0	0
1008733	1	0.3575094	0	1
1008734	2	0.9486418	0	0
1008735	2	0.9431339	0	0
1008736	2	0.9330049	0	0
1008737	2	0.7472676	0	0
1008738	1	0.51449	2	1
1008739	2	0.7151394	0	1
1008740	2	0.4638417	0	1
1008741	1	0.0297698	6	0
1008742	2	0.8143077	0	0
1008743	2	0.6631602	0	0
1008744	2	0.8825185	0	0
1008745	1	0.6660834	2	1
1008746	1	0.4524742	6	1
1008747	1	0.6003122	12	1
1008748	1	0.4528818	0	1
1008749	1	0.9285819	0	0
1008750	2	0.6210378	2	1
1008751	2	0.4349635	1	0
1008752	1	0.7838778	0	0

Patient ID	Side	Propensity score	Number of tranfusions	Matched
1008753	1	0.6557096	2	1
1008754	2	0.1910429	8	0
1008755	2	0.7175789	0	0
1008756	2	0.693725	0	0
1008757	1	0.8403986	0	0
1008758	1	0.7672793	0	0
1008759	1	0.9086713	0	0
1008760	2	0.1230849	4	0
1008761	2	0.9070476	0	0
1008762	2	0.5795027	0	1
1008763	2	0.8531178	0	0
1008764	2	0.8987589	0	0
1008765	2	0.6111393	0	1
1008766	2	0.3351131	2	1
1008767	1	0.3795239	2	0
1008768	1	0.8485192	0	0
1008769	2	0.8033172	2	1
1008770	1	0.6210378	2	1
1008771	2	0.7816533	2	1
1008772	1	0.925874	0	0
1008773	1	0.8771182	0	0
1008774	2	0.9173771	0	0
1008775	2	0.7871609	2	1
1008776	1	0.4676726	0	1
1008777	1	0.7992524	0	1
1008778	2	0.321832	3	1
1008779	1	0.1216806	3	0
1008780	1	0.8220941	0	0
1008781	1	0.810253	0	0
1008782	1	0.4796653	0	1
1008783	1	0.8151695	3	1
1008784	2	0.9550222	0	0
1008784	1	0.9756276	0	0
1008785	2	0.6626641	0	1
1008786	2	0.8740013	0	1
1008787	1	0.7797947	0	1
1008788	1	0.473773	0	1
1008789	2	0.9372145	0	0
1008790	1	0.9186629	0	0
1008791	1	0.1508069	6	0

Patient ID	Side	Propensity score	Number of tranfusions	Matched
1008792	1	0.5307699	0	1
1008793	2	0.3400615	1	0
1008794	1	0.1548002	0	1
1008795	2	0.8061834	0	0
1008796	2	0.5797566	0	1
1008797	2	0.5575969	2	1
1008798	1	0.034326	11	0
1008799	1	0.9671038	0	0
1008800	1	0.8143077	0	0
1008800	2	0.8143077	0	0
1008801	2	0.755473	0	1
1008802	2	0.473773	5	1
1008803	2	0.4332378	1	0
1008804	1	0.9166842	0	0
1008804	2	0.9743555	0	0
1008805	1	0.678968	0	0
1008806	1	0.9508042	0	0
1008807	1	0.6210378	0	1
1008808	1	0.3133725	1	0
1008809	2	0.810253	0	0
1008810	1	0.9639877	0	0
1008811	1	0.0855581	6	0
1008812	1	0.6938656	0	1
1008813	2	0.6704192	2	1
1008814	1	0.5236873	3	1
1008815	1	0.6898651	0	0
1008816	1	0.7281684	0	1
1008817	1	0.7836873	0	0
1008818	2	0.1124742	4	0
1008819	2	0.9682459	0	0
1008820	2	0.9723309	2	1
1008821	1	0.4037822	0	1
1008822	1	0.8143077	2	1
1008823	1	0.7999699	0	1
1008824	1	0.8143077	0	0
1008825	1	0.8151675	0	1
1008826	1	0.1621539	2	0
1008827	1	0.7797947	0	0
1008828	2	0.6288589	2	1
1008829	2	0.5092125	0	1

Patient ID	Side	Propensity score	Number of tranfusions	Matched
1008830	2	0.6100852	0	1
1008831	1	0.8841555	0	0
1008832	2	0.9624106	0	0
1008833	2	0.6500681	0	1
1008834	1	0.9585671	0	0
1008835	1	0.6210378	2	1
1008836	1	0.4349635	0	1
1008837	1	0.9398549	0	0
1008838	1	0.1947112	11	0
1008839	1	0.5740564	0	1
1008840	2	0.6177947	0	1
1008841	2	0.2492797	0	1
1008842	1	0.745027	0	0
1008843	1	0.7656533	0	1
1008844	1	0.6466385	0	0
1008845	1	0.8759232	2	1
1008846	2	0.844758	0	0
1008847	1	0.321832	2	1
1008848	1	0.8512175	0	0
1008849	2	0.9555191	0	0
1008850	2	0.4220087	0	1
1008851	2	0.8410779	0	0
1008852	2	0.5231775	0	1
1008853	1	0.4549305	3	1
1008854	1	0.6489229	0	1
1008855	2	0.678968	0	0
1008856	1	0.8443209	0	0
1008857	2	0.8622236	0	0
1008858	1	0.1548002	4	0
1008859	2	0.6581494	0	0
1008860	2	0.397871	2	0
1008861	2	0.8143077	0	0
1008862	1	0.7010806	0	1
1008863	1	0.3588556	0	1
1008864	1	0.0979794	1	0
1008865	1	0.9512951	0	0
1008866	2	0.7142609	9	1
1008867	2	0.8861318	0	1
1008868	2	0.2550914	2	0

Patient ID	Side	Propensity score	Number of tranfusions	Matched
1008869	1	0.9454951	0	0
1008870	1	0.7470569	0	1
1008871	1	0.6433478	2	1
1008872	2	0.9684105	0	0
1008873	2	0.7853797	0	0
1008874	2	0.1548002	3	1
1008875	2	0.7369108	0	1
1008876	2	0.2191047	2	0
1008877	2	0.5375759	0	1
1008878	2	0.6538066	0	0
1008879	2	0.6210378	0	1
1008880	2	0.1987473	1	1
1008881	2	0.678968	0	0
1008882	1	0.2457842	0	1
1008883	2	0.9684105	0	0
1008884	2	0.242314	0	1
1008885	1	0.559449	0	1
1008886	1	0.2709859	2	1
1008887	1	0.1658363	4	1
1008888	1	0.9706023	0	0
1008889	1	0.8410779	0	0
1008890	1	0.8720679	0	0
1008891	2	0.7400587	0	0
1008892	1	0.7663047	0	1
1008893	2	0.3632663	0	1
1008894	2	0.4415582	0	1
1008895	1	0.5432829	3	1
1008896	1	0.410127	2	1
1008897	2	0.56959	0	1
1008898	1	0.824614	0	0
1008899	2	0.9030837	0	1
1008900	2	0.678968	0	1
1008901	1	0.4006762	0	1
1008902	2	0.9398549	0	0
1008903	2	0.8326454	0	0
1008904	2	0.7010806	0	0
1008905	1	0.4638417	4	1
1008906	2	0.9499006	0	0
1008906	1	0.7454145	0	0

Patient ID	Side	Propensity score	Number of tranfusions	Matched
1008907	2	0.8601149	0	0
1008908	1	0.2874452	4	0
1008909	1	0.8199402	0	0
1008910	1	0.9653663	0	0
1008911	2	0.6271352	0	1
1008911	1	0.8242614	0	0
1008912	1	0.8082396	0	1
1008913	2	0.9684105	2	1
1008914	2	0.2363879	2	0
1008915	2	0.9568595	0	0
1008916	1	0.9467434	0	0
1008917	2	0.7838778	0	0
1008918	2	0.928416	0	0
1008919	1	0.7266501	0	1
1008920	2	0.5435919	4	1
1008921	2	0.9603292	0	0
1008922	2	0.1049629	9	1
1008923	1	0.6305341	0	1
1008924	2	0.6349738	2	1
1008925	2	0.8976958	0	0
1008925	1	0.9680528	0	0
1008926	1	0.730173	0	0
1008926	2	0.8429886	0	0
1008927	1	0.7199393	0	1
1008928	1	0.9527564	0	0
1008929	1	0.2024319	2	0
1008930	2	0.9112114	0	0
1008931	2	0.9482724	0	0
1008932	1	0.9016513	0	0
1008933	1	0.9482519	0	0
1008934	1	0.2345615	9	1
1008935	1	0.8698594	0	1
1008936	1	0.242314	0	1
1008937	2	0.1256096	2	0
1008938	1	0.559449	2	1
1008939	1	0.6339748	0	1
1008939	2	0.3282734	2	0
1008940	1	0.5307699	0	1
1008941	2	0.4987304	0	1
1008941	1	0.5041998	0	1

Patient ID	Side	Propensity score	Number of tranfusions	Matched
1008942	1	0.9306491	0	0
1008943	2	0.9653663	0	0
1008944	2	0.9557483	2	1
1008945	1	0.9191106	0	0
1008946	1	0.7158842	0	0
1008947	2	0.6618387	1	1
1008948	2	0.9559048	0	0
1008949	1	0.8838318	0	0
1008950	2	0.9395485	0	0
1008951	2	0.0327426	11	0
1008952	1	0.4209728	0	1
1008953	1	0.4733407	2	1
1008954	1	0.7138674	0	0
1008954	2	0.467087	2	1
1008955	1	0.9278036	0	0
1008956	2	0.4473092	1	1
1008957	2	0.7175789	0	1
1008958	2	0.4484059	3	1
1008959	2	0.139166	0	1
1008960	1	0.511364	0	1
1008961	1	0.9040454	0	0
1008962	1	0.5400624	1	1
1008963	2	0.948741	0	0
1008964	2	0.9555191	0	0
1008965	1	0.8825185	0	0
1008965	2	0.8583781	0	0
1008966	2	0.1774018	2	0
1008967	1	0.957775	0	0
1008968	1	0.7022124	0	0
1008969	1	0.8199402	0	0
1008970	1	0.0517391	2	0
1008971	1	0.6822033	0	0
1008972	2	0.9639877	0	0
1008973	1	0.9639877	0	0
1008974	1	0.7696973	4	1
1008975	2	0.8380845	0	0
1008976	1	0.9493857	1	1
1008977	1	0.678968	0	0
1008978	2	0.7171246	0	0

Patient ID	Side	Propensity score	Number of tranfusions	Matched
1008979	1	0.7471596	0	0
1008980	1	0.8182057	0	0
1008981	1	0.9386415	0	0
1008982	1	0.9050807	0	0
1008983	1	0.7978948	1	1
1008984	2	0.2398293	2	1
1008985	2	0.148605	2	0
1008986	1	0.9059724	0	0
1008987	1	0.8700188	0	0
1008988	1	0.8199402	2	1
1008988	2	0.7587227	0	1
1008989	1	0.9395485	0	0
1008990	1	0.0147431	11	0
1008991	1	0.7558137	0	1
1008991	2	0.7558137	0	1
1008992	1	0.6870216	2	1
1008993	2	0.8569771	0	1
1008994	2	0.2408308	0	1
1008994	1	0.3468054	0	1
1008995	2	0.3318409	1	1
1008996	1	0.6804478	0	0
1008997	2	0.7436793	0	0
1008997	1	0.8454359	0	0
1008998	1	0.6339748	0	1
1008999	2	0.9278036	0	0
1009000	2	0.9493857	0	0
1009000	1	0.9493857	0	0
1009001	1	0.3571163	0	1
1009002	1	0.4658061	0	1
1009003	1	0.6723223	0	1
1009004	2	0.7010806	0	0
1009005	2	0.7885835	2	1
1009006	1	0.766746	0	1
1009007	2	0.948741	4	1
1009008	1	0.6506863	0	0
1009009	1	0.9653663	0	0
1009010	2	0.9568595	0	0
1009011	2	0.3451385	5	1
1009012	1	0.9004673	0	0
1009013	1	0.5703638	0	1

Patient ID	Side	Propensity score	Number of tranfusions	Matched
1009014	1	0.8151238	0	0
1009015	2	0.7451044	2	1
1009015	1	0.4375402	2	1
1009016	1	0.9653663	0	0
1009017	2	0.5985009	0	1
1009017	1	0.8983983	0	0
1009018	2	0.1739724	10	0
1009019	2	0.9380204	0	1
1009020	2	0.9607372	2	1
1009021	1	0.6179632	0	1
1009022	1	0.9398549	0	0
1009023	2	0.3049089	1	0
1009024	1	0.6103809	0	1
1009025	2	0.7051151	0	1
1009026	1	0.6504241	0	1
1009027	1	0.9573394	0	0
1009028	2	0.5359595	0	1
1009029	1	0.1620448	9	0
1009030	1	0.1267193	2	0
1009031	2	0.9154188	0	0
1009032	1	0.8148102	0	0
1009033	2	0.9120077	0	0
1009033	1	0.9454951	0	1
1009034	1	0.475834	2	1
1009035	1	0.9653663	0	0
1009036	1	0.0828412	4	0
1009037	2	0.8143077	0	0
1009038	1	0.5307699	4	1
1009039	2	0.9151475	0	0
1009040	2	0.1548002	5	0
1009041	2	0.1568261	2	0
1009042	1	0.8615083	0	0
1009043	1	0.693725	0	0
1009044	1	0.9827961	0	0
1009045	1	0.8436325	0	1
1009046	1	0.2525808	2	1
1009047	1	0.8372281	0	0
1009048	1	0.6349469	0	0
1009049	2	0.0198193	16	0

Patient ID	Side	Propensity score	Number of tranfusions	Matched
1009050	1	0.9706023	0	0
1009051	1	0.9570311	0	0
